# Supplementary figures and images for: Critical role of the BAF chromatin remodeling complex during murine neural crest development
Source: PLoS Genet. 2021 Mar 22;17(3):e1009446. doi: 10.1371/journal.pgen.1009446 (PMC8016319; doi:10.1371/journal.pgen.1009446)

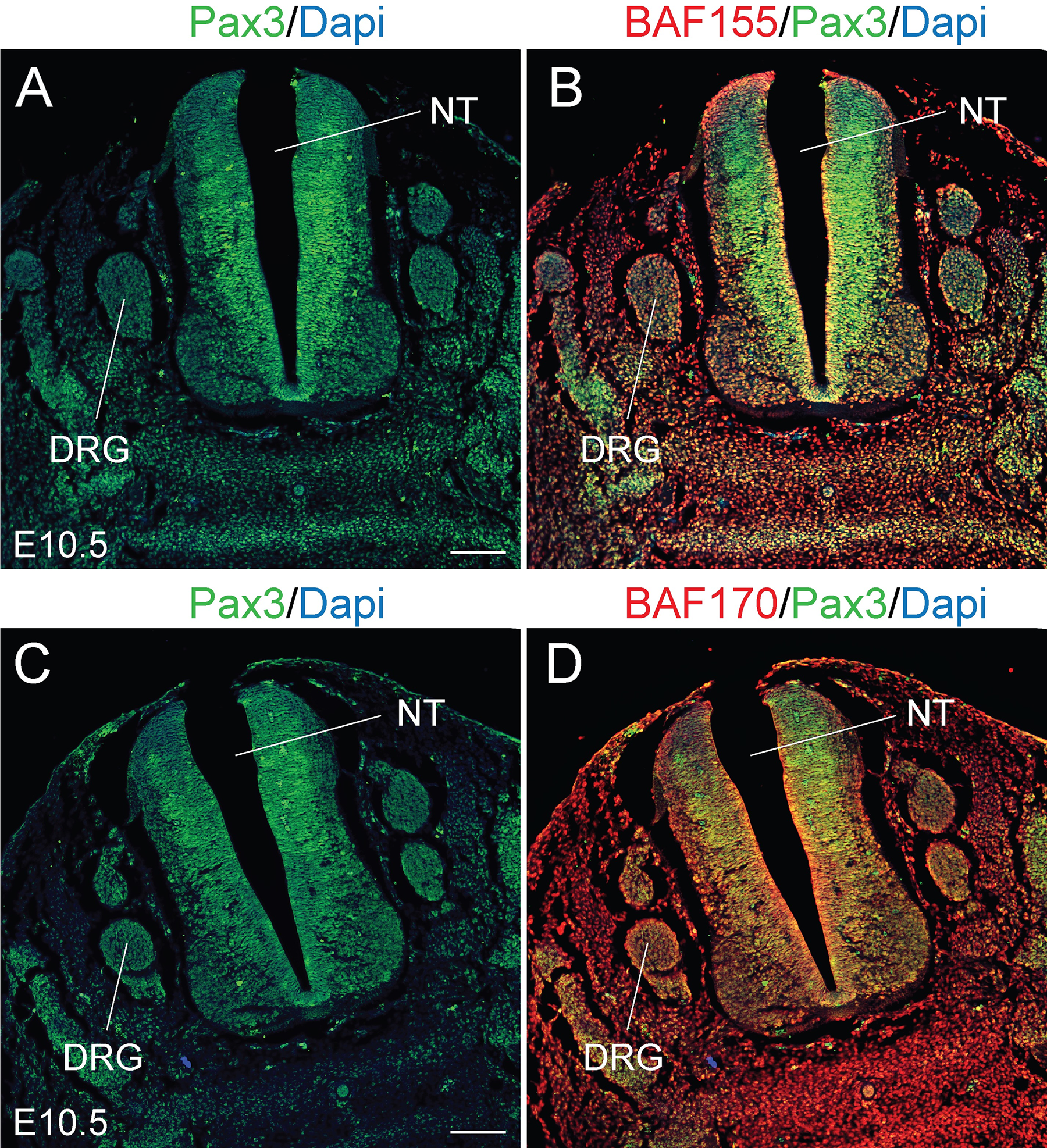

Supplement: S1 Fig — (A-B) Double immunostaining for BAF155 and Pax3 was performed on E10.5 transverse sections. (C-D) Double immunostaining for BAF170 and Pax3 was performed on E10.5 transverse sections. Scale bars 100μM. NT, Neural tube, DRG, dorsal root ganglion. (TIF) [file pgen.1009446.s001.tif]

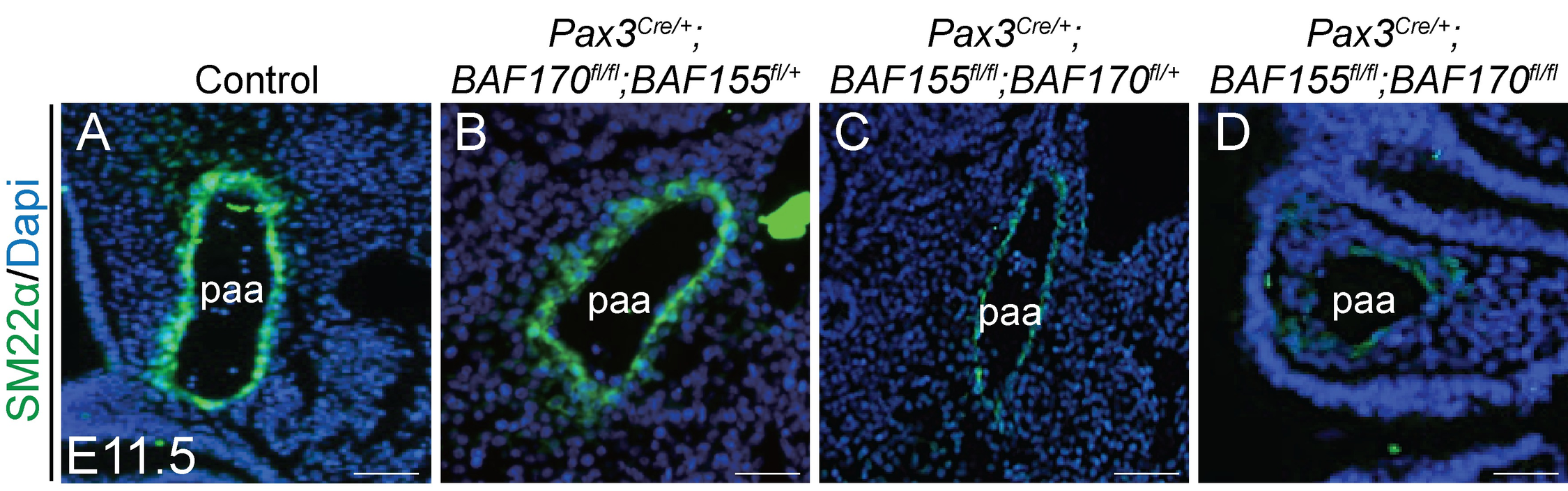

Supplement: S2 Fig — (A-D) Immunostaining for SM22α on the frontal section from E11.5 control, BAF170-deficient (Pax3Cre/+;BAF170fl/fl;BAF155fl/+), BAF155-deficient (Pax3Cre/+;BAF155fl/fl;BAF170fl/+) and BAF155/170-deficient (Pax3Cre/+;BAF155fl/fl;BAF170fl/fl) embryos (n = 3–4 for each genotype). Nuclei were visualized by Dapi staining (blue). 4th Pharyngeal arch artery (paa) is shown. Scale bar 50μM (A-D). (TIF) [file pgen.1009446.s002.tif]

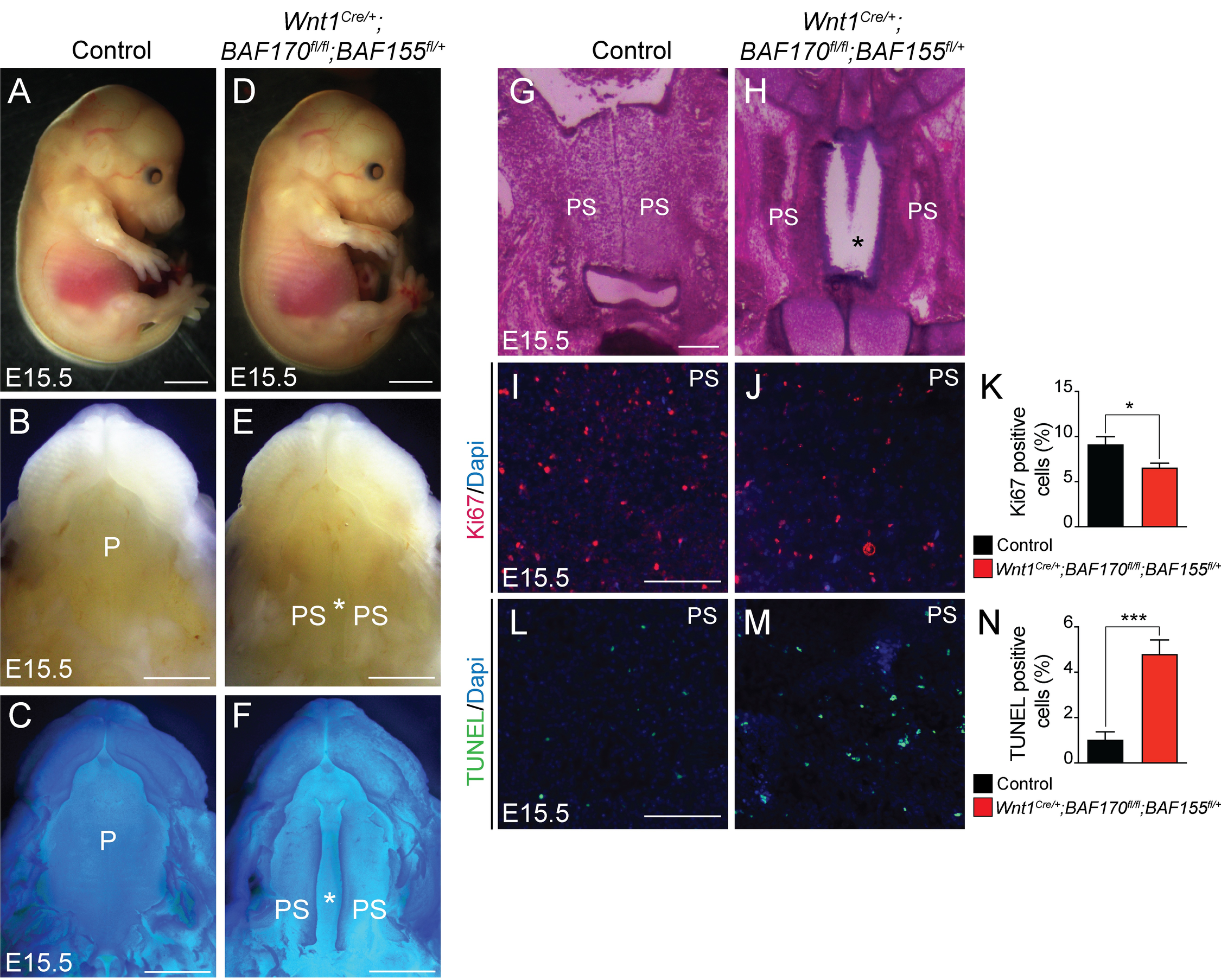

Supplement: S3 Fig — (A-F) To analyze the palates, E15.5 control (A-C, n = 4) and BAF170-deficient (Wnt1Cre/+;BAF170fl/fl;BAF155fl/+) embryos (D-F, n = 4) were harvested. No obvious defects were observed by whole embryos morphology (A and D). Compared to the controls, palates were not fused in the BAF170-deficient (Wnt1Cre/+;BAF170fl/fl;BAF155fl/+) embryos (B and E). Alcian blue staining showed cleft palate in the BAF170-deficient (Wnt1Cre/+;BAF170fl/fl;BAF155fl/+) embryos (C and F). (G-H) H&E stained palate sections of control (G) and BAF170-deficient (H) embryos. (I-K) Immunohistochemistry for Ki67 on palate sections of control (I) and BAF170-deficient (J) embryos and quantification (K). (L-N) TUNEL staining on palate sections of control (L) and BAF170-deficient (M) embryos and quantification (N). Nuclei were visualized by DAPI staining (blue). Asterisks (*) represent cleft palate in BAF170-deficient embryos (E, F and H). P, Palate; PS, Palatal shelves. Values are reported as means ± SEM (*P < 0.05, **P < 0.01, ***P < 0.001; NS, not significant). Scale bar 1mM (A and D). Scale bar 500μM (B-C and E-F). Scale bar 100μM (G-M). (TIF) [file pgen.1009446.s003.tif]

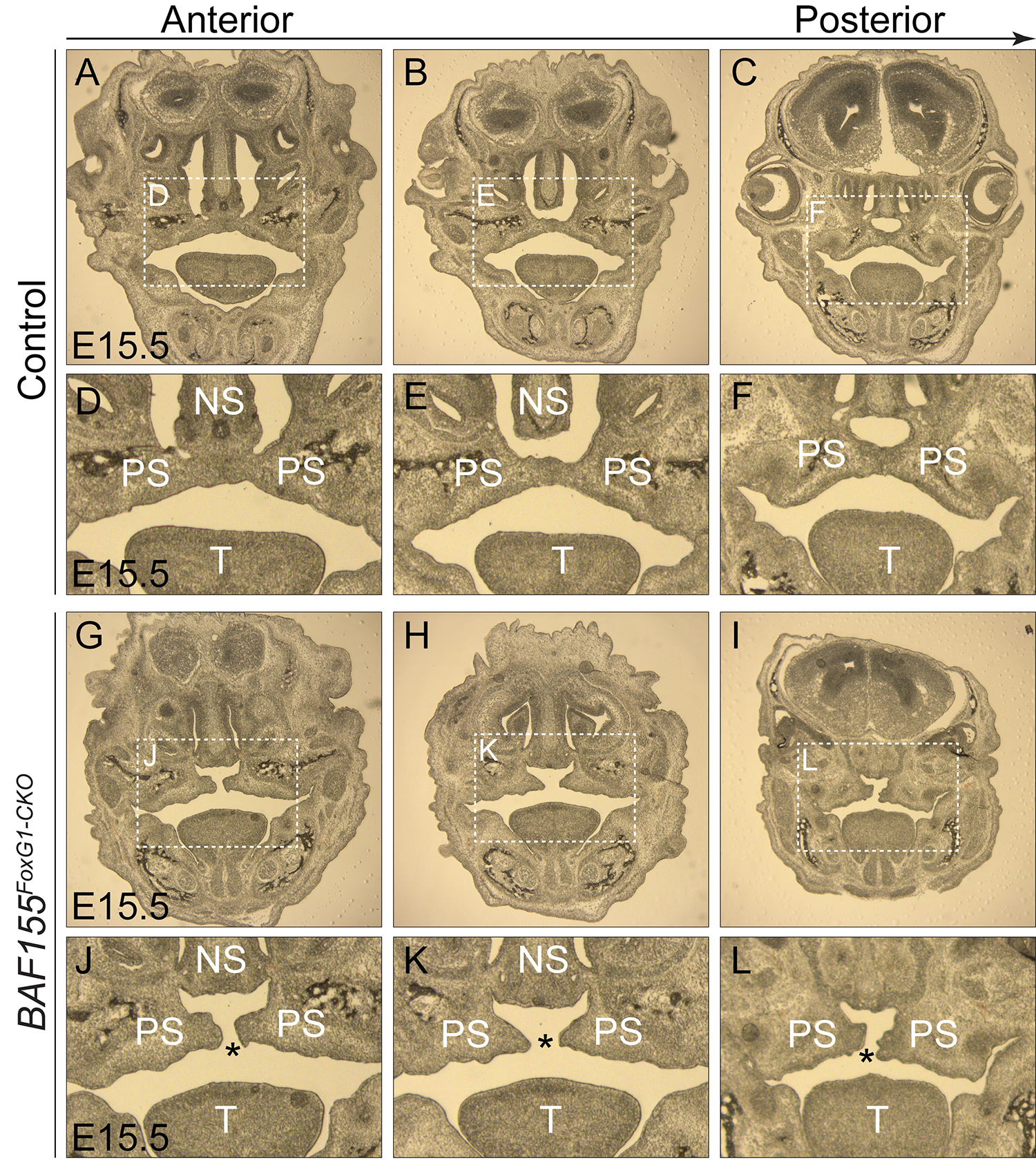

Supplement: S4 Fig — (A-L) Histological analysis of E15.5 control (A-F) and BAF155FoxG1-CKO (FoxG1Cre/+;BAF155fl/fl) (G-L) embryos (n = 3 controls, n = 4 BAF155FoxG1-CKO). Transverse sections of control and BAF155FoxG1-CKO embryos at the level of the anterior to posterior palatal shelves were analyzed. Asterisks (*) represent cleft palate in BAF155FoxG1-CKO embryos (J-L). PS, Palatal shelves; NS, Nasal septum; T, Tongue. (TIF) [file pgen.1009446.s004.tif]

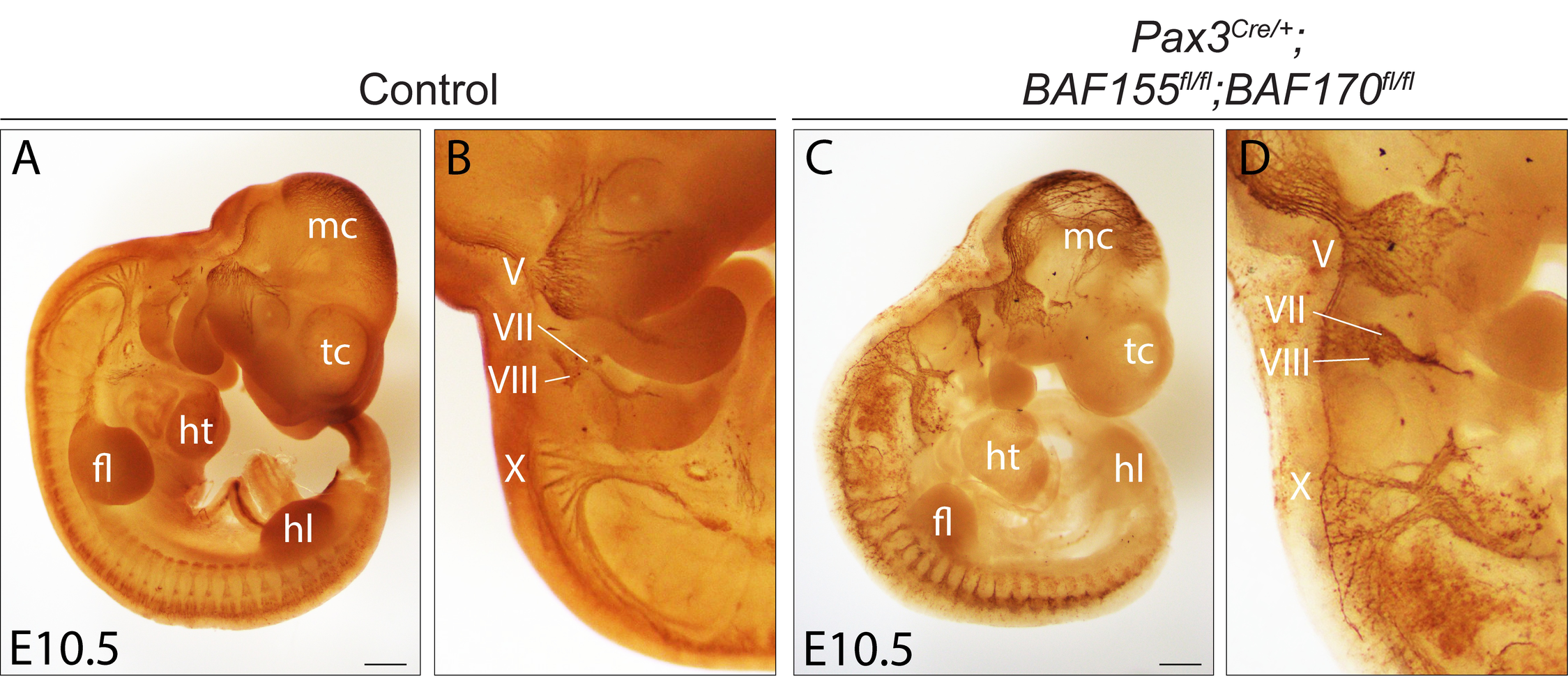

Supplement: S5 Fig — (A-D) Whole-mount neurofilament (2H3) immunostaining of E10.5 control and BAF155/170-deficient (Pax3Cre/+;BAF155fl/fl;BAF170fl/fl) embryos (n = 3 for each genotype). Impaired development of the cranial nerves (V: trigeminal, VII/VIII: facial/vestibulocochlear, X: vagal) is highlighted. Scale bars 200μM. (TIF) [file pgen.1009446.s005.tif]

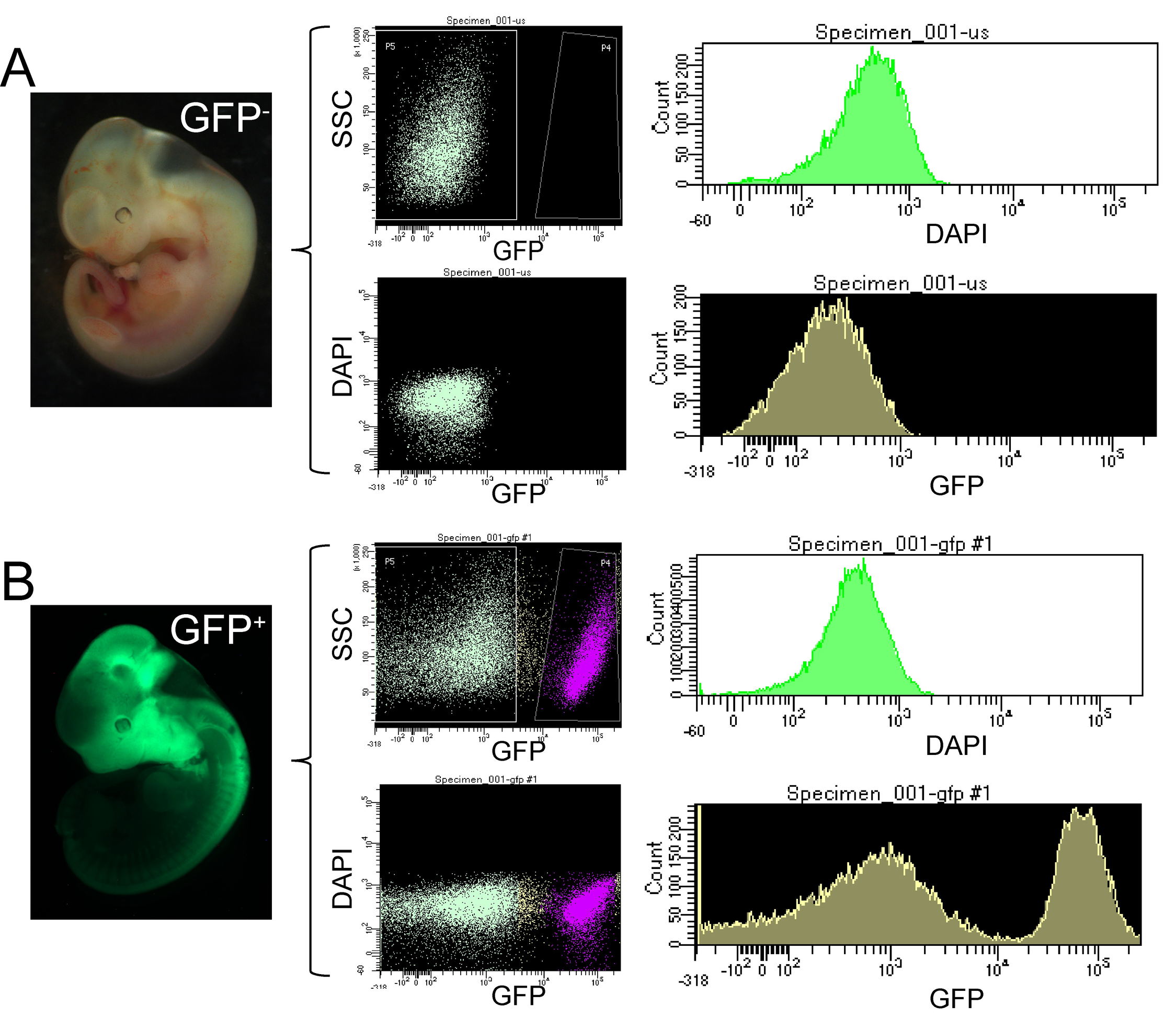

Supplement: S6 Fig — (A-B) Experimental design for NCCs isolation for RNAseq analysis using FACS. To setup the FACS, single cell suspensions were prepared from lineage traced GFP- control (BAF155fl/+:BAF170fl/+:Rosa26mTmG/+), GFP+ double heterozygous controls (Wnt1Cre/+:BAF155fl/+:BAF170fl/+:Rosa26mTmG/+) and BAF155/170-deficient (Wnt1Cre/+:BAF155fl/fl:BAF170fl/fl:Rosa26mTmG/+) embryos for FACS. NCCs that were positive for GFP were collected from double heterozygous controls (Wnt1Cre/+:BAF155fl/+:BAF170fl/+:Rosa26mTmG/+) and BAF155/170-deficient (Wnt1Cre/+:BAF155fl/fl:BAF170fl/fl:Rosa26mTmG/+) embryos for RNA isolation and subsequently for library preparation and RNAseq analysis. (TIF) [file pgen.1009446.s006.tif]

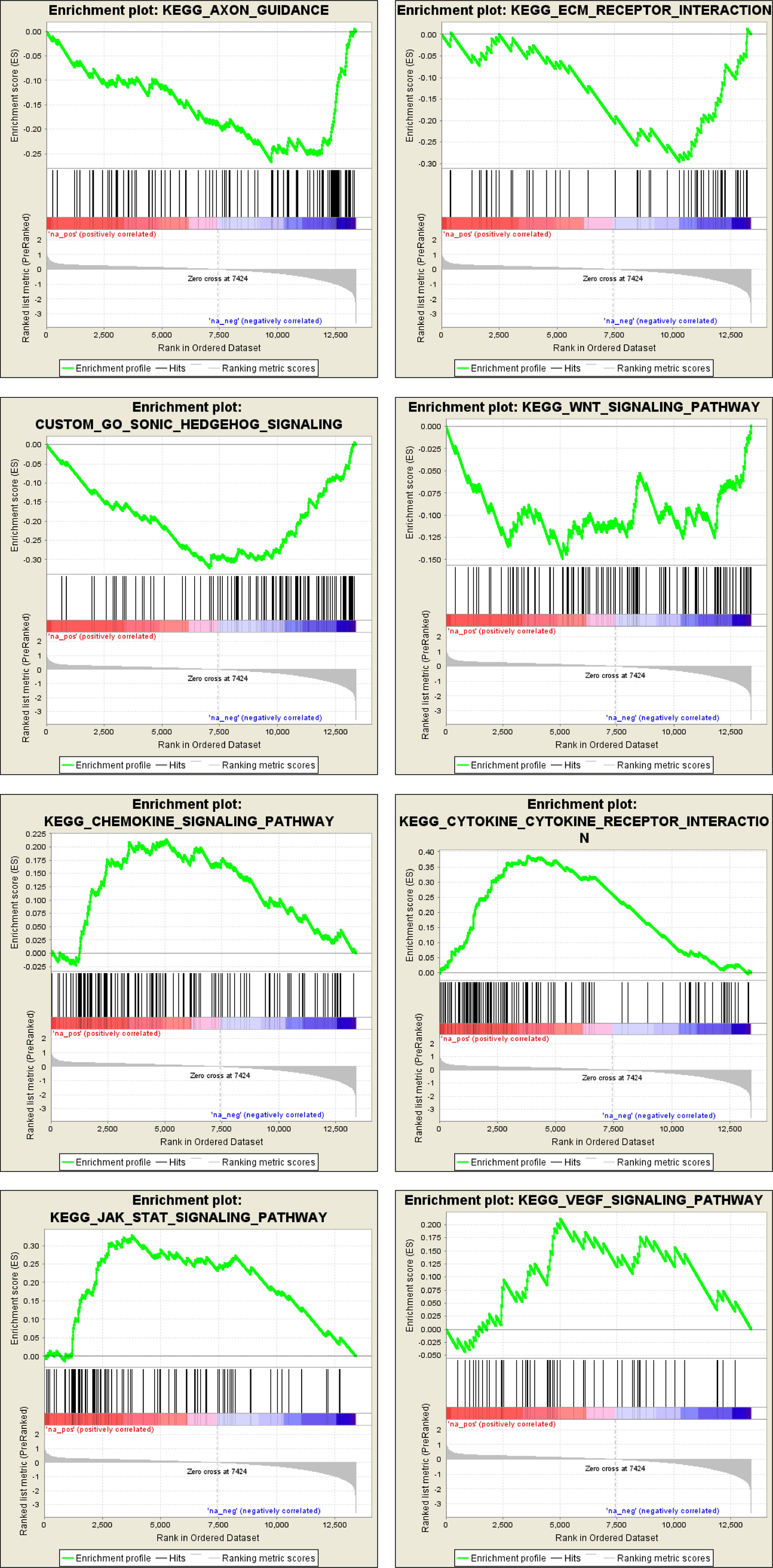

Supplement: S7 Fig — GSEA analysis showing changes in signaling pathways known to regulate different aspects of neural crest development. (TIF) [file pgen.1009446.s007.tif]

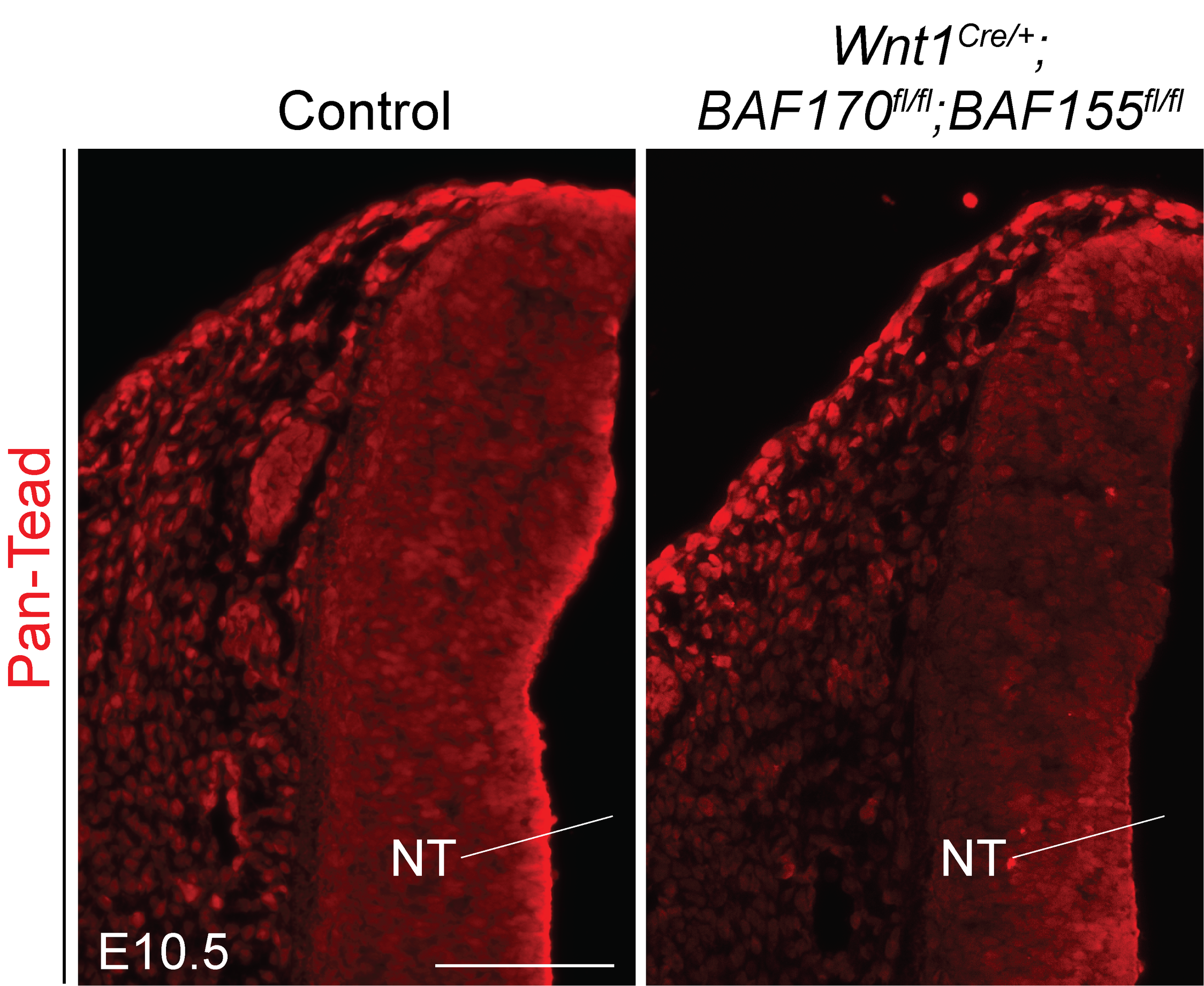

Supplement: S8 Fig — Immunostaining for Pan-Tead was performed on E10.5 transverse sections. Scale bars 100μM. NT, Neural tube. (TIF) [file pgen.1009446.s008.tif]

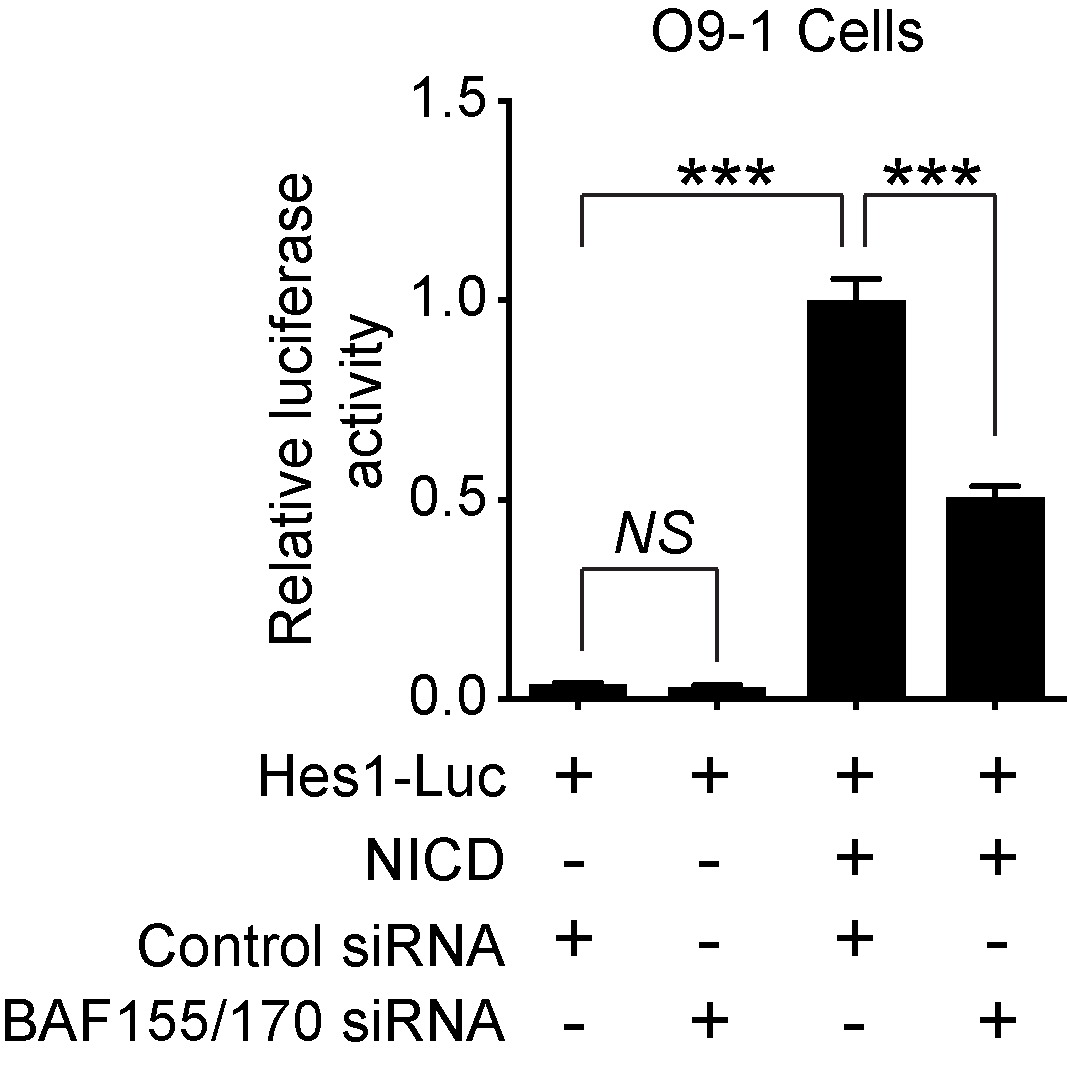

Supplement: S9 Fig — Results of normalized luciferase reporter assays in O9-1 cells with Hes1-luciferase reporters in the presence of control siRNA or BAF155/170 siRNA. Values are reported as means ± SD (*P < 0.05, **P < 0.01, ***P < 0.001; NS, not significant). (TIF) [file pgen.1009446.s009.tif]

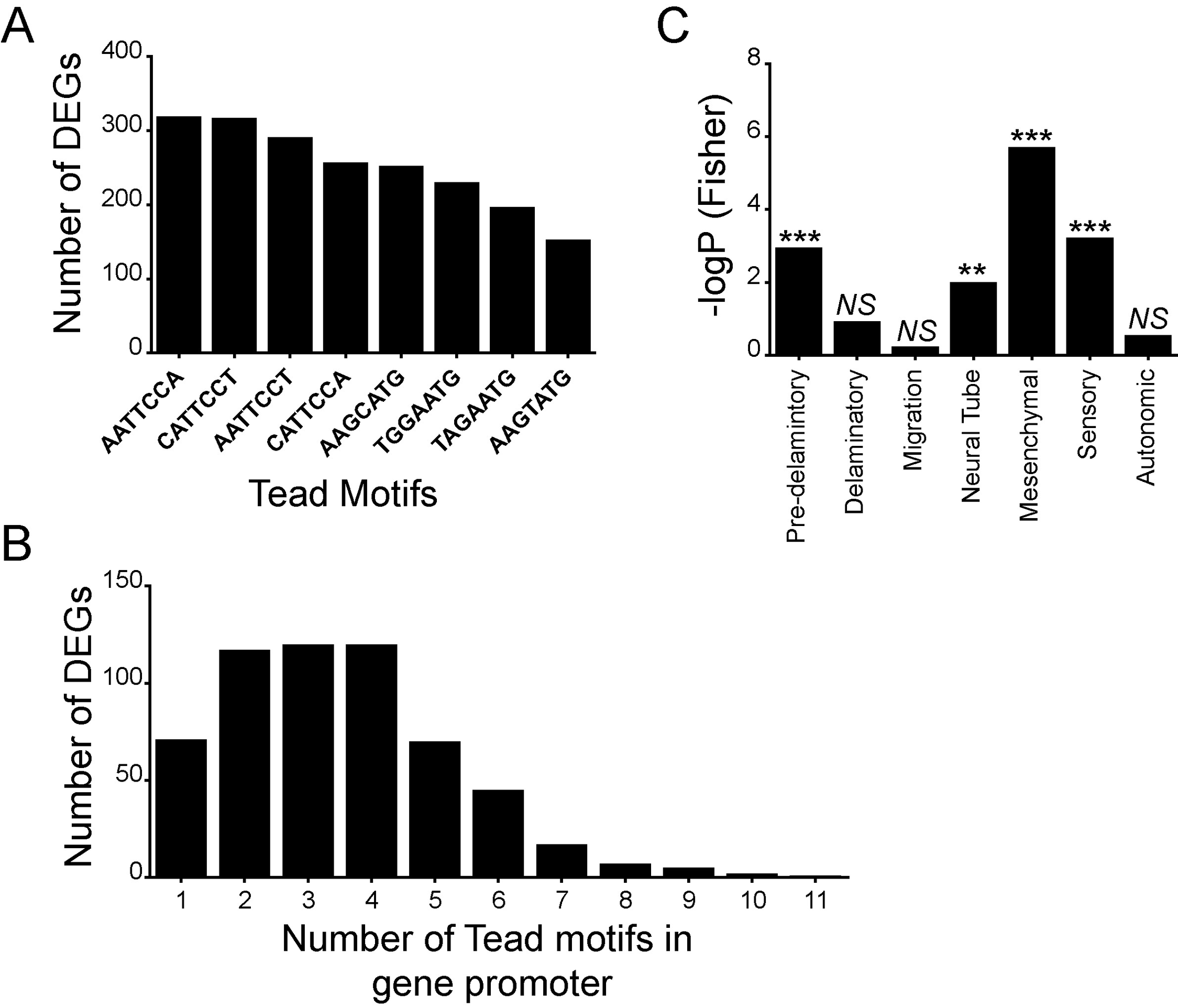

Supplement: S10 Fig — (A) Tead binding motifs in the 5kb promoter of differentially expressed genes (DEGs) identified in BAF155/170-deficient NCCs. (B) Number of Tead motifs in the gene promoter of DEGs. Over 100 DEGs have more than one and less than five Tead motifs. (C) Fisher’s test showing enrichment of differentially expressed neural crest genes with Tead motifs in scRNAseq clusters identified by Soldato et al., 2019. (TIF) [file pgen.1009446.s010.tif]
