# Supplementary material for: Critical role of the BAF chromatin remodeling complex during murine neural crest development
Source: PLoS Genet. 2021 Mar 22;17(3):e1009446. doi: 10.1371/journal.pgen.1009446 (PMC8016319; doi:10.1371/journal.pgen.1009446)
Supplement: S1 Table — (PDF) [file pgen.1009446.s011.pdf]

Supplemental table 1. List of differentially expressed genes between control and BAF155/170 double mutant NCCs.

ensembl	gene	logFC	AveExpr	t	P.Value	adj.P.Val	B	Control_1	Control_2	Control_3	dKO_1	dKO_2	dKO_3
ENSMUSG00000108741.1	Gm44553	-3.59227	-1.2438	-4.4442	0.000493	0.020558	-0.56355	0.776780271	0.829071718	0.1045059	-0.407326071	-3.461021292	-5.304833203
ENSMUSG00000102594.1	Gm38381	-3.29207	-0.27941	-6.55207	9.99E-06	0.004774	1.994255	1.347095996	1.948370647	0.88691447	-1.847898663	-1.876058791	-2.134908201
ENSMUSG00000028197.4	Col24a1	-2.7867	-0.90224	-5.19167	0.000116	0.010533	0.334098	0.255948108	0.829071718	0.41484602	-3.070291084	-1.345544075	-2.497478281
ENSMUSG00000002266.15	Zim1	-2.55856	0.053176	-4.8462	0.000224	0.014573	0.219449	1.404811494	1.585800567	0.88691447	-0.262936162	-0.798056279	-2.497478281
ENSMUSG00000026452.15	Syt2	-2.53487	-0.11807	-5.21738	0.00011	0.010497	0.611963	1.286975003	1.643516065	0.58993273	-1.485328583	-1.139093197	-1.604393485
ENSMUSG00000028626.5	Col9a2	-2.5209	-0.72711	-4.74575	0.000273	0.015809	-0.18963	0.487273654	0.614946913	0.50504383	-1.847898663	-1.139093197	-2.982905108
ENSMUSG00000055235.11	Wdr86	-2.51388	-0.5025	-4.76931	0.00026	0.015528	-0.1015	0.376242341	1.180544089	0.74605193	-1.195821966	-2.724055698	-1.397942607
ENSMUSG00000019906.14	Lin7a	-2.48425	-0.19264	-4.99116	0.00017	0.012548	0.299088	0.861669169	1.525679575	0.74605193	-0.567790743	-1.876058791	-1.845401584
ENSMUSG00000030554.15	Synm	-2.41608	0.20537	-4.57428	0.000381	0.018298	-0.08536	1.286975003	2.120837843	0.81820172	0.099633918	-1.876058791	-1.217370362
ENSMUSG00000026147.16	Col9a1	-2.41201	0.25423	-4.72345	0.000285	0.016299	0.138266	1.565276166	2.07961518	0.88691447	-0.954813866	-1.876058791	-0.175550186
ENSMUSG00000029754.13	Dlx6	-2.39082	-0.45719	-5.09089	0.00014	0.011551	0.304244	1.017788371	0.614946913	0.58993273	-1.485328583	-1.876058791	-1.604393485
ENSMUSG00000067276.5	Capn6	-2.30385	1.127891	-6.49429	1.10E-05	0.004774	3.016836	2.332298994	2.510249534	1.93638314	0.630148634	-0.091787482	-0.549945701
ENSMUSG00000021567.14	Nkd2	-2.29834	-0.09071	-4.53173	0.000414	0.018974	-0.25778	0.376242341	1.585800567	1.18857017	-1.195821966	-0.65366637	-1.845401584
ENSMUSG00000098973.1	Mir6236	-2.26927	12.41283	-4.06675	0.001046	0.03013	-0.88472	13.44637	13.92300716	13.2765145	10.92497554	13.16635584	9.739774368
ENSMUSG00000086503.3	Xist	-2.26411	6.980508	-5.18987	0.000116	0.010533	1.177974	7.831405143	8.336592991	8.17253174	5.001171279	7.416774073	5.124573539
ENSMUSG00000074818.12	Pdzd7	-2.22033	-0.83396	-4.43343	0.000503	0.020653	-0.62636	0.255948108	0.494652679	0.1045059	-1.485328583	-2.238628871	-2.134908201
ENSMUSG00000104315.1	Gm37906	-2.18914	-0.60648	-3.15633	0.006638	0.085817	-2.32663	0.124703574	0.829071718	0.41484602	-1.195821966	-0.091787482	-3.719870702
ENSMUSG00000047793.13	Sned1	-2.16537	0.811676	-6.40112	1.29E-05	0.005109	2.707325	1.999172692	2.037179913	1.6894684	-0.407326071	-0.188002798	-0.260439084
ENSMUSG00000033676.13	Gabrb3	-2.16005	-0.26787	-4.78875	0.000251	0.015223	0.018307	0.941839517	0.829071718	0.67010308	-1.485328583	-0.958520951	-1.604393485
ENSMUSG00000030074.9	Gxylt2	-2.14555	-0.43596	-3.48733	0.003385	0.057354	-1.82479	0.487273654	1.015484842	0.31863071	0.298942726	-2.238628871	-2.497478281
ENSMUSG00000043496.7	Tril	-2.14122	1.785544	-5.23594	0.000106	0.010484	1.422956	2.765809153	3.664577681	2.14715024	1.227389465	0.786906222	0.121431552
ENSMUSG00000105624.1	Gm43187	-2.1269	-0.21527	-4.46059	0.000477	0.020447	-0.39478	0.376242341	1.015484842	1.07535956	-0.954813866	-0.958520951	-1.845401584
ENSMUSG00000087943.1	Gm24245	-2.11013	5.879888	-3.95751	0.001304	0.033264	-1.239	6.76512068	7.293582261	6.73619227	4.402738138	6.717643559	3.364051781
ENSMUSG00000057716.6	Tmem178b	-2.07554	0.702099	-4.93418	0.000189	0.013297	0.649276	1.460306606	2.478885363	1.34329276	-0.011397395	-0.798056279	-0.260439084
ENSMUSG00000030757.13	Zkscan2	-2.06979	1.038779	-6.04461	2.42E-05	0.006757	2.395893	2.0361669	1.948370647	2.11886119	0.77101117	-0.29109629	-0.350636892
ENSMUSG00000030500.5	Slc17a6	-2.04028	0.754148	-4.63169	0.000341	0.017922	0.207435	1.999172692	2.237877264	1.18857017	-0.011397395	-1.139093197	0.249755649
ENSMUSG00000017692.8	Rhbdl3	-2.02177	0.999562	-5.31131	9.24E-05	0.01	1.329489	2.208119583	2.310940726	1.48301752	0.630148634	-0.188002798	-0.446852208
ENSMUSG00000031837.13	Necab2	-2.01018	-0.20499	-4.06516	0.00105	0.030161	-0.94312	0.776780271	1.015484842	0.74605193	-2.33332549	-0.522421837	-0.91251578
ENSMUSG00000105985.1	Gm42993	-2.00761	1.301332	-5.56373	5.78E-05	0.008819	1.81878	2.175329648	2.712557709	1.99980852	1.07266687	-0.402127603	0.249755649
ENSMUSG00000000031.15	H19	-2.00431	4.50662	-7.52259	2.03E-06	0.003403	5.27057	5.547769448	6.169535764	4.81158889	3.766811182	3.225479235	3.518534037
ENSMUSG00000040310.12	Alx4	-2.00036	1.611952	-5.28042	9.79E-05	0.010266	1.462897	2.0361669	3.47093661	2.426434	0.554199781	0.380280962	0.803691254
ENSMUSG00000099746.1	Ppnr	-1.99782	-0.55854	-3.82686	0.001698	0.038362	-1.37055	0.590367147	0.494652679	0.21553721	-0.567790743	-2.238628871	-1.845401584
ENSMUSG00000102657.1	Gm37899	-1.99768	1.047763	-4.68209	0.000309	0.017041	0.372026	2.141777088	2.414034219	1.5267389	1.17763643	-0.798056279	-0.175550186
ENSMUSG00000042961.13	Egflam	-1.97579	0.509333	-3.8764	0.001536	0.036283	-1.03935	1.565276166	2.380481659	0.58993273	0.099633918	-0.522421837	-1.056905689
ENSMUSG00000052854.15	Nrk	-1.97469	0.449124	-4.7918	0.000249	0.015202	0.351382	1.224239248	1.948370647	1.13307505	-0.262936162	-0.29109629	-1.056905689
ENSMUSG00000059049.14	Frem1	-1.9641	2.371988	-7.96146	1.03E-06	0.002902	5.614134	3.63325296	3.502472184	2.9604956	1.453270872	1.329055639	1.35337828
ENSMUSG00000102428.1	Pcdhga12	-1.95499	0.518525	-4.12854	0.000924	0.028569	-0.63502	2.072236155	1.643516065	0.74605193	0.389140535	-0.958520951	-0.781271247
ENSMUSG00000049038.2	Mterf2	-1.94767	0.035259	-4.42268	0.000514	0.020905	-0.34048	1.347095996	1.10037374	0.58993273	-0.567790743	-1.345544075	-0.91251578
ENSMUSG00000026674.9	Ddr2	-1.947	2.308156	-7.73053	1.47E-06	0.002902	5.287844	3.425873109	3.533333147	2.89527794	1.494493535	1.429749638	1.070206228
ENSMUSG00000035305.5	Ror1	-1.94259	0.101093	-3.86655	0.001567	0.0366	-1.14181	0.776780271	1.993458536	0.50504383	-0.748362989	-0.522421837	-1.397942607
ENSMUSG00000029778.12	Adcyap1r1	-1.93204	0.999274	-4.87788	0.000211	0.014141	0.670819	1.663123489	2.65759125	1.56917417	0.554199781	-0.001589673	-0.446852208
ENSMUSG00000062961.6	Ccdc177	-1.93165	0.234899	-4.69446	0.000301	0.016984	0.128917	1.017788371	1.256492942	1.29353973	-0.262936162	-1.345544075	-0.549945701
ENSMUSG00000106022.1	Gm42929	-1.92554	0.105411	-3.70693	0.002166	0.043866	-1.38227	1.460306606	1.397355478	0.31863071	-0.407326071	-0.29109629	-1.845401584
ENSMUSG00000030020.13	Prickle2	-1.91835	0.787717	-4.86821	0.000215	0.014141	0.591865	1.513745865	2.199909414	1.48301752	0.630148634	-0.65366637	-0.446852208
ENSMUSG00000102516.1	Gm38340	-1.91518	-0.33107	-4.03481	0.001116	0.030999	-1.01712	0.487273654	0.614946913	0.67010308	-0.567790743	-1.345544075	-1.845401584
ENSMUSG00000021301.8	Hecw1	-1.90796	0.530379	-4.93618	0.000189	0.013297	0.596215	1.754753965	1.397355478	1.34329276	-0.407326071	-0.958520951	0.052718802
ENSMUSG00000025867.8	Cplx2	-1.9077	1.596546	-6.50505	1.08E-05	0.004774	3.314931	2.851327224	2.346130154	2.37951295	1.126106129	0.508605059	0.367592139
ENSMUSG00000066113.15	Adamtsl1	-1.90251	-0.21786	-3.23074	0.005707	0.078674	-2.16254	0.376242341	1.180544089	0.58993273	-1.195821966	0.239418426	-2.497478281
ENSMUSG00000004110.14	Cacna1e	-1.89826	0.900766	-3.73033	0.002065	0.042506	-1.22451	2.446631669	2.478885363	0.74605193	-0.131691629	0.311568212	-0.446852208
ENSMUSG00000078190.6	Dnm3os	-1.8916	2.225104	-7.82317	1.27E-06	0.002902	5.380798	3.008510557	3.150999813	3.2871982	1.754137352	0.884753545	1.265022405
ENSMUSG00000010066.15	Cacna2d2	-1.89097	1.140336	-5.49375	6.58E-05	0.009194	1.658808	1.922210711	2.629302206	1.76443046	0.099633918	0.445869304	-0.019430984
ENSMUSG00000044576.6	Garem2	-1.8828	-0.18172	-3.33517	0.004614	0.06951	-2.00371	0.686582462	1.585800567	0.21553721	-2.33332549	-0.188002798	-1.056905689
ENSMUSG00000108394.1	Gm45477	-1.87323	-0.17155	-4.05137	0.001079	0.030547	-0.9416	0.686582462	1.180544089	0.41484602	-0.748362989	-0.958520951	-1.604393485
ENSMUSG00000103722.1	2700078F05Ri	-1.87074	0.281979	-4.33946	0.000606	0.022923	-0.37235	0.686582462	1.643516065	1.29353973	-0.131691629	-1.139093197	-0.660977013
ENSMUSG00000041729.15	Coro2b	-1.85803	0.569644	-3.32954	0.004668	0.070051	-1.91839	0.776780271	2.65759125	1.01523856	0.554199781	-0.188002798	-1.397942607
ENSMUSG00000047757.16	Fancb	-1.85349	-0.60421	-3.02589	0.008649	0.098298	-2.512	0.124703574	0.494652679	0.21553721	-0.131691629	-1.345544075	-2.982905108
ENSMUSG00000027820.12	Mme	-1.85332	0.908681	-5.45477	7.07E-05	0.009244	1.510379	1.961204842	2.07961518	1.5267389	-0.011397395	-0.29109629	0.187019893
ENSMUSG00000023236.7	Scg5	-1.84908	-0.11986	-3.88658	0.001505	0.035994	-1.16434	0.686582462	1.015484842	0.67010308	-0.954813866	-0.29109629	-1.845401584
ENSMUSG00000025478.15	Dpysl4	-1.84622	3.288136	-5.91275	3.06E-05	0.007194	2.673527	4.244138805	4.904781378	3.53211207	2.76259893	1.896530713	2.388653755
ENSMUSG00000004328.15	Hif3a	-1.84293	0.013741	-3.46039	0.003576	0.058949	-1.77639	1.017788371	1.256492942	0.50504383	0.202727411	-2.238628871	-0.660977013
ENSMUSG00000021763.16	BC067074	-1.8358	0.461326	-4.38861	0.00055	0.021644	-0.24479	1.513745865	1.901828061	0.81820172	-0.407326071	-0.798056279	-0.260439084
ENSMUSG00000057777.4	Mab21l2	-1.82545	1.282284	-5.49356	6.58E-05	0.009194	1.715518	2.361742772	2.414034219	1.80049971	0.899335267	-0.091787482	0.309876641
ENSMUSG00000058897.17	Col25a1	-1.81378	0.852793	-4.24417	0.000733	0.025228	-0.37549	1.404811494	2.629302206	1.29353973	0.099633918	0.239418426	-0.549945701
ENSMUSG00000016346.17	Kcnq2	-1.80716	1.541892	-5.6346	5.07E-05	0.008615	2.013795	2.240180792	3.04790632	2.09000633	0.836599512	0.508605059	0.528056811
ENSMUSG00000045994.16	B3gat1	-1.80536	-0.57383	-3.5032	0.003277	0.056198	-1.82713	0.124703574	0.725978225	0.21553721	-2.33332549	-0.958520951	-1.217370362
ENSMUSG00000103780.1	Gm37524	-1.80202	0.180489	-4.19154	0.000815	0.026726	-0.62141	1.158650906	1.10037374	0.88691447	-0.011397395	-0.65366637	-1.397942607
ENSMUSG00000106386.1	Gm42991	-1.80036	0.559338	-3.96421	0.001286	0.03302	-0.88294	1.709666075	1.699011178	0.88691447	0.77101117	-0.798056279	-0.91251578
ENSMUSG00000029913.14	Prdm5	-1.79306	0.290865	-3.65455	0.002409	0.046328	-1.42616	1.347095996	1.803980737	0.41484602	-0.131691629	-0.29109629	-1.397942607
ENSMUSG00000015619.10	Gata3	-1.79237	0.245616	-4.18684	0.000822	0.026847	-0.61082	0.686582462	1.585800567	1.13307505	-0.131691629	-1.139093197	-0.660977013
ENSMUSG00000085884.1	Gm15342	-1.78879	-0.23764	-3.64921	0.002435	0.04655	-1.54241	0.686582462	0.925287033	0.31863071	-0.262936162	-1.876058791	-1.217370362
ENSMUSG00000097391.8	Mirg	-1.78479	1.247243	-4.77401	0.000258	0.015525	0.573458	2.208119583	2.446824154	1.6894684	1.017171757	0.568726051	-0.446852208
ENSMUSG00000053552.14	Ebf4	-1.78476	-0.03384	-3.06167	0.008044	0.094798	-2.39415	0.776780271	1.180544089	0.50504383	-0.407326071	0.239418426	-2.497478281
ENSMUSG00000023868.15	Pde10a	-1.77817	0.429921	-3.84828	0.001626	0.037299	-1.09584	1.286975003	1.803980737	1.01523856	-1.485328583	0.508605059	-0.549945701
ENSMUSG00000018554.13	Ybx2	-1.76893	0.280776	-4.27599	0.000688	0.024172	-0.46546	1.224239248	1.462943819	0.81820172	-0.131691629	-1.139093197	-0.549945701
ENSMUSG00000025789.9	St8sia2	-1.7673	1.497958	-5.85573	3.39E-05	0.007586	2.336545	2.271544963	2.865908486	2.09000633	0.474029432	0.568726051	0.71753461
ENSMUSG00000022231.9	Sema5a	-1.76415	2.285511	-5.1912	0.000116	0.010533	1.415084	2.951554672	3.883980059	2.66032206	1.787689911	1.668261725	0.761255988
ENSMUSG00000051243.13	Islr2	-1.7639	1.318431	-4.32037	0.00063	0.023329	-0.17552	2.361742772	2.982317978	1.39138705	0.298942726	0.508605059	0.367592139
ENSMUSG00000036913.14	Trim67	-1.7633	0.888028	-4.49263	0.000448	0.019825	0.037306	1.565276166	2.310940726	1.39138705	0.70229842	-0.091787482	-0.549945701
ENSMUSG00000047787.7	Flrt1	-1.76008	0.949807	-4.4877	0.000452	0.019825	0.043863	1.961204842	2.120837843	1.34329276	0.77101117	0.163469573	-0.660977013
ENSMUSG00000016763.14	Scube1	-1.75854	2.277245	-5.55813	5.84E-05	0.008819	2.038538	3.199991702	3.771990623	2.55868179	1.719785847	0.97638402	1.436633784
ENSMUSG00000042515.13	Mum1l1	-1.75615	0.758174	-3.21364	0.005909	0.080014	-2.10162	2.240180792	1.015484842	1.39138705	1.126106129	0.380280962	-1.604393485
ENSMUSG00000036109.17	Mbnl3	-1.74698	0.254883	-4.01153	0.001169	0.031531	-0.87246	1.158650906	0.829071718	1.24200942	0.099633918	-0.402127603	-1.397942607
ENSMUSG00000000120.6	Ngfr	-1.74544	1.417664	-4.43024	0.000506	0.02072	0.024396	2.390597634	3.069122806	1.5267389	0.630148634	0.311568212	0.577809846
ENSMUSG00000059022.14	Kcp	-1.74477	1.157045	-4.31118	0.000642	0.023329	-0.20902	2.332298994	2.120837843	1.5267389	1.410835606	-0.188002798	-0.260439084
ENSMUSG00000028348.7	Cavin4	-1.74218	0.476476	-4.73063	0.000281	0.016167	0.282538	1.089938156	1.462943819	1.48301752	-0.262936162	-0.65366637	-0.260439084
ENSMUSG00000041216.15	Clvs1	-1.74058	-0.26673	-3.07451	0.007837	0.093484	-2.40175	0.376242341	1.462943819	0.1045059	-0.954813866	-2.238628871	-0.350636892
ENSMUSG00000042109.7	Csdc2	-1.73991	-0.47688	-3.28556	0.005105	0.073913	-2.12111	0.124703574	1.015484842	0.1045059	-0.954813866	-2.238628871	-0.91251578
ENSMUSG00000002006.12	Pdzd4	-1.73952	0.646294	-4.74435	0.000273	0.015809	0.365055	1.615029201	1.256492942	1.56917417	0.389140535	-0.402127603	-0.549945701
ENSMUSG00000029093.14	Sorcs2	-1.73947	0.546888	-4.07492	0.001029	0.030012	-0.70823	1.017788371	2.160915282	1.07535956	0.099633918	-0.29109629	-0.781271247
ENSMUSG00000032327.14	Stra6	-1.73848	0.284272	-3.61312	0.002621	0.048717	-1.49361	0.861669169	2.160915282	0.58993273	-0.954813866	-0.402127603	-0.549945701
ENSMUSG00000032186.14	Tmod2	-1.73461	1.989923	-4.44631	0.000491	0.020558	0.090494	3.06330303	3.593142542	1.96844435	1.534570974	0.97638402	0.803691254
ENSMUSG00000058153.14	Sez6l	-1.72828	-0.06048	-3.38519	0.004168	0.065001	-1.9036	0.686582462	1.752450437	0.1045059	-1.195821966	-0.65366637	-1.056905689
ENSMUSG00000040387.16	Klhl32	-1.72689	-0.2517	-3.44729	0.003673	0.0601	-1.84107	0.376242341	0.725978225	0.58993273	-0.011397395	-1.345544075	-1.845401584
ENSMUSG00000033006.9	Sox10	-1.72684	1.166771	-4.06716	0.001045	0.03013	-0.62361	2.107425583	2.841455443	1.24200942	0.630148634	-0.188002798	0.367592139
ENSMUSG00000104063.1	Pcdhgb7	-1.72663	0.004036	-3.55567	0.002945	0.052912	-1.62949	1.513745865	0.725978225	0.31863071	-0.131691629	-1.345544075	-1.056905689
ENSMUSG00000031486.14	Adgra2	-1.72251	2.246117	-5.05421	0.00015	0.011837	1.175197	2.809201749	3.883980059	2.62052105	1.914602024	1.363407143	0.884991356
ENSMUSG00000053024.14	Cntn2	-1.7212	4.432282	-7.2159	3.31E-06	0.004077	4.78785	5.109596682	5.938316982	4.81158889	3.940936172	3.457841945	3.335411733
ENSMUSG00000085133.2	B930095G15R	-1.71085	1.122149	-4.97742	0.000174	0.012702	0.872514	1.709666075	2.274871471	1.87004065	0.899335267	0.239418426	-0.260439084
ENSMUSG00000052187.3	Hbb-y	-1.70799	2.526903	-4.17551	0.000841	0.027264	-0.42902	3.216201929	4.482843377	2.57959078	1.883905227	1.143840766	1.855038134
ENSMUSG00000057751.14	Megf6	-1.70478	2.581552	-4.61713	0.00035	0.018054	0.387819	3.533286052	4.185409844	2.66032206	2.215111135	1.020105398	1.875075887
ENSMUSG00000023036.14	Pcdhgc4	-1.70371	-0.01365	-3.55949	0.002923	0.052789	-1.62779	1.089938156	1.256492942	0.21553721	-0.954813866	-0.29109629	-1.397942607
ENSMUSG00000031661.11	Nkd1	-1.69432	1.72434	-5.58985	5.51E-05	0.008819	1.999129	2.627203915	2.95977541	2.11886119	1.275483753	0.836659257	0.528056811
ENSMUSG00000044707.7	Ccnjl	-1.69108	1.039648	-4.44896	0.000488	0.02053	-0.00163	1.961204842	2.346130154	1.43792963	0.389140535	-0.522421837	0.625904135
ENSMUSG00000021136.13	Smoc1	-1.68236	0.992993	-4.58012	0.000377	0.018298	0.20632	2.0361669	1.643516065	1.6894684	0.899335267	0.239418426	-0.549945701
ENSMUSG00000025352.6	Gdf11	-1.68133	1.73998	-5.03834	0.000155	0.011953	1.101051	2.141777088	3.302007601	2.30613976	1.227389465	0.884753545	0.577809846
ENSMUSG00000090063.5	Dlx6os1	-1.68121	0.555785	-4.28621	0.000674	0.023999	-0.37229	1.404811494	1.752450437	1.13307505	-0.407326071	-0.798056279	0.249755649
ENSMUSG00000000861.15	Bcl11a	-1.68087	3.022581	-7.21023	3.34E-06	0.004077	4.753867	3.680753383	4.214264707	3.70382369	2.563914936	1.61222769	2.360502714
ENSMUSG00000097536.2	2610037D02R	-1.67984	0.267864	-3.9321	0.001373	0.034361	-0.99287	1.709666075	0.925287033	0.67010308	-0.262936162	-0.65366637	-0.781271247
ENSMUSG00000042631.7	Xkr7	-1.67825	0.275633	-3.30312	0.004925	0.07261	-1.98597	0.686582462	2.274871471	0.50504383	-0.407326071	-0.958520951	-0.446852208
ENSMUSG00000048583.16	Igf2	-1.67751	4.432567	-6.07899	2.28E-05	0.006757	2.879428	5.181534797	6.008637677	4.60785464	4.034744387	3.384468759	3.378161381
ENSMUSG00000020431.5	Adcy1	-1.67534	0.984724	-4.12407	0.000933	0.02863	-0.54919	1.615029201	2.712557709	1.24200942	0.202727411	0.083299224	0.052718802
ENSMUSG00000025927.13	Tfap2b	-1.66695	2.803677	-6.35942	1.39E-05	0.005186	3.407999	3.493937384	4.053239764	3.33716666	2.620870821	1.461810848	1.855038134
ENSMUSG00000102602.1	A930004J17Ri	-1.66364	1.511779	-5.64548	4.97E-05	0.008553	2.030091	2.271544963	2.199909414	2.53746531	0.899335267	0.163469573	0.998947545
ENSMUSG00000026494.12	Kif26b	-1.65427	2.733642	-4.36695	0.000574	0.022014	-0.08995	3.369010772	4.596799566	2.80937787	1.944659258	2.192420947	1.489582663
ENSMUSG00000056174.8	Col8a2	-1.65286	0.228072	-3.0393	0.008417	0.097285	-2.40898	0.941839517	2.237877264	0.1045059	-0.567790743	-0.29109629	-1.056905689

ENSMUSG00000104184.1	Gm37818	-1.65258	-0.39707	-2.76815	0.014534	0.132596	-2.86333	0.376242341	0.925287033	-0.14703287	0.099633918	-1.139093197	-2.497478281
ENSMUSG00000039239.14	Tgfb2	-1.6519	0.737813	-4.31292	0.000639	0.023329	-0.28316	1.017788371	2.07961518	1.56917417	0.298942726	-0.188002798	-0.350636892
ENSMUSG00000039037.5	St6galnac5	-1.65181	-0.58527	-3.06803	0.007941	0.094053	-2.44939	0.255948108	0.494652679	0.1045059	-2.33332549	-1.586552174	-0.446852208
ENSMUSG00000000567.5	Sox9	-1.65113	1.889978	-5.07985	0.000143	0.011551	1.19162	2.721070838	3.247215128	2.17489523	1.410835606	1.257796956	0.528056811
ENSMUSG00000070880.10	Gad1	-1.64834	0.375229	-3.97418	0.001261	0.032787	-0.90098	1.224239248	1.015484842	1.24200942	-0.011397395	-0.001589673	-1.217370362
ENSMUSG00000061080.11	Lsamp	-1.64818	0.347967	-2.72999	0.015687	0.138972	-2.89591	0.590367147	1.948370647	0.81820172	0.70229842	0.163469573	-2.134908201
ENSMUSG00000021097.14	Clmn	-1.64436	1.188995	-4.33351	0.000614	0.022937	-0.16551	1.922210711	2.274871471	1.76443046	0.70229842	1.020105398	-0.549945701
ENSMUSG00000015222.17	Map2	-1.63585	4.72261	-7.7095	1.51E-06	0.002902	5.548626	5.277350584	6.086134373	5.24036907	4.301848457	3.57240171	3.857558126
ENSMUSG00000034684.12	Sema3f	-1.63493	1.632608	-4.24014	0.000739	0.025303	-0.28662	2.30224176	3.265711472	1.80049971	1.453270872	0.445869304	0.528056811
ENSMUSG00000020926.16	Adam11	-1.62902	1.3337	-4.57457	0.000381	0.018298	0.260085	2.240180792	2.765506589	1.5267389	0.630148634	0.311568212	0.528056811
ENSMUSG00000025555.14	Farp1	-1.62799	2.994904	-6.49381	1.10E-05	0.004774	3.638647	3.726739589	4.306481078	3.38546221	2.602134258	2.113887544	1.83471815
ENSMUSG00000003665.6	Has1	-1.6265	-0.31593	-2.80439	0.013516	0.126608	-2.80463	0.376242341	0.614946913	0.50504383	-0.407326071	-2.724055698	-0.260439084
ENSMUSG00000107353.1	4930430O22R	-1.62531	-0.59155	-3.13451	0.006939	0.087573	-2.35491	0.255948108	0.219018236	0.1045059	-0.407326071	-1.876058791	-1.845401584
ENSMUSG00000058297.16	Spock2	-1.62207	1.673571	-2.97215	0.009642	0.104125	-2.57105	2.892257488	3.63642664	1.07535956	1.07266687	0.836659257	0.528056811
ENSMUSG00000024593.15	Megf10	-1.62093	1.83035	-5.07534	0.000144	0.011551	1.17794	2.765809153	2.913605228	2.17489523	1.684596418	1.020105398	0.423087252
ENSMUSG00000030000.10	Add2	-1.62	2.35585	-5.76116	4.03E-05	0.008063	2.387444	2.932058497	3.758993282	2.82697193	1.944659258	1.182834898	1.489582663
ENSMUSG00000031285.14	Dcx	-1.61962	4.2606	-6.48765	1.12E-05	0.004774	3.598675	4.763053288	5.704106045	4.79380506	3.529621758	2.925559761	3.847451639
ENSMUSG00000050822.11	Slc29a4	-1.61479	0.314266	-3.50268	0.003281	0.056198	-1.6619	1.404811494	1.643516065	0.41484602	-0.262936162	-1.139093197	-0.175550186
ENSMUSG00000027684.16	Mecom	-1.61369	1.88415	-4.66306	0.00032	0.017543	0.472881	3.008510557	2.446824154	2.4493344	2.190236466	0.681936662	0.528056811
ENSMUSG00000107023.2	Gm42715	-1.61363	-0.4647	-3.10256	0.007404	0.090735	-2.3839	0.124703574	0.725978225	0.1045059	-0.262936162	-1.876058791	-1.604393485
ENSMUSG00000018698.15	Lhx1	-1.61108	1.845227	-5.3232	9.04E-05	0.009985	1.591942	2.787668587	3.150999813	2.11886119	0.899335267	0.97638402	1.138110293
ENSMUSG00000033763.14	Mtss1l	-1.60938	1.893357	-4.06299	0.001054	0.030224	-0.60195	2.500570476	3.533333147	2.03050532	1.852541056	1.020105398	0.423087252
ENSMUSG00000079330.8	Lemd1	-1.60929	0.741357	-4.80145	0.000245	0.015126	0.486106	1.709666075	1.462943819	1.43792963	0.099633918	-0.001589673	-0.260439084
ENSMUSG00000073867.2	AA474408	-1.60796	-0.59438	-3.31623	0.004796	0.071188	-2.0959	0.255948108	0.219018236	0.1045059	-0.954813866	-1.345544075	-1.845401584
ENSMUSG00000021464.14	Ror2	-1.60702	1.277451	-4.91779	0.000195	0.013581	0.817347	2.072236155	2.540946331	1.65047427	0.77101117	0.380280962	0.249755649
ENSMUSG00000104273.1	A530064N14R	-1.60493	0.283572	-4.01745	0.001155	0.03138	-0.85505	0.776780271	1.328642728	1.13307505	-0.131691629	-0.958520951	-0.446852208
ENSMUSG00000025738.7	Fbxl16	-1.59748	0.683952	-4.57545	0.00038	0.018298	0.119142	1.513745865	1.585800567	1.34329276	-0.131691629	0.239418426	-0.446852208
ENSMUSG00000020105.8	Lrig3	-1.59671	2.252761	-4.5631	0.00039	0.018542	0.300385	3.150238667	3.47093661	2.4493344	2.421562012	1.220802748	0.803691254
ENSMUSG00000085925.2	Rtl1	-1.59009	0.971681	-3.14847	0.006745	0.086205	-2.2121	1.961204842	2.510249534	0.81820172	0.77101117	0.681936662	-0.91251578
ENSMUSG00000039154.6	Shd	-1.58845	0.241898	-4.02103	0.001147	0.03138	-0.8585	0.861669169	0.925287033	1.24200942	-0.262936162	-0.402127603	-0.91251578
ENSMUSG00000103427.1	Gm37534	-1.58605	1.405212	-4.45781	0.00048	0.020447	0.075483	2.271544963	2.07961518	2.09000633	1.719785847	0.083299224	0.187019893
ENSMUSG00000021611.8	Tert	-1.58032	0.776787	-4.18413	0.000827	0.026928	-0.48129	1.709666075	1.585800567	1.34329276	0.77101117	-0.65366637	-0.095379837
ENSMUSG00000046580.10	Gm7862	-1.57986	0.25289	-3.65642	0.0024	0.046328	-1.42513	1.158650906	0.829071718	1.07535956	-0.567790743	0.239418426	-1.217370362
ENSMUSG00000021700.9	Rab3c	-1.57674	1.452747	-5.07496	0.000145	0.011551	1.111618	2.446631669	2.571003565	1.76443046	0.77101117	0.445869304	0.71753461
ENSMUSG00000037568.12	Vash2	-1.57494	1.788217	-6.03685	2.45E-05	0.006757	2.715758	2.473853138	2.913605228	2.35546747	1.17763643	0.884753545	0.923985488
ENSMUSG00000089827.1	1700023H06R	-1.57274	0.097371	-3.38503	0.004169	0.065001	-1.8775	0.776780271	1.525679575	0.50504383	-1.485328583	-0.188002798	-0.549945701
ENSMUSG00000027692.16	Tnik	-1.57218	2.463261	-4.65464	0.000326	0.01769	0.460873	3.081114306	3.84760767	2.79156659	2.002957898	2.21140405	0.844913917
ENSMUSG00000034701.9	Neurod1	-1.57152	0.643368	-2.91104	0.010907	0.111729	-2.60632	1.347095996	2.571003565	0.50504383	0.389140535	-1.139093197	0.187019893
ENSMUSG00000038677.13	Scube3	-1.57095	2.596921	-5.22556	0.000109	0.010484	1.484266	3.669024091	3.835276868	2.67981823	2.058991933	1.848833971	1.489582663
ENSMUSG00000042436.12	Mfap4	-1.56609	1.466078	-4.5527	0.000398	0.018566	0.243204	1.961204842	3.004513725	1.83568914	0.836599512	0.681936662	0.476526511
ENSMUSG00000032492.14	Pth1r	-1.56209	0.82193	-4.46133	0.000476	0.020447	-0.02396	1.513745865	1.901828061	1.39138705	0.474029432	-0.402127603	0.052718802
ENSMUSG00000005397.7	Nid1	-1.56109	2.453977	-4.98948	0.00017	0.012548	1.065628	3.045269107	4.053239764	2.67981823	1.787689911	1.668261725	1.489582663
ENSMUSG00000103937.1	Gm37186	-1.56075	-0.31626	-3.38552	0.004165	0.065001	-1.9446	0.124703574	0.829071718	0.50504383	-1.485328583	-0.958520951	-0.91251578
ENSMUSG00000034958.11	Atcay	-1.56074	0.473129	-3.8226	0.001713	0.038564	-1.12221	1.709666075	1.180544089	0.81820172	0.202727411	-0.29109629	-0.781271247
ENSMUSG00000020297.10	Nsg2	-1.56051	1.696375	-4.37833	0.000561	0.021658	-0.03915	1.798475342	3.319818877	2.35546747	1.07266687	0.786906222	0.844913917
ENSMUSG00000048015.3	Neurod4	-1.55911	0.74112	-3.46357	0.003553	0.058821	-1.68201	1.089938156	2.346130154	1.13307505	0.77101117	-0.798056279	-0.095379837
ENSMUSG00000097042.1	Gm17491	-1.55883	-0.03698	-3.48576	0.003396	0.057392	-1.74015	0.776780271	0.363408146	0.95250281	-0.262936162	-0.65366637	-1.397942607
ENSMUSG00000027932.14	Slc27a3	-1.55835	0.61141	-4.1695	0.000851	0.02733	-0.53883	1.286975003	1.328642728	1.5267389	0.202727411	-0.798056279	0.121431552
ENSMUSG00000021057.15	Akap5	-1.5566	-0.04988	-3.0601	0.00807	0.094989	-2.39621	1.158650906	0.725978225	0.21553721	-0.262936162	-0.29109629	-1.845401584
ENSMUSG00000098090.7	2700099C18R	-1.55547	0.107515	-3.53088	0.003098	0.054188	-1.64589	0.861669169	0.614946913	1.07535956	0.099633918	-1.345544075	-0.660977013
ENSMUSG00000028073.15	Pear1	-1.55483	-0.38033	-3.03676	0.00846	0.097312	-2.46742	0.376242341	0.363408146	0.31863071	-0.407326071	-0.798056279	-2.134908201
ENSMUSG00000065820.1	Gm26316	-1.55463	-0.0702	-2.94178	0.010251	0.107825	-2.58006	0.861669169	1.256492942	0.1045059	-1.485328583	0.239418426	-1.397942607
ENSMUSG00000104344.1	Gm38077	-1.55214	-0.044	-2.83853	0.012621	0.122107	-2.7352	1.017788371	0.829071718	0.21553721	0.099633918	-0.29109629	-2.134908201
ENSMUSG00000022055.7	Nefl	-1.54515	1.742782	-4.27282	0.000692	0.024199	-0.22409	2.418886679	3.209495462	1.90359321	1.611532956	0.836659257	0.476526511
ENSMUSG00000067786.16	Nnat	-1.54451	3.927313	-5.9677	2.77E-05	0.006901	2.724947	4.706800741	5.361259679	4.06427606	3.233489664	3.298312115	2.899737941
ENSMUSG00000079470.8	Utp14b	-1.543	2.136419	-6.01958	2.53E-05	0.006787	2.775832	2.787668587	3.090031796	2.77353267	1.852541056	1.429749638	0.884991356
ENSMUSG00000033453.7	Adamts15	-1.53671	0.398352	-3.17828	0.006349	0.083118	-2.17521	0.686582462	2.120837843	0.81820172	-0.954813866	0.380280962	-0.660977013
ENSMUSG00000045871.4	Slitrk6	-1.53179	0.612974	-3.07265	0.007867	0.093671	-2.33936	1.224239248	1.901828061	0.88691447	0.899335267	0.163469573	-1.397942607
ENSMUSG00000032796.7	Lama1	-1.52996	2.444625	-4.41933	0.000518	0.020975	0.029353	2.892257488	4.105517043	2.71803755	1.494493535	2.192420947	1.265022405
ENSMUSG00000049929.7	Lpar4	-1.5298	0.383775	-3.18796	0.006225	0.082384	-2.15987	1.286975003	1.397355478	0.74605193	0.554199781	-1.586552174	-0.095379837
ENSMUSG00000044288.6	Cnr1	-1.52955	0.527416	-3.80052	0.001791	0.039598	-1.15029	1.224239248	1.901828061	0.88691447	-0.567790743	-0.402127603	0.121431552
ENSMUSG00000019027.7	Dnah1	-1.52953	0.127149	-3.74704	0.001996	0.042282	-1.30664	0.861669169	0.925287033	0.81820172	-0.131691629	-0.65366637	-1.056905689
ENSMUSG00000026768.10	Itga8	-1.52927	0.558206	-3.79933	0.001796	0.039628	-1.14539	0.686582462	1.643516065	1.56917417	0.298942726	-0.402127603	-0.446852208
ENSMUSG00000027977.15	Ndst3	-1.52913	0.727182	-3.9863	0.00123	0.032487	-0.81487	1.460306606	2.120837843	1.01523856	-0.262936162	-0.091787482	0.121431552
ENSMUSG00000024713.15	Pcsk5	-1.52785	0.579123	-3.64205	0.002471	0.047032	-1.40012	1.017788371	1.803980737	1.18857017	-0.131691629	0.508605059	-0.91251578
ENSMUSG00000024172.10	St6gal2	-1.52738	0.077288	-2.39198	0.030554	0.188589	-3.40935	0.487273654	1.328642728	0.74605193	0.202727411	-2.724055698	0.423087252
ENSMUSG00000040896.16	Kcnd3	-1.52437	-0.16045	-3.16158	0.006568	0.085239	-2.253	0.487273654	0.614946913	0.58993273	-0.407326071	-0.402127603	-1.845401584
ENSMUSG00000078302.4	Foxd1	-1.52378	0.711062	-2.9318	0.01046	0.109063	-2.57322	2.332298994	1.803980737	0.31863071	0.389140535	0.083299224	-0.660977013
ENSMUSG00000086502.1	B130055M24R	-1.52311	-0.26133	-3.0368	0.00846	0.097312	-2.45292	0.487273654	0.219018236	0.67010308	-0.011397395	-1.876058791	-1.056905689
ENSMUSG00000000782.15	Tcf7	-1.51839	-0.05333	-2.50993	0.024263	0.171081	-3.23156	0.861669169	0.614946913	0.41484602	0.474029432	-0.188002798	-2.497478281
ENSMUSG00000097136.1	Gm26772	-1.51776	-0.19787	-3.10108	0.007426	0.090926	-2.3494	0.487273654	0.925287033	0.21553721	-0.011397395	-1.586552174	-1.217370362
ENSMUSG00000027500.10	Stmn2	-1.51678	2.324297	-4.40355	0.000534	0.021261	0.006636	3.150238667	3.719283573	2.47187697	1.883905227	0.786906222	1.933571536
ENSMUSG00000036862.14	Dchs1	-1.51465	4.359069	-6.54307	1.01E-05	0.004774	3.683278	5.006181751	5.550406661	4.74836209	4.254639499	3.168335328	3.426485828
ENSMUSG00000024134.11	Six2	-1.51366	1.492757	-4.81587	0.000238	0.014985	0.692711	2.072236155	2.841455443	1.90359321	0.630148634	0.931296131	0.577809846
ENSMUSG00000088088.1	Rmrp	-1.51264	0.268887	-2.83615	0.012682	0.122354	-2.72806	0.376242341	0.925287033	1.61039683	-0.262936162	0.568726051	-1.604393485
ENSMUSG00000043531.15	Sorcs1	-1.51059	-0.11714	-2.59781	0.020402	0.158008	-3.10111	1.158650906	0.725978225	-0.01578833	-0.748362989	0.311568212	-2.134908201
ENSMUSG00000059493.13	Nhs	-1.50946	0.473279	-3.96514	0.001284	0.03302	-0.8945	1.286975003	1.585800567	0.88691447	-0.567790743	-0.001589673	-0.350636892
ENSMUSG00000022012.8	Enox1	-1.50831	0.645614	-3.70328	0.002182	0.043943	-1.29126	1.404811494	1.993458536	0.81820172	0.298942726	-0.091787482	-0.549945701
ENSMUSG00000000416.16	Cttnbp2	-1.50598	0.959959	-4.24945	0.000726	0.025028	-0.34016	1.347095996	1.948370647	1.83568914	0.554199781	-0.402127603	0.476526511
ENSMUSG00000044365.15	Cxxc4	-1.50424	2.004597	-6.19054	1.87E-05	0.006116	3.013075	2.932058497	2.841455443	2.49407271	1.494493535	1.062540664	1.202961437
ENSMUSG00000029005.4	Draxin	-1.50175	1.999027	-5.52795	6.17E-05	0.009102	1.957784	2.787668587	2.982317978	2.49407271	1.611532956	0.681936662	1.436633784
ENSMUSG00000016386.15	Mpped2	-1.50038	1.242706	-4.67127	0.000315	0.017334	0.409195	2.271544963	2.199909414	1.5267389	0.70229842	0.445869304	0.309876641
ENSMUSG00000034336.3	Ina	-1.50007	2.541745	-4.68871	0.000305	0.017041	0.51984	2.970790891	4.155966066	2.844354	1.944659258	1.363407143	1.971291202
ENSMUSG00000002900.15	Lamb1	-1.49844	2.723235	-5.66877	4.76E-05	0.008412	2.25676	3.397722069	3.99899672	3.02289214	2.442778498	1.748432074	1.728589799
ENSMUSG00000057098.14	Ebf1	-1.49844	2.915293	-6.7477	7.17E-06	0.004774	4.035949	3.411866251	4.185409844	3.44364329	2.215111135	2.07295728	2.162772347
ENSMUSG00000020333.17	Acsl6	-1.49807	1.296899	-4.60151	0.000361	0.018229	0.295819	1.754753965	2.037179913	2.09000633	0.95945626	0.735375921	-0.095379837
ENSMUSG00000040836.15	Gpr161	-1.49794	1.946652	-4.2089	0.000787	0.026068	-0.33809	2.526802018	2.765506589	2.6405588	2.442778498	0.380280962	0.923985488
ENSMUSG00000004360.8	9330159F19Ri	-1.49782	1.069603	-3.91872	0.00141	0.03453	-0.88345	1.663123489	2.120837843	1.6894684	0.836599512	-0.65366637	0.761255988
ENSMUSG00000019899.15	Lama2	-1.49515	0.884627	-3.67365	0.002317	0.045594	-1.31734	1.347095996	2.446824154	1.24200942	-0.407326071	0.626441549	0.052718802
ENSMUSG00000022422.13	Dscc1	-1.49473	0.382	-3.54353	0.003019	0.05369	-1.58407	0.776780271	1.180544089	1.29353973	0.389140535	-0.29109629	-1.056905689
ENSMUSG00000027217.13	Tspan18	-1.49266	2.231397	-3.98404	0.001236	0.032515	-0.76517	3.199991702	3.784871914	2.06056255	1.648527164	1.257796956	1.436633784
ENSMUSG00000025650.12	Col7a1	-1.49157	0.688338	-4.28325	0.000678	0.024077	-0.33846	1.565276166	1.525679575	1.18857017	0.298942726	-0.188002798	-0.260439084
ENSMUSG00000032289.15	Thsd4	-1.48906	0.281496	-3.54093	0.003035	0.05369	-1.6038	0.941839517	1.643516065	0.58993273	-0.748362989	-0.188002798	-0.549945701
ENSMUSG00000070576.4	Mn1	-1.48878	3.41927	-6.00507	2.59E-05	0.006815	2.824762	3.966005828	4.775221744	3.75066856	3.092939265	2.500910667	2.429876417
ENSMUSG00000103916.1	Gm38071	-1.48854	-0.26316	-3.10303	0.007397	0.090735	-2.35361	0.487273654	0.219018236	0.58993273	-0.131691629	-1.139093197	-1.604393485
ENSMUSG00000001986.16	Gria3	-1.48708	0.119248	-3.61389	0.002617	0.048717	-1.51444	0.686582462	0.925287033	0.95250281	-0.262936162	-1.139093197	-0.446852208
ENSMUSG00000027254.13	Map1a	-1.48469	2.979355	-5.68633	4.61E-05	0.008366	2.287892	3.480579001	4.359108008	3.34939296	2.620870821	1.919800492	2.146377909
ENSMUSG00000027833.16	Shox2	-1.48378	1.349114	-3.73247	0.002056	0.042387	-1.19314	2.577876203	2.380481659	1.29353973	1.275483753	0.445869304	0.121431552
ENSMUSG00000037541.19	Shank2	-1.4826	0.685424	-3.34314	0.00454	0.068762	-1.8864	0.487273654	1.948370647	1.76443046	0.554199781	-0.29109629	-0.350636892
ENSMUSG00000101609.1	Kcnq1ot1	-1.48252	5.384532	-7.84061	1.24E-06	0.002902	5.73169	6.182566399	6.264562372	5.90255642	5.241078819	4.291638109	4.424787541
ENSMUSG00000031785.15	Adgrg1	-1.48208	2.878853	-6.56967	9.69E-06	0.004774	3.752168	3.45348534	4.13599765	3.33716666	2.139162282	1.965243463	2.242061257
ENSMUSG00000050908.12	Tvp23a	-1.48162	0.281787	-3.81918	0.001725	0.03864	-1.16191	1.224239248	0.829071718	0.95250281	-0.131691629	-0.402127603	-0.781271247
ENSMUSG00000106320.1	Gm42992	-1.48001	1.010519	-3.56386	0.002897	0.052591	-1.49682	1.460306606	2.571003565	1.24200942	0.95945626	-0.29109629	0.121431552
ENSMUSG00000021983.15	Atp8a2	-1.47967	0.117166	-3.13465	0.006937	0.087573	-2.26525	0.487273654	0.829071718	1.29353973	-1.485328583	0.239418426	-0.660977013
ENSMUSG00000020354.15	Sgcd	-1.47922	-0.04897	-3.29649	0.004992	0.072879	-2.03215	0.590367147	1.015484842	0.41484602	-0.262936162	-0.65366637	-1.397942607
ENSMUSG00000044433.15	Camsap3	-1.47907	0.534635	-3.74416	0.002008	0.042282	-1.2388	1.117788371	1.803980737	1.01523856	-0.131691629	0.163469573	-0.660977013
ENSMUSG00000042581.14	Thsd7b	-1.47479	1.24182	-4.07299	0.001033	0.030012	-0.60331	1.840910608	2.739275048	1.43792963	0.554199781	0.568726051	0.309876641
ENSMUSG00000083757.1	Rps15a-ps4	-1.47218	2.373778	-5.14001	0.000128	0.011055	1.331054	3.354437971	2.88995396	3.08270153	1.227389465	2.453862094	1.234325608
ENSMUSG00000034245.10	Hdac11	-1.47134	0.120351	-3.30143	0.004942	0.072641	-2.00279	0.686582462	1.462943819	0.41484602	-0.131691629	-0.65366637	-1.056905689
ENSMUSG00000021268.17	Meg3	-1.47046	4.179628	-5.9468	2.88E-05	0.006901	2.664009	4.884670994	5.496611532	4.37054279	3.529621758	3.783738942	3.012579411
ENSMUSG00000020814.13	Mxra7	-1.47036	0.7	-3.5344	0.003076	0.054094	-1.56624	1.615029201	2.037179913	0.74605193	0.202727411	-0.522421837	0.121431552
ENSMUSG00000047497.9	Adamts12	-1.46821	0.059228	-3.26748	0.005296	0.075533	-2.0637	0.255948108	1.525679575	0.67010308	-0.748362989	-0.798056279	-0.549945701
ENSMUSG00000048191.15	Muc6	-1.46644	0.651506	-3.62271	0.00257	0.048573	-1.42339	1.663123489	1.752450437	0.74605193	0.298942726	-0.001589673	-0.549945701
ENSMUSG00000097451.9	Rian	-1.46582	4.210951	-9.97272	6.07E-08	0.000729	8.654415	5.078899885	5.071753062	4.67739557	3.559065536	3.565041005	3.313552299
ENSMUSG00000092746.2	Rn7s6	-1.46519	0.234122	-3.5356	0.003068	0.054085	-1.61815	1.158650906	0.925287033	0.81820172	-0.748362989	0.163469573	-0.91251578
ENSMUSG00000106375.1	Gm43361	-1.46377	0.664894	-4.22608	0.00076	0.025525	-0.43445	1.615029201	1.180544089	1.34329276	0.202727411	-0.091787482	-0.260439084

ENSMUSG00000044681.18	Cnpy1	-1.46205	0.783069	-3.78858	0.001835	0.040041	-1.13199	1.224239248	1.643516065	1.56917417	0.95945626	-0.522421837	-0.175550186
ENSMUSG00000052217.5	Hbb-bh1	-1.46187	0.957101	-2.62554	0.019312	0.154133	-3.09572	1.882133271	2.765506589	0.58993273	0.70229842	-0.958520951	0.761255988
ENSMUSG00000029126.7	Nsg1	-1.46185	1.353907	-4.25015	0.000725	0.025028	-0.28745	1.709666075	2.816580774	1.83568914	0.298942726	0.836659257	0.625904135
ENSMUSG00000104292.1	Gm38042	-1.45874	1.019013	-4.57988	0.000377	0.018298	0.215013	1.404811494	1.948370647	1.87004065	0.389140535	0.380280962	0.121431552
ENSMUSG00000075316.11	Scn9a	-1.45858	-0.18767	-2.41343	0.029305	0.185875	-3.37229	1.347095996	0.829071718	-0.45188745	-1.485328583	0.239418426	-1.604393485
ENSMUSG00000032816.14	Igdcc4	-1.45839	1.999334	-5.37727	8.17E-05	0.009818	1.708802	2.627203915	3.150999813	2.426434	1.611532956	0.884753545	1.295079639
ENSMUSG00000025964.15	Adam23	-1.45807	1.505275	-3.79778	0.001801	0.039629	-1.07605	2.141777088	2.685336241	1.90359321	0.474029432	1.77419517	0.052718802
ENSMUSG00000055609.8	Hba-x	-1.45758	1.983989	-3.00089	0.009097	0.100476	-2.56518	2.240180792	4.115748966	1.90359321	1.754137352	0.508605059	1.381667324
ENSMUSG00000054793.8	Cadm4	-1.4558	2.027723	-5.34627	8.65E-05	0.009929	1.660098	2.473853138	3.319818877	2.53746531	1.322026339	1.103763327	1.409412315
ENSMUSG00000047143.3	Dmrta2	-1.4556	0.208613	-2.87083	0.011827	0.117391	-2.67481	1.224239248	1.397355478	0.21553721	-0.567790743	0.380280962	-1.397942607
ENSMUSG00000102969.1	Gm33366	-1.45555	0.981689	-4.41037	0.000527	0.021091	-0.06911	1.999172692	1.328642728	1.72743625	0.474029432	0.380280962	-0.019430984
ENSMUSG00000005958.14	Ephb3	-1.45515	2.355661	-5.15385	0.000124	0.010981	1.354975	2.830418235	3.533333147	2.82697193	2.3105307	1.429749638	1.202961437
ENSMUSG00000034384.11	Barhl2	-1.45508	0.530333	-3.02574	0.008651	0.098298	-2.418	1.224239248	2.037179913	0.50504383	0.389140535	0.083299224	-1.056905689
ENSMUSG00000021379.1	Id4	-1.45427	-0.05211	-3.43187	0.00379	0.061196	-1.82543	0.861669169	0.614946913	0.58993273	-1.195821966	-0.402127603	-0.781271247
ENSMUSG00000048001.7	Hes5	-1.45314	0.033752	-3.23742	0.00563	0.078287	-2.11238	0.861669169	0.829071718	0.50504383	0.202727411	-1.139093197	-1.056905689
ENSMUSG00000034687.8	Fras1	-1.45229	2.127932	-4.45201	0.000485	0.02053	0.100698	2.851237224	3.438696273	2.25506558	1.974103035	1.363407143	0.884991356
ENSMUSG00000026825.17	Dnm1	-1.45126	1.861351	-4.9365	0.000188	0.013297	0.946477	2.526802018	3.110642075	2.14715024	1.322026339	1.257796956	0.803691254
ENSMUSG00000033419.15	Snap91	-1.45052	0.643413	-4.0504	0.001081	0.030547	-0.72249	1.565276166	1.525679575	1.01523856	0.202727411	-0.188002798	-0.260439084
ENSMUSG00000030376.7	Slc8a2	-1.45003	1.334345	-3.80394	0.001779	0.039454	-1.06708	2.072236155	2.478885363	1.56917417	1.126106129	1.020105398	-0.260439084
ENSMUSG00000030498.14	Gas2	-1.4494	0.291733	-3.34912	0.004485	0.06816	-1.90758	1.347095996	1.10037374	0.58993273	0.202727411	-1.139093197	-0.350636892
ENSMUSG00000008658.15	Rbfox1	-1.44833	-0.06552	-3.35724	0.004412	0.067272	-1.94202	0.376242341	0.925287033	0.74605193	-1.195821966	-0.798056279	-0.446852208
ENSMUSG00000097767.8	Miat	-1.44653	5.868991	-9.53222	1.09E-07	0.000729	8.130932	6.516202911	6.88023377	6.37129244	5.470159886	4.98053965	4.995519357
ENSMUSG00000046844.6	Vat1l	-1.44651	0.688503	-3.58551	0.002772	0.050885	-1.48116	1.158650906	1.853733772	1.18857017	0.202727411	0.508605059	-0.781271247
ENSMUSG00000032128.14	Robo3	-1.44477	4.47228	-6.63579	8.66E-06	0.004774	3.831864	5.173255319	5.492691163	4.92621612	3.56874809	4.373976385	3.298793142
ENSMUSG00000072653.12	Zfp783	-1.44443	0.118123	-2.49972	0.024755	0.172784	-3.24925	1.158650906	1.015484842	0.31863071	0.630148634	-2.238628871	-0.175550186
ENSMUSG00000043079.17	Synpo	-1.44434	2.677603	-5.01688	0.000161	0.012383	1.110106	3.133265588	4.053239764	3.00754417	2.524655505	1.640516735	1.706394053
ENSMUSG00000070304.11	Scn2b	-1.44364	0.054026	-2.17781	0.046092	0.217853	-3.72169	1.017788371	0.494652679	0.67010308	1.126106129	-2.724055698	-0.260439084
ENSMUSG00000060487.5	Samd5	-1.44362	0.008992	-2.6598	0.018042	0.150179	-3.00572	0.255948108	1.180544089	0.58993273	0.77101117	-1.139093197	-1.604393485
ENSMUSG00000021047.6	Nova1	-1.44344	1.905411	-4.53354	0.000413	0.018974	0.245235	2.743613407	2.88995396	2.25506558	1.754137352	0.380280962	1.409412315
ENSMUSG00000041592.16	Sdk2	-1.44312	1.691258	-3.93945	0.001352	0.034174	-0.8226	2.208119583	3.354795016	1.80049971	0.836599512	1.062540664	0.884991356
ENSMUSG00000040260.7	Daam2	-1.44254	1.945612	-5.34154	8.73E-05	0.009929	1.642788	2.743613407	2.88995396	2.33101443	1.611532956	1.29386621	0.803691254
ENSMUSG00000051375.15	Pcdh1	-1.44203	0.687743	-3.26276	0.005347	0.075847	-2.02088	1.840910608	1.803980737	0.58993273	0.389140535	0.163469573	-0.660977013
ENSMUSG00000021130.7	Galnt16	-1.44114	0.324848	-3.11004	0.007293	0.090256	-2.28881	0.941839517	1.699011178	0.50504383	0.389140535	-1.139093197	-0.446852208
ENSMUSG00000028369.15	Svep1	-1.44088	0.956727	-3.07496	0.00783	0.093483	-2.3379	0.590367147	2.65759125	1.80049971	0.630148634	-0.188002798	0.249755649
ENSMUSG00000025776.13	Crispld1	-1.4403	0.691429	-3.86614	0.001568	0.0366	-1.0148	1.347095996	1.256492942	1.5267389	0.298942726	0.380280962	-0.660977013
ENSMUSG00000031548.7	Sfrp1	-1.43974	1.916105	-5.03872	0.000155	0.011953	1.126761	2.500570476	3.228478566	2.25506558	1.227389465	1.020105398	1.265022405
ENSMUSG00000010476.13	Ebf3	-1.43809	3.516042	-7.02958	4.49E-06	0.000632	4.512325	4.022404999	4.729313938	3.96447545	3.038233373	2.675115396	2.666710351
ENSMUSG00000000325.15	Arvcf	-1.43734	2.843859	-6.14427	2.03E-05	0.006477	3.061677	3.467095772	3.871957531	3.31239875	2.442778498	2.354895644	1.614030034
ENSMUSG00000018604.18	Tbx3	-1.43667	1.596443	-3.68058	0.002285	0.045287	-1.28838	2.552565114	2.841455443	1.5267389	1.494493535	0.97638402	0.187019893
ENSMUSG00000058975.6	Kcnc1	-1.43638	-0.37361	-2.74362	0.015265	0.13661	-2.89608	0.376242341	0.058553564	0.41484602	0.099633918	-1.345544075	-1.845401584
ENSMUSG00000035864.14	Syt1	-1.43393	0.313616	-3.11747	0.007183	0.089586	-2.27725	1.017788371	1.328642728	0.67010308	0.099633918	0.163469573	-1.397942607
ENSMUSG00000020601.7	Trib2	-1.43309	1.84672	-5.20585	0.000113	0.010533	1.401141	2.390597634	3.04790632	2.33101443	1.07266687	0.884753545	1.35337828
ENSMUSG00000021294.7	Kif26a	-1.43259	1.335427	-3.9122	0.001429	0.034672	-0.8767	2.107425583	2.685336241	1.39138705	1.07266687	0.445869304	0.309876641
ENSMUSG00000026805.14	Barhl1	-1.43131	0.859189	-3.19458	0.006142	0.081899	-2.13258	1.286975003	2.199909414	1.24200942	1.017171757	-0.958520951	0.367592139
ENSMUSG00000022454.16	Nell2	-1.42993	2.11242	-4.68422	0.000307	0.017041	0.518517	2.743613407	3.533333147	2.33101443	1.07266687	1.61222769	1.381667324
ENSMUSG00000096870.2	Gm21816	-1.42549	-0.00257	-2.91281	0.010868	0.111652	-2.61824	0.376242341	0.614946913	0.95250281	0.298942726	-0.65366637	-1.604393485
ENSMUSG00000046402.10	Rbp1	-1.42538	0.36902	-3.25135	0.005472	0.076896	-2.05808	0.776780271	1.948370647	0.58993273	-0.131691629	-0.522421837	-0.446852208
ENSMUSG00000103657.1	Gm37204	-1.4246	-0.09881	-2.64643	0.018527	0.152011	-3.02797	0.686582462	0.614946913	0.50504383	0.099633918	-2.238628871	-0.260439084
ENSMUSG00000001497.18	Pax9	-1.42407	0.309967	-3.36581	0.004335	0.066555	-1.87842	0.861669169	1.397355478	0.74605193	0.202727411	-0.29109629	-1.056905689
ENSMUSG00000027200.17	Sema6d	-1.42357	2.077156	-5.99526	2.64E-05	0.006815	2.727019	2.912295242	2.936875008	2.53746531	1.322026339	1.583372827	1.170900228
ENSMUSG00000035948.12	Acss3	-1.42281	-0.30564	-2.65856	0.018086	0.150179	-3.01705	0.255948108	0.725978225	0.21553721	-0.131691629	-2.238628871	-0.660977013
ENSMUSG00000042035.11	Igsf3	-1.42245	3.103065	-4.41595	0.000521	0.020975	-0.04368	3.738010701	4.668058249	3.09727433	2.563914936	2.561346521	1.989787546
ENSMUSG00000022206.6	Npr3	-1.42216	1.387023	-3.59174	0.002737	0.050452	-1.44291	1.882133271	2.765506589	1.65047427	1.494493535	-0.188002798	0.71753461
ENSMUSG00000097051.1	Gm26836	-1.41904	-0.0725	-2.60056	0.020292	0.158008	-3.09626	0.861669169	0.494652679	0.50504383	0.202727411	-2.238628871	-0.260439084
ENSMUSG00000021994.14	Wnt5a	-1.41882	2.91791	-6.63383	8.69E-06	0.004774	3.858561	3.596572296	3.810294511	3.44364329	2.71106863	1.799506258	2.146377909
ENSMUSG00000038816.14	Ctnnal1	-1.41502	1.308528	-3.88549	0.001508	0.03601	-0.92431	1.754753965	2.199909414	1.99980852	0.899335267	1.257796956	-0.260439084
ENSMUSG00000063564.13	Col23a1	-1.41313	1.78465	-4.61866	0.000349	0.018054	0.38961	2.418886679	3.026373159	2.03050532	1.494493535	0.97638402	0.761255988
ENSMUSG00000093843.1	Gm25939	-1.40747	0.920645	-3.65366	0.002413	0.046328	-1.34787	1.565276166	2.037179913	1.39138705	-0.567790743	0.97638402	0.121431552
ENSMUSG00000035275.14	Raver2	-1.40643	1.069299	-3.92614	0.001389	0.034458	-0.86964	1.882133271	2.037179913	1.34329276	1.017171757	0.311568212	-0.175550186
ENSMUSG00000097265.1	Gm26803	-1.40603	-0.18746	-2.40397	0.02985	0.186992	-3.38618	-0.019686335	0.219018236	1.07535956	0.389140535	-0.65366637	-2.134908201
ENSMUSG00000034460.9	Six4	-1.40585	1.936242	-5.07815	0.000144	0.011551	1.197577	2.721070838	2.712557709	2.426434	1.883905227	0.735375921	1.138110293
ENSMUSG00000055629.4	B4galnt4	-1.40497	2.290657	-4.45814	0.000479	0.020447	0.107199	3.045269107	3.337412937	2.51593215	2.421562012	1.461810848	0.961953338
ENSMUSG00000097348.8	#N/A	-1.40433	2.063295	-5.18031	0.000118	0.010654	1.384383	2.577876203	2.791269685	2.82697193	1.754137352	1.668261725	0.761255988
ENSMUSG00000032172.7	Olfm2	-1.40177	-0.06771	-2.65628	0.018168	0.150309	-3.01243	-0.180151007	1.525679575	0.50504383	0.099633918	-1.139093197	-1.217370362
ENSMUSG00000021986.7	Amer2	-1.40083	0.455731	-3.87307	0.001546	0.036343	-1.04269	1.089938156	1.328642728	1.07535956	-0.407326071	-0.001589673	-0.350636892
ENSMUSG00000027674.16	Pex5l	-1.39922	0.640278	-3.47077	0.003501	0.058435	-1.67641	1.404811494	1.643516065	1.01523856	-0.407326071	0.735375921	-0.549945701
ENSMUSG00000010505.15	Myt1	-1.39902	3.420118	-7.42222	2.38E-06	0.003546	5.114341	3.877044395	4.482843377	3.98812672	2.922175243	2.780564695	2.469953857
ENSMUSG00000047261.9	Gap43	-1.39798	2.098049	-4.14681	0.000891	0.028111	-0.45662	2.970790891	3.228478566	2.14715024	2.139162282	1.257796956	0.844913917
ENSMUSG00000103037.1	Pcdhgb1	-1.39451	-0.41757	-2.65796	0.018108	0.150179	-3.02317	0.255948108	0.219018236	0.21553721	-0.262936162	-0.798056279	-2.134908201
ENSMUSG00000029510.15	Gpc2	-1.39244	1.554755	-4.15286	0.00088	0.027989	-0.4428	2.332298994	2.600447343	1.76443046	1.534570974	0.786906222	0.309876641
ENSMUSG00000039153.16	Runx2	-1.39098	0.788943	-3.67695	0.002302	0.045489	-1.31887	1.922210711	1.585800567	1.01523856	-0.262936162	0.568726051	-0.095379837
ENSMUSG00000033981.14	Gria2	-1.38862	1.87525	-4.17151	0.000848	0.027304	-0.40502	2.361742772	2.765506589	2.51593215	1.275483753	1.965243463	0.367592139
ENSMUSG00000038253.6	Hoxa5	-1.38792	1.215441	-4.1275	0.000926	0.028569	-0.51018	1.513745865	2.274871471	1.87004065	1.07266687	0.508605059	0.052718802
ENSMUSG00000015653.13	Steap2	-1.38658	0.916867	-3.9299	0.001379	0.034436	-0.87932	1.565276166	1.993458536	1.29353973	0.630148634	-0.29109629	0.309876641
ENSMUSG00000040037.13	Negr1	-1.3865	-0.30725	-2.28873	0.0373	0.204049	-3.54875	0.124703574	0.829071718	0.1045059	-0.567790743	0.163469573	-2.497478281
ENSMUSG00000066842.18	Hmcn1	-1.38578	3.920938	-6.93136	5.28E-06	0.004721	4.349441	4.740814774	4.794457963	4.25831124	3.662159029	3.271428821	2.798454606
ENSMUSG00000106049.1	Gm43019	-1.38577	0.028101	-2.65907	0.018068	0.150179	-3.0066	0.255948108	1.10037374	0.74605193	0.389140535	-1.876058791	-0.446852208
ENSMUSG00000018169.12	Mfng	-1.3851	1.287898	-4.3136	0.000638	0.023329	-0.18383	1.754753965	2.510249534	1.6894684	0.836599512	0.626441549	0.309876641
ENSMUSG00000059743.12	Fdps	-1.3844	-0.03533	-2.55621	0.02215	0.164159	-3.16247	0.590367147	0.363408146	0.95250281	-1.195821966	0.681936662	-1.604393485
ENSMUSG00000079481.11	Nhsl2	-1.38292	1.619515	-4.14229	0.000899	0.028188	-0.45926	2.500570476	2.120837843	2.17489523	1.914602024	0.380280962	0.625904135
ENSMUSG00000030584.13	Dpf1	-1.38166	0.930392	-3.10969	0.007298	0.090256	-2.27796	1.513745865	2.380481659	1.01523856	0.202727411	1.020105398	-0.549945701
ENSMUSG00000041734.15	Kirrel	-1.38079	3.101308	-4.8735	0.000213	0.014141	0.817041	3.726739589	4.2047104	3.38546221	3.281384354	1.896530713	2.113019312
ENSMUSG00000069270.5	Hist1h2ac	-1.38047	1.022335	-3.85276	0.001611	0.037216	-0.99942	1.663123489	2.237877264	1.24200942	0.77101117	0.239418426	-0.019430984
ENSMUSG00000022483.16	Col2a1	-1.37992	4.42806	-6.04965	2.40E-05	0.006757	2.822776	5.105251212	5.659341032	4.57699367	4.15531805	3.527663395	3.543789738
ENSMUSG00000039323.18	Igfbp2	-1.37936	2.291281	-2.75557	0.014905	0.134503	-3.06921	3.06330303	3.771990623	2.09000633	1.883905227	2.688725828	0.249755649
ENSMUSG00000028883.17	Sema3a	-1.37818	2.105528	-4.12301	0.000934	0.02863	-0.50129	2.787668587	2.816580774	2.62052105	2.263609653	1.668261725	0.476526511
ENSMUSG00000022044.14	Stmn4	-1.37453	0.162383	-2.80676	0.013452	0.126608	-2.77631	1.224239248	1.10037374	0.21553721	-0.407326071	0.239418426	-1.397942607
ENSMUSG00000069601.13	Ank3	-1.37451	4.620828	-5.19477	0.000115	0.010533	1.242224	5.177400998	5.917996997	4.8721607	3.53950327	4.60686118	3.611046176
ENSMUSG00000031209.14	Heph	-1.37298	0.561677	-3.32964	0.004667	0.070051	-1.91561	1.286975003	1.525679575	0.88691447	0.202727411	0.380280962	-0.91251578
ENSMUSG00000108238.1	Gm43984	-1.37221	-0.21229	-3.11097	0.007279	0.090189	-2.33523	0.487273654	0.614946913	0.31863071	-0.567790743	-1.345544075	-0.781271247
ENSMUSG00000028137.9	Celf3	-1.37053	2.937819	-5.16668	0.000121	0.010788	1.368119	3.584135193	4.214264707	3.09727433	2.484297768	2.371868722	1.875075887
ENSMUSG00000002265.15	Peg3	-1.37015	4.747267	-6.12304	2.10E-05	0.006568	2.927983	5.495598519	5.724307472	5.02860579	4.637068048	4.085873168	3.51215042
ENSMUSG00000105950.1	Gm43679	-1.36948	-0.49617	-2.17567	0.046279	0.217853	-3.70063	0.376242341	-0.569477658	0.50504383	0.554199781	-2.238628871	-1.604393485
ENSMUSG00000046456.14	Tmem150b	-1.36923	0.177101	-3.41205	0.003946	0.062723	-1.82006	0.861669169	0.829071718	0.81820172	-0.011397395	-0.522421837	-0.91251578
ENSMUSG00000056222.14	Spock1	-1.3675	-0.07169	-2.47155	0.026161	0.176763	-3.28826	0.255948108	1.699011178	0.1045059	-1.847898663	-0.091787482	-0.549945701
ENSMUSG00000063415.12	Cyp26b1	-1.36741	1.917286	-5.79412	3.79E-05	0.008043	2.377005	2.446631669	2.685336241	2.6405588	1.367114228	1.329055639	1.0350168
ENSMUSG00000040270.16	Bach2	-1.36739	2.760201	-5.22626	0.000108	0.010484	1.48261	3.480579001	3.607715344	3.15413667	2.728450708	2.153688552	1.436633784
ENSMUSG00000016918.15	Sulf1	-1.36439	2.892239	-6.45774	1.17E-05	0.004774	3.575829	3.749194439	3.810294511	3.1817489	2.355973671	2.230140613	2.026083675
ENSMUSG00000054967.6	Zfp647	-1.36419	0.657626	-3.38162	0.004198	0.065275	-1.82351	1.754753965	1.643516065	0.67010308	0.298942726	-0.402127603	-0.019430984
ENSMUSG00000102555.1	6430511E19Ri	-1.36262	-0.0741	-2.63697	0.018879	0.152876	-3.04156	0.590367147	1.180544089	-0.01578833	0.202727411	-0.798056279	-1.604393485
ENSMUSG00000057378.14	Ryr3	-1.36007	-0.24101	-2.43051	0.028346	0.182324	-3.34743	0.487273654	1.015484842	-0.14703287	-1.195821966	0.239418426	-1.845401584
ENSMUSG00000036422.10	Pcdh8	-1.35996	1.534834	-4.80846	0.000241	0.014998	0.689355	2.446631669	2.310940726	1.96844435	0.630148634	0.681936662	1.170900228
ENSMUSG00000036158.12	Prickle1	-1.35848	0.703424	-3.61353	0.002618	0.048717	-1.43241	1.347095996	1.752450437	1.01523856	0.554199781	-0.001589673	-0.446852208
ENSMUSG00000057706.6	Mex3b	-1.35776	3.02288	-4.34503	0.0006	0.022799	-0.1696	3.657198655	4.393155489	3.09727433	2.891640875	1.695483194	2.402525929
ENSMUSG00000086181.1	C230034O21R	-1.35734	0.289459	-2.60391	0.020158	0.157448	-3.09331	1.158650906	1.643516065	0.1045059	-0.011397395	0.239418426	-1.397942607
ENSMUSG00000018411.17	Mapt	-1.35693	1.63412	-4.07578	0.001027	0.030012	-0.57792	2.175329648	3.069122806	1.80049971	0.77101117	1.143840766	0.844913917
ENSMUSG00000014329.14	Bicc1	-1.35637	0.960183	-3.84135	0.001649	0.037697	-1.02424	1.460306606	2.160915282	1.29353973	0.70229842	0.163469573	-0.019430984
ENSMUSG00000103088.1	Pcdhgb6	-1.35626	0.916102	-3.61648	0.002603	0.048717	-1.41043	1.565276166	1.462943819	1.61039683	0.899335267	0.508605059	-0.549945701
ENSMUSG00000086537.7	Nespas	-1.35616	1.099676	-3.46327	0.003555	0.058821	-1.66845	1.404811494	1.993458536	1.80049971	1.534570974	-0.188002798	0.052718802
ENSMUSG00000026587.15	Astn1	-1.35609	2.568415	-5.7034	4.47E-05	0.008335	2.310549	3.098708366	3.622142414	2.99203117	2.333431102	1.77419517	1.58998456
ENSMUSG00000102573.1	Gm7265	-1.35584	0.952694	-4.0928	0.000993	0.029675	-0.6004	1.404811494	1.993458536	1.48301752	0.630148634	0.083299224	0.121431552
ENSMUSG00000107201.1	5930420M18F	-1.35544	-0.01378	-2.49359	0.025055	0.173779	-3.25638	0.861669169	-0.122018681	1.01523856	0.099633918	-0.091787482	-1.845401584
ENSMUSG00000032625.14	Thsd7a	-1.35307	3.402136	-5.27209	9.95E-05	0.010268	1.522538	3.897293078	4.768752249	3.59507603	3.052105547	2.590640828	2.508947988
ENSMUSG00000036225.14	Kctd1	-1.34901	1.242951	-4.51961	0.000424	0.019179	0.159472	2.107425583	2.037179913	1.61039683	0.77101117	0.508605059	0.423087252
ENSMUSG00000031734.12	Irx3	-1.34837	1.714995	-4.20266	0.000797	0.026202	-0.34946	2.526802018	2.739275048	1.87004065	1.494493535	1.182834898	0.476526511
ENSMUSG00000046314.15	Stxbp6	-1.34771	0.215725	-3.1115	0.007271	0.090174	-2.29259	0.686582462	0.829071718	1.01523856	0.474029432	-0.798056279	-0.91251578

ENSMUSG00000044349.15	Snhg11	-1.34768	2.203336	-5.28084	9.79E-05	0.010266	1.568006	2.809201749	2.913605228	2.82697193	1.883905227	1.824380927	0.961953338
ENSMUSG00000031517.7	Gpm6a	-1.34494	1.301433	-3.59224	0.002734	0.050452	-1.4414	1.798475342	2.65759125	1.5267389	1.07266687	-0.091787482	0.844913917
ENSMUSG00000027996.13	Sfrp2	-1.34483	0.955425	-3.14746	0.006759	0.086299	-2.21391	1.565276166	2.380481659	0.95250281	1.07266687	-0.29109629	0.052718802
ENSMUSG00000042671.12	Rgs8	-1.34453	0.199485	-2.85321	0.012254	0.119766	-2.70257	1.709666075	0.829071718	0.1045059	-0.262936162	-0.522421837	-0.660977013
ENSMUSG00000062380.3	Tubb3	-1.34119	4.26519	-4.8082	0.000241	0.014998	0.543412	4.740814774	5.693898254	4.41193443	3.872223421	3.014712139	3.857558126
ENSMUSG00000052387.15	Trpm3	-1.34021	-0.14195	-2.66175	0.017972	0.149791	-3.00619	0.124703574	1.462943819	0.1045059	-1.195821966	-0.29109629	-1.056905689
ENSMUSG00000050530.14	Fam171a1	-1.3388	2.066084	-4.35505	0.000588	0.022413	-0.07463	2.787668587	3.150999813	2.22883404	2.086213402	1.143840766	0.998947545
ENSMUSG00000079466.2	Prdm12	-1.33875	0.206843	-3.42469	0.003846	0.061912	-1.7965	0.590367147	1.180544089	0.88691447	-0.567790743	-0.402127603	-0.446852208
ENSMUSG00000103928.1	Gm37893	-1.33849	-0.1418	-2.2754	0.038267	0.205896	-3.57236	1.224239248	0.058553564	0.1045059	0.298942726	-0.402127603	-2.134908201
ENSMUSG00000050272.9	Dscam	-1.33841	0.13221	-3.15727	0.006626	0.08574	-2.22757	0.487273654	1.328642728	0.67010308	-0.954813866	-0.188002798	-0.549945701
ENSMUSG00000104469.1	Gm37663	-1.33795	-0.14589	-2.97128	0.009659	0.104187	-2.539	0.487273654	0.219018236	0.74605193	-0.131691629	-1.139093197	-1.056905689
ENSMUSG00000096010.2	Hist4h4	-1.3368	1.309238	-2.96604	0.009761	0.104875	-2.54808	2.30224176	2.414034219	1.24200942	1.534570974	-0.522421837	0.884991356
ENSMUSG00000054863.8	Fam19a5	-1.33585	0.222732	-3.08702	0.007641	0.092209	-2.33134	0.590367147	1.397355478	0.67010308	-0.262936162	-0.001589673	-1.056905689
ENSMUSG00000023972.9	Ptk7	-1.33532	3.271629	-5.43999	7.27E-05	0.009286	1.838054	3.897293078	4.474916461	3.45500315	2.876127876	2.71556744	2.210866635
ENSMUSG00000068130.11	Zfp442	-1.33477	0.369932	-2.84996	0.012334	0.120323	-2.70482	0.941839517	1.180544089	0.88691447	0.099633918	0.508605059	-1.397942607
ENSMUSG00000000901.15	Mmp11	-1.33159	0.549971	-3.41993	0.003883	0.062178	-1.76758	1.709666075	1.256492942	0.74605193	-0.131691629	-0.402127603	0.121431552
ENSMUSG00000026883.17	Dab2ip	-1.32754	2.939011	-5.45465	7.07E-05	0.009244	1.882504	3.57158994	3.758993282	3.40901694	3.024226515	1.77419517	2.096046233
ENSMUSG00000043290.6	Zfp784	-1.32592	0.16535	-2.74279	0.015291	0.13661	-2.876	1.224239248	0.725978225	0.50504383	0.298942726	-1.586552174	-0.175550186
ENSMUSG00000026347.13	Tmem163	-1.32407	0.57858	-3.15791	0.006617	0.085711	-2.19898	1.565276166	1.585800567	0.67010308	-0.567790743	0.568726051	-0.350636892
ENSMUSG00000027797.15	Dclk1	-1.32289	2.900678	-5.66961	4.76E-05	0.008412	2.259633	3.411866251	4.031786655	3.24855227	2.639367164	1.942700894	2.129795025
ENSMUSG00000102142.1	Gm26930	-1.32228	0.675077	-3.50844	0.003243	0.055858	-1.60977	1.286975003	0.925287033	1.65047427	0.630148634	-0.091787482	-0.350636892
ENSMUSG00000022464.13	Slc38a4	-1.32149	1.153796	-4.14481	0.000895	0.028111	-0.48693	1.663123489	2.274871471	1.5267389	0.70229842	0.445869304	0.309876641
ENSMUSG00000036617.16	Etl4	-1.32136	1.340961	-3.73389	0.00205	0.042346	-1.19005	2.552565114	2.274871471	1.48301752	0.899335267	1.182834898	0.249755649
ENSMUSG00000105293.1	Gm42843	-1.32087	-0.11451	-3.08868	0.007615	0.092209	-2.35782	0.376242341	0.725978225	0.58993273	-1.195821966	-0.522421837	-0.660977013
ENSMUSG00000037003.15	Tns2	-1.31999	0.655211	-3.6574	0.002395	0.046328	-1.36423	1.347095996	1.462943819	1.13307505	0.389140535	-0.522421837	0.121431552
ENSMUSG00000053414.7	Hunk	-1.31901	2.216552	-4.24148	0.000737	0.0253	-0.28808	2.743613407	3.593142542	2.33101443	1.974103035	1.220802748	1.436633784
ENSMUSG00000071064.13	Zfp827	-1.31716	2.80384	-5.13257	0.00013	0.01114	1.312974	3.439745284	3.822839764	3.08270153	2.620870821	2.266899163	1.58998456
ENSMUSG00000031290.14	Lrch2	-1.31568	2.068191	-5.32079	9.08E-05	0.009985	1.624038	2.627203915	3.04790632	2.49407271	1.754137352	1.220802748	1.265022405
ENSMUSG00000006720.16	Zfp184	-1.31331	0.673827	-3.15225	0.006694	0.085955	-2.20568	1.158650906	1.525679575	1.18857017	0.70229842	0.380280962	-0.91251578
ENSMUSG00000024598.8	Fbn2	-1.313	4.800936	-5.2284	0.000108	0.010484	1.287996	5.370822066	5.938316982	5.01715577	4.858474011	3.945538053	3.675306375
ENSMUSG00000031673.5	Cdh11	-1.31284	3.481645	-6.24178	1.71E-05	0.005836	3.225293	3.985051584	4.567280479	3.848667	3.257635752	2.576068027	2.655168729
ENSMUSG00000035236.17	Scai	-1.31226	2.818391	-5.55703	5.85E-05	0.008819	2.064659	3.397722069	3.405718951	3.4995687	2.891640875	1.965243463	1.750449233
ENSMUSG00000025104.13	Hdgfl3	-1.3113	2.875162	-6.03335	2.47E-05	0.006757	2.878914	3.771305141	3.607715344	3.19535933	2.620870821	1.942700894	2.113019312
ENSMUSG00000025529.14	Zfp711	-1.30966	1.176773	-4.01636	0.001158	0.03138	-0.7046	1.460306606	2.160915282	1.83568914	1.017171757	0.163469573	0.423087252
ENSMUSG00000035936.6	Aldh5a1	-1.30889	0.763272	-3.19487	0.006138	0.081899	-2.13267	1.460306606	0.829071718	1.80049971	0.95945626	-0.522421837	0.052718802
ENSMUSG00000063681.16	Crb1	-1.30855	-0.10115	-2.83863	0.012618	0.122107	-2.73783	0.487273654	1.180544089	0.1045059	-1.195821966	-0.402127603	-0.781271247
ENSMUSG00000045215.16	Asxl3	-1.30841	3.043087	-4.83898	0.000227	0.014674	0.7579	3.814535193	3.976715272	3.23543677	3.038233373	2.421621757	1.771982394
ENSMUSG00000074480.4	Mex3a	-1.30795	4.144616	-5.17427	0.00012	0.010705	1.25333	4.75197688	5.335225378	4.30300005	4.034744387	2.937009782	3.505738432
ENSMUSG00000035357.16	Pdzrn3	-1.30688	1.791443	-4.02504	0.001138	0.03138	-0.66949	2.500570476	2.95977541	1.87004065	1.534570974	1.257796956	0.625904135
ENSMUSG00000108563.1	Gm44686	-1.30581	0.220006	-2.88031	0.011604	0.115859	-2.6591	1.663123489	0.219018236	0.67010308	-0.262936162	-0.188002798	-0.781271247
ENSMUSG00000063430.9	Wscd2	-1.30532	0.381123	-3.07137	0.007888	0.093832	-2.34802	1.286975003	1.585800567	0.31863071	-0.262936162	-0.29109629	-0.350636892
ENSMUSG00000000948.16	Gm38393	-1.30524	1.741878	-4.75679	0.000267	0.015701	0.627364	1.961204842	2.712557709	2.49407271	1.17763643	1.220802748	0.884991356
ENSMUSG00000005338.13	Cadm3	-1.30254	1.438201	-4.40912	0.000528	0.021091	-0.00287	1.999172692	2.346130154	1.96844435	0.899335267	0.311568212	1.104557733
ENSMUSG00000021714.14	Cenpk	-1.30202	0.187927	-3.08737	0.007636	0.092209	-2.33283	0.686582462	1.015484842	0.74605193	0.298942726	-0.958520951	-0.660977013
ENSMUSG00000104118.1	Gm37298	-1.30048	1.108748	-3.15071	0.006715	0.085977	-2.2133	1.460306606	1.180544089	2.426434	1.367114228	-0.091787482	0.309876641
ENSMUSG00000040254.11	Sema3d	-1.29917	0.216035	-2.89456	0.011276	0.113704	-2.63672	1.404811494	0.829071718	0.31863071	-0.011397395	-0.188002798	-1.056905689
ENSMUSG00000029595.9	Lhx5	-1.29908	1.022542	-3.54066	0.003037	0.05369	-1.53593	1.709666075	2.199909414	1.18857017	0.099633918	0.884753545	0.052718802
ENSMUSG00000033565.16	Rbfox2	-1.29842	4.504431	-6.5671	9.73E-06	0.004774	3.711749	5.07446082	5.527596778	4.82478437	4.358766886	3.67853006	3.562445537
ENSMUSG00000037138.16	Aff3	-1.29726	2.550849	-4.71989	0.000287	0.016299	0.574863	3.116090444	3.47093661	2.92825526	2.524655505	2.030831804	1.234325608
ENSMUSG00000001864.13	Aif1l	-1.29214	-0.01943	-2.31131	0.035715	0.201284	-3.52498	0.376242341	1.699011178	-0.14703287	0.202727411	-1.586552174	-0.660977013
ENSMUSG00000029636.10	Wasf3	-1.29096	1.661195	-4.87036	0.000214	0.014141	0.813125	2.175329648	2.629302206	2.11886119	1.227389465	0.931296131	0.884991356
ENSMUSG00000038777.19	Sema6c	-1.29095	2.784803	-5.8895	3.19E-05	0.00726	2.63433	3.116090444	3.79763921	3.38546221	2.11293074	2.421621757	1.875075887
ENSMUSG00000041605.16	5730559C18Ri	-1.29012	0.714803	-2.76012	0.01477	0.133664	-2.85814	1.347095996	1.752450437	0.95250281	1.126106129	-1.139093197	0.249755649
ENSMUSG00000074793.10	Hspa12b	-1.29004	0.678709	-3.40967	0.003965	0.062816	-1.77504	0.776780271	1.853733772	1.39138705	-0.011397395	-0.188002798	0.249755649
ENSMUSG00000074415.10	3110039I08Ril	-1.2882	3.415986	-5.94986	2.86E-05	0.006901	2.727803	3.887204261	3.96544416	4.20548355	3.458488581	2.590640828	2.388653755
ENSMUSG00000097709.7	2810429I04Ril	-1.28691	-0.02928	-2.67889	0.017369	0.146735	-2.97723	0.487273654	0.725978225	0.31863071	0.099633918	-0.402127603	-1.604393485
ENSMUSG00000024420.8	Zfp521	-1.28643	3.100279	-5.65326	4.90E-05	0.008542	2.226059	3.749194439	3.954084298	3.46627426	2.952076729	2.546473245	1.933571536
ENSMUSG00000029168.14	Dpysl5	-1.28562	3.394783	-5.57189	5.69E-05	0.008819	2.064409	4.049798765	4.514122543	3.55340685	3.106297648	2.675115396	2.469953857
ENSMUSG00000036885.14	Arhgef26	-1.28556	1.13839	-3.10396	0.007383	0.090726	-2.2957	1.663123489	2.07961518	1.56917417	1.410835606	-0.65366637	0.761255988
ENSMUSG00000032850.16	Rnft2	-1.28452	0.986557	-3.81915	0.001725	0.03864	-1.05935	1.224239248	2.120837843	1.56917417	0.474029432	0.163469573	0.367592139
ENSMUSG00000028212.16	Ccne2	-1.28432	1.194694	-4.1195	0.000941	0.028746	-0.52539	1.798475342	2.07961518	1.61039683	1.017171757	0.239418426	0.423087252
ENSMUSG00000004035.12	Gstm7	-1.28322	0.038412	-3.03808	0.008438	0.097299	-2.42114	0.861669169	0.614946913	0.50504383	-0.011397395	-0.958520951	-0.781271247
ENSMUSG00000038227.15	Hoxa9	-1.28306	0.759309	-2.93187	0.010459	0.109063	-2.57468	1.347095996	2.199909414	0.67010308	0.70229842	0.083299224	-0.446852208
ENSMUSG00000032609.12	Klhdc8b	-1.28155	1.421585	-4.5817	0.000376	0.018298	0.291607	1.840910608	1.984370647	2.35546747	0.836599512	0.786906222	0.761255988
ENSMUSG00000022456.15	Sep-03	-1.28099	3.471406	-5.1429	0.000127	0.011055	1.277001	4.058815639	4.775221744	3.56393752	2.995798107	2.605067899	2.829593117
ENSMUSG00000019647.16	Sema6a	-1.2796	3.116811	-6.51066	1.07E-05	0.004774	3.673507	3.726739589	4.084832889	3.46627426	2.745625852	2.302744362	2.374646897
ENSMUSG00000000093.6	Tbx2	-1.27906	2.499496	-4.82523	0.000234	0.014899	0.769114	3.133265588	3.664577681	2.67981823	2.086213402	1.461810848	1.971291202
ENSMUSG00000022995.16	Enah	-1.27871	4.810577	-6.86051	5.94E-06	0.004774	4.186247	5.431090818	5.773607088	5.11707457	4.618542015	4.085873168	3.837273854
ENSMUSG00000078794.4	Dact3	-1.27778	1.876952	-3.20771	0.00598	0.080654	-2.17563	2.526802018	3.228478566	1.76443046	1.820479846	1.55392905	0.367592139
ENSMUSG00000030077.11	Chl1	-1.27712	1.118776	-3.34131	0.004557	0.068941	-1.8811	1.754753965	2.571003565	1.07535956	0.389140535	0.445869304	0.476526511
ENSMUSG00000041482.16	Piezo2	-1.27655	0.091206	-2.48805	0.025329	0.174569	-3.26649	0.776780271	1.585800567	-0.14703287	0.202727411	-0.958520951	-0.91251578
ENSMUSG00000056947.5	Mab21l1	-1.27562	0.205091	-2.6251	0.019329	0.154133	-3.05893	1.347095996	1.015484842	0.1045059	0.474029432	-0.65366637	-1.056905689
ENSMUSG00000017491.8	Rarb	-1.27555	1.032053	-3.44401	0.003697	0.06021	-1.70265	1.663123489	1.853733772	1.39138705	1.126106129	0.508605059	-0.350636892
ENSMUSG00000107756.1	Gm44164	-1.27526	-0.29228	-2.8302	0.012834	0.123295	-2.7626	0.686582462	0.219018236	0.1045059	-0.748362989	-0.798056279	-1.217370362
ENSMUSG00000028175.15	Depdc1a	-1.27492	-0.19014	-2.38818	0.030781	0.18895	-3.40908	0.861669169	-0.328469559	0.58993273	0.298942726	-0.958520951	-1.604393485
ENSMUSG00000056832.14	Ttc26	-1.27475	1.122281	-4.04841	0.001086	0.030593	-0.65459	1.513745865	2.160915282	1.61039683	0.630148634	0.568726051	0.249755649
ENSMUSG00000026447.16	Pik3c2b	-1.27396	2.992014	-4.90917	0.000199	0.013738	0.892914	3.680753383	4.105517043	3.12598563	2.442778498	2.702209057	1.894839142
ENSMUSG00000006586.15	Runx1t1	-1.27394	2.851342	-4.5506	0.000399	0.018566	0.238374	3.584135193	3.976715272	2.92825526	2.728450708	1.695483194	2.195012684
ENSMUSG00000042834.14	Nrep	-1.27319	3.82807	-5.36903	8.29E-05	0.009818	1.652475	4.365381349	4.856787096	4.13660674	3.856499069	2.661375339	3.091771578
ENSMUSG00000034324.16	Tmem132c	-1.27188	2.914248	-4.65019	0.000328	0.017703	0.41824	3.24808599	4.063847864	3.2871982	3.010082333	1.942700894	1.933571536
ENSMUSG00000048540.8	Nhlh2	-1.26885	2.836619	-4.38244	0.000557	0.021658	-0.07696	3.294628576	3.919457955	3.22220094	2.71106863	1.363407143	2.508947988
ENSMUSG00000026442.14	Nfasc	-1.26753	3.257093	-5.31307	9.21E-05	0.01	1.609772	4.022404999	4.367695504	3.33716666	2.620870821	2.805765249	2.388653755
ENSMUSG00000028664.14	Ephb2	-1.26726	3.710803	-5.23942	0.000106	0.010484	1.427362	4.415380735	4.880983807	3.75985773	3.426895457	2.805765249	2.975937567
ENSMUSG00000104358.1	Gm37127	-1.26724	-0.0696	-2.49168	0.025149	0.173779	-3.25864	0.861669169	0.925287033	-0.14703287	0.298942726	-1.139093197	-1.217370362
ENSMUSG00000029287.14	Tgfbr3	-1.26685	1.0427	-3.19161	0.006179	0.08218	-2.13976	1.513745865	2.07961518	1.56917417	-0.567790743	1.29386621	0.367592139
ENSMUSG00000052302.5	Tbc1d30	-1.26586	0.211743	-2.80755	0.013431	0.126604	-2.77401	0.686582462	1.585800567	0.31863071	-0.011397395	-0.958520951	-0.350636892
ENSMUSG00000027985.14	Lef1	-1.26475	1.806348	-4.57692	0.000379	0.018298	0.318401	2.107425583	2.936875008	2.30613976	1.322026339	0.931296131	1.234325608
ENSMUSG00000060568.14	Fam78b	-1.26408	1.243494	-3.47524	0.003469	0.058192	-1.64772	1.840910608	2.510249534	1.29353973	0.95945626	0.735375921	0.121431552
ENSMUSG00000103081.1	Pcdhgb8	-1.26278	0.193558	-3.02893	0.008596	0.097998	-2.42506	0.861669169	1.10037374	0.50504383	0.099633918	-0.958520951	-0.446852208
ENSMUSG00000100826.6	Snhg14	-1.26272	1.886739	-4.0586	0.001064	0.030302	-0.61094	2.361742772	2.600447343	2.4493344	2.139162282	1.143840766	0.625904135
ENSMUSG00000024112.16	Cacna1h	-1.2626	2.643488	-3.64719	0.002445	0.046675	-1.45443	3.439745284	3.931092655	2.51593215	1.974103035	2.590640828	1.409412315
ENSMUSG00000021685.11	Otp	-1.26244	0.748182	-2.95844	0.009913	0.10582	-2.52989	1.460306606	2.07961518	0.74605193	0.099633918	-0.522421837	0.625904135
ENSMUSG00000004415.15	Col26a1	-1.262	2.453937	-4.37805	0.000562	0.021658	-0.04998	2.871937504	3.771990623	2.6405588	2.239564179	1.493175018	1.706394053
ENSMUSG00000048450.10	Msx1	-1.25687	1.773641	-3.9915	0.001217	0.032224	-0.73015	2.208119583	2.88995396	2.06056255	1.684596418	1.220802748	0.577809846
ENSMUSG00000039057.9	Myo16	-1.25528	0.661719	-3.04372	0.008342	0.096751	-2.38687	1.460306606	1.901828061	0.58993273	0.298942726	-0.402127603	0.121431552
ENSMUSG00000028597.11	Gpx7	-1.25494	-0.28213	-2.22406	0.042214	0.212176	-3.64012	0.124703574	0.494652679	0.31863071	0.389140535	-2.238628871	-0.781271247
ENSMUSG00000029869.7	Ephb6	-1.25491	0.83831	-3.65003	0.002431	0.046539	-1.35896	1.286975003	1.803980737	1.34329276	-0.011397395	0.626441549	-0.019430984
ENSMUSG00000056679.13	Gpr173	-1.25446	0.150141	-2.80823	0.013413	0.126604	-2.77405	0.941839517	0.614946913	0.67010308	0.474029432	-1.139093197	-0.660977013
ENSMUSG00000017639.13	Rab11fip4	-1.25261	2.263021	-3.84672	0.001631	0.037353	-1.02749	3.133265588	3.533333147	2.09000633	1.787689911	1.493175018	1.540656848
ENSMUSG00000022537.17	Tmem44	-1.25075	2.310336	-5.55213	5.90E-05	0.008819	2.038029	2.989773994	3.13096206	2.67981823	1.852541056	1.799506258	1.409412315
ENSMUSG00000013921.14	Clip3	-1.25028	2.898968	-5.23013	0.000108	0.010484	1.48397	3.771305141	3.835276868	3.00754417	2.504617752	1.987439209	2.287623834
ENSMUSG00000044927.6	H1fx	-1.24962	1.915853	-4.05163	0.001079	0.030547	-0.62426	2.577876203	3.170763068	1.93638314	1.410835606	1.329055639	1.070206228
ENSMUSG00000034520.14	Gjc1	-1.24962	2.565363	-4.45554	0.000482	0.020456	0.085481	3.324843189	3.319818877	2.844354	2.795957527	1.493175018	1.614030034
ENSMUSG00000033618.6	Map3k13	-1.24839	2.014081	-4.59454	0.000366	0.01826	0.357202	2.526802018	3.004513725	2.35546747	1.719785847	1.55392905	0.923985488
ENSMUSG00000006445.3	Epha2	-1.24636	-0.29658	-2.69879	0.016694	0.143634	-2.95705	0.376242341	0.494652679	0.1045059	-0.954813866	-0.402127603	-1.397942607
ENSMUSG00000041117.8	Ccdc8	-1.24564	0.900074	-2.80571	0.013481	0.126608	-2.79271	1.158650906	2.274871471	1.13307505	1.17763643	-0.65366637	0.309876641
ENSMUSG00000097234.1	Gm26518	-1.24515	0.398529	-3.00161	0.009084	0.100476	-2.45985	0.941839517	0.614946913	1.34329276	0.554199781	-0.402127603	-0.660977013
ENSMUSG00000023912.9	Slc25a27	-1.24479	1.68723	-3.22102	0.005821	0.07959	-2.13109	1.999172692	2.120837843	2.57959078	1.974103035	1.396959703	0.052718802
ENSMUSG00000029765.12	Plxna4	-1.2438	2.239166	-3.74479	0.002006	0.042282	-1.21648	2.473853138	3.835276868	2.37951295	1.534570974	1.722000532	1.489582663
ENSMUSG00000022054.11	Nefm	-1.24375	3.654091	-6.46136	1.17E-05	0.004774	3.587123	4.252022405	4.674994296	3.91597693	3.145646315	3.158587352	2.777315838
ENSMUSG00000031028.13	Tub	-1.24341	1.265616	-3.85082	0.001618	0.037269	-0.98642	1.961204842	1.993458536	1.65047427	0.95945626	0.97638402	0.052718802
ENSMUSG00000042453.14	Reln	-1.24297	2.400245	-4.03529	0.001115	0.030999	-0.68707	2.743613407	3.486790561	2.82697193	1.754137352	2.485397668	1.104557733
ENSMUSG00000046743.6	Fat4	-1.23959	4.720134	-6.83584	6.18E-06	0.004774	4.149632	5.388811015	5.704106045	4.91803059	4.341925896	4.1512329	3.816700314
ENSMUSG00000036602.13	Alx1	-1.23755	1.553632	-4.07389	0.001031	0.030012	-0.58257	2.526802018	2.237877264	1.72743625	1.275483753	0.97638402	0.577809846
ENSMUSG00000050382.14	Kif7	-1.23581	1.102687	-4.01555	0.00116	0.03138	-0.71258	1.663123489	2.037179913	1.48301752	0.554199781	0.568726051	0.309876641
ENSMUSG00000093979.7	Gm2237	-1.23433	-0.01334	-2.61481	0.019727	0.155682	-3.0741	0.776780271	0.058553564	0.88691447	-0.748362989	0.163469573	-1.217370362

ENSMUSG00000028519.16	Dab1	-1.2342	0.087268	-2.90357	0.011073	0.11248	-2.6276	1.089938156	0.829071718	0.21553721	-0.262936162	-0.798056279	-0.549945701
ENSMUSG00000086968.8	4933431E20Ri	-1.23404	-0.19182	-2.32647	0.034686	0.199707	-3.49781	0.255948108	1.10037374	-0.14703287	0.202727411	-0.958520951	-1.604393485
ENSMUSG00000020875.9	Hoxb9	-1.23338	2.566282	-3.87889	0.001528	0.036222	-1.00188	3.339716465	3.732641955	2.47187697	2.563914936	1.77419517	1.51534576
ENSMUSG00000085581.1	Gm12786	-1.23188	0.302352	-2.39884	0.030149	0.18787	-3.41059	1.224239248	1.525679575	-0.01578833	0.389140535	-0.091787482	-1.217370362
ENSMUSG00000040649.15	Rimklb	-1.23186	3.38066	-5.14878	0.000126	0.011014	1.296007	4.120403437	3.859833972	3.88271448	3.468867727	2.805765249	2.146377909
ENSMUSG00000022367.7	Has2	-1.23181	0.706599	-3.19778	0.006102	0.081642	-2.12888	1.798475342	1.462943819	0.81820172	-0.407326071	0.380280962	0.187019893
ENSMUSG00000089774.2	Slc5a3	-1.23169	2.500142	-3.29624	0.004995	0.072879	-2.10279	3.45348534	3.337412937	2.4493344	3.065845604	1.429749638	1.265022405
ENSMUSG00000025577.7	Cbx2	-1.23163	2.44753	-4.54997	0.0004	0.018566	0.268262	3.199991702	3.533333147	2.49407271	2.139162282	1.61222769	1.706394053
ENSMUSG00000041272.11	Tox	-1.23155	0.759345	-3.1548	0.006659	0.085817	-2.2003	0.776780271	1.853733772	1.43792963	0.77101117	-0.188002798	-0.095379837
ENSMUSG00000044807.13	Zfp354c	-1.23138	1.901602	-3.74553	0.002003	0.042282	-1.18639	2.390597634	3.004513725	2.09000633	2.031246943	1.220802748	0.672446721
ENSMUSG00000069171.13	Nr2f1	-1.23066	3.785611	-5.01085	0.000163	0.012385	0.9897	4.328575908	4.695604576	4.11528574	3.597411848	3.520070246	2.45671803
ENSMUSG00000030352.14	Tspan9	-1.23062	1.687595	-4.63994	0.000335	0.017796	0.420319	2.390597634	2.571003565	2.03050532	0.836599512	1.062540664	1.234325608
ENSMUSG00000049907.8	Rasl11b	-1.23042	0.121119	-2.44291	0.027668	0.18028	-3.3356	0.124703574	1.397355478	0.58993273	0.630148634	-0.958520951	-1.056905689
ENSMUSG00000085246.1	Gm15893	-1.22981	0.269301	-2.70777	0.016398	0.142241	-2.93054	0.686582462	1.328642728	0.58993273	-0.011397395	0.239418426	-1.217370362
ENSMUSG00000020135.13	Apc2	-1.22921	4.335832	-6.62493	8.82E-06	0.004774	3.820046	4.884670994	5.395257012	4.58218323	3.955771213	3.465768861	3.73134041
ENSMUSG00000034799.16	Unc13a	-1.22914	2.255161	-3.17904	0.006339	0.083118	-2.28344	2.500570476	3.337412937	2.67981823	2.031246943	2.453862094	0.528056811
ENSMUSG00000024534.15	Sncaip	-1.22886	0.689938	-3.15081	0.006713	0.085977	-2.20788	1.347095996	1.853733772	0.81820172	0.099633918	-0.402127603	0.423087252
ENSMUSG00000029436.9	Mmp17	-1.22815	-0.27186	-2.82174	0.013054	0.124854	-2.77356	0.124703574	0.494652679	0.41484602	-0.954813866	-0.798056279	-0.91251578
ENSMUSG00000049804.9	Armcx4	-1.22802	3.909097	-4.99377	0.000169	0.012548	0.941295	4.723907998	4.850673973	3.95650474	3.895493201	3.108834316	2.919168471
ENSMUSG00000032012.7	Nectin1	-1.22714	2.472093	-4.00357	0.001188	0.031763	-0.75434	2.830418235	3.664577681	2.75527046	2.54441876	1.182834898	1.855038134
ENSMUSG00000040258.6	Nxph4	-1.22646	0.514888	-2.68395	0.017195	0.146277	-2.97351	1.089938156	2.120837843	0.31863071	-0.262936162	-0.001589673	-0.175550186
ENSMUSG00000037709.13	Fam13a	-1.22471	-0.01279	-2.20537	0.043743	0.214017	-3.67856	1.347095996	0.725978225	-0.29142278	0.389140535	-1.586552174	-0.660977013
ENSMUSG00000032291.8	Crabp1	-1.22377	3.011026	-4.98482	0.000172	0.012592	1.030299	3.397722069	4.242553751	3.24855227	2.675663294	2.371868722	2.129795025
ENSMUSG00000034818.16	Celf5	-1.22298	0.084446	-2.64928	0.018423	0.151573	-3.02107	0.487273654	1.015484842	0.50504383	-0.011397395	-0.091787482	-1.397942607
ENSMUSG00000031342.17	Gpm6b	-1.22071	2.997573	-6.66395	8.26E-06	0.004774	3.913806	3.558934639	3.758993282	3.48855571	2.69347457	2.153688552	2.331791418
ENSMUSG00000048078.16	Tenm4	-1.2199	3.806501	-4.74874	0.000271	0.015809	0.489198	4.654227089	4.850673973	3.74142048	3.56874809	3.225479235	2.798454606
ENSMUSG00000041741.10	Pde3a	-1.21925	1.083601	-3.93655	0.00136	0.034272	-0.84952	1.615029201	1.803980737	1.65047427	0.77101117	0.083299224	0.577809846
ENSMUSG00000014303.13	Glis2	-1.21863	2.514309	-3.95319	0.001315	0.033365	-0.85477	3.06330303	3.593142542	2.66032206	2.484297768	2.113887544	1.170900228
ENSMUSG00000062312.5	Erbb2	-1.21838	2.048786	-4.01983	0.00115	0.03138	-0.68907	2.809201749	2.936875008	2.17489523	2.002957898	1.523871816	0.844913917
ENSMUSG00000048385.8	Scrt1	-1.21766	2.140492	-3.888	0.0015	0.035955	-0.93911	2.473853138	3.517985183	2.426434	1.017171757	1.493175018	1.914335318
ENSMUSG00000078878.9	Gm14305	-1.21765	0.075808	-2.63055	0.019121	0.153734	-3.04978	0.776780271	0.219018236	0.95250281	0.202727411	-1.345544075	-0.350636892
ENSMUSG00000003410.7	Elavl3	-1.2176	3.195304	-4.23495	0.000747	0.025405	-0.40718	3.726739589	4.58211552	3.15413667	2.876127876	2.500910667	2.331791418
ENSMUSG00000027220.2	Syt13	-1.21759	0.557368	-2.97813	0.009526	0.103577	-2.49572	1.158650906	1.699011178	0.74605193	-0.567790743	0.568726051	-0.260439084
ENSMUSG00000048251.15	Bcl11b	-1.21716	1.8871	-3.46376	0.003551	0.058821	-1.70486	2.577876203	3.228478566	1.76443046	1.611532956	0.786906222	1.35337828
ENSMUSG00000104145.1	D130019J16Ri	-1.21654	0.52793	-2.9115	0.010897	0.111729	-2.60495	1.089938156	1.328642728	0.95250281	0.70229842	-0.958520951	0.052718802
ENSMUSG00000037071.2	Scd1	-1.21611	2.194396	-3.83858	0.001658	0.03778	-1.03675	2.871937504	3.371970159	2.2021167	2.086213402	1.020105398	1.614030034
ENSMUSG00000027223.15	Mapk8ip1	-1.21294	2.325373	-3.91761	0.001413	0.034545	-0.90094	3.027006901	3.607715344	2.22883404	1.852541056	1.799506258	1.436633784
ENSMUSG00000045179.9	Sox3	-1.21263	1.191413	-4.10912	0.000961	0.028977	-0.5432	1.840910608	1.993458536	1.56917417	0.77101117	0.445869304	0.528056811
ENSMUSG00000025001.11	Hells	-1.20962	3.20538	-5.70697	4.44E-05	0.008335	2.31516	3.927143006	3.678449855	3.73211274	3.245613223	2.302744362	2.346218488
ENSMUSG00000061615.2	Hist1h2ab	-1.20913	2.189177	-3.23522	0.005655	0.078474	-2.16808	2.698170436	3.388943238	2.25506558	2.620870821	0.735375921	1.436633784
ENSMUSG00000097242.1	Gm16907	-1.20889	0.39057	-3.16656	0.006502	0.084626	-2.19262	1.089938156	0.614946913	1.18857017	0.202727411	-0.402127603	-0.350636892
ENSMUSG00000007207.10	Stx1a	-1.20886	0.670002	-3.5161	0.003192	0.05514	-1.59719	1.089938156	1.256492942	1.43792963	0.099633918	0.311568212	-0.175550186
ENSMUSG00000021318.15	Gli3	-1.20866	3.555559	-4.80914	0.000241	0.014998	0.638862	4.477219989	4.175661867	3.74142048	3.643954434	2.878828711	2.416265986
ENSMUSG00000049916.9	2610318N02R	-1.20567	0.698489	-2.59501	0.020516	0.158008	-3.12748	1.513745865	1.462943819	0.81820172	0.77101117	0.681936662	-1.056905689
ENSMUSG00000018427.7	Ypel2	-1.2052	0.974001	-3.25007	0.005487	0.076935	-2.03794	1.286975003	1.643516065	1.80049971	0.630148634	-0.402127603	0.884991356
ENSMUSG00000042078.14	Svop	-1.20422	0.421597	-2.8352	0.012706	0.122484	-2.72843	1.089938156	1.462943819	0.67010308	-0.567790743	-0.65366637	0.528056811
ENSMUSG00000038765.13	Lmx1b	-1.20349	0.382873	-3.01459	0.008849	0.099056	-2.43929	0.861669169	0.494652679	1.48301752	0.099633918	-0.29109629	-0.350636892
ENSMUSG00000068270.15	Shroom4	-1.2026	1.375413	-3.23397	0.005669	0.078512	-2.08127	1.961204842	1.699011178	2.06056255	1.852541056	0.626441549	0.052718802
ENSMUSG00000029121.15	Crmp1	-1.20175	3.840663	-4.63679	0.000337	0.017814	0.268891	4.283132937	5.148510261	3.89944156	3.625517186	3.187635884	2.899737941
ENSMUSG00000026675.12	Hsd17b7	-1.20128	2.003655	-4.32287	0.000627	0.023298	-0.13218	2.418886679	3.170763068	2.28082867	1.573565106	1.062540664	1.51534576
ENSMUSG00000026915.16	Strbp	-1.2002	3.718887	-5.7351	4.22E-05	0.008091	2.326451	4.290806865	4.521837508	4.07903521	3.766811182	2.866905544	2.787923938
ENSMUSG00000032135.14	Mcam	-1.20016	0.673427	-3.20615	0.005999	0.080764	-2.11556	1.404811494	1.643516065	0.81820172	-0.011397395	0.445869304	-0.260439084
ENSMUSG00000073805.4	Fam196a	-1.2	0.125576	-2.81402	0.013258	0.125817	-2.7656	0.776780271	0.925287033	0.41484602	0.202727411	-0.65366637	-0.91251578
ENSMUSG00000070643.11	Sox13	-1.19877	1.773711	-4.27327	0.000692	0.024199	-0.22178	2.446631669	2.380481659	2.2021167	1.719785847	1.220802748	0.672446721
ENSMUSG00000027298.17	Tyro3	-1.19835	1.32898	-3.63167	0.002524	0.047898	-1.37164	1.922210711	2.199909414	1.61039683	1.453270872	0.311568212	0.476526511
ENSMUSG00000027574.15	Nkain4	-1.19803	-0.02549	-2.4793	0.025767	0.17605	-3.27756	0.255948108	1.10037374	0.31863071	0.298942726	-1.345544075	-0.781271247
ENSMUSG00000010175.13	Prox1	-1.1973	1.584516	-3.8782	0.001531	0.036222	-0.93227	2.107425583	2.816580774	1.72743625	0.836599512	1.020105398	0.998947545
ENSMUSG00000032101.6	Ddx25	-1.1965	-0.04715	-2.67946	0.01735	0.1467	-2.97678	0.590367147	1.015484842	0.1045059	-0.407326071	-1.139093197	-0.446852208
ENSMUSG00000087620.7	5330434G04R	-1.19619	0.68989	-3.01003	0.008931	0.099585	-2.44308	1.089938156	1.397355478	1.29353973	0.95945626	-0.65366637	0.052718802
ENSMUSG00000060510.13	Zfp266	-1.19576	4.052442	-5.45001	7.13E-05	0.009244	1.774496	4.811870936	4.722634782	4.34634608	4.17446915	3.108834316	3.150494017
ENSMUSG00000032064.17	Dixdc1	-1.19477	1.878957	-3.33506	0.004615	0.06951	-1.9419	2.473853138	3.265711472	1.72743625	1.787689911	1.020105398	0.998947545
ENSMUSG00000062184.11	Hs6st2	-1.19469	0.451618	-2.79574	0.013753	0.128071	-2.79227	0.590367147	1.699011178	0.81820172	0.554199781	-0.402127603	-0.549945701
ENSMUSG00000042873.11	Lhfpl4	-1.19406	1.591883	-3.75743	0.001955	0.04175	-1.15009	2.0361669	2.346130154	2.14715024	1.611532956	0.239418426	1.170900228
ENSMUSG00000062588.4	Gm6104	-1.19348	0.873637	-2.85544	0.012199	0.119575	-2.70778	1.615029201	1.585800567	1.18857017	1.07266687	-0.798056279	0.577809846
ENSMUSG00000037892.13	Pcdh18	-1.19345	3.460378	-5.6188	5.22E-05	0.008758	2.141937	4.058815639	4.35046909	3.72274456	3.394594965	2.688725828	2.546915839
ENSMUSG00000029546.12	Uncx	-1.19335	1.61958	-2.71684	0.016104	0.140566	-3.02486	2.30224176	2.816580774	1.5267389	1.126106129	1.965243463	-0.019430984
ENSMUSG00000086324.8	Gm15564	-1.19332	9.113104	-4.33675	0.00061	0.022923	-0.43477	9.57777819	10.00098182	9.55777853	8.783148438	9.148080476	7.61085948
ENSMUSG00000029231.15	Pdgfra	-1.19255	3.998645	-6.31731	1.50E-05	0.005308	3.32112	4.671965366	4.618549149	4.4293165	4.006524513	3.09867445	3.166842012
ENSMUSG00000026185.8	Igfbp5	-1.19216	4.17502	-6.23126	1.74E-05	0.005836	3.159164	4.779510064	5.087434684	4.41775175	3.977741613	3.565041005	3.222643803
ENSMUSG00000070509.15	Rgma	-1.19128	2.180952	-3.7407	0.002022	0.042285	-1.21907	2.627203915	3.47093661	2.25506558	2.139162282	1.103763327	1.489582663
ENSMUSG00000078435.5	AU041133	-1.18912	0.009085	-2.9084	0.010966	0.112023	-2.62394	0.776780271	0.494652679	0.50504383	-0.262936162	-0.798056279	-0.660977013
ENSMUSG00000023886.9	Smoc2	-1.18743	0.217141	-3.11382	0.007237	0.090084	-2.28858	0.686582462	1.015484842	0.74605193	-0.407326071	-0.29109629	-0.446852208
ENSMUSG00000036523.16	Greb1	-1.18732	2.242572	-3.27028	0.005266	0.075492	-2.11062	2.912295242	3.422301835	2.14715024	2.139162282	2.030831804	0.803691254
ENSMUSG00000030555.15	Ttc23	-1.18569	1.577354	-3.51543	0.003197	0.05514	-1.58744	2.107425583	2.478885363	1.83568914	1.648527164	1.143840766	0.249755649
ENSMUSG00000059674.6	Cdh24	-1.18556	1.753647	-3.23043	0.00571	0.078674	-2.12086	2.446631669	2.629302206	1.87004065	2.263609653	0.508605059	0.803691254
ENSMUSG00000014837.14	4931428F04Ri	-1.18475	1.784864	-4.02048	0.001148	0.03138	-0.67793	2.698170436	2.120837843	2.2021167	1.787689911	1.182834898	0.71753461
ENSMUSG00000030411.15	Nova2	-1.18397	2.492522	-4.54445	0.000404	0.018666	0.255334	2.912295242	3.692189912	2.67981823	2.164925378	1.799506258	1.706394053
ENSMUSG00000006362.15	Cbfa2t3	-1.18363	0.572613	-2.76996	0.014482	0.132398	-2.83655	0.376242341	1.752450437	1.43792963	-0.262936162	-0.29109629	0.423087252
ENSMUSG00000044548.11	Dact1	-1.18361	1.445861	-3.53513	0.003071	0.054085	-1.54586	2.240180792	2.478885363	1.43792963	1.227389465	0.445869304	0.844913917
ENSMUSG00000032482.9	Cspg5	-1.18322	-0.18271	-2.71632	0.016121	0.140566	-2.92558	0.776780271	0.219018236	0.21553721	-0.567790743	-0.958520951	-0.781271247
ENSMUSG00000053441.4	Adamts19	-1.18229	0.492472	-3.02419	0.008679	0.098507	-2.42094	0.941839517	1.585800567	0.74605193	0.298942726	-0.522421837	-0.095379837
ENSMUSG00000038319.14	Kcnh2	-1.18228	1.859974	-3.82359	0.001709	0.038552	-1.04003	2.390597634	3.04790632	1.96844435	1.227389465	1.640516735	0.884991356
ENSMUSG00000039476.13	Prrx2	-1.18107	0.104397	-2.41784	0.029055	0.184844	-3.37263	0.686582462	1.10037374	0.31863071	0.202727411	-1.586552174	-0.095379837
ENSMUSG00000028524.21	Sgip1	-1.18079	0.411433	-3.12208	0.007117	0.089222	-2.26382	0.776780271	1.10037374	1.07535956	0.298942726	-0.522421837	-0.260439084
ENSMUSG00000068115.13	Ninl	-1.1791	1.855747	-3.54737	0.002996	0.053527	-1.54861	1.798475342	3.090031796	2.40316422	1.684596418	1.396959703	0.761255988
ENSMUSG00000097156.7	Gm3764	-1.17857	2.03851	-3.68766	0.002252	0.044897	-1.3048	2.871937504	2.540946331	2.426434	2.139162282	0.568726051	1.683851484
ENSMUSG00000062591.5	Tubb4a	-1.17766	1.334445	-3.93412	0.001367	0.034312	-0.83721	2.072236155	2.07961518	1.61039683	1.126106129	0.445869304	0.672446721
ENSMUSG00000042589.18	Cux2	-1.17738	1.967682	-2.69143	0.016941	0.145166	-3.12786	2.830418235	3.190259244	1.61039683	2.058991933	1.748432074	0.367592139
ENSMUSG00000022197.15	Pdzd2	-1.16796	0.734863	-3.24318	0.005564	0.077675	-2.05164	1.347095996	1.015484842	1.48301752	0.77101117	-0.188002798	-0.019430984
ENSMUSG00000074274.7	D930028M14F	-1.16748	0.40112	-2.75411	0.014949	0.134718	-2.85842	0.776780271	1.699011178	0.50504383	0.298942726	-0.522421837	-0.350636892
ENSMUSG00000038738.15	Shank1	-1.16704	1.94262	-3.96616	0.001281	0.03302	-0.78241	2.240180792	3.13096206	2.22883404	1.453270872	1.640516735	0.961953338
ENSMUSG00000088246.1	Gm25911	-1.16679	7.486755	-4.01675	0.001157	0.03138	-1.11131	7.955482842	8.389733972	7.87189586	7.156522471	7.604394843	5.942500975
ENSMUSG00000028389.10	Zfp37	-1.16677	0.908571	-3.56263	0.002904	0.052591	-1.50256	1.286975003	1.853733772	1.34329276	0.554199781	0.163469573	0.249755649
ENSMUSG00000020866.17	Cacna1g	-1.16631	3.410379	-4.56704	0.000387	0.018464	0.197888	3.83567396	4.442765937	3.66522086	3.171294903	3.168335328	2.178982574
ENSMUSG00000001985.9	Grik3	-1.1655	0.981959	-3.409	0.00397	0.062828	-1.76392	1.798475342	1.853733772	1.07535956	0.474029432	0.568726051	0.121431552
ENSMUSG00000031502.9	Col4a1	-1.16462	4.117989	-4.64299	0.000333	0.017796	0.24043	4.401271136	5.46894256	4.2518126	3.856499069	3.289406562	3.440000635
ENSMUSG00000031425.15	Plp1	-1.16363	0.950028	-2.74359	0.015266	0.13661	-2.90085	1.404811494	2.199909414	1.01523856	1.126106129	-0.522421837	0.476526511
ENSMUSG00000106099.1	Gm42664	-1.16313	2.363417	-5.60998	5.31E-05	0.008781	2.140451	2.989773994	2.913605228	2.89527794	1.944659258	1.896530713	1.540656848
ENSMUSG00000041220.10	Elovl6	-1.16226	2.614692	-4.64809	0.00033	0.017703	0.43876	3.216201929	3.438696273	2.91186082	2.602134258	1.493175018	2.026083675
ENSMUSG00000102440.1	Pcdhga9	-1.15881	0.624921	-3.32462	0.004714	0.070371	-1.91949	1.017788371	1.256492942	1.29353973	0.202727411	0.239418426	-0.260439084
ENSMUSG00000062661.6	Ncs1	-1.15855	2.409834	-4.12559	0.00093	0.028613	-0.51859	2.892257488	3.593142542	2.53746531	2.139162282	1.363407143	1.933571536
ENSMUSG00000061578.8	Ksr2	-1.15765	1.061068	-3.28464	0.005114	0.073952	-1.97915	1.286975003	2.237877264	1.39138705	0.77101117	0.626441549	0.052718802
ENSMUSG00000020185.16	E2f7	-1.15575	1.795656	-3.44972	0.003654	0.059903	-1.72336	2.271544963	2.765506589	1.99980852	2.139162282	0.836659257	0.761255988
ENSMUSG00000042680.7	Garem1	-1.15558	0.769406	-3.39927	0.00405	0.063659	-1.78711	1.224239248	1.525679575	1.29353973	0.099633918	0.568726051	-0.095379837
ENSMUSG00000033762.7	Recql4	-1.15531	0.78305	-2.67871	0.017376	0.146735	-2.99694	1.286975003	1.256492942	1.34329276	1.453270872	-0.091787482	-0.549945701
ENSMUSG00000064796.1	Terc	-1.15531	0.690229	-2.54401	0.02269	0.166062	-3.20941	1.017788371	1.803980737	0.88691447	0.899335267	0.445869304	-0.91251578
ENSMUSG00000025469.10	Msx3	-1.15426	0.519246	-2.33395	0.034189	0.198521	-3.52637	0.124703574	1.853733772	1.24200942	0.298942726	0.508605059	-0.91251578
ENSMUSG00000056481.6	Cd248	-1.15149	0.833184	-3.0709	0.007895	0.093838	-2.34246	1.286975003	1.993458536	0.95250281	0.630148634	0.311568212	-0.175550186
ENSMUSG00000021806.3	Nid2	-1.15114	2.208908	-4.14525	0.000894	0.028111	-0.4667	2.851327224	3.13096206	2.35546747	1.914602024	1.896530713	1.104557733
ENSMUSG00000066057.6	Gm1976	-1.15076	0.524316	-2.78147	0.014152	0.130303	-2.81677	0.941839517	0.925287033	1.29353973	0.899335267	-0.65366637	-0.260439084
ENSMUSG00000034926.3	Dhcr24	-1.1499	3.452036	-4.91784	0.000195	0.013581	0.857659	4.154450918	4.401543275	3.52134564	3.361554703	2.561346521	2.711975085
ENSMUSG00000030283.7	St8sia1	-1.14981	1.581527	-2.9671	0.009741	0.104827	-2.57637	2.0361669	3.04790632	1.43792963	1.573565106	0.508605059	0.884991356
ENSMUSG00000029094.11	Afap1	-1.14922	3.622653	-5.07765	0.000144	0.011551	1.136146	4.252022405	4.762253612	3.65540657	3.038233373	2.902383439	3.125619349
ENSMUSG00000031748.15	Gnao1	-1.14911	2.073964	-3.96108	0.001295	0.033088	-0.79932	2.271544963	3.170763068	2.55868179	1.17763643	2.030831804	1.234325608
ENSMUSG00000021359.15	Tfap2a	-1.14739	2.258336	-4.52655	0.000419	0.018982	0.232882	2.765809153	3.283973678	2.49407271	1.883905227	1.329055639	1.79319888
ENSMUSG00000047139.8	Cd24a	-1.14737	3.709027	-4.43905	0.000498	0.020613	-0.09415	4.267661528	4.939757516	3.66522086	3.519672098	2.914018139	2.94783223

ENSMUSG00000041147.10	Brca2	-1.14724	2.693387	-4.23382	0.000749	0.025405	-0.34391	3.06330303	3.319818877	3.2998535	3.010082333	1.61222769	1.855038134
ENSMUSG00000107947.1	Gm44438	-1.14723	-0.04125	-2.25977	0.039431	0.208097	-3.59912	0.590367147	1.015484842	-0.01578833	0.298942726	-1.586552174	-0.549945701
ENSMUSG00000005360.14	Slc1a3	-1.1463	0.954573	-3.44214	0.003711	0.060365	-1.70738	1.565276166	1.752450437	1.29353973	0.630148634	-0.091787482	0.577809846
ENSMUSG00000051817.8	Sox12	-1.1453	3.010423	-4.59777	0.000364	0.01826	0.309086	3.546167343	4.195092397	3.06798002	2.639367164	2.21140405	2.402525929
ENSMUSG00000035551.6	Igfbpl1	-1.14411	2.991191	-4.21417	0.000779	0.02597	-0.41785	3.546167343	4.155966066	3.08270153	2.54441876	1.872879445	2.745015347
ENSMUSG00000031302.16	Nlgn3	-1.14327	0.089007	-2.55815	0.022066	0.163863	-3.16033	1.089938156	0.725978225	0.21553721	-0.262936162	-1.139093197	-0.095379837
ENSMUSG00000028763.17	Hspg2	-1.14148	4.061674	-4.88769	0.000207	0.014101	0.718777	4.456900005	5.343955783	4.13660674	3.56874809	3.557642552	3.306191594
ENSMUSG00000062444.14	Ap3b2	-1.14018	1.485534	-3.09151	0.007572	0.091786	-2.34424	1.798475342	2.510249534	1.80049971	1.227389465	1.523871816	0.052718802
ENSMUSG00000033768.16	Nrxn2	-1.13828	1.915547	-4.50975	0.000433	0.019425	0.203889	2.552565114	2.765506589	2.17489523	1.275483753	1.55392905	1.170900228
ENSMUSG00000025658.16	Cnksr2	-1.13725	1.404752	-3.85451	0.001606	0.037213	-0.97557	1.798475342	2.120837843	1.96844435	1.275483753	0.380280962	0.884991356
ENSMUSG00000026039.9	Sgo2a	-1.13685	0.568755	-2.65112	0.018355	0.151219	-3.02926	1.089938156	1.015484842	1.13307505	1.126106129	-0.29109629	-0.660977013
ENSMUSG00000102336.1	Gm37233	-1.13559	0.335577	-2.83657	0.012671	0.122354	-2.72645	0.686582462	1.462943819	0.67010308	-0.567790743	-0.29109629	0.052718802
ENSMUSG00000044452.10	Zfp507	-1.13557	2.142309	-4.23304	0.00075	0.025405	-0.30095	2.698170436	2.913605228	2.4493344	2.139162282	1.583372827	1.070206228
ENSMUSG00000035413.8	Tmem98	-1.13524	0.891317	-3.08704	0.007641	0.092209	-2.31625	1.404811494	1.256492942	1.56917417	0.95945626	0.508605059	-0.350636892
ENSMUSG00000037736.18	Limch1	-1.13499	2.540083	-3.55878	0.002927	0.052791	-1.60997	2.951554672	3.607715344	2.71803755	2.876127876	1.29386621	1.79319888
ENSMUSG00000057880.12	Abat	-1.13494	1.963759	-3.46431	0.003547	0.058821	-1.71265	2.500570476	3.110642075	1.99980852	2.002957898	0.786906222	1.381667324
ENSMUSG00000030386.17	Zfp606	-1.13467	2.455357	-4.47838	0.00046	0.020062	0.134339	2.932058497	2.95977541	3.05310675	2.602134258	1.748432074	1.436633784
ENSMUSG00000024011.16	Pi16	-1.13449	0.029783	-2.68463	0.017172	0.146277	-2.96739	0.861669169	0.219018236	0.67010308	-0.262936162	-0.958520951	-0.350636892
ENSMUSG00000026211.17	Obsl1	-1.13396	2.884538	-3.9122	0.001429	0.034672	-0.98392	3.30981498	3.976715272	3.03807854	2.69347457	2.675115396	1.614030034
ENSMUSG00000038331.15	Satb2	-1.13361	1.165268	-3.56367	0.002898	0.052591	-1.49232	1.615029201	1.993458536	1.56917417	1.07266687	0.163469573	0.577809846
ENSMUSG00000022623.15	Shank3	-1.13284	1.190702	-3.17182	0.006433	0.084053	-2.18025	1.347095996	2.380481659	1.5267389	1.227389465	0.239418426	0.423087252
ENSMUSG00000053617.11	Sh3pxd2a	-1.13272	3.230394	-5.46337	6.96E-05	0.009244	1.881639	3.749194439	4.155966066	3.477458	3.065845604	2.437831985	2.496066697
ENSMUSG00000038236.8	Hoxa7	-1.13242	0.021051	-2.54458	0.022664	0.166012	-3.18023	1.017788371	0.614946913	0.1045059	-0.262936162	-0.29109629	-1.056905689
ENSMUSG00000025558.15	Dock9	-1.13125	1.226601	-3.32905	0.004672	0.070051	-1.90536	1.961204842	1.993458536	1.39138705	1.322026339	0.163469573	0.528056811
ENSMUSG00000030268.17	Bcat1	-1.13113	2.196722	-4.01561	0.00116	0.03138	-0.70696	2.651249389	3.302007601	2.33101443	2.11293074	1.429749638	1.35337828
ENSMUSG00000030350.8	Prmt8	-1.13071	0.844231	-2.97536	0.009579	0.103779	-2.50451	1.347095996	1.525679575	1.24200942	1.017171757	0.380280962	-0.446852208
ENSMUSG00000022203.6	Efs	-1.13057	1.836855	-3.99279	0.001214	0.032203	-0.72962	2.446631669	2.712557709	2.03050532	1.534570974	1.493175018	0.803691254
ENSMUSG00000042258.13	Isl1	-1.12853	1.883613	-3.35887	0.004397	0.067181	-1.89913	2.361742772	3.170763068	1.83568914	1.852541056	0.97638402	1.104557733
ENSMUSG00000048814.10	Lonrf2	-1.12761	0.630509	-2.92983	0.010502	0.109327	-2.57564	0.861669169	1.901828061	0.88691447	-0.011397395	0.239418426	-0.095379837
ENSMUSG00000086296.2	D030055H07R	-1.1269	0.079691	-2.74947	0.015088	0.135634	-2.86689	0.861669169	0.829071718	0.31863071	-0.748362989	-0.522421837	-0.260439084
ENSMUSG00000032744.4	Heyl	-1.12576	0.978102	-2.71312	0.016224	0.141185	-2.95426	1.286975003	2.414034219	1.01523856	0.77101117	-0.29109629	0.672446721
ENSMUSG00000031523.16	Dlc1	-1.12531	2.583297	-4.57981	0.000377	0.018298	0.314827	3.30981498	3.578421037	2.6405588	1.944659258	1.896530713	2.129795025
ENSMUSG00000100252.6	Mir124-2hg	-1.12509	3.10717	-5.29024	9.61E-05	0.010239	1.57901	3.946705272	3.84760767	3.22220094	2.779374643	2.500910667	2.346218488
ENSMUSG00000103793.1	Pcdhga6	-1.12245	-0.36522	-2.14655	0.048898	0.221375	-3.7443	-0.360723253	0.494652679	0.31863071	0.099633918	-1.139093197	-1.604393485
ENSMUSG00000003273.13	Car11	-1.12227	0.037341	-2.76344	0.014672	0.133118	-2.84632	0.590367147	0.725978225	0.50504383	-0.748362989	-0.188002798	-0.660977013
ENSMUSG00000018417.14	Myo1b	-1.12201	3.875419	-7.15193	3.67E-06	0.004108	4.702845	4.267661528	4.681897157	4.34023296	3.587920399	3.216111053	3.15869117
ENSMUSG00000051586.14	Mical3	-1.12132	3.032874	-5.78771	3.84E-05	0.008043	2.461776	3.57158994	3.678449855	3.477458	2.812351965	2.561346521	2.096046233
ENSMUSG00000030796.15	Tead2	-1.12061	3.543493	-4.5302	0.000416	0.018974	0.10713	4.187713372	4.60408592	3.52134564	3.426895457	2.842759456	2.678160372
ENSMUSG00000095427.1	Rps2-ps6	-1.12026	1.274235	-3.23566	0.00565	0.078474	-2.07216	1.565276166	2.237877264	1.6894684	0.70229842	1.329055639	0.121431552
ENSMUSG00000002341.8	Ncan	-1.11839	3.581361	-3.84855	0.001625	0.037299	-1.22917	3.846128318	4.632868821	3.91597693	3.783538268	2.192420947	3.117231563
ENSMUSG00000042750.7	Bex2	-1.11821	0.936835	-2.77563	0.014318	0.131331	-2.84602	1.709666075	2.199909414	0.67010308	0.474029432	0.445869304	0.121431552
ENSMUSG00000017386.10	Traf4	-1.11755	2.62162	-4.385	0.000554	0.021658	-0.05141	3.183597264	3.622142414	2.75527046	2.484297768	1.640516735	2.043894951
ENSMUSG00000042978.10	Sbk1	-1.1167	4.155177	-4.82263	0.000235	0.014899	0.581318	4.826057614	5.263412356	4.10089465	3.848572153	3.324703614	3.5869505
ENSMUSG00000030592.16	Ryr1	-1.11629	1.183456	-3.77808	0.001875	0.040638	-1.11688	1.615029201	1.993458536	1.61039683	0.836599512	0.568726051	0.476526511
ENSMUSG00000027546.15	Atp9a	-1.11582	2.169031	-5.092	0.00014	0.011551	1.2406	2.674900657	2.765506589	2.69905445	1.852541056	1.640516735	1.381667324
ENSMUSG00000015599.8	Ttbk1	-1.11555	0.90059	-2.23301	0.041499	0.2115	-3.72509	1.999172692	2.120837843	0.50504383	-0.748362989	1.103763327	0.423087252
ENSMUSG00000091383.1	Hist1h2al	-1.11325	1.518613	-3.62029	0.002583	0.048607	-1.39552	2.175329648	2.274871471	1.72743625	1.367114228	1.143840766	0.423087252
ENSMUSG00000102918.1	Pcdhgc3	-1.1128	1.346511	-3.8341	0.001673	0.03806	-1.01302	1.798475342	2.310940726	1.65047427	0.77101117	0.786906222	0.761255988
ENSMUSG00000041842.15	Fhdc1	-1.11247	0.833434	-2.69294	0.01689	0.144822	-2.97697	1.347095996	1.699011178	1.01523856	1.17763643	0.311568212	-0.549945701
ENSMUSG00000028370.7	Pappa	-1.1121	0.384687	-2.34534	0.033445	0.196453	-3.49809	0.686582462	1.397355478	0.67010308	0.202727411	0.568726051	-1.217370362
ENSMUSG00000042439.12	Zfp532	-1.11186	3.701406	-5.32079	9.08E-05	0.009985	1.576487	4.490609366	4.35046909	3.89944156	3.383665405	3.417540581	2.666710351
ENSMUSG00000031949.17	Adat1	-1.1114	0.696078	-2.96684	0.009746	0.104827	-2.51523	1.286975003	1.10037374	1.24200942	0.899335267	-0.001589673	-0.350636892
ENSMUSG00000021699.17	Pde4d	-1.11039	1.597431	-3.18408	0.006275	0.082773	-2.18872	2.072236155	2.765506589	1.65047427	1.611532956	0.380280962	1.104557733
ENSMUSG00000048960.13	Prex2	-1.10917	1.546762	-3.7863	0.001844	0.040147	-1.09722	2.361742772	2.380481659	1.61039683	1.07266687	0.931296131	0.923985488
ENSMUSG00000052957.7	Gas1	-1.10872	3.593525	-5.32585	8.99E-05	0.009985	1.597968	4.103073559	4.434615096	3.86579117	3.606841261	2.741918767	2.808908963
ENSMUSG00000029673.17	Auts2	-1.1086	4.518853	-4.84501	0.000225	0.014573	0.580084	4.996828752	5.456920032	4.73444531	4.324885993	4.264628989	3.335411733
ENSMUSG00000042155.3	Klhl23	-1.10764	1.269264	-3.12054	0.007139	0.089222	-2.27528	1.615029201	1.752450437	1.93638314	1.494493535	0.836659257	-0.019430984
ENSMUSG00000028289.12	Epha7	-1.10721	3.332451	-3.87916	0.001528	0.036222	-1.12459	3.927143006	3.79763921	3.7870792	3.479172736	3.108834316	1.894839142
ENSMUSG00000015217.11	Hmgb3	-1.10692	2.863846	-4.4788	0.00046	0.020062	0.100672	3.657198655	3.63642664	2.97634955	2.71106863	1.799506258	2.402525929
ENSMUSG00000020646.16	Mboat2	-1.10631	1.413258	-3.53747	0.003057	0.05397	-1.54087	2.175329648	2.237877264	1.48301752	1.275483753	0.681936662	0.625904135
ENSMUSG00000028487.18	Bnc2	-1.10459	1.810759	-3.56295	0.002902	0.052591	-1.51681	2.473853138	2.414034219	2.09000633	2.002957898	1.257796956	0.625904135
ENSMUSG00000009828.15	Ick	-1.10449	2.439664	-4.60967	0.000356	0.018057	0.377696	2.830418235	3.265711472	2.82697193	2.400028851	1.77419517	1.540656848
ENSMUSG00000068101.10	Cenpm	-1.10389	-0.14897	-2.16699	0.047046	0.219041	-3.72551	0.255948108	1.10037374	-0.01578833	-1.485328583	0.163469573	-0.91251578
ENSMUSG00000032181.6	Scg3	-1.10271	1.247411	-3.26164	0.005359	0.07594	-2.02485	1.754753965	2.414034219	1.34329276	0.630148634	0.380280962	0.961953338
ENSMUSG00000052544.9	St6galnac3	-1.10265	0.560305	-3.00037	0.009107	0.100499	-2.45908	1.158650906	1.397355478	0.81820172	0.202727411	-0.402127603	0.187019893
ENSMUSG00000050288.6	Fzd2	-1.10253	3.068634	-4.50992	0.000433	0.019425	0.135452	3.771305141	3.96544416	3.15413667	2.891640875	2.009298643	2.619979301
ENSMUSG00000025020.11	Slit1	-1.10202	2.155236	-3.10724	0.007334	0.090456	-2.40125	3.045269107	3.388943238	1.80049971	1.494493535	1.848833971	1.35337828
ENSMUSG00000060257.2	Scrt2	-1.09859	2.417143	-4.17615	0.00084	0.027264	-0.42471	2.830418235	3.607715344	2.53746531	1.944659258	1.668261725	1.914335318
ENSMUSG00000028469.15	Npr2	-1.0985	2.986223	-3.92612	0.001389	0.034458	-0.97345	3.216201929	4.074378531	3.26154961	2.981371036	2.590640828	1.79319888
ENSMUSG00000033577.18	Myo6	-1.0977	1.316367	-3.13154	0.006981	0.087881	-2.25864	1.754753965	2.571003565	1.34329276	0.70229842	1.103763327	0.423087252
ENSMUSG00000008999.7	Bmp7	-1.09719	0.591354	-2.78126	0.014158	0.130303	-2.81885	1.347095996	1.643516065	0.50504383	-0.011397395	0.239418426	-0.175550186
ENSMUSG00000050069.3	Grem2	-1.09687	0.819707	-3.23247	0.005687	0.07859	-2.06831	1.089938156	1.803980737	1.24200942	0.389140535	0.083299224	0.309876641
ENSMUSG00000008307.11	1700109H08R	-1.09455	0.44859	-2.21983	0.042555	0.212347	-3.69392	1.017788371	0.058553564	1.6894684	0.899335267	-0.798056279	-0.175550186
ENSMUSG00000035067.9	Xkr6	-1.09428	1.141235	-3.28988	0.00506	0.073404	-1.97174	1.882133271	1.643516065	1.48301752	1.227389465	0.083299224	0.528056811
ENSMUSG00000007827.10	Ankrd26	-1.09393	2.36609	-4.5921	0.000368	0.01826	0.348671	2.526802018	3.209495462	2.9604956	2.190236466	1.695483194	1.614030034
ENSMUSG00000036667.14	Tcaf1	-1.09393	3.970847	-5.44756	7.16E-05	0.009244	1.778591	4.336012497	4.813441066	4.39434037	3.840601442	2.890654146	3.55003518
ENSMUSG00000039542.15	Ncam1	-1.09306	4.0832	-5.90409	3.11E-05	0.007194	2.592221	4.422384123	5.123375565	4.35243341	3.741351197	3.481493213	3.378161381
ENSMUSG00000094910.1	D430019H16R	-1.09265	2.320024	-3.67703	0.002301	0.045489	-1.35528	2.871937504	3.4549065	2.28082867	2.263609653	1.61222769	1.436633784
ENSMUSG00000037692.14	Ahdc1	-1.09218	2.679124	-3.76432	0.001928	0.041387	-1.23756	3.116090444	3.883980059	2.67981823	2.657629371	1.965243463	1.771982394
ENSMUSG00000035390.16	Brsk1	-1.09209	1.714381	-3.98281	0.001239	0.032515	-0.745	2.0361669	2.765506589	2.03050532	1.126106129	1.257796956	1.070206228
ENSMUSG00000049791.3	Fzd4	-1.09169	-0.0799	-2.13673	0.049811	0.222753	-3.77224	1.017788371	0.614946913	-0.29142278	0.099633918	-0.522421837	-1.397942607
ENSMUSG00000024501.19	Dpysl3	-1.09168	4.947054	-5.40946	7.69E-05	0.009615	1.615196	5.222234112	5.994845076	5.24691934	4.8465836	4.170762066	4.200978351
ENSMUSG00000028444.17	Cntfr	-1.08939	2.209842	-3.32528	0.004708	0.070356	-2.00332	2.851327224	3.371970159	2.06056255	1.974103035	1.896530713	1.104557733
ENSMUSG00000023232.17	Serinc2	-1.08896	0.595182	-2.17494	0.046343	0.217853	-3.77626	1.158650906	2.274871471	0.1045059	0.099633918	0.380280962	-0.446852208
ENSMUSG00000053046.14	Brsk2	-1.08766	2.828191	-5.52048	6.26E-05	0.009128	2.000644	3.439745284	3.533333147	3.14012981	2.378169417	2.469716046	2.008049752
ENSMUSG00000040118.15	Cacna2d1	-1.08736	2.922576	-5.50673	6.42E-05	0.009164	1.974435	3.397722069	3.745877783	3.24855227	2.69347457	2.192420947	2.257409221
ENSMUSG00000079038.2	D130040H23R	-1.0871	0.087326	-2.76868	0.014519	0.132596	-2.83691	0.487273654	0.725978225	0.67010308	-0.407326071	-0.402127603	-0.549945701
ENSMUSG00000007035.15	Msh5	-1.08661	-0.08014	-2.19185	0.044881	0.215787	-3.69459	-0.019686335	0.494652679	0.74605193	0.474029432	-0.958520951	-1.217370362
ENSMUSG00000025812.17	Pard3	-1.08547	3.593924	-4.09723	0.000984	0.029478	-0.74347	4.120403437	4.418173949	3.82259348	3.32773999	3.587011404	2.287623834
ENSMUSG00000103851.1	Gm37606	-1.08513	0.291417	-2.78636	0.014014	0.129406	-2.80653	0.590367147	1.10037374	0.81820172	-0.011397395	-0.65366637	-0.095379837
ENSMUSG00000019124.10	Scrn1	-1.0851	1.572761	-3.37914	0.004219	0.065529	-1.83427	1.999172692	2.274871471	1.99980852	1.820479846	0.380280962	0.961953338
ENSMUSG00000031075.17	Ano1	-1.08488	0.479598	-2.95959	0.009889	0.105743	-2.52659	1.404811494	0.925287033	0.74605193	-0.011397395	-0.091787482	-0.095379837
ENSMUSG00000034780.6	B3galt1	-1.08433	0.163114	-2.51671	0.023942	0.170532	-3.22505	0.941839517	0.925287033	0.21553721	0.099633918	-0.29109629	-0.91251578
ENSMUSG00000029438.9	Bcl7a	-1.08382	3.598132	-4.28937	0.00067	0.023975	-0.3688	3.994480997	4.589476225	3.77806232	3.732763701	2.959640757	2.534370585
ENSMUSG00000054893.15	Zfp667	-1.08367	0.32368	-2.26966	0.038691	0.206683	-3.6077	1.286975003	0.829071718	0.50504383	0.298942726	-1.345544075	0.367592139
ENSMUSG00000090115.7	Usp49	-1.08251	1.824931	-3.80182	0.001786	0.039559	-1.0788	2.674900657	2.120837843	2.2021167	1.883905227	1.143840766	0.923985488
ENSMUSG00000052371.5	Hoxd3os1	-1.08222	0.288192	-2.19088	0.044964	0.216014	-3.72156	0.124703574	1.699011178	0.74605193	-0.011397395	-1.139093197	0.309876641
ENSMUSG00000039137.18	Whrn	-1.08221	1.543719	-3.13896	0.006877	0.087057	-2.26498	2.361742772	2.510249534	1.39138705	1.367114228	1.103763327	0.528056811
ENSMUSG00000038872.9	Zfhx3	-1.08197	5.437355	-6.9467	5.14E-06	0.004721	4.305535	5.974369301	6.320924814	5.63682286	5.101302738	4.877343693	4.713366976
ENSMUSG00000020723.3	Cacng4	-1.08091	0.916786	-2.9559	0.009963	0.105943	-2.54028	1.224239248	2.07961518	1.07535956	0.836599512	0.163469573	0.121431552
ENSMUSG00000057337.13	Chst3	-1.07958	0.797137	-3.23282	0.005683	0.07859	-2.06797	1.565276166	1.10037374	1.34329276	0.298942726	-0.001589673	0.476526511
ENSMUSG00000024795.10	Kif20b	-1.07936	2.800903	-4.08341	0.001012	0.030012	-0.64406	3.324843189	2.936875008	3.59507603	2.876127876	2.388644435	1.683851484
ENSMUSG00000050240.14	Hic2	-1.0793	1.250656	-3.04705	0.008286	0.096541	-2.40283	1.882133271	2.160915282	1.34329276	0.70229842	1.29386621	0.121431552
ENSMUSG00000039748.11	Exo1	-1.07746	1.951391	-2.96293	0.009823	0.105284	-2.63688	2.674900657	2.346130154	2.28082867	2.657629371	0.786906222	0.961953338
ENSMUSG00000028698.13	Pik3r3	-1.0773	3.414047	-5.69255	4.56E-05	0.008366	2.276208	4.00384918	4.155966066	3.68465139	3.269558919	2.561346521	2.808908963
ENSMUSG00000030671.8	Pde3b	-1.07611	2.513955	-4.07785	0.001023	0.030012	-0.61957	2.851327224	3.388943238	2.87850223	2.620870821	1.429749638	1.914335318
ENSMUSG00000028226.15	Mmp16	-1.07606	1.901636	-3.71888	0.002114	0.043174	-1.23614	2.30224176	2.765506589	2.22883404	1.944659258	0.786906222	1.381667324
ENSMUSG00000047342.16	Zfp286	-1.07508	0.269767	-2.25253	0.039981	0.208688	-3.62938	0.376242341	1.015484842	0.88691447	0.95945626	-0.958520951	-0.660977013
ENSMUSG00000027239.14	Mdk	-1.07399	3.582314	-3.91963	0.001408	0.03453	-1.08975	4.128990933	4.775221744	3.46627426	3.426895457	3.088442528	2.608056133
ENSMUSG00000022053.13	Ebf2	-1.07298	2.557282	-4.22643	0.00076	0.025525	-0.34328	3.06330303	3.578421037	2.71803755	2.11293074	1.583372827	2.287623834
ENSMUSG00000041817.13	Fam169a	-1.07293	1.075228	-2.31187	0.035676	0.20128	-3.62699	2.107425583	1.462943819	1.18857017	0.630148634	1.61222769	-0.549945701
ENSMUSG00000032666.16	1700025G04R	-1.07289	2.624789	-4.42499	0.000512	0.020873	0.022833	2.765809153	3.593142542	3.09727433	2.355973671	2.230140613	1.706394053
ENSMUSG00000035566.6	Pcdh17	-1.07255	2.624412	-5.11109	0.000135	0.011458	1.280034	3.232232039	3.438696273	2.844354	2.086213402	2.21140405	1.933571536
ENSMUSG00000051278.12	Zgrf1	-1.07249	2.336773	-4.1487	0.000888	0.028111	-0.47006	2.892257488	2.865908486	2.75527046	2.463687488	1.748432074	1.295079639
ENSMUSG00000084917.3	Gm17477	-1.07168	0.35951	-2.55654	0.022136	0.164159	-3.17013	0.776780271	1.180544089	0.67010308	0.202727411	0.239418426	-0.91251578
ENSMUSG00000102411.1	Gm36936	-1.07144	1.698555	-3.86914	0.001559	0.036506	-0.95082	2.107425583	2.346130154	2.17489523	1.754137352	0.884753545	0.923985488

ENSMUSG00000034910.5	Pygo1	-1.07136	2.222778	-3.86061	0.001586	0.036884	-0.99954	2.577876203	2.95977541	2.6405588	2.463687488	1.429749638	1.265022405
ENSMUSG00000021198.16	Unc79	-1.07	1.617829	-3.23967	0.005604	0.07801	-2.09031	1.961204842	2.346130154	2.2021167	0.474029432	1.919800492	0.803691254
ENSMUSG00000054178.1	Gm9938	-1.06948	0.184117	-2.51143	0.024192	0.170977	-3.23375	0.861669169	0.925287033	0.31863071	0.202727411	-0.29109629	-0.91251578
ENSMUSG00000019230.14	Lhx9	-1.06927	1.87826	-3.39711	0.004068	0.064065	-1.82862	2.208119583	2.765506589	2.22883404	2.058991933	0.626441549	1.381667324
ENSMUSG00000039683.16	Sdk1	-1.06925	1.162552	-2.44611	0.027495	0.180155	-3.4203	1.404811494	2.765506589	1.01523856	0.554199781	1.182834898	0.052718802
ENSMUSG00000052852.7	Reep1	-1.0679	1.387402	-3.71361	0.002137	0.043572	-1.22614	1.922210711	2.274871471	1.65047427	0.630148634	0.884753545	0.961953338
ENSMUSG00000054423.13	Cadps	-1.0674	2.370832	-4.18708	0.000822	0.026847	-0.40052	2.809201749	3.502472184	2.49407271	1.719785847	1.824380927	1.875075887
ENSMUSG00000044647.16	Csrnp3	-1.06694	2.391278	-4.10567	0.000968	0.029048	-0.55513	2.721070838	3.228478566	2.79156659	2.442778498	1.329055639	1.83471815
ENSMUSG00000031391.18	L1cam	-1.06672	2.566498	-3.93618	0.001361	0.034272	-0.89373	3.232232039	3.650570823	2.51593215	1.820479846	2.284933085	1.894839142
ENSMUSG00000040852.5	Plekhh2	-1.06669	1.042826	-3.26905	0.005279	0.07552	-2.00596	1.709666075	1.948370647	1.13307505	0.474029432	0.568726051	0.423087252
ENSMUSG00000079259.2	Trim71	-1.06572	2.207271	-2.32154	0.035018	0.200218	-3.82417	2.912295242	3.388943238	1.83568914	2.620870821	2.009298643	0.476526511
ENSMUSG00000008575.17	Nfib	-1.06469	3.030158	-4.89063	0.000206	0.014093	0.853388	3.324843189	3.719283573	3.56393752	3.092939265	2.284933085	2.195012684
ENSMUSG00000034685.4	Fam171a2	-1.06336	2.304379	-4.32542	0.000624	0.023244	-0.13875	2.765809153	3.302007601	2.53746531	1.611532956	1.583372827	2.026083675
ENSMUSG00000013076.15	Amotl1	-1.06294	3.488122	-4.44354	0.000493	0.020558	-0.05216	4.103073559	4.418173949	3.52134564	3.32773999	3.088442528	2.469953857
ENSMUSG00000039607.15	Rbms3	-1.06287	1.857148	-3.66932	0.002338	0.045861	-1.3243	2.072236155	2.982317978	2.11886119	1.573565106	1.396959703	0.998947545
ENSMUSG00000040856.17	Dlk1	-1.06236	2.684414	-3.68631	0.002258	0.044897	-1.3882	3.354437971	3.745877783	2.55868179	2.583151155	2.113887544	1.750449233
ENSMUSG00000017499.15	Cdc6	-1.06175	1.809607	-4.07865	0.001022	0.030012	-0.57283	2.674900657	2.310940726	2.03050532	1.494493535	1.143840766	1.202961437
ENSMUSG00000014592.19	Camta1	-1.06167	2.824179	-5.77916	3.90E-05	0.008043	2.448116	3.279280612	3.486790561	3.2744309	2.524655505	2.266899163	2.113019312
ENSMUSG00000087535.1	Zmiz1os1	-1.06092	0.229498	-2.44918	0.027331	0.180136	-3.33031	0.776780271	1.328642728	0.31863071	-0.954813866	0.083299224	-0.175550186
ENSMUSG00000059146.12	Ntrk3	-1.0607	2.393181	-2.99605	0.009187	0.100966	-2.64896	2.932058497	3.907728662	2.06056255	1.883905227	1.824380927	1.750449233
ENSMUSG00000028546.17	Elavl4	-1.06069	3.068635	-4.07788	0.001023	0.030012	-0.69273	3.584135193	4.042553086	3.22220094	2.860446254	1.872879445	2.829593117
ENSMUSG00000070583.1	Fv1	-1.06035	0.090135	-2.47842	0.025811	0.176099	-3.28125	0.376242341	1.10037374	0.41484602	-0.131691629	-0.958520951	-0.260439084
ENSMUSG00000031626.16	Sorbs2	-1.05923	2.752838	-5.43091	7.39E-05	0.009355	1.844624	3.008510557	3.533333147	3.32483586	2.239564179	2.09356756	2.317218617
ENSMUSG00000043857.16	Mgat5b	-1.05758	1.60694	-3.42081	0.003876	0.062141	-1.76117	1.663123489	2.65759125	2.06056255	1.367114228	1.220802748	0.672446721
ENSMUSG00000084941.1	Gm11944	-1.05746	0.038444	-2.50367	0.024564	0.171946	-3.24212	0.376242341	1.015484842	0.31863071	-0.131691629	-0.798056279	-0.549945701
ENSMUSG00000041444.14	Arhgap32	-1.05487	3.091926	-3.41502	0.003922	0.062504	-1.98515	3.608903098	3.883980059	3.24855227	3.448034224	2.633496307	1.728589799
ENSMUSG00000020359.13	Phykpl	-1.05346	3.331923	-4.31843	0.000632	0.023329	-0.26967	3.814535193	4.2047104	3.51049826	3.509653342	2.405227319	2.546915839
ENSMUSG00000034675.17	Dbn1	-1.05337	4.485833	-5.9593	2.82E-05	0.006901	2.65388	5.065541502	5.290762844	4.66277353	4.347561431	3.833599457	3.714757525
ENSMUSG00000030315.15	Vgll4	-1.05222	2.750892	-5.2041	0.000113	0.010533	1.442743	3.369010772	3.302007601	3.14012981	2.563914936	1.872879445	2.257409221
ENSMUSG00000023092.16	Fhl1	-1.0514	1.4773	-3.0545	0.008162	0.095707	-2.40978	2.240180792	2.274871471	1.48301752	1.126106129	1.429749638	0.309876641
ENSMUSG00000027242.14	Wdr76	-1.05109	2.351847	-3.25172	0.005468	0.076896	-2.16378	2.809201749	3.319818877	2.47187697	2.675663294	1.062540664	1.771982394
ENSMUSG00000060572.17	Mfap2	-1.05104	1.51641	-3.18976	0.006203	0.082246	-2.17119	2.332298994	2.446824154	1.39138705	1.322026339	0.681936662	0.923985488
ENSMUSG00000040653.5	Ppp1r14c	-1.0508	0.403927	-2.32844	0.034555	0.199437	-3.52533	0.590367147	1.328642728	0.88691447	0.389140535	-1.139093197	0.367592139
ENSMUSG00000036446.4	Lum	-1.05004	0.583504	-2.6385	0.018821	0.152835	-3.05033	0.376242341	1.643516065	1.29353973	0.298942726	-0.091787482	-0.019430984
ENSMUSG00000037016.11	Frem2	-1.04951	1.91019	-2.64846	0.018453	0.151728	-3.19487	2.418886679	3.337412937	1.56917417	1.944659258	1.429749638	0.761255988
ENSMUSG00000097023.8	Mir9-3hg	-1.0494	1.444581	-3.12042	0.00714	0.089222	-2.28925	1.709666075	2.446824154	1.6894684	1.611532956	0.786906222	0.423087252
ENSMUSG00000061991.2	Hist1h2af	-1.04829	1.115498	-2.58082	0.0211	0.159786	-3.19088	1.460306606	2.380481659	1.07535956	1.410835606	-0.001589673	0.367592139
ENSMUSG00000045045.7	Lrfn4	-1.04809	2.07442	-3.61132	0.00263	0.048817	-1.45141	2.390597634	3.04790632	2.30613976	2.11293074	1.55392905	1.0350168
ENSMUSG00000028565.18	Nfia	-1.04795	2.180994	-4.44185	0.000495	0.020563	0.080344	2.765809153	2.913605228	2.426434	1.852541056	1.77419517	1.35337828
ENSMUSG00000067889.4	Sptbn2	-1.04572	3.645683	-4.55773	0.000394	0.018566	0.144156	4.146014081	4.788074346	3.62555664	3.20893256	3.13889155	2.966629825
ENSMUSG00000028358.15	Zfp618	-1.04512	3.366334	-4.77569	0.000257	0.015525	0.599942	4.094329925	4.095212035	3.477458	3.269558919	2.421621757	2.83982504
ENSMUSG00000029769.16	Ccdc136	-1.04488	0.95228	-2.36436	0.032236	0.193285	-3.52572	1.224239248	2.380481659	0.81820172	0.95945626	0.681936662	-0.350636892
ENSMUSG00000066357.6	Wdr6	-1.04334	3.962246	-4.7633	0.000263	0.015641	0.492769	4.477219989	4.990681451	4.00368153	3.783538268	3.09867445	3.41968065
ENSMUSG00000040478.14	Prdm13	-1.0429	0.524302	-2.7596	0.014785	0.133694	-2.85226	1.017788371	1.180544089	0.88691447	0.298942726	0.311568212	-0.549945701
ENSMUSG00000031284.16	Pak3	-1.04214	1.781249	-3.48068	0.003431	0.057698	-1.66537	2.526802018	2.629302206	1.80049971	1.534570974	0.786906222	1.409412315
ENSMUSG00000025666.16	Tmem47	-1.04165	2.051548	-2.80286	0.013558	0.126871	-2.94427	2.765809153	2.712557709	2.14715024	2.675663294	0.626441549	1.381667324
ENSMUSG00000020649.11	Rrm2	-1.04159	3.612567	-4.49286	0.000447	0.019825	0.023321	4.013156922	4.482843377	3.848667	3.783538268	2.780564695	2.76662916
ENSMUSG00000058773.2	Hist1h1b	-1.04091	3.710572	-3.67503	0.002311	0.045533	-1.5967	4.137527615	4.709182983	3.80494562	4.034744387	2.546473245	3.030557152
ENSMUSG00000064080.12	Fbln2	-1.04046	2.578221	-4.75878	0.000266	0.015701	0.644021	2.809201749	3.354795016	3.12598563	2.031246943	2.354895644	1.79319888
ENSMUSG00000020160.18	Meis1	-1.04016	3.34422	-5.07482	0.000145	0.011551	1.162356	4.040725182	4.053239764	3.477458	3.233489664	2.688725828	2.571683744
ENSMUSG00000033420.12	Antxr1	-1.03969	2.09844	-3.52867	0.003112	0.054302	-1.60814	2.552565114	3.265711472	2.09000633	1.883905227	1.257796956	1.540656848
ENSMUSG00000048728.15	Zfp454	-1.03915	-0.16544	-2.44474	0.027569	0.180155	-3.32714	0.255948108	0.363408146	0.41484602	-0.407326071	-0.958520951	-0.660977013
ENSMUSG00000038526.14	Car14	-1.03912	0.736111	-2.40742	0.02965	0.186632	-3.43246	1.565276166	1.256492942	0.81820172	1.126106129	0.311568212	-0.660977013
ENSMUSG00000087574.1	C030037D09R	-1.0389	0.125125	-2.35395	0.032892	0.19504	-3.46933	0.686582462	0.494652679	0.67010308	0.389140535	-1.139093197	-0.350636892
ENSMUSG00000062980.15	Cped1	-1.03839	1.898519	-3.29328	0.005025	0.073135	-2.02262	1.922210711	3.026373159	2.28082867	1.648527164	1.668261725	0.844913917
ENSMUSG00000102950.1	Gm37018	-1.03792	0.254242	-2.63124	0.019094	0.153707	-3.05029	0.861669169	0.925287033	0.50504383	0.202727411	-0.522421837	-0.446852208
ENSMUSG00000014301.12	Pam16	-1.03773	0.526753	-2.32037	0.035097	0.200239	-3.54849	0.776780271	1.180544089	1.01523856	1.017171757	0.083299224	-0.91251578
ENSMUSG00000004885.5	Crabp2	-1.03741	2.458405	-3.47301	0.003485	0.058241	-1.76152	2.892257488	3.607715344	2.426434	2.484297768	1.824380927	1.51534576
ENSMUSG00000027408.7	Cpxm1	-1.03665	0.892886	-2.51289	0.024123	0.170977	-3.27874	0.861669169	1.643516065	1.56917417	1.322026339	0.311568212	-0.350636892
ENSMUSG00000013415.9	Igf2bp1	-1.03661	5.239398	-4.62774	0.000343	0.017991	0.099714	5.913538409	6.142267521	5.20380111	5.238047946	4.548340811	4.390395089
ENSMUSG00000032607.12	Amt	-1.03628	0.459953	-2.33809	0.033917	0.197603	-3.51534	1.224239248	1.10037374	0.50504383	0.899335267	-0.188002798	-0.781271247
ENSMUSG00000069114.8	Zbtb10	-1.03613	2.961512	-3.96162	0.001293	0.033088	-0.90152	3.546167343	3.650570823	3.15413667	3.145646315	2.437831985	1.83471815
ENSMUSG00000030257.16	Srgap3	-1.0353	4.283898	-5.24305	0.000105	0.010484	1.362595	4.939400472	5.123375565	4.34023296	3.955771213	3.945538053	3.399070371
ENSMUSG00000070822.4	Zscan18	-1.03406	0.385851	-2.59899	0.020355	0.158008	-3.10448	0.590367147	1.180544089	0.95250281	-0.407326071	0.445869304	-0.446852208
ENSMUSG00000103085.1	Gm38120	-1.03327	0.260719	-2.29038	0.037183	0.203612	-3.57219	0.487273654	1.256492942	0.58993273	0.389140535	-1.139093197	-0.019430984
ENSMUSG00000028541.14	B4galt2	-1.03173	1.499753	-3.37107	0.004289	0.066005	-1.84396	1.999172692	2.310940726	1.6894684	1.494493535	0.97638402	0.528056811
ENSMUSG00000042606.9	Hirip3	-1.02966	2.531964	-3.34917	0.004485	0.06816	-2.00954	3.081114306	2.841455443	3.03807854	3.010082333	1.896530713	1.324523417
ENSMUSG00000026930.15	Gpsm1	-1.02888	3.303211	-5.75035	4.11E-05	0.008071	2.385545	3.692388084	4.195092397	3.59507603	2.876127876	2.71556744	2.745015347
ENSMUSG00000035365.15	Parpbp	-1.02795	0.431712	-2.30711	0.036005	0.202153	-3.56032	1.158650906	1.180544089	0.41484602	0.899335267	-0.402127603	-0.660977013
ENSMUSG00000035799.5	Twist1	-1.02794	2.629797	-4.25635	0.000716	0.024814	-0.29453	3.24808599	3.593142542	2.6405588	2.190236466	2.173184728	1.933571536
ENSMUSG00000020000.7	Moxd1	-1.02793	0.950984	-2.94346	0.010217	0.107613	-2.56323	1.158650906	1.853733772	1.34329276	1.017171757	0.083299224	0.249755649
ENSMUSG00000044201.9	Cdc25c	-1.02715	1.018161	-2.21386	0.043042	0.217853	-3.77446	1.089938156	1.585800567	1.76443046	0.836599512	1.493175018	-0.660977013
ENSMUSG00000021066.8	Atl1	-1.0271	0.741651	-3.04693	0.008288	0.096541	-2.38179	1.158650906	1.525679575	1.07535956	0.474029432	0.163469573	0.052718802
ENSMUSG00000035298.8	Klhl35	-1.02681	0.321077	-2.29768	0.036664	0.202683	-3.56566	0.255948108	1.643516065	0.58993273	0.389140535	-0.402127603	-0.549945701
ENSMUSG00000027210.20	Meis2	-1.02672	3.990771	-4.95753	0.000181	0.012958	0.859227	4.581009092	4.544737911	4.32792845	3.808270789	3.802639148	2.88004214
ENSMUSG00000055240.16	Zfp101	-1.02637	1.761849	-2.63094	0.019106	0.153707	-3.20079	2.107425583	2.478885363	2.09000633	2.563914936	0.445869304	0.884991356
ENSMUSG00000016028.9	Celsr1	-1.02594	3.194086	-3.54364	0.003018	0.05369	-1.7546	3.645275488	4.242553751	3.19535933	3.479172736	2.284933085	2.317218617
ENSMUSG00000038143.14	Stox2	-1.0259	4.1097	-3.56753	0.002875	0.052351	-1.88257	4.718228089	4.825959106	4.25831124	4.034744387	4.131435745	2.689520234
ENSMUSG00000052632.15	Asap2	-1.02515	2.662708	-4.64865	0.000329	0.017703	0.43587	3.081114306	3.247215128	3.12598563	2.71106863	1.896530713	1.914335318
ENSMUSG00000101174.6	Hoxd4	-1.0242	1.249944	-2.97875	0.009514	0.103577	-2.52257	2.0361669	1.853733772	1.39138705	0.70229842	1.329055639	0.187019893
ENSMUSG00000046442.4	Ppm1e	-1.02389	1.671769	-2.74121	0.015339	0.136818	-2.99187	2.500570476	2.310940726	1.65047427	2.215111135	0.508605059	0.844913917
ENSMUSG00000025255.18	Zfhx4	-1.02388	5.129131	-5.44907	7.14E-05	0.009244	1.677951	5.557344892	6.008637677	5.35091399	4.798017049	4.895063814	4.164808614
ENSMUSG00000004562.16	Arhgef40	-1.02382	3.689248	-5.27512	9.89E-05	0.010268	1.493029	4.067776506	4.529511436	3.97240236	3.587920399	3.158587352	2.819288109
ENSMUSG00000041688.16	Amot	-1.02274	3.033446	-3.7224	0.002099	0.043063	-1.37687	3.411866251	3.942634277	3.23543677	3.316289969	1.919800492	2.374646897
ENSMUSG00000103585.1	Pcdhgb4	-1.02229	0.935593	-2.34715	0.033328	0.196152	-3.55116	1.347095996	2.199909414	0.81820172	1.227389465	-0.402127603	0.423087252
ENSMUSG00000030393.8	Zik1	-1.02157	1.539451	-2.51181	0.024174	0.170977	-3.36824	1.663123489	3.04790632	1.5267389	1.494493535	0.239418426	1.265022405
ENSMUSG00000028347.14	Tmeff1	-1.02138	3.461438	-5.05518	0.00015	0.011837	1.112451	3.793082083	4.434615096	3.71331514	3.20893256	2.633496307	2.985185644
ENSMUSG00000069227.4	Gprin1	-1.01801	1.779751	-3.52428	0.00314	0.054506	-1.58555	2.072236155	2.600447343	2.14715024	1.883905227	0.836659257	1.138110293
ENSMUSG00000044066.14	Cep68	-1.01774	2.780153	-3.98316	0.001238	0.032515	-0.83328	3.520288711	3.265711472	2.99203117	2.995798107	2.05204829	1.855038134
ENSMUSG00000042099.15	Kank3	-1.01756	1.171889	-3.52257	0.003151	0.054625	-1.56427	1.798475342	1.752450437	1.5267389	0.554199781	0.681936662	0.71753461
ENSMUSG00000032113.15	Chek1	-1.01619	1.836879	-3.35392	0.004442	0.067651	-1.90432	2.526802018	2.380481659	2.03050532	2.058991933	1.220802748	0.803691254
ENSMUSG00000066705.6	Fxyd6	-1.01616	1.241773	-2.78128	0.014157	0.130303	-2.86459	1.798475342	2.414034219	1.07535956	1.17763643	0.508605059	0.476526511
ENSMUSG00000025912.16	Mybl1	-1.01595	1.061695	-2.77131	0.014443	0.132202	-2.86421	1.224239248	1.462943819	1.90359321	0.77101117	1.103763327	-0.095379837
ENSMUSG00000047085.14	Lrrc4b	-1.01584	1.873621	-3.29376	0.00502	0.073135	-2.01861	2.500570476	2.982317978	1.72743625	1.534570974	1.29386621	1.202961437
ENSMUSG00000021745.13	Ptprg	-1.0158	3.368736	-5.38434	8.06E-05	0.009818	1.727101	3.917261495	4.042553086	3.63557539	3.183950203	2.937009782	2.496066697
ENSMUSG00000033149.16	Phldb2	-1.01568	3.26077	-5.25287	0.000103	0.010484	1.498021	3.897293078	3.96544416	3.43219326	3.092939265	2.605067899	2.571683744
ENSMUSG00000036790.5	Slitrk2	-1.01517	0.700035	-2.52544	0.023535	0.168973	-3.24012	0.941839517	1.699011178	1.07535956	0.202727411	-0.522421837	0.803691254
ENSMUSG00000039963.18	Ccdc40	-1.01494	0.694779	-2.92202	0.010668	0.110463	-2.58982	0.861669169	1.585800567	1.18857017	0.099633918	0.311568212	0.121431552
ENSMUSG00000012017.3	Scarf2	-1.01409	2.383114	-3.99856	0.0012	0.031957	-0.75579	3.199991702	3.069122806	2.426434	2.164925378	1.523871816	1.914335318
ENSMUSG00000039943.16	Plcb4	-1.01401	1.585389	-2.77135	0.014442	0.132202	-2.9262	1.922210711	1.803980737	2.35546747	1.719785847	1.523871816	0.187019893
ENSMUSG00000034023.16	Fancd2	-1.014	1.86662	-3.20208	0.006049	0.081173	-2.18727	2.552565114	1.853733772	2.53746531	2.190236466	1.103763327	0.961953338
ENSMUSG00000028399.17	Ptprd	-1.01335	3.325378	-3.73624	0.002041	0.042323	-1.40345	3.703929706	4.105517043	3.60530795	3.304748347	3.20668164	2.026083675
ENSMUSG00000043635.12	Adamts3	-1.01312	0.679807	-2.44387	0.027616	0.180155	-3.3691	1.158650906	1.10037374	1.24200942	0.899335267	-0.798056279	0.476526511
ENSMUSG00000031217.8	Efnb1	-1.01272	2.483666	-3.92105	0.001403	0.034494	-0.91334	3.167014379	3.265711472	2.51593215	2.442778498	1.896530713	1.614030034
ENSMUSG00000039976.3	Tbc1d16	-1.01148	3.457233	-3.86931	0.001558	0.036506	-1.16647	3.897293078	4.552291305	3.45500315	3.479172736	2.469716046	2.889923652
ENSMUSG00000046295.13	Ankle1	-1.01103	0.758727	-2.65476	0.018223	0.150647	-3.03485	1.089938156	1.015484842	1.5267389	1.017171757	0.163469573	-0.260439084
ENSMUSG00000024462.17	Gabbr1	-1.01078	3.313378	-4.28158	0.00068	0.024093	-0.33859	3.846128318	3.758993282	3.73211274	3.489404658	2.842759456	2.210866635
ENSMUSG00000105265.4	Sox2ot	-1.0103	1.005083	-2.19385	0.044711	0.215501	-3.80332	2.208119583	1.10037374	1.24200942	0.899335267	-0.65366637	1.234325608
ENSMUSG00000061353.11	Cxcl12	-1.00855	2.223244	-3.34596	0.004514	0.068446	-1.96679	2.651249389	3.486790561	2.11886119	1.914602024	1.55392905	1.614030034
ENSMUSG00000055675.6	Kbtbd11	-1.00808	0.674088	-2.17537	0.046305	0.217853	-3.78583	1.158650906	2.07961518	0.31863071	0.77101117	0.163469573	-0.446852208
ENSMUSG00000031990.14	Jam3	-1.00718	2.060244	-4.55317	0.000397	0.018566	0.283661	2.577876203	2.685336241	2.426434	1.684596418	1.523871816	1.463351122
ENSMUSG00000020900.15	Myh10	-1.00628	5.500386	-4.79635	0.000247	0.015137	0.417499	6.08000752	6.215454558	5.69785967	5.565124743	4.98881517	4.45505498
ENSMUSG00000020532.18	Acaca	-1.00585	3.322997	-4.55451	0.000396	0.018566	0.185154	3.83567396	4.223756155	3.40901694	3.171294903	2.854883015	2.443359647
ENSMUSG00000062175.13	Tgif2	-1.00521	0.481058	-2.64359	0.018632	0.152281	-3.03741	0.941839517	1.462943819	0.58993273	-0.011397395	0.163469573	-0.260439084
ENSMUSG00000024590.8	Lmnb1	-1.0036	4.595768	-5.74441	4.15E-05	0.008071	2.257623	5.078899885	5.317604456	4.86791748	4.580760607	3.851861663	3.877561151
ENSMUSG00000056445.8	Hoxaas2	-1.00342	0.590935	-2.21213	0.043184	0.212903	-3.72057	0.941839517	1.462943819	0.74605193	1.17763643	-0.001589673	-0.781271247

ENSMUSG00000032549.7	Rab6b	-1.00285	2.30429	-4.95742	0.000181	0.012958	1.008192	2.809201749	2.88995396	2.71803755	1.883905227	1.77419517	1.750449233
ENSMUSG00000037605.16	Adgrl3	-1.00267	2.811048	-3.87545	0.001539	0.036283	-1.04458	3.383437843	3.486790561	3.02289214	2.54441876	2.767797399	1.660951082
ENSMUSG00000035835.14	Plppr3	-1.00257	3.296988	-3.70275	0.002184	0.043943	-1.46312	4.058815639	4.288503337	3.08270153	3.010082333	2.925559761	2.416265986
ENSMUSG00000066392.11	Nrxn3	-1.00207	1.493839	-2.60459	0.02013	0.157417	-3.20251	1.709666075	2.346130154	1.93638314	0.630148634	2.030831804	0.309876641
ENSMUSG00000031906.8	Smpd3	-1.00204	1.233715	-2.88379	0.011523	0.115391	-2.68641	1.347095996	2.540946331	1.39138705	0.836599512	0.568726051	0.71753461
ENSMUSG00000066829.6	Zfp810	-1.00148	1.399691	-3.55298	0.002962	0.053131	-1.51293	1.922210711	1.853733772	1.87004065	1.322026339	0.626441549	0.803691254
ENSMUSG00000000305.12	Cdh4	-0.9997	0.424102	-2.56548	0.021749	0.162769	-3.15899	0.941839517	1.10037374	0.67010308	0.474029432	-0.091787482	-0.549945701
ENSMUSG00000032826.16	Ank2	-0.99861	4.527594	-5.07463	0.000145	0.011551	1.01808	5.096520807	5.411959891	4.57178538	4.218190528	4.209044777	3.658062802
ENSMUSG00000028438.16	Kif24	-0.99734	2.029899	-4.33521	0.000612	0.022923	-0.11046	2.473853138	2.600447343	2.49407271	1.787689911	1.257796956	1.565531517
ENSMUSG00000036006.19	Ripor2	-0.99726	1.05691	-2.85215	0.01228	0.119933	-2.72594	1.709666075	1.853733772	1.07535956	1.07266687	0.508605059	0.121431552
ENSMUSG00000021589.12	Rhobtb3	-0.99717	3.31453	-5.04293	0.000154	0.011953	1.10629	3.994480997	3.919457955	3.4995687	3.245613223	2.561346521	2.666710351
ENSMUSG00000056214.8	Pard6g	-0.99688	1.339768	-3.03612	0.008471	0.097312	-2.42964	1.922210711	2.237877264	1.34329276	1.275483753	0.836659257	0.423087252
ENSMUSG00000029245.16	Epha5	-0.99624	2.046208	-3.70516	0.002174	0.043866	-1.2739	2.830418235	2.446824154	2.28082867	2.031246943	1.583372827	1.104557733
ENSMUSG00000025938.16	Slco5a1	-0.99595	2.267428	-4.27682	0.000687	0.024172	-0.22699	2.651249389	2.982317978	2.62052105	2.164925378	1.722200532	1.463351122
ENSMUSG00000089417.1	Gm22009	-0.99577	3.313829	-3.902	0.001459	0.03514	-1.07726	3.493937384	3.732641955	4.09364491	3.119533475	3.280445694	2.162772347
ENSMUSG00000097769.7	Snhg4	-0.9953	0.308297	-2.42553	0.028622	0.183246	-3.37061	0.255948108	1.462943819	0.74605193	-0.262936162	-0.091787482	-0.260439084
ENSMUSG00000036295.4	Lrrn3	-0.99518	0.754234	-2.8927	0.011318	0.113949	-2.64035	1.347095996	1.180544089	1.29353973	-0.131691629	0.163469573	0.672446721
ENSMUSG00000063919.10	Srrm4	-0.99471	3.070633	-4.12947	0.000922	0.028569	-0.59412	3.425873109	4.155966066	3.15413667	2.69347457	2.767797399	2.226548258
ENSMUSG00000024044.15	Epb41l3	-0.99425	2.304152	-3.65386	0.002412	0.046328	-1.39744	2.871937504	3.354795016	2.25506558	1.719785847	2.009298643	1.614030034
ENSMUSG00000032368.14	Zic1	-0.99419	3.103387	-3.31801	0.004778	0.071009	-2.17603	3.324843189	3.883980059	3.46627426	3.405442347	2.767797399	1.771982394
ENSMUSG00000013367.5	Iglon5	-0.99219	0.995807	-2.62557	0.019311	0.154133	-3.10316	1.286975003	2.120837843	1.07535956	0.70229842	0.884753545	-0.095379837
ENSMUSG00000075324.13	Fign	-0.99194	2.65175	-3.83871	0.001658	0.03778	-1.09177	3.279280612	3.548519551	2.6405588	2.563914936	1.748432074	2.129795025
ENSMUSG00000028678.13	Kif2c	-0.99178	2.312611	-3.77715	0.001878	0.040649	-1.16606	2.989773994	3.026373159	2.37951295	2.355973671	1.583372827	1.540656848
ENSMUSG00000003934.5	Efnb3	-0.99109	2.006298	-4.29783	0.000659	0.023696	-0.17803	2.446631669	2.571003565	2.4493344	1.820479846	1.396959703	1.35337828
ENSMUSG00000025350.15	Rdh5	-0.99086	-0.02334	-2.32243	0.034957	0.200218	-3.50922	0.124703574	0.494652679	0.81820172	-0.748362989	-0.65366637	-0.175550186
ENSMUSG00000102252.5	Snrpn	-0.99082	1.177639	-3.1396	0.006868	0.087026	-2.23625	1.961204842	1.803980737	1.29353973	0.554199781	0.97638402	0.476526511
ENSMUSG00000033295.13	Ptprf	-0.98906	4.98175	-4.20627	0.000791	0.026141	-0.71675	5.438018539	6.073066394	4.91392034	4.830575724	4.566884705	4.068031857
ENSMUSG00000000690.5	Hoxb6	-0.98776	1.711404	-2.91446	0.010832	0.111558	-2.68897	2.271544963	2.841455443	1.5267389	1.573565106	1.29386621	0.761255988
ENSMUSG00000031112.10	Stk26	-0.98728	0.436493	-2.58931	0.020748	0.158889	-3.12196	0.861669169	0.494652679	1.34329276	0.202727411	-0.188002798	-0.095379837
ENSMUSG00000036902.9	Neto2	-0.98678	1.858897	-3.09911	0.007456	0.091098	-2.37449	2.361742772	2.414034219	2.30613976	1.648527164	0.508605059	1.914335318
ENSMUSG00000075514.4	Gm13375	-0.98672	2.33447	-4.11873	0.000943	0.028746	-0.52606	2.627203915	3.090031796	2.71803755	2.215111135	1.919800492	1.436633784
ENSMUSG00000025395.14	Prim1	-0.98631	1.514255	-2.71878	0.016042	0.140453	-3.00815	1.961204842	2.120837843	1.80049971	1.787689911	1.29386621	0.121431552
ENSMUSG00000039081.9	Zfp503	-0.98386	2.907972	-4.48805	0.000452	0.019825	0.112272	3.397722069	3.578421037	3.16800884	2.90698884	2.388644435	2.008049752
ENSMUSG00000063455.16	D630045J12Ri	-0.98364	3.28614	-3.97664	0.001255	0.032787	-0.92635	3.645275488	4.242553751	3.42065164	3.145646315	3.036165249	2.226548258
ENSMUSG00000050730.16	Arhgap42	-0.98336	1.6768	-3.02313	0.008697	0.098507	-2.4893	2.361742772	2.199909414	1.83568914	1.914602024	1.220802748	0.528056811
ENSMUSG00000100158.1	Gm28119	-0.98242	0.157769	-2.44405	0.027606	0.180155	-3.33545	1.017788371	0.614946913	0.31863071	-0.131691629	-0.522421837	-0.350636892
ENSMUSG00000024376.6	Epb41l4a	-0.9813	1.77049	-3.07775	0.007786	0.093204	-2.40193	2.240180792	2.865908486	1.6894684	1.684596418	1.257796956	0.884991356
ENSMUSG00000029414.11	Kntc1	-0.98109	3.248283	-3.73838	0.002032	0.042311	-1.38542	3.814535193	3.486790561	3.75985773	3.671175903	2.469716046	2.287623834
ENSMUSG00000022199.10	Slc22a17	-0.98086	2.634139	-4.14768	0.000889	0.028111	-0.50095	3.150238667	3.283973678	2.92825526	2.139162282	2.619352125	1.683851484
ENSMUSG00000056427.10	Slit3	-0.98005	1.640706	-2.91539	0.010812	0.111434	-2.67751	2.332298994	2.739275048	1.39138705	1.275483753	1.220802748	0.884991356
ENSMUSG00000003070.6	Efna2	-0.97981	1.41885	-3.27061	0.005262	0.075492	-2.01891	2.072236155	2.274871471	1.43792963	1.017171757	0.786906222	0.923985488
ENSMUSG00000058070.14	Eml1	-0.97952	2.213882	-2.97495	0.009587	0.103779	-2.65814	2.577876203	3.04790632	2.40316422	2.728450708	1.062540664	1.463351122
ENSMUSG00000014030.15	Pax5	-0.9795	0.652443	-2.38907	0.030727	0.188787	-3.453	0.776780271	2.07961518	0.67010308	0.202727411	-0.001589673	0.187019893
ENSMUSG00000046341.5	Gm11223	-0.97928	3.65757	-5.40614	7.74E-05	0.009615	1.737383	3.936957296	4.618549149	3.92417408	3.233489664	3.057304016	3.174947061
ENSMUSG00000055546.6	Timd4	-0.97849	0.072057	-2.41766	0.029065	0.184844	-3.37208	0.487273654	0.363408146	0.81820172	-0.407326071	-0.65366637	-0.175550186
ENSMUSG00000030519.14	Apba2	-0.97653	1.552849	-3.48462	0.003404	0.057409	-1.64219	2.107425583	2.414034219	1.65047427	1.126106129	1.020105398	0.998947545
ENSMUSG00000015053.14	Gata2	-0.97645	0.78199	-2.85386	0.012238	0.119766	-2.70632	1.089938156	1.643516065	1.13307505	0.298942726	-0.001589673	0.528056811
ENSMUSG00000052512.15	Nav2	-0.97631	4.0874	-4.37595	0.000564	0.021686	-0.27801	4.555753392	5.178105043	4.04936435	3.489404658	3.845799911	3.405973231
ENSMUSG00000074519.11	Zfp971	-0.97564	0.534978	-2.57715	0.021253	0.160616	-3.1462	0.941839517	0.614946913	1.48301752	-0.262936162	0.311568212	0.121431552
ENSMUSG00000029814.10	Igf2bp3	-0.97498	3.53266	-4.77129	0.000259	0.015528	0.568245	4.204061366	3.96544416	3.81379654	3.587920399	2.992935197	2.631804736
ENSMUSG00000061887.14	Ssbp3	-0.97476	4.681265	-6.37739	1.35E-05	0.005171	3.373274	5.065541502	5.557930511	4.89319161	4.301848457	4.141368281	4.127708698
ENSMUSG00000026558.13	Uck2	-0.97467	3.492411	-3.76967	0.001907	0.041006	-1.36921	3.975560135	4.552291305	3.40901694	3.519672098	2.842759456	2.655168729
ENSMUSG00000106457.1	Gm42585	-0.97399	0.474088	-2.18641	0.045346	0.216675	-3.74672	0.487273654	1.180544089	1.07535956	0.70229842	0.311568212	-0.91251578
ENSMUSG00000048027.7	Rgmb	-0.97347	3.707674	-5.45716	7.04E-05	0.009244	1.824961	4.187713372	4.5063661	3.91597693	3.405442347	2.902383439	3.328161994
ENSMUSG00000054555.11	Adam12	-0.9727	0.351815	-2.26969	0.038688	0.206683	-3.61033	0.861669169	1.10037374	0.50504383	0.70229842	-0.798056279	-0.260439084
ENSMUSG00000020493.8	Prr11	-0.97261	1.705453	-3.46725	0.003526	0.058645	-1.68436	2.072236155	2.120837843	2.28082867	1.611532956	1.429749638	0.71753461
ENSMUSG00000047181.12	Samd14	-0.97206	2.572702	-4.01601	0.001159	0.03138	-0.7434	3.199991702	3.486790561	2.57959078	2.031246943	1.896530713	2.242061257
ENSMUSG00000030428.16	Ttyh1	-0.97184	1.114545	-2.34499	0.033468	0.196453	-3.57993	1.513745865	1.993458536	1.18857017	1.410835606	0.931296131	-0.350636892
ENSMUSG00000030029.14	Lrig1	-0.9718	2.372172	-4.22441	0.000763	0.025825	-0.33162	2.912295242	3.004513725	2.62052105	2.215111135	1.965243463	1.51534576
ENSMUSG00000037169.14	Mycn	-0.97108	2.133649	-3.26759	0.005295	0.075533	-2.10127	2.743613407	3.090031796	2.03050532	2.164925378	1.363407143	1.409412315
ENSMUSG00000029376.8	Mthfd2l	-0.97017	1.732286	-3.72042	0.002107	0.043105	-1.22312	2.500570476	2.037179913	2.09000633	1.494493535	0.97638402	1.295079639
ENSMUSG00000028718.16	Stil	-0.96983	2.187078	-2.85509	0.012208	0.119575	-2.87402	2.627203915	2.791269685	2.4493344	2.602134258	1.848833971	0.803691254
ENSMUSG00000060716.6	Plekhh1	-0.9697	0.697015	-2.58536	0.020911	0.159164	-3.14342	0.941839517	1.803980737	0.81820172	0.554199781	0.083299224	-0.019430984
ENSMUSG00000046572.10	Zfp518b	-0.96943	3.138879	-4.49571	0.000445	0.019825	0.098243	3.596572296	3.942634277	3.32483586	3.106297648	2.266899163	2.596033605
ENSMUSG00000024302.15	Dtna	-0.96891	0.606502	-2.25586	0.039727	0.208663	-3.65546	1.089938156	1.585800567	0.58993273	0.298942726	0.735375921	-0.660977013
ENSMUSG00000052713.8	Zfp608	-0.96868	3.07048	-4.29975	0.000656	0.023674	-0.26705	3.533286052	3.758993282	3.32483586	2.90698884	2.854883015	2.043894951
ENSMUSG00000008489.18	Elavl2	-0.9685	2.861593	-3.90492	0.00145	0.034996	-0.99536	3.199991702	3.758993282	3.12598563	2.620870821	1.77419517	2.689520234
ENSMUSG00000040717.5	Il17rd	-0.96707	2.955044	-4.68581	0.000306	0.017041	0.479058	3.692388084	3.388943238	3.19535933	2.891640875	2.230140613	2.331791418
ENSMUSG00000025592.17	Dach2	-0.96649	0.389242	-2.2457	0.040506	0.209437	-3.6496	0.776780271	1.180544089	0.58993273	0.389140535	0.311568212	-0.91251578
ENSMUSG00000053646.13	Plxnb1	-0.96622	4.775906	-4.21327	0.00078	0.02597	-0.68547	5.173255319	5.565415327	5.00561414	4.73920911	4.566884705	3.605059881
ENSMUSG00000023800.14	Tiam2	-0.96617	0.575382	-2.53589	0.023055	0.167189	-3.21438	1.460306606	1.180544089	0.58993273	0.202727411	-0.29109629	0.309876641
ENSMUSG00000097545.7	Mir124a-1hg	-0.9636	2.442535	-3.78172	0.001861	0.040404	-1.17292	2.651249389	3.4549065	2.66032206	2.287260921	1.987439209	1.614030034
ENSMUSG00000075394.4	Hoxc4	-0.9634	1.787086	-2.88228	0.011558	0.115571	-2.75821	2.418886679	2.478885363	1.83568914	1.820479846	1.640516735	0.528056811
ENSMUSG00000024502.9	Jakmip2	-0.96303	2.102271	-2.52461	0.023573	0.169161	-3.45128	2.871937504	2.478885363	2.25506558	2.287260921	2.192420947	0.528056811
ENSMUSG00000060301.5	2610008E11Ri	-0.96281	2.865778	-5.19662	0.000115	0.010533	1.424356	3.493937384	3.170763068	3.32483586	2.639367164	2.354895644	2.210866635
ENSMUSG00000020642.12	Rnf144a	-0.96255	2.607367	-4.33846	0.000608	0.022923	-0.13823	3.027006901	3.302007601	2.89527794	2.602134258	1.942700894	1.875075887
ENSMUSG00000027993.16	Trim2	-0.96175	3.674374	-4.47608	0.000463	0.020085	-0.01919	4.195910525	4.46694575	3.7870792	3.715433822	2.780564695	3.10030826
ENSMUSG00000063632.6	Sox11	-0.96098	6.857468	-5.07497	0.000145	0.011551	0.953127	7.345715206	7.699436438	6.96794884	6.814896851	6.076197109	6.240613979
ENSMUSG00000030806.6	Stx1b	-0.9597	1.131763	-2.95883	0.009905	0.10582	-2.54822	1.615029201	1.585800567	1.56917417	0.836599512	1.062540664	0.121431552
ENSMUSG00000006369.14	Fbln1	-0.9595	2.71112	-4.39809	0.00054	0.021429	-0.03624	3.045269107	3.486790561	3.00754417	2.602134258	2.230140613	1.894839142
ENSMUSG00000052430.15	Bmpr1b	-0.95943	1.621258	-2.61598	0.019681	0.155538	-3.20376	2.240180792	1.948370647	1.93638314	2.058991933	1.29386621	0.249755649
ENSMUSG00000006575.14	Rundc3a	-0.95809	2.098455	-4.04757	0.001087	0.030593	-0.64148	2.446631669	2.65759125	2.60020106	1.648527164	1.942700894	1.295079639
ENSMUSG00000002799.5	Jag2	-0.9571	0.964958	-2.27958	0.037961	0.205148	-3.66315	0.941839517	1.853733772	1.43792963	0.899335267	1.103763327	-0.446852208
ENSMUSG00000060206.11	Zfp462	-0.95666	5.453646	-5.36891	8.30E-05	0.009818	1.513818	5.888469228	6.189048421	5.70739031	5.348966882	5.074254085	4.513748975
ENSMUSG00000031616.7	Ednra	-0.95651	1.516005	-2.9921	0.009261	0.101526	-2.52532	2.072236155	2.380481659	1.61039683	1.17763643	0.445869304	1.409412315
ENSMUSG00000079065.3	BC005561	-0.95645	2.453574	-2.98323	0.009428	0.102859	-2.68611	3.216201929	2.841455443	2.66032206	2.190236466	2.675115396	1.138110293
ENSMUSG00000005718.7	Tfap4	-0.95462	0.625974	-2.54822	0.022502	0.165568	-3.19825	1.224239248	1.328642728	0.74605193	0.298942726	0.508605059	-0.350636892
ENSMUSG00000026463.17	Atp2b4	-0.95453	2.158836	-3.00915	0.008947	0.09963	-2.58511	2.851327224	3.13096206	1.99980852	1.494493535	2.21140405	1.265022405
ENSMUSG00000059921.15	Unc5c	-0.95405	3.203377	-3.92397	0.001395	0.034469	-1.01513	3.83567396	3.593142542	3.4995687	3.426895457	2.702209057	2.162772347
ENSMUSG00000021256.5	Vash1	-0.95401	2.466993	-3.52041	0.003165	0.054795	-1.6732	2.851327224	3.388943238	2.57959078	2.583151155	1.523871816	1.875075887
ENSMUSG00000105383.4	#N/A	-0.95384	0.588007	-2.4006	0.030046	0.187691	-3.42931	0.590367147	1.462943819	1.13307505	0.554199781	-0.522421837	0.309876641
ENSMUSG00000025265.14	Fgd1	-0.95357	1.268006	-2.76549	0.014612	0.133032	-2.89498	1.798475342	2.346130154	1.13307505	1.126106129	0.626441549	0.577809846
ENSMUSG00000026594.14	Ralgps2	-0.95335	2.860466	-4.06058	0.001059	0.030247	-0.69616	3.216201929	3.758993282	3.02289214	2.876127876	2.09356756	2.195012684
ENSMUSG00000025203.5	Scd2	-0.95308	6.044205	-4.72105	0.000286	0.016299	0.260711	6.716092344	6.764694032	6.07627935	6.078609135	5.277070968	5.352485247
ENSMUSG00000055320.16	Tead1	-0.95238	3.673726	-3.89632	0.001475	0.035482	-1.15391	4.228241179	4.426437943	3.74142048	3.715433822	3.35915767	2.571683744
ENSMUSG00000031024.12	St5	-0.9521	2.223386	-3.62092	0.002579	0.048607	-1.45046	3.027006901	2.600447343	2.40316422	2.190236466	1.824380927	1.295079639
ENSMUSG00000052981.5	Ube2ql1	-0.95197	0.260581	-2.3955	0.030346	0.188174	-3.41392	0.686582462	1.10037374	0.41484602	0.099633918	-0.188002798	-0.549945701
ENSMUSG00000060969.8	Irx1	-0.95175	0.408428	-2.17815	0.046062	0.217853	-3.75219	1.089938156	0.725978225	0.81820172	-0.407326071	0.884753545	-0.660977013
ENSMUSG00000020019.4	Ntn4	-0.95116	0.258117	-2.42436	0.028688	0.183578	-3.36991	0.776780271	0.614946913	0.74605193	-0.011397395	0.083299224	-0.660977013
ENSMUSG00000089235.1	Gm23119	-0.95069	0.637198	-2.20449	0.043817	0.214195	-3.73761	0.941839517	0.829071718	1.43792963	0.298942726	0.97638402	-0.660977013
ENSMUSG00000025050.7	Pcgf6	-0.95007	1.084081	-2.71608	0.016128	0.140566	-2.95997	1.663123489	1.948370647	1.07535956	1.126106129	0.163469573	0.528056811
ENSMUSG00000074476.5	Spc24	-0.94864	1.172472	-2.82618	0.012938	0.124043	-2.78067	1.460306606	1.643516065	1.76443046	1.322026339	0.083299224	0.761255988
ENSMUSG00000024597.10	Slc12a2	-0.94771	2.795054	-3.41897	0.003891	0.062194	-1.92171	3.669024091	3.371970159	2.71803755	2.891640875	2.284933085	1.83471815
ENSMUSG00000097816.1	Gm26811	-0.94749	0.422083	-2.57705	0.021257	0.160616	-3.14068	0.776780271	1.015484842	0.88691447	0.202727411	-0.402127603	0.052718802
ENSMUSG00000026556.15	Vangl2	-0.94719	3.932595	-4.65682	0.000324	0.017686	0.290711	4.463705183	4.60408592	4.10089465	4.027740999	3.315959981	3.083184082
ENSMUSG00000062044.13	Lmtk3	-0.947	3.233538	-2.82297	0.013022	0.124635	-3.15256	3.793082083	3.919457955	3.2998535	3.416208778	3.244035054	1.728589799
ENSMUSG00000029761.16	Cald1	-0.94689	3.442753	-5.58952	5.51E-05	0.008819	2.090025	3.897293078	4.242553751	3.64552505	2.995798107	2.937009782	2.938340781
ENSMUSG00000071658.4	Gng3	-0.94596	0.092393	-2.22145	0.042425	0.212176	-3.66238	0.861669169	0.829071718	0.1045059	-0.567790743	-0.65366637	-0.019430984
ENSMUSG00000027901.12	Dennd2d	-0.94572	0.121874	-2.18517	0.045453	0.216816	-3.71709	1.224239248	0.494652679	0.1045059	-0.262936162	-0.65366637	-0.175550186
ENSMUSG00000024222.16	Fkbp5	-0.94465	2.546236	-4.16736	0.000855	0.027359	-0.4544	2.932058497	3.354795016	2.75527046	2.463687488	1.896530713	1.875075887
ENSMUSG00000033952.14	Aspm	-0.94461	3.724109	-3.77062	0.001903	0.041006	-1.41162	4.306033339	4.105517043	4.05683947	3.992204842	3.324703614	2.559352942
ENSMUSG00000033031.13	C330027C09Ri	-0.94373	2.655007	-3.78567	0.001846	0.040147	-1.19435	3.279280612	3.090031796	2.92825526	2.620870821	2.421621757	1.58998456
ENSMUSG00000035863.13	Palm	-0.94329	2.865148	-4.82126	0.000235	0.014899	0.739421	3.324843189	3.607715344	3.08270153	2.639367164	2.248636956	2.287623834
ENSMUSG00000051331.14	Cacna1c	-0.94305	2.396916	-3.59423	0.002723	0.050335	-1.52305	2.602750871	3.228478566	2.73677412	2.164925378	2.266899163	1.381667324
ENSMUSG00000049230.8	Gm9833	-0.94286	2.767109	-3.90774	0.001442	0.03486	-0.97633	3.279280612	3.517985183	2.87850223	2.860446254	2.113887544	1.95255464
ENSMUSG00000030551.13	Nr2f2	-0.94109	3.666499	-3.71071	0.002149	0.043697	-1.51874	4.415380735	4.393155489	3.57439188	3.840601442	2.818202352	2.957261643

ENSMUSG00000055717.13	Slain1	-0.93958	0.59553	-2.58081	0.0211	0.159786	-3.1439	1.404811494	1.180544089	0.67010308	0.099633918	-0.091787482	0.309876641
ENSMUSG00000020808.3	Pimreg	-0.93913	1.379173	-3.07598	0.007814	0.093372	-2.36271	1.798475342	2.274871471	1.48301752	1.275483753	0.681936662	0.761255988
ENSMUSG00000032875.7	Arhgef17	-0.93829	3.401756	-4.37826	0.000561	0.021658	-0.16578	3.994480997	4.042553086	3.53211207	3.499564524	2.675115396	2.666710351
ENSMUSG00000048915.12	Efna5	-0.93818	2.53983	-3.62159	0.002576	0.048607	-1.49133	2.951554672	3.319818877	2.69905445	2.71106863	1.896530713	1.660951082
ENSMUSG00000094338.1	Hist1h2bl	-0.93809	2.337452	-2.64511	0.018576	0.152181	-3.28367	2.871937504	3.438696273	2.17489523	2.400028851	0.97638402	2.162772347
ENSMUSG00000027784.10	Ppm1l	-0.9377	2.649743	-4.87787	0.000211	0.014141	0.857676	3.232232039	3.04790632	3.03807854	2.442778498	2.09356756	2.043894951
ENSMUSG00000041073.10	Nacad	-0.93761	1.284783	-2.52282	0.023656	0.169529	-3.3108	1.615029201	2.571003565	1.13307505	0.95945626	1.062540664	0.367592139
ENSMUSG00000046546.3	Fam43a	-0.93748	0.872892	-2.59556	0.020494	0.158008	-3.14102	1.224239248	1.803980737	1.13307505	-0.011397395	0.163469573	0.923985488
ENSMUSG00000037172.14	E330009J07Ril	-0.93703	2.133052	-3.91334	0.001426	0.034672	-0.89339	2.851327224	2.540946331	2.37951295	2.031246943	1.429749638	1.565531517
ENSMUSG00000042489.15	Clspn	-0.93549	2.426913	-3.02667	0.008635	0.098298	-2.60031	2.970790891	2.913605228	2.66032206	2.922175243	1.799506258	1.295079639
ENSMUSG00000064168.4	#N/A	-0.9337	1.9607	-2.84082	0.012563	0.121898	-2.86021	2.552565114	3.004513725	1.76443046	2.002957898	0.97638402	1.463351122
ENSMUSG00000020811.16	Wscd1	-0.93323	1.422862	-3.23439	0.005665	0.078512	-2.08428	1.754753965	1.752450437	2.06056255	1.367114228	0.97638402	0.625904135
ENSMUSG00000106847.1	Peg13	-0.93283	2.862774	-4.57346	0.000382	0.018298	0.277911	3.098708366	3.664577681	3.20884256	2.71106863	2.266899163	2.226548258
ENSMUSG00000039713.16	Plekhg5	-0.9325	1.143673	-2.59516	0.02051	0.158008	-3.17084	1.089938156	1.803980737	1.83568914	1.017171757	1.062540664	0.052718802
ENSMUSG00000025092.6	Hspa12a	-0.93129	1.604179	-2.57881	0.021184	0.160328	-3.26462	1.565276166	3.04790632	1.6894684	1.322026339	0.735375921	1.265022405
ENSMUSG00000068748.7	Ptprz1	-0.93124	2.148442	-2.82118	0.013069	0.124906	-2.92885	2.698170436	2.95977541	2.11886119	2.263609653	1.965243463	0.884991356
ENSMUSG00000032340.7	Neo1	-0.92983	4.107276	-4.86814	0.000215	0.014141	0.672602	4.624169855	4.709182983	4.34023296	4.006524513	3.821294947	3.142250023
ENSMUSG00000009681.9	Bcr	-0.9291	3.280802	-3.45626	0.003606	0.059306	-1.94276	3.782234701	4.359108008	3.1117014	3.233489664	2.754916107	2.443359647
ENSMUSG00000031119.4	Gpc4	-0.92907	1.07713	-2.72989	0.01569	0.138972	-2.93594	1.089938156	2.160915282	1.39138705	0.77101117	0.681936662	0.367592139
ENSMUSG00000028545.13	Bend5	-0.9284	0.263964	-2.47361	0.026056	0.176497	-3.2948	0.776780271	0.725978225	0.67010308	-0.011397395	-0.402127603	-0.175550186
ENSMUSG00000042763.9	Maneal	-0.9279	0.388004	-2.20792	0.043531	0.213843	-3.70604	1.158650906	0.925287033	0.41484602	0.70229842	-0.522421837	-0.350636892
ENSMUSG00000044060.17	#N/A	-0.92761	0.634048	-2.60686	0.02004	0.157078	-3.10454	0.941839517	0.925287033	1.34329276	0.389140535	0.380280962	-0.175550186
ENSMUSG00000024968.13	Rcor2	-0.9242	4.103806	-4.37859	0.000561	0.021658	-0.27611	4.706800741	4.880983807	4.09364491	4.006524513	3.712239422	3.222643803
ENSMUSG00000032394.5	Igdcc3	-0.92413	3.683224	-3.21548	0.005887	0.080014	-2.49679	4.648265531	4.010009712	3.68465139	4.096290474	2.830533154	2.829593117
ENSMUSG00000028414.16	Fktn	-0.92378	2.203887	-4.26802	0.000699	0.024305	-0.23987	2.602750871	2.685336241	2.67981823	2.002957898	1.523871816	1.728589799
ENSMUSG00000027792.11	Bche	-0.92226	0.294149	-2.23681	0.041199	0.210715	-3.65478	0.487273654	1.256492942	0.50504383	0.389140535	-0.522421837	-0.350636892
ENSMUSG00000098912.7	1500004A13Ri	-0.92221	2.054953	-3.3063	0.004894	0.072321	-2.01886	2.651249389	2.160915282	2.62052105	2.263609653	1.143840766	1.489582663
ENSMUSG00000099250.1	Rn7s2	-0.92196	2.327803	-2.907	0.010996	0.112128	-2.8043	2.721070838	3.110642075	2.47187697	2.239564179	2.388644435	1.0350168
ENSMUSG00000026779.6	Mastl	-0.92184	1.670725	-2.93519	0.010389	0.108772	-2.64692	2.072236155	2.037179913	2.14715024	2.086213402	0.836659257	0.844913917
ENSMUSG00000030346.16	Rad51ap1	-0.92174	1.249098	-2.98931	0.009313	0.101934	-2.50433	2.175329648	1.462943819	1.48301752	0.77101117	0.97638402	0.625904135
ENSMUSG00000027326.13	Knl1	-0.92172	2.649882	-3.20605	0.006001	0.080764	-2.30287	3.150238667	3.302007601	2.79156659	3.092939265	1.748432074	1.81410787
ENSMUSG00000050503.8	Fbxl22	-0.92125	0.699416	-2.65265	0.0183	0.150932	-3.03432	1.286975003	1.10037374	1.07535956	0.554199781	-0.188002798	0.367592139
ENSMUSG00000024304.14	Cdh2	-0.91929	4.051201	-5.01328	0.000163	0.012385	0.956556	4.542957786	4.794457963	4.17832613	4.006524513	3.39280806	3.392134323
ENSMUSG00000061393.13	Acvr2b	-0.91921	2.64574	-3.63505	0.002506	0.047638	-1.48156	3.232232039	3.422301835	2.6405588	2.69347457	2.030831804	1.855038134
ENSMUSG00000037989.15	Wnk2	-0.91912	2.596082	-3.26538	0.005319	0.075683	-2.17972	2.989773994	3.745877783	2.49407271	2.164925378	2.388644435	1.79319888
ENSMUSG00000056602.11	Fry	-0.919	2.109657	-3.0353	0.008486	0.097322	-2.52888	2.240180792	3.319818877	2.25506558	1.275483753	2.05204829	1.51534576
ENSMUSG00000022856.8	Tmem41a	-0.91783	0.856132	-2.58265	0.021024	0.159596	-3.16091	1.347095996	1.525679575	1.13307505	0.474029432	-0.188002798	0.844913917
ENSMUSG00000003779.15	Kif20a	-0.91636	2.50886	-3.47743	0.003454	0.058008	-1.76179	2.809201749	3.026373159	2.9604956	2.844592302	1.640516735	1.771982394
ENSMUSG00000027167.11	Elp4	-0.91613	1.318471	-3.27886	0.005175	0.074585	-1.99839	1.922210711	1.803980737	1.61039683	0.836599512	1.020105398	0.71753461
ENSMUSG00000038916.6	Soga3	-0.91592	2.561585	-3.55046	0.002977	0.053262	-1.63046	3.008510557	3.405718951	2.62052105	2.675663294	1.824380927	1.83471815
ENSMUSG00000029563.16	Foxp2	-0.91592	2.912948	-4.05188	0.001078	0.030547	-0.7207	3.369010772	3.47093661	3.20884256	2.812351965	2.702209057	1.914335318
ENSMUSG00000007080.14	Pole	-0.91572	3.394225	-4.55764	0.000394	0.018566	0.180132	3.877044395	3.84760767	3.75066856	3.416208778	3.003864757	2.469953857
ENSMUSG00000071847.12	Apcdd1	-0.91527	2.154282	-2.93505	0.010392	0.108772	-2.72139	2.500570476	3.170763068	2.17489523	2.3105307	1.062540664	1.706394053
ENSMUSG00000036834.16	Plch1	-0.91519	2.127282	-3.50107	0.003292	0.056198	-1.66421	2.721070838	2.346130154	2.57959078	2.3105307	1.396959703	1.409412315
ENSMUSG00000074024.5	4632427E13Ri	-0.91512	2.831294	-3.21398	0.005905	0.080014	-2.32246	3.425873109	3.090031796	3.19535933	3.293113647	2.21140405	1.771982394
ENSMUSG00000078919.10	Dpm1	-0.91489	1.231826	-3.01505	0.008841	0.099056	-2.45782	1.663123489	1.948370647	1.5267389	0.70229842	0.445869304	1.104557733
ENSMUSG00000031398.13	Plxna3	-0.91445	3.662985	-3.05162	0.00821	0.095879	-2.81095	4.146014081	4.529511436	3.66522086	3.372652412	3.875857144	2.388653755
ENSMUSG00000029406.15	Pitpnm2	-0.91398	1.821082	-3.04534	0.008315	0.096751	-2.46803	2.175329648	2.600447343	2.03050532	1.534570974	1.824380927	0.761255988
ENSMUSG00000095123.1	Gm21781	-0.91397	1.320248	-3.18102	0.006314	0.08304	-2.17211	1.615029201	1.901828061	1.76443046	1.17763643	0.884753545	0.577809846
ENSMUSG00000048332.13	Lhfp	-0.91348	0.69275	-2.46929	0.026277	0.176849	-3.32978	1.224239248	1.643516065	0.67010308	-0.011397395	0.508605059	0.121431552
ENSMUSG00000006800.13	Sulf2	-0.91312	4.298652	-4.39118	0.000547	0.021597	-0.27868	4.811870936	5.308712625	4.20548355	3.775198967	3.745179096	3.945465215
ENSMUSG00000041633.14	Kctd12b	-0.91268	0.747573	-2.34824	0.033258	0.196152	-3.52749	1.798475342	0.614946913	1.13307505	0.298942726	0.735375921	-0.095379837
ENSMUSG00000029122.11	Evc	-0.91196	2.425759	-3.01604	0.008823	0.099056	-2.62003	3.183597264	2.510249534	2.79156659	2.779374643	1.965243463	1.324523417
ENSMUSG00000014932.15	Yes1	-0.91095	1.511488	-2.53759	0.022978	0.167019	-3.32112	1.798475342	1.901828061	2.03050532	1.787689911	1.363407143	0.187019893
ENSMUSG00000033985.17	Tesk2	-0.91094	0.421521	-2.499	0.02479	0.172857	-3.26335	0.861669169	0.614946913	1.13307505	-0.131691629	-0.001589673	0.052718802
ENSMUSG00000042363.14	Lgalsl	-0.90977	1.069049	-2.64715	0.018501	0.152003	-3.07486	1.615029201	1.948370647	1.01523856	0.95945626	0.626441549	0.249755649
ENSMUSG00000039835.15	Nhsl1	-0.90928	2.889527	-3.79327	0.001818	0.039791	-1.21565	3.216201929	3.883980059	2.94446549	2.71106863	2.485397668	2.096046233
ENSMUSG00000032783.8	Troap	-0.9091	1.045983	-2.35156	0.033045	0.195344	-3.55951	1.798475342	1.585800567	1.07535956	0.77101117	1.220802748	-0.175550186
ENSMUSG00000028246.13	Faxc	-0.90894	2.602836	-3.5412	0.003033	0.05369	-1.6545	2.892257488	3.622142414	2.67981823	2.54441876	1.799506258	2.07887109
ENSMUSG00000041911.3	Dlx1	-0.90789	1.394562	-2.59969	0.020327	0.158008	-3.19666	1.754753965	2.237877264	1.5267389	1.684596418	0.239418426	0.923985488
ENSMUSG00000021356.9	Irf4	-0.90769	0.541057	-2.42852	0.028456	0.182618	-3.38178	1.460306606	0.829071718	0.67010308	0.298942726	0.163469573	-0.175550186
ENSMUSG00000028195.4	Cyr61	-0.90744	2.428486	-3.11235	0.007259	0.090174	-2.43968	2.830418235	3.354795016	2.47187697	2.031246943	2.500910667	1.381667324
ENSMUSG00000033906.5	Zdhhc15	-0.90713	1.057343	-2.34114	0.033717	0.197061	-3.57806	1.158650906	1.699011178	1.5267389	1.453270872	0.681936662	-0.175550186
ENSMUSG00000105572.1	Gm43300	-0.9064	0.912635	-2.58643	0.020867	0.159099	-3.16012	1.615029201	1.180544089	1.29353973	0.202727411	1.062540664	0.121431552
ENSMUSG00000022840.8	Adcy5	-0.90563	1.115107	-2.63775	0.01885	0.152835	-3.09576	1.513745865	1.993458536	1.18857017	1.227389465	0.239418426	0.528056811
ENSMUSG00000028134.11	Ptbp2	-0.90492	4.653931	-6.31498	1.50E-05	0.005308	3.266546	4.934509976	5.258803103	5.1027014	4.507137744	4.156140031	3.964293476
ENSMUSG00000025507.13	Pidd1	-0.90479	1.676673	-3.29607	0.004997	0.072879	-1.99435	1.754753965	2.414034219	2.17489523	1.573565106	1.143840766	0.998947545
ENSMUSG00000059708.12	Akap17b	-0.90433	0.947761	-2.65673	0.018152	0.150268	-3.04678	1.565276166	1.803980737	0.88691447	0.554199781	0.508605059	0.367592139
ENSMUSG00000012609.18	Ttll5	-0.90424	2.559764	-3.95355	0.001314	0.033365	-0.86116	3.008510557	3.283973678	2.71803755	2.463687488	2.133925297	1.750449233
ENSMUSG00000019889.9	Ptprk	-0.90386	2.166862	-2.68134	0.017285	0.14661	-3.18518	2.892257488	2.936875008	2.03050532	1.787689911	2.354895644	0.998947545
ENSMUSG00000028532.14	Cachd1	-0.90346	3.632677	-4.16118	0.000866	0.027591	-0.62639	4.179469378	4.279429753	3.74142048	3.715433822	3.168335328	2.711975085
ENSMUSG00000036377.18	C530008M17R	-0.90287	3.930859	-3.79599	0.001808	0.039736	-1.39853	4.683670867	4.729313938	3.76898874	3.458488581	3.82746032	3.117231563
ENSMUSG00000100210.1	Hist1h3f	-0.90123	2.847692	-3.96399	0.001287	0.03302	-0.88	3.354437971	3.705800343	2.86152915	2.69347457	2.153688552	2.317218617
ENSMUSG00000050064.14	Zfp697	-0.90037	1.297127	-2.4481	0.027389	0.180155	-3.43808	1.961204842	1.699011178	1.56917417	1.410835606	-0.091787482	1.234325608
ENSMUSG00000036766.12	Dner	-0.90012	1.18839	-2.68009	0.017328	0.14661	-3.0322	1.286975003	2.346130154	1.39138705	0.474029432	0.786906222	0.844913917
ENSMUSG00000001300.15	Efnb2	-0.8995	3.472211	-3.50658	0.003255	0.055998	-1.88317	3.927143006	4.324237549	3.46627426	3.671175903	2.948369644	2.496066697
ENSMUSG00000028402.17	Mpdz	-0.8984	3.56223	-4.61221	0.000354	0.018054	0.259833	3.985051584	4.010009712	3.95650474	3.616209443	3.13889155	2.666710351
ENSMUSG00000026043.18	Col3a1	-0.89831	5.523096	-5.55101	5.91E-05	0.008819	1.851333	5.699612411	6.38366057	5.83242636	5.317726201	4.889181257	5.015967346
ENSMUSG00000000142.15	Axin2	-0.89808	3.548397	-3.81325	0.001746	0.038925	-1.2948	3.917261495	4.242553751	3.75985773	3.662159029	3.225479235	2.483069357
ENSMUSG00000084883.1	Ccdc85c	-0.89708	1.474431	-2.36062	0.03247	0.19391	-3.61262	2.208119583	2.629302206	1.01523856	1.17763643	1.143840766	0.672446721
ENSMUSG00000053110.13	Yap1	-0.89681	3.690203	-4.08301	0.001013	0.030012	-0.78965	4.313586734	4.315386631	3.74142048	3.732763701	3.271428821	2.76662916
ENSMUSG00000022604.18	Cep97	-0.89681	2.015026	-3.09879	0.007461	0.091098	-2.39789	2.30224176	2.571003565	2.40316422	2.239564179	1.61222769	0.961953338
ENSMUSG00000041245.13	Wnk3	-0.89675	2.547147	-4.22538	0.000761	0.025525	-0.34526	2.912295242	3.228478566	2.82697193	2.3105307	2.21140405	1.79319888
ENSMUSG00000041120.6	Nbl1	-0.8952	1.011412	-2.76988	0.014484	0.132398	-2.86236	1.286975003	1.525679575	1.56917417	0.298942726	1.020105398	0.367592139
ENSMUSG00000025427.14	Rnf165	-0.89417	3.047964	-3.52641	0.003126	0.05441	-1.76084	3.715379726	3.907728862	2.87850223	2.952076729	2.546473245	2.287623834
ENSMUSG00000061410.6	Zcchc14	-0.89399	3.703166	-3.88397	0.001513	0.036056	-1.18383	4.275417972	4.537144761	3.62555664	3.732763701	3.12894189	2.919168471
ENSMUSG00000036882.6	Arhgap33	-0.89358	3.0794	-3.04896	0.008254	0.096313	-2.68892	3.339716445	4.165847577	3.1117014	2.54441876	3.253224226	2.061489011
ENSMUSG00000021796.14	Bmpr1a	-0.89228	3.705758	-4.59114	0.000369	0.01826	0.197778	4.336012497	4.270298741	3.81379654	3.653085446	3.271428821	2.889923652
ENSMUSG00000022883.10	Robo1	-0.89207	4.536846	-4.6257	0.000345	0.017993	0.151162	5.173255319	5.281703505	4.50230886	4.187096759	4.318150862	3.758561878
ENSMUSG00000061576.15	Dpp6	-0.89169	0.307997	-2.21487	0.042959	0.212526	-3.68841	0.590367147	1.256492942	0.50504383	-0.567790743	0.239418426	-0.175550186
ENSMUSG00000047749.2	Zc3hav1l	-0.89165	2.7138	-3.68891	0.002246	0.044897	-1.3888	2.951554672	3.578421037	2.9604956	2.657629371	1.77419517	2.360502714
ENSMUSG00000021572.8	Cep72	-0.89061	0.944946	-2.42909	0.028424	0.182501	-3.42114	1.565276166	1.015484842	1.43792963	1.17763643	0.568726051	-0.095379837
ENSMUSG00000003452.15	Bicd1	-0.89046	2.107015	-3.18418	0.006273	0.082773	-2.25322	2.552565114	2.913605228	2.17489523	2.239564179	1.220802748	1.540656848
ENSMUSG00000001802.16	Lrp3	-0.89008	2.423943	-2.95704	0.009941	0.105823	-2.72938	2.787668587	3.502472184	2.33101443	2.563914936	1.523871816	1.83471815
ENSMUSG00000023473.11	Celsr3	-0.88938	4.056352	-3.1643	0.006532	0.08485	-2.67017	4.252022405	5.045232186	4.2255213	3.468867727	4.361283972	2.985185644
ENSMUSG00000044949.4	Ubtd2	-0.88762	2.049328	-3.82765	0.001695	0.038362	-1.0463	2.271544963	2.712557709	2.51593215	1.648527164	1.396959703	1.750449233
ENSMUSG00000056486.17	Chn1	-0.88755	1.376394	-2.8924	0.011325	0.113949	-2.68646	2.271544963	1.901828061	1.34329276	0.836599512	1.020105398	0.884991356
ENSMUSG00000020623.11	Map2k6	-0.88722	1.691797	-2.90571	0.011025	0.112165	-2.7027	2.418886679	1.803980737	2.06056255	1.974103035	0.931296131	0.961953338
ENSMUSG00000028145.9	Them4	-0.88706	0.854822	-2.61152	0.019856	0.155907	-3.11343	0.941839517	1.699011178	1.29353973	0.202727411	0.681936662	0.309876641
ENSMUSG00000046312.4	AI464131	-0.88634	0.422218	-2.21741	0.042752	0.212429	-3.69517	0.255948108	1.10037374	1.18857017	0.099633918	0.239418426	-0.350636892
ENSMUSG00000052727.5	Map1b	-0.88619	6.623697	-4.31491	0.000637	0.023329	-0.53594	7.072792643	7.526159114	6.59956143	6.384350933	6.41498397	5.74433467
ENSMUSG00000026640.12	Plxna2	-0.88612	3.39534	-5.51462	6.33E-05	0.009128	1.959555	3.803848514	4.063847864	3.66522086	2.952076729	3.067758373	2.819288109
ENSMUSG00000035202.7	Lars2	-0.88607	15.21829	-3.26653	0.005306	0.075586	-2.39435	15.76917015	16.04102424	15.1789535	15.23852638	15.05540334	14.02663706
ENSMUSG00000025019.15	Lcor	-0.88589	2.990521	-3.97511	0.001258	0.032787	-0.88054	3.133265588	3.883980059	3.26154961	2.937203453	2.469716046	2.257409221
ENSMUSG00000055884.7	Fancm	-0.88577	2.620354	-4.0013	0.001194	0.031844	-0.7779	3.027006901	3.13096206	2.9604956	2.675663294	2.113887544	1.81410787
ENSMUSG00000020598.15	Nrcam	-0.88565	2.881341	-3.53376	0.00308	0.054094	-1.71599	3.279280612	3.883980059	2.82697193	2.779374643	2.388644435	2.129795025
ENSMUSG00000020176.17	Grb10	-0.88537	6.145507	-4.23737	0.000743	0.025379	-0.69459	6.641529199	6.902566957	6.21803972	6.178427164	5.760565829	5.171913001
ENSMUSG00000097504.1	4930516B21Ri	-0.88515	1.272549	-2.62036	0.019511	0.154831	-3.14445	1.513745865	2.120837843	1.5267389	1.275483753	0.163469573	1.0350168
ENSMUSG00000075478.2	Slitrk1	-0.88511	0.564979	-2.23864	0.041056	0.210441	-3.67762	0.776780271	0.614946913	1.48301752	0.554199781	0.311568212	-0.350636892
ENSMUSG00000022656.15	Nectin3	-0.88363	2.580909	-2.91324	0.010859	0.11166	-2.84177	2.932058497	3.265711472	2.75527046	3.038233373	2.030831804	1.463351122
ENSMUSG00000087138.1	Gm15545	-0.88342	0.084795	-2.23329	0.041478	0.211469	-3.64484	0.487273654	0.725978225	0.41484602	-0.567790743	-0.29109629	-0.260439084
ENSMUSG00000087396.7	4933407K13Ri	-0.88315	0.946258	-2.74795	0.015134	0.13593	-2.89408	1.513745865	1.462943819	1.18857017	0.77101117	0.163469573	0.577809846
ENSMUSG00000056592.14	Zfp658	-0.88241	0.570034	-2.30871	0.035894	0.201956	-3.57062	0.590367147	1.643516065	0.81820172	0.389140535	-0.001589673	-0.019430984
ENSMUSG00000049321.17	Zfp2	-0.88218	0.704828	-2.14041	0.049467	0.222451	-3.84319	1.565276166	1.328642728	0.50504383	1.017171757	-0.091787482	-0.095379837
ENSMUSG00000051367.8	Six1	-0.88207	2.432226	-3.39454	0.004089	0.064176	-1.90742	2.970790891	3.170763068	2.4493344	2.54441876	1.77419517	1.683851484
ENSMUSG00000042804.13	Gpr153	-0.88125	0.824366	-2.49233	0.025117	0.173779	-3.30523	1.565276166	1.328642728	0.95250281	0.474029432	-0.091787482	0.71753461

ENSMUSG00000043587.15	Pxylp1	-0.88097	0.697719	-2.23391	0.041429	0.211469	-3.6995	1.158650906	1.803980737	0.58993273	0.099633918	-0.091787482	0.625904135
ENSMUSG00000036334.12	Igsf10	-0.88091	1.289834	-2.36391	0.032264	0.193369	-3.57588	1.798475342	2.571003565	0.95250281	0.77101117	0.884753545	0.761255988
ENSMUSG00000022098.9	Bmp1	-0.87957	3.176557	-5.46172	6.98E-05	0.009244	1.881593	3.584135193	3.678449855	3.56393752	2.90698884	2.741918767	2.583910046
ENSMUSG00000037007.17	Zfp113	-0.87923	1.959162	-3.226	0.005762	0.079222	-2.15526	2.552565114	2.160915282	2.37951295	1.914602024	1.748432074	0.998947545
ENSMUSG00000087143.9	A830082K12Ri	-0.87896	1.612199	-2.47557	0.025956	0.176177	-3.44408	1.882133271	2.07961518	2.03050532	1.974103035	1.396959703	0.309876641
ENSMUSG00000102748.1	Pcdhgb2	-0.87855	0.660679	-2.16027	0.047647	0.21983	-3.80755	0.590367147	1.699011178	0.95250281	0.899335267	0.083299224	-0.260439084
ENSMUSG00000024548.11	Setbp1	-0.87855	4.126445	-3.37463	0.004258	0.065754	-2.26876	4.510463281	4.781662358	4.38842767	3.653085446	4.439845516	2.985185644
ENSMUSG00000071252.5	2210408I21Ril	-0.8782	0.892248	-2.64325	0.018645	0.152281	-3.06436	1.089938156	1.643516065	1.24200942	0.630148634	0.626441549	0.121431552
ENSMUSG00000032782.19	Cntrob	-0.8779	2.471098	-3.10287	0.007399	0.090735	-2.46549	2.809201749	3.170763068	2.66032206	2.71106863	2.09356756	1.381667324
ENSMUSG00000104882.1	Gm43096	-0.87789	0.159695	-2.1533	0.048279	0.220594	-3.76615	0.861669169	0.494652679	0.50504383	-0.567790743	-0.522421837	0.187019893
ENSMUSG00000031790.7	Mmp15	-0.8777	1.872591	-2.75678	0.014869	0.134268	-2.99676	1.999172692	3.170763068	1.83568914	1.611532956	1.29386621	1.324523417
ENSMUSG00000035062.13	Zc4h2	-0.87716	1.502811	-3.09367	0.007539	0.091676	-2.34293	2.208119583	2.07961518	1.56917417	1.017171757	1.257796956	0.884991356
ENSMUSG00000030849.17	Fgfr2	-0.87697	3.531763	-4.61352	0.000353	0.018054	0.26689	3.994480997	4.010009712	3.848667	3.416208778	3.289406562	2.631804736
ENSMUSG00000038486.9	Sv2a	-0.87602	0.556069	-2.15473	0.048149	0.220397	-3.80317	0.776780271	1.397355478	0.81820172	0.630148634	-0.65366637	0.367592139
ENSMUSG00000045348.15	Nyap1	-0.87529	1.475662	-2.62643	0.019278	0.154133	-3.16276	1.404811494	2.65759125	1.6894684	1.322026339	1.062540664	0.71753461
ENSMUSG00000020546.14	Stxbp4	-0.87341	2.651739	-3.7337	0.002051	0.042346	-1.29364	2.932058497	3.228478566	3.02289214	2.76259893	2.192420947	1.771982394
ENSMUSG00000019773.7	Fbxo5	-0.87303	1.500241	-2.88874	0.011408	0.114674	-2.70761	2.107425583	2.037179913	1.61039683	1.648527164	0.836659257	0.761255988
ENSMUSG00000023391.8	Dlx2	-0.8722	0.628193	-2.29634	0.036759	0.202822	-3.59567	1.404811494	1.180544089	0.58993273	0.474029432	0.380280962	-0.260439084
ENSMUSG00000032216.14	Nedd4	-0.87218	6.8132	-4.83131	0.000231	0.014822	0.48243	7.267175363	7.558399451	6.92366312	6.803665622	6.269731865	6.05656233
ENSMUSG00000005672.12	Kit	-0.87149	1.677627	-3.374	0.004264	0.065762	-1.85236	2.30224176	2.274871471	1.80049971	1.227389465	1.257796956	1.202961437
ENSMUSG00000032558.18	Nphp3	-0.87072	1.39733	-2.68027	0.017322	0.14661	-3.05873	2.0361669	1.803980737	1.61039683	1.07266687	1.493175018	0.367592139
ENSMUSG00000025608.9	Podxl	-0.8703	1.361124	-3.01872	0.008775	0.098962	-2.46261	1.460306606	1.948370647	1.96844435	1.07266687	0.681936662	1.0350168
ENSMUSG00000016200.13	Syt14	-0.87025	1.21901	-2.44417	0.0276	0.180155	-3.43289	1.663123489	2.07961518	1.24200942	1.322026339	0.083299224	0.923985488
ENSMUSG00000021782.14	Dlg5	-0.86904	3.743928	-2.98861	0.009326	0.101996	-2.95027	4.146014081	4.625726752	3.71331514	3.824526679	3.657919781	2.496066697
ENSMUSG00000038248.8	Sobp	-0.86877	1.27738	-2.42126	0.028862	0.184162	-3.47956	1.798475342	2.380481659	1.01523856	1.017171757	0.97638402	0.476526511
ENSMUSG00000031169.13	Porcn	-0.86845	0.543123	-2.17579	0.046269	0.217853	-3.77005	0.941839517	1.180544089	0.88691447	-0.567790743	0.836659257	-0.019430984
ENSMUSG00000003436.11	Dll3	-0.86814	2.291031	-3.37173	0.004283	0.065991	-1.92892	2.851327224	3.209495462	2.2021167	1.820479846	1.748432074	1.914335318
ENSMUSG00000107000.1	Gm43481	-0.86681	1.115425	-2.46113	0.0267	0.177985	-3.39042	1.513745865	1.752450437	1.34329276	1.367114228	-0.001589673	0.71753461
ENSMUSG00000026235.14	Epha4	-0.86658	3.727229	-4.0226	0.001144	0.03138	-0.91524	4.372631088	3.871957531	4.1295347	3.864382668	3.315959981	2.808908963
ENSMUSG00000025153.9	Fasn	-0.86636	5.492131	-4.3845	0.000555	0.021658	-0.39146	5.888469228	6.35371475	5.52808407	5.505878298	4.897996122	4.778646124
ENSMUSG00000068699.12	Flnc	-0.86541	2.311041	-3.09642	0.007497	0.091373	-2.44942	2.141777088	3.283973678	2.77353267	2.3105307	1.695483194	1.660951082
ENSMUSG00000027966.19	Col11a1	-0.86452	2.997315	-2.63615	0.018909	0.152876	-3.44584	3.520288711	3.607715344	3.05310675	3.20893256	3.003864757	1.58998456
ENSMUSG00000029836.15	Cbx3	-0.86438	2.883457	-3.99364	0.001212	0.032203	-0.82849	3.439745284	3.486790561	2.99203117	2.90698884	2.248636956	2.226548258
ENSMUSG00000088789.1	Scarna13	-0.86403	1.60006	-2.55203	0.022334	0.164957	-3.31079	1.286975003	2.739275048	2.06056255	1.410835606	1.257796956	0.844913917
ENSMUSG00000104369.1	Gm38082	-0.86367	0.558065	-2.49961	0.024761	0.172784	-3.27103	0.941839517	1.180544089	0.88691447	-0.011397395	0.163469573	0.187019893
ENSMUSG00000027583.13	Zbtb46	-0.8635	1.429306	-2.67178	0.017617	0.148028	-3.07788	1.754753965	2.160915282	1.61039683	1.719785847	0.568726051	0.761255988
ENSMUSG00000047146.15	Tet1	-0.86341	2.891236	-3.43627	0.003756	0.060796	-1.90637	3.546167343	3.265711472	3.08270153	2.812351965	2.805765249	1.83471815
ENSMUSG00000013033.16	Adgrl1	-0.86334	4.086819	-3.79557	0.001809	0.039736	-1.42667	4.636267997	4.482843377	4.35849517	4.440143438	3.197190191	3.405973231
ENSMUSG00000022449.14	Adamts20	-0.86261	1.857429	-2.66693	0.017788	0.148904	-3.15448	2.072236155	2.199909414	2.426434	2.378169417	1.143840766	0.923985488
ENSMUSG00000030313.15	Dennd5b	-0.86179	2.703842	-3.75008	0.001984	0.042194	-1.26989	3.232232039	3.405718951	2.77353267	2.657629371	1.896530713	2.257409221
ENSMUSG00000052565.6	Hist1h1d	-0.86096	4.23353	-3.40693	0.003987	0.063019	-2.22283	4.671965366	5.163383538	4.14364429	4.445408754	3.342033493	3.634746011
ENSMUSG00000049532.10	Sall2	-0.85902	2.971374	-3.73791	0.002034	0.042311	-1.33656	3.480579001	3.84760767	2.92825526	2.745625852	2.230140613	2.596033605
ENSMUSG00000037725.7	Ckap2	-0.85896	1.235374	-3.00304	0.009058	0.100453	-2.47929	1.663123489	1.853733772	1.48301752	0.95945626	0.735375921	0.71753461
ENSMUSG00000033849.3	B3galt2	-0.85862	0.722807	-2.35405	0.032886	0.19504	-3.51569	1.347095996	1.328642728	0.74605193	0.77101117	0.239418426	-0.095379837
ENSMUSG00000021965.2	Ska3	-0.85726	0.971709	-2.54821	0.022503	0.165568	-3.22938	1.513745865	1.10037374	1.48301752	1.126106129	0.239418426	0.367592139
ENSMUSG00000027896.15	Slc16a4	-0.85636	0.980977	-2.22492	0.042145	0.212176	-3.75184	1.709666075	1.256492942	1.13307505	1.494493535	0.239418426	0.052718802
ENSMUSG00000030319.8	Cand2	-0.85634	3.179234	-3.75922	0.001948	0.04175	-1.33205	3.533286052	4.084832889	3.22220094	3.145646315	2.388644435	2.700791346
ENSMUSG00000044702.13	Palb2	-0.85634	1.411344	-2.9116	0.010895	0.111729	-2.65686	1.840910608	1.752450437	1.83568914	1.367114228	1.143840766	0.528056811
ENSMUSG00000051323.16	Pcdh19	-0.85626	2.023274	-2.96378	0.009806	0.105187	-2.6471	1.922210711	3.110642075	2.35546747	1.573565106	1.824380927	1.35337828
ENSMUSG00000019846.11	Lama4	-0.85626	1.76365	-2.58169	0.021063	0.159689	-3.28731	1.513745865	2.95977541	2.17489523	1.367114228	0.97638402	1.58998456
ENSMUSG00000038860.15	Garnl3	-0.85548	1.335503	-2.4704	0.02622	0.176849	-3.40699	1.663123489	1.752450437	1.72743625	1.820479846	0.681936662	0.367592139
ENSMUSG00000078716.9	Tmem8b	-0.85517	1.980023	-3.14492	0.006794	0.086582	-2.30762	2.526802018	2.791269685	1.93638314	1.852541056	1.257796956	1.51534576
ENSMUSG00000084911.1	Gm16185	-0.85494	0.5237	-2.21801	0.042703	0.212429	-3.70454	0.941839517	0.925287033	0.88691447	0.836599512	-0.188002798	-0.260439084
ENSMUSG00000051251.3	Nhlh1	-0.85459	2.369614	-3.15446	0.006664	0.085817	-2.3505	2.552565114	3.438696273	2.4493344	2.215111135	1.55392905	2.008049752
ENSMUSG00000071226.11	Cecr2	-0.85418	3.778052	-3.05376	0.008174	0.095707	-2.83161	4.422384123	3.919457955	4.14364429	3.94837276	3.725505677	2.508947988
ENSMUSG00000030499.9	Kctd15	-0.8539	2.272338	-3.30272	0.004929	0.07261	-2.05638	2.651249389	3.069122806	2.35546747	2.355973671	1.61222769	1.58998456
ENSMUSG00000001036.17	Epn2	-0.85308	1.886364	-3.00525	0.009017	0.100253	-2.55044	2.418886679	2.685336241	1.83568914	1.754137352	1.55392905	1.070206228
ENSMUSG00000054115.11	Skp2	-0.85277	2.970502	-4.13251	0.000917	0.028546	-0.57414	3.150238667	3.692189912	3.33716666	2.891640875	2.192420947	2.559352942
ENSMUSG00000039601.15	Rcan2	-0.85265	1.38033	-3.01611	0.008822	0.099056	-2.469	1.615029201	2.160915282	1.6894684	0.95945626	0.786906222	1.070206228
ENSMUSG00000075028.11	Prdm11	-0.85226	2.205647	-3.42314	0.003858	0.06192	-1.82048	2.577876203	2.600447343	2.66032206	2.287260921	1.29386621	1.81410787
ENSMUSG00000021071.15	Trim9	-0.85076	1.351653	-3.01964	0.008759	0.098935	-2.46017	1.840910608	1.803980737	1.65047427	1.275483753	0.735375921	0.803691254
ENSMUSG00000056758.14	Hmga2	-0.85067	4.514102	-5.78914	3.83E-05	0.008043	2.344859	4.94913201	5.216643429	4.6578664	4.272524377	3.973606936	4.014838918
ENSMUSG00000024620.11	Pdgfrb	-0.8499	2.764422	-3.82111	0.001718	0.038617	-1.14239	3.183597264	3.63642664	2.844354	2.031246943	2.516258632	2.374646897
ENSMUSG00000019831.14	Wasf1	-0.84694	1.925868	-2.67324	0.017566	0.147784	-3.15535	2.602750871	2.510249534	1.87004065	2.058991933	1.668261725	0.844913917
ENSMUSG00000028218.19	Fam92a	-0.84617	1.475529	-2.26079	0.039354	0.208097	-3.7778	2.208119583	1.752450437	1.65047427	1.974103035	0.163469573	1.104557733
ENSMUSG00000039410.16	Prdm16	-0.846	1.763425	-3.0785	0.007774	0.093146	-2.40014	2.208119583	2.629302206	1.76443046	1.410835606	1.429749638	1.138110293
ENSMUSG00000049119.14	Fam110b	-0.84524	1.162121	-2.70505	0.016487	0.142645	-2.98715	1.615029201	1.699011178	1.39138705	1.07266687	0.884753545	0.309876641
ENSMUSG00000018537.16	Pcgf2	-0.84492	2.436496	-3.85037	0.001619	0.037269	-1.04234	2.892257488	2.913605228	2.77353267	1.883905227	2.405227319	1.750449233
ENSMUSG00000055254.14	Ntrk2	-0.84479	1.524854	-2.55073	0.022391	0.16509	-3.30067	2.0361669	1.948370647	1.87004065	0.70229842	1.919800492	0.672446721
ENSMUSG00000091277.1	Gm1818	-0.84452	0.511248	-2.16552	0.047177	0.219053	-3.78203	1.017788371	1.10037374	0.67010308	0.099633918	0.626441549	-0.446852208
ENSMUSG00000001120.15	Pcbp3	-0.84422	1.218579	-2.29339	0.036968	0.203267	-3.67981	1.565276166	2.037179913	1.24200942	1.648527164	0.568726051	0.249755649
ENSMUSG00000055202.10	Zfp811	-0.84355	1.759561	-3.12013	0.007145	0.089222	-2.32404	2.271544963	2.380481659	1.87004065	1.611532956	1.461810848	0.961953338
ENSMUSG00000020282.18	Rhbdf1	-0.84077	1.588665	-2.95695	0.009942	0.105823	-2.59733	1.663123489	2.237877264	2.09000633	1.275483753	1.461810848	0.803691254
ENSMUSG00000039270.9	Megf9	-0.84019	2.691994	-3.75749	0.001955	0.04175	-1.25391	3.199991702	3.486790561	2.67981823	2.421562012	2.284933085	2.07887109
ENSMUSG00000025094.7	Slc18a2	-0.83881	0.983989	-2.52562	0.023526	0.168973	-3.26786	1.286975003	1.901828061	1.07535956	0.70229842	0.311568212	0.625904135
ENSMUSG00000075592.9	Nynrin	-0.83782	2.547367	-2.58651	0.020864	0.159099	-3.43427	3.081114306	3.63642664	2.2021167	2.54441876	2.230140613	1.58998456
ENSMUSG00000004872.15	Pax3	-0.83669	2.595292	-2.94171	0.010253	0.107825	-2.79153	3.045269107	3.170763068	2.73677412	2.728450708	2.453862094	1.436633784
ENSMUSG00000054256.11	Msi1	-0.83654	4.643894	-4.04241	0.001099	0.030728	-1.01248	5.238197706	5.244886322	4.67739557	4.650808105	4.348478902	3.703595419
ENSMUSG00000050714.9	Zbtb26	-0.83631	2.035819	-3.38482	0.004171	0.065001	-1.87019	2.526802018	2.739275048	2.11886119	1.852541056	1.363407143	1.614030034
ENSMUSG00000029710.15	Ephb4	-0.83609	3.203667	-3.35847	0.004401	0.067181	-2.11834	3.621129401	4.010009712	3.19535933	3.416208778	2.590640828	2.388653755
ENSMUSG00000053693.16	Mast1	-0.83588	0.858277	-2.13546	0.049931	0.222753	-3.87217	0.686582462	1.901828061	1.18857017	0.95945626	0.508605059	-0.095379837
ENSMUSG00000034109.15	Golim4	-0.83545	3.148228	-4.99911	0.000167	0.012548	1.042149	3.715379726	3.502472184	3.45500315	2.937203453	2.647503165	2.631804736
ENSMUSG00000053702.16	Nebl	-0.83538	1.188403	-2.41041	0.029478	0.186147	-3.48423	1.347095996	2.237877264	1.24200942	1.017171757	0.97638402	0.309876641
ENSMUSG00000071176.10	Arhgef10	-0.83514	1.929886	-3.10638	0.007347	0.09053	-2.37174	2.418886679	2.380481659	2.17489523	1.883905227	1.722200532	0.998947545
ENSMUSG00000004231.15	Pax2	-0.83458	1.078298	-2.149	0.048672	0.220972	-3.88565	1.017788371	2.274871471	1.18857017	0.95945626	0.97638402	0.052718802
ENSMUSG00000056900.13	Usp13	-0.83453	1.921522	-3.4495	0.003656	0.059903	-1.73713	2.30224176	2.478885363	2.2021167	1.787689911	1.523871816	1.234325608
ENSMUSG00000092341.2	Malat1	-0.83396	9.467767	-4.60824	0.000357	0.018057	0.107334	9.960227687	9.906163667	9.79443401	9.276314985	9.367445805	8.502018459
ENSMUSG00000090100.7	Ttbk2	-0.83365	2.689237	-3.62909	0.002537	0.048082	-1.50009	3.116090444	3.319818877	2.86152915	2.76259893	1.848833971	2.226548258
ENSMUSG00000058392.12	Rrp1b	-0.83332	2.478887	-2.93584	0.010375	0.108772	-2.77961	2.787668587	3.302007601	2.55868179	2.795957527	1.55392905	1.875075887
ENSMUSG00000034751.15	Mast4	-0.83327	3.024826	-4.74434	0.000273	0.015809	0.581004	3.425873109	3.593142542	3.32483586	2.504617752	2.830533154	2.469953857
ENSMUSG00000062785.13	Kcnc3	-0.83286	0.668982	-2.14568	0.048978	0.221591	-3.83051	1.158650906	1.180544089	1.01523856	-0.011397395	-0.29109629	0.961953338
ENSMUSG00000046997.5	Spsb4	-0.83275	2.083393	-2.59636	0.020461	0.158008	-3.32093	2.526802018	3.090031796	1.93638314	2.11293074	1.020105398	1.81410787
ENSMUSG00000022369.13	Mtbp	-0.83266	2.154317	-3.0582	0.008101	0.095272	-2.49463	2.743613407	2.160915282	2.69905445	1.974103035	2.113887544	1.234325608
ENSMUSG00000020534.14	Shmt1	-0.83248	1.647313	-2.947	0.010144	0.107268	-2.62264	2.240180792	2.237877264	1.6894684	1.573565106	1.257796956	0.884991356
ENSMUSG00000007989.6	Fzd3	-0.83223	3.875653	-3.86437	0.001574	0.036668	-1.25395	4.624169855	4.434615096	3.79604007	3.856499069	3.433796471	3.108794726
ENSMUSG00000041757.15	Plekha6	-0.83209	2.950543	-3.49281	0.003348	0.056901	-1.808	3.645275488	3.678449855	2.80937787	2.639367164	2.688725828	2.242061257
ENSMUSG00000057541.14	Pus7	-0.83149	2.121004	-3.57426	0.002836	0.05178	-1.52642	2.332298994	2.841455443	2.426434	1.944659258	1.640516735	1.540656848
ENSMUSG00000063087.14	Gm10125	-0.83142	0.532288	-2.32309	0.034913	0.200218	-3.54499	0.686582462	0.829071718	1.29353973	0.099633918	0.163469573	0.121431552
ENSMUSG00000038156.15	Spon1	-0.83112	2.127039	-2.31181	0.03568	0.20128	-3.82423	2.30224176	3.607715344	1.80049971	1.974103035	1.668261725	1.409412315
ENSMUSG00000005089.15	Slc1a2	-0.82935	4.065401	-4.61286	0.000353	0.018054	0.185922	4.443192585	4.521837508	4.42354572	4.193369356	3.298312115	3.51215042
ENSMUSG00000040003.17	Magi2	-0.82912	0.776525	-2.3066	0.03604	0.202184	-3.59624	1.089938156	1.803980737	0.74605193	0.389140535	0.380280962	0.249755649
ENSMUSG00000022773.16	Ypel1	-0.82898	1.705052	-2.70015	0.016649	0.143489	-3.07007	1.999172692	2.685336241	1.6894684	1.754137352	0.931296131	1.170900228
ENSMUSG00000037032.15	Apbb1	-0.82871	2.315277	-3.6973	0.002209	0.044365	-1.31757	2.851327224	2.841455443	2.51593215	1.787689911	2.21140405	1.683851484
ENSMUSG00000027864.9	Ptgfrn	-0.82789	3.062254	-3.54215	0.003028	0.05369	-1.7331	3.294628576	4.084832889	3.12598563	2.728450708	2.320338422	2.819288109
ENSMUSG00000071856.10	Mcc	-0.82676	1.560159	-2.43759	0.027957	0.181144	-3.49923	2.0361669	2.629302206	1.29353973	1.494493535	1.103763327	0.803691254
ENSMUSG00000025813.13	Homer2	-0.82565	1.200165	-2.32285	0.034929	0.200218	-3.629	1.224239248	2.199909414	1.48301752	0.95945626	0.163469573	1.170900228
ENSMUSG00000020312.11	Shc2	-0.82465	2.31933	-3.61374	0.002617	0.048717	-1.47606	2.851327224	2.865908486	2.47187697	2.002957898	2.133925297	1.58998456
ENSMUSG00000022791.16	Tnk2	-0.82389	2.549276	-3.21543	0.005887	0.080014	-2.2669	2.892257488	3.422301835	2.55868179	2.504617752	2.21140405	1.706394053
ENSMUSG00000026837.15	Col5a1	-0.82217	3.912688	-5.37306	8.23E-05	0.009818	1.646542	4.259863158	4.58211552	4.1295347	3.724124782	3.367644136	3.41284322
ENSMUSG00000031740.7	Mmp2	-0.82215	2.342793	-3.66669	0.00235	0.045973	-1.3788	3.008510557	2.865908486	2.4493344	1.820479846	1.799506258	2.113019312
ENSMUSG00000085334.7	Gm12940	-0.82203	2.020282	-3.01594	0.008825	0.099056	-2.55124	2.446631669	2.510249534	2.28082867	2.239564179	1.103763327	1.540656848
ENSMUSG00000099583.1	Hist1h3d	-0.82072	2.599556	-2.19538	0.044581	0.214964	-4.12961	3.24808599	3.719283573	2.09000633	2.966798235	1.583372827	1.989787546
ENSMUSG00000064125.14	Prr36	-0.81994	1.921674	-2.80424	0.01352	0.126608	-2.92027	2.602750871	2.685336241	1.72743625	1.787689911	1.523871816	1.202961437
ENSMUSG00000038797.13	Zscan2	-0.81988	1.602246	-3.05212	0.008201	0.095864	-2.42828	2.107425583	2.274871471	1.6894684	1.17763643	1.329055639	1.0350168
ENSMUSG00000037474.13	Dtl	-0.81906	3.259696	-3.95046	0.001323	0.033486	-0.9736	3.645275488	3.719283573	3.56393752	3.350370965	2.890654146	2.388653755
ENSMUSG00000021514.17	Zfp369	-0.81894	2.040694	-3.62779	0.002544	0.048142	-1.41788	2.651249389	2.414034219	2.28082867	1.754137352	1.55392905	1.58998456

ENSMUSG00000026383.14	Epb41l5	-0.81812	2.366039	-3.38268	0.004189	0.06521	-1.91983	2.602750871	3.026373159	2.6405588	2.463687488	1.848833971	1.614030034
ENSMUSG00000045751.16	Mms22l	-0.81677	2.74466	-3.53305	0.003084	0.054102	-1.69391	2.932058497	3.026373159	3.38546221	2.90698884	2.173184728	2.043894951
ENSMUSG00000002416.13	Ndufb2	-0.81646	1.159521	-2.22023	0.042523	0.212347	-3.78724	1.017788371	1.462943819	2.2021167	0.298942726	1.257796956	0.71753461
ENSMUSG00000030309.16	Caprin2	-0.81625	1.709979	-2.34634	0.03338	0.196286	-3.68078	1.922210711	2.160915282	2.17489523	1.494493535	2.030831804	0.476526511
ENSMUSG00000035125.10	Gcfc2	-0.81614	1.91465	-3.33906	0.004578	0.069179	-1.94078	2.240180792	2.237877264	2.4493344	1.787689911	1.182834898	1.58998456
ENSMUSG00000026490.17	Cdc42bpa	-0.81602	3.392738	-4.00834	0.001177	0.031531	-0.88391	3.803848514	3.895903227	3.64552505	3.56874809	2.633496307	2.808908963
ENSMUSG00000054074.9	Skida1	-0.81563	2.522621	-2.67688	0.017439	0.147179	-3.26553	2.989773994	3.319818877	2.4493344	2.463687488	2.531445035	1.381667324
ENSMUSG00000096847.1	Tmem151b	-0.81499	1.387876	-2.21354	0.043068	0.21274	-3.83807	1.460306606	2.346130154	1.5267389	1.852541056	0.380280962	0.761255988
ENSMUSG00000051043.16	Gprc5c	-0.81412	1.373389	-2.86505	0.011966	0.118239	-2.73434	1.798475342	1.948370647	1.56917417	1.275483753	0.931296131	0.71753461
ENSMUSG00000001504.10	Irx2	-0.81357	1.531793	-3.03472	0.008495	0.097353	-2.45162	1.961204842	2.120837843	1.72743625	1.275483753	1.220802748	0.884991356
ENSMUSG00000042156.15	Dzip1	-0.81199	2.843534	-3.19655	0.006118	0.081764	-2.35831	3.150238667	3.337412937	3.16800884	2.891640875	2.741918767	1.771982394
ENSMUSG00000055980.2	Irs1	-0.81089	2.905909	-3.68656	0.002257	0.044897	-1.42512	3.232232039	3.517985183	3.12598563	3.024226515	2.405227319	2.129795025
ENSMUSG00000027004.3	Frzb	-0.81077	1.943766	-2.59718	0.020428	0.158008	-3.29318	2.627203915	2.600447343	1.80049971	2.190236466	1.062540664	1.381667324
ENSMUSG00000026955.13	Sapcd2	-0.8103	0.798908	-2.24622	0.040466	0.209437	-3.69341	0.776780271	1.462943819	1.29353973	0.899335267	0.380280962	-0.019430984
ENSMUSG00000095253.4	Zfp799	-0.8096	2.186027	-3.42855	0.003815	0.061537	-1.80773	2.674900657	2.739275048	2.33101443	2.164925378	1.640516735	1.565531517
ENSMUSG00000005534.9	Insr	-0.80901	3.745331	-5.24296	0.000105	0.010484	1.425944	4.120403437	4.393155489	3.94042998	3.529621758	3.234786977	3.25358751
ENSMUSG00000030878.10	Cdr2	-0.80897	1.75685	-3.31445	0.004813	0.071288	-1.96881	2.141777088	2.346130154	1.99980852	1.494493535	1.29386621	1.265022405
ENSMUSG00000022708.16	Zbtb20	-0.80854	3.641359	-3.67126	0.002328	0.045748	-1.59237	4.179469378	4.490726976	3.4995687	3.529621758	2.981922204	3.166842012
ENSMUSG00000076431.4	Sox4	-0.80845	4.188676	-4.59246	0.000368	0.01826	0.129066	4.593473058	4.951230301	4.23872684	3.984991352	3.857898053	3.505738432
ENSMUSG00000038042.9	Ptpdc1	-0.80679	1.422031	-2.55375	0.022258	0.164654	-3.2795	1.513745865	1.993458536	1.87004065	1.534570974	1.143840766	0.476526511
ENSMUSG00000036898.16	Zfp157	-0.80668	2.535206	-3.18006	0.006326	0.083061	-2.33149	3.008510557	2.816580774	2.87850223	2.69347457	2.248636956	1.565531517
ENSMUSG00000054263.11	Lifr	-0.80665	2.130101	-2.93941	0.010301	0.108241	-2.70967	2.0361669	3.004513725	2.55868179	2.058991933	1.329055639	1.79319888
ENSMUSG00000045515.3	Pou3f3	-0.80626	4.06364	-3.97515	0.001258	0.032787	-1.06652	4.671965366	4.544737911	4.13660674	4.168113655	3.62997324	3.230442174
ENSMUSG00000027805.16	Pfn2	-0.80395	2.520373	-3.52688	0.003123	0.05441	-1.66934	3.027006901	3.265711472	2.51593215	2.287260921	1.896530713	2.129795025
ENSMUSG00000038126.17	Mphosph9	-0.80274	2.156866	-3.6615	0.002375	0.046124	-1.3669	2.698170436	2.65759125	2.33101443	1.852541056	1.695483194	1.706394053
ENSMUSG00000002249.17	Tead3	-0.80266	1.078683	-2.46933	0.026275	0.176849	-3.37207	1.224239248	2.037179913	1.24200942	0.554199781	0.836659257	0.577809846
ENSMUSG00000024268.15	Celf4	-0.80245	1.578176	-2.7359	0.015503	0.137951	-2.98758	1.798475342	2.571003565	1.65047427	1.17763643	0.97638402	1.295079639
ENSMUSG00000028933.11	Xrcc2	-0.80211	1.117486	-2.23369	0.041446	0.211469	-3.75907	1.460306606	1.525679575	1.5267389	1.322026339	-0.091787482	0.961953338
ENSMUSG00000020181.15	Nav3	-0.80127	1.362645	-2.28438	0.037613	0.204554	-3.71887	1.565276166	2.274871471	1.43792963	1.126106129	1.461810848	0.309876641
ENSMUSG00000039781.14	Cep131	-0.80082	2.657888	-4.07413	0.001031	0.030012	-0.644	3.06330303	2.936875008	3.1117014	2.524655505	2.302744362	2.008049752
ENSMUSG00000046994.9	Mars2	-0.8007	0.952626	-2.34724	0.033322	0.196152	-3.55396	0.776780271	1.585800567	1.65047427	0.77101117	0.508605059	0.423087252
ENSMUSG00000017667.9	Zfp334	-0.80062	3.092145	-3.37528	0.004253	0.065754	-2.06326	3.263767612	3.822839764	3.32483586	3.281384354	2.633496307	2.226548258
ENSMUSG00000026586.16	Prrx1	-0.80046	4.062769	-4.54374	0.000405	0.018666	0.051967	4.593473058	4.521837508	4.23213918	4.122823545	3.535216789	3.37112383
ENSMUSG00000075590.2	Nrbp2	-0.79979	0.682834	-2.3446	0.033493	0.196453	-3.52636	1.158650906	1.015484842	1.07535956	0.099633918	0.626441549	0.121431552
ENSMUSG00000040606.13	Kazn	-0.79937	1.146523	-2.34316	0.033586	0.196627	-3.58787	1.286975003	2.120837843	1.24200942	0.899335267	1.020105398	0.309876641
ENSMUSG00000018849.6	Wwc1	-0.79934	1.584075	-2.37842	0.031369	0.190935	-3.60326	1.882133271	2.571003565	1.48301752	1.648527164	1.29386621	0.625904135
ENSMUSG00000038970.7	Lmtk2	-0.79888	3.276423	-4.48805	0.000452	0.019825	0.063873	3.771305141	3.784871914	3.477458	3.106297648	2.561346521	2.957261643
ENSMUSG00000052105.16	Mtcl1	-0.79851	3.802096	-2.86982	0.011851	0.117456	-3.19092	4.379844578	4.376232186	3.82259348	3.509653342	4.08072068	2.643534029
ENSMUSG00000050199.13	Lgr4	-0.79849	3.018765	-2.66475	0.017865	0.149363	-3.39827	3.680753383	3.209495462	3.22220094	3.316289969	2.890654146	1.79319888
ENSMUSG00000027799.12	Nbea	-0.79844	4.452647	-3.73799	0.002033	0.042311	-1.59656	4.811870936	5.123375565	4.59764082	4.324885993	4.411397086	3.446710856
ENSMUSG00000045071.13	E130308A19Ri	-0.7982	2.969976	-4.47442	0.000464	0.020089	0.078786	3.294628576	3.47093661	3.31239875	2.76259893	2.647503165	2.331791418
ENSMUSG00000032883.15	Acsl3	-0.79799	2.751803	-3.48376	0.00341	0.057409	-1.7899	3.216201929	3.283973678	2.91186082	2.90698884	1.965243463	2.226548258
ENSMUSG00000026620.11	Mark1	-0.79795	1.835757	-2.60604	0.020073	0.15715	-3.25787	2.418886679	2.629302206	1.65047427	1.883905227	1.396959703	1.0350168
ENSMUSG00000032589.14	Bsn	-0.79794	2.676362	-3.74349	0.002011	0.042282	-1.27847	3.294628576	3.302007601	2.69905445	2.086213402	2.388644435	2.287623834
ENSMUSG00000025816.15	Sec61a2	-0.79751	2.615868	-2.93618	0.010368	0.108772	-2.80601	3.24808599	3.004513725	2.69905445	2.844592302	2.284933085	1.614030034
ENSMUSG00000003378.9	Grik5	-0.7969	3.235507	-3.21266	0.005921	0.080088	-2.40792	3.608903098	3.810294511	3.40901694	3.293113647	3.12894189	2.162772347
ENSMUSG00000041703.7	Zic5	-0.79605	1.09788	-2.26517	0.039025	0.207621	-3.70592	1.513745865	1.853733772	1.07535956	1.275483753	0.681936662	0.187019893
ENSMUSG00000044700.15	Tmem201	-0.79545	2.891355	-4.11257	0.000954	0.028907	-0.60116	3.558934639	3.388943238	2.9604956	2.442778498	2.500910667	2.496066697
ENSMUSG00000069274.3	Hist1h4f	-0.79476	3.370289	-2.52237	0.023677	0.169529	-3.74254	3.985051584	4.306481078	2.99203117	3.689042323	2.805765249	2.443359647
ENSMUSG00000025932.14	Eya1	-0.79443	2.443639	-3.41805	0.003898	0.062194	-1.86475	3.027006901	2.841455443	2.60020106	2.524655505	1.896530713	1.771982394
ENSMUSG00000004356.7	Utp20	-0.79437	3.558963	-4.68813	0.000305	0.017041	0.405541	3.726739589	4.074378531	4.01907042	3.509653342	3.057304016	2.966629825
ENSMUSG00000004610.4	Etfb	-0.79391	1.516165	-2.94379	0.01021	0.107613	-2.61201	1.663123489	1.948370647	2.09000633	1.17763643	1.29386621	0.923985488
ENSMUSG00000031004.7	Mki67	-0.79356	5.750371	-3.74163	0.002018	0.042285	-1.68327	6.223237028	6.15224298	6.05404083	6.039104303	5.207863692	4.82573736
ENSMUSG00000026193.15	Fn1	-0.79319	5.528044	-5.29898	9.46E-05	0.010152	1.379514	5.878318298	6.262261422	5.63682286	5.182374349	5.290522767	4.9179617
ENSMUSG00000027201.16	Myef2	-0.79193	3.631097	-4.90152	0.000202	0.01387	0.801032	3.994480997	3.954084298	4.07167451	3.606841261	3.12894189	3.030557152
ENSMUSG00000029454.15	Mapkapk5	-0.79178	1.124401	-2.17288	0.046524	0.217853	-3.85589	1.709666075	1.752450437	1.24200942	-0.131691629	1.329055639	0.844913917
ENSMUSG00000029090.12	Adgra3	-0.79132	3.709979	-3.69277	0.002229	0.044692	-1.56316	4.195910525	4.315386631	3.75985773	3.903167129	3.118923134	2.966629825
ENSMUSG00000028031.6	Dkk2	-0.79043	1.284314	-2.3476	0.033299	0.196152	-3.60236	1.961204842	1.180544089	1.76443046	1.573565106	0.508605059	0.71753461
ENSMUSG00000020844.6	Nxn	-0.78936	2.252498	-2.46427	0.026536	0.177689	-3.58834	2.830418235	3.170763068	1.93638314	2.421562012	1.77419517	1.381667324
ENSMUSG00000026622.15	Nek2	-0.78768	1.687455	-2.19729	0.04442	0.214943	-3.92273	1.922210711	1.993458536	2.14715024	2.421562012	0.836659257	0.803691254
ENSMUSG00000074994.4	Qser1	-0.78719	3.823857	-4.88419	0.000208	0.014125	0.741573	4.313586734	4.482843377	3.88271448	3.519672098	3.35915767	3.385164769
ENSMUSG00000040204.6	Pclaf	-0.78683	2.96152	-2.83879	0.012615	0.122107	-3.06165	3.411866251	3.84760767	2.80937787	3.183950203	2.07295728	2.443359647
ENSMUSG00000022010.19	Tsc22d1	-0.78593	4.811927	-5.34204	8.72E-05	0.009929	1.497087	5.226241601	5.460938683	4.92621612	4.664418536	4.300529941	4.293219297
ENSMUSG00000035914.10	Cd276	-0.78554	2.050274	-3.41098	0.003954	0.062723	-1.82333	2.500570476	2.685336241	2.17489523	1.719785847	1.583372827	1.637681302
ENSMUSG00000055407.13	Map6	-0.78463	3.120503	-4.04227	0.001099	0.030728	-0.7716	3.263767612	3.942634277	3.34939296	2.90698884	2.605067899	2.655168729
ENSMUSG00000061603.7	Akap6	-0.78457	3.359389	-3.24703	0.005521	0.077252	-2.36733	3.715379726	4.270298741	3.33716666	2.728450708	3.449871234	2.655168729
ENSMUSG00000043889.5	Gm8399	-0.78456	6.384591	-3.01974	0.008757	0.098935	-3.12282	6.756833276	6.640861612	6.93486265	5.444094226	6.88954962	5.641342038
ENSMUSG00000053080.10	2700081O15R	-0.78432	4.303433	-3.74051	0.002023	0.042285	-1.57044	4.718228089	5.192677844	4.19874197	4.212025155	3.587011404	3.911912655
ENSMUSG00000032118.15	Fez1	-0.78407	1.982702	-2.4957	0.024951	0.173481	-3.47845	2.208119583	2.600447343	2.2021167	2.378169417	1.583372827	0.923985488
ENSMUSG00000049539.2	Hist1h1a	-0.78397	3.775284	-2.81679	0.013184	0.125387	-3.28629	4.365381349	4.574717068	3.55340685	4.076065446	2.805765249	3.276367379
ENSMUSG00000040929.16	Rfx3	-0.78376	3.433541	-3.21652	0.005874	0.080014	-2.44254	3.897293078	4.031786655	3.48855571	3.509653342	3.271428821	2.402525929
ENSMUSG00000094655.1	Gm25360	-0.78342	1.494294	-2.80558	0.013484	0.126608	-2.85358	1.565276166	2.07961518	1.99980852	1.07266687	1.363407143	0.884991356
ENSMUSG00000037795.14	N4bp2	-0.78323	4.32089	-5.76279	4.01E-05	0.008063	2.314531	4.740814774	4.625726752	4.73909917	4.116235885	4.02815767	3.675306375
ENSMUSG00000046111.16	Cep295	-0.78313	3.436988	-5.1229	0.000132	0.011275	1.240074	3.927143006	3.758993282	3.76898874	3.257635752	2.970824495	2.938340781
ENSMUSG00000051149.15	Adnp	-0.78305	1.780822	-2.74102	0.015345	0.136818	-3.01012	2.072236155	2.629302206	1.83568914	1.453270872	1.695483194	0.998947545
ENSMUSG00000037995.15	Igsf9	-0.78245	3.596487	-4.49423	0.000446	0.019825	0.027383	3.887204261	4.242553751	3.86579117	3.010082333	3.542730843	3.030557152
ENSMUSG00000029283.17	Cdc7	-0.78227	2.238781	-2.31832	0.035236	0.200644	-3.83794	2.627203915	2.791269685	2.35546747	2.504617752	2.230140613	0.923985488
ENSMUSG00000024109.18	Nrxn1	-0.78159	1.84643	-2.42935	0.02841	0.182497	-3.56637	2.072236155	2.936875008	1.72743625	1.684596418	1.695483194	0.961953338
ENSMUSG00000018334.16	Ksr1	-0.78142	1.723887	-3.04189	0.008373	0.096943	-2.46169	2.30224176	2.120837843	1.90359321	1.453270872	1.493175018	1.070206228
ENSMUSG00000067377.12	Tspan6	-0.78109	1.40259	-2.18959	0.045074	0.216249	-3.87924	2.208119583	1.948370647	1.24200942	1.534570974	0.311568212	1.170900228
ENSMUSG00000044337.5	Ackr3	-0.78026	0.877996	-2.35185	0.033026	0.195344	-3.53711	1.513745865	1.10037374	1.13307505	0.899335267	0.311568212	0.309876641
ENSMUSG00000033585.4	Ndn	-0.77934	1.999746	-2.62077	0.019495	0.154813	-3.26194	2.602750871	2.765506589	1.80049971	2.11293074	1.363407143	1.35337828
ENSMUSG00000027499.12	Pkia	-0.7781	1.719497	-3.00979	0.008935	0.099585	-2.51917	2.240180792	2.237877264	1.83568914	1.611532956	1.220802748	1.170900228
ENSMUSG00000032531.15	Amotl2	-0.77768	3.898777	-4.27728	0.000686	0.024172	-0.44359	4.162838703	4.674994296	4.01907042	3.808270789	3.489291584	3.238198617
ENSMUSG00000075254.11	Heg1	-0.77658	3.169501	-4.00901	0.001175	0.031531	-0.84419	3.760292148	3.771990623	3.1817489	2.891640875	2.561346521	2.849984906
ENSMUSG00000034648.9	Lrrn1	-0.77556	2.042324	-3.18371	0.006279	0.082773	-2.24493	2.602750871	2.414034219	2.30613976	1.684596418	1.29386621	1.95255464
ENSMUSG00000066643.12	Wdr35	-0.77521	2.487702	-2.69554	0.016803	0.144352	-3.22427	2.651249389	2.865908486	2.9604956	2.891640875	2.09356756	1.463351122
ENSMUSG00000026764.15	Kif5c	-0.77407	4.071481	-3.92906	0.001381	0.034436	-1.15912	4.450062575	4.951230301	4.01907042	3.824526679	3.409343428	3.774651581
ENSMUSG00000032411.15	Tfdp2	-0.77371	3.448727	-3.14302	0.00682	0.086751	-2.58847	3.975560135	3.810294511	3.63557539	3.864382668	2.546473245	2.860073724
ENSMUSG00000028702.15	Rad54l	-0.77255	2.328005	-2.91645	0.010789	0.111283	-2.7872	2.698170436	2.739275048	2.60020106	2.620870821	1.872879445	1.436633784
ENSMUSG00000047238.4	Mageh1	-0.77222	0.801238	-2.17433	0.046397	0.217853	-3.80466	0.686582462	1.585800567	1.29353973	0.630148634	0.083299224	0.528056811
ENSMUSG00000056004.16	9330182L06Ri	-0.77165	1.089282	-2.35859	0.032598	0.194379	-3.55444	1.404811494	1.643516065	1.43792963	0.202727411	1.220802748	0.625904135
ENSMUSG00000052698.14	Tln2	-0.77033	2.707128	-3.00462	0.009029	0.100298	-2.69569	3.354437971	3.533333147	2.4493344	2.421562012	2.3377205	2.146377909
ENSMUSG00000040464.15	Gtpbp10	-0.76929	1.571106	-2.80631	0.013464	0.126608	-2.86274	1.663123489	2.237877264	1.99980852	0.95945626	1.461810848	1.104557733
ENSMUSG00000021556.11	Golm1	-0.76849	3.417891	-4.99285	0.000169	0.012548	1.000399	3.715379726	3.99899672	3.69426939	3.145646315	3.014712139	2.938340781
ENSMUSG00000034317.14	Trim59	-0.76806	2.541591	-3.04002	0.008405	0.097227	-2.59676	3.098708366	3.319818877	2.37951295	2.442778498	2.113887544	1.894839142
ENSMUSG00000057894.10	Zfp329	-0.76642	2.500516	-3.22027	0.00583	0.07959	-2.24923	3.045269107	3.026373159	2.57959078	2.504617752	1.668261725	2.178982574
ENSMUSG00000021952.15	Xpo4	-0.76632	2.607883	-2.94776	0.010129	0.107188	-2.78274	2.892257488	3.209495462	2.79156659	2.795957527	2.320338422	1.637681302
ENSMUSG00000042104.18	Uggt2	-0.76629	2.633229	-2.542	0.02278	0.166062	-3.53343	2.951554672	2.936875008	3.00754417	2.981371036	2.485397668	1.436633784
ENSMUSG00000097971.3	Gm26917	-0.76567	13.89966	-2.88615	0.011468	0.115188	-3.16822	14.43694165	14.46209433	13.9570627	13.93279122	13.93104173	12.67805456
ENSMUSG00000021087.17	Rtn1	-0.76531	2.631579	-3.73656	0.002039	0.042323	-1.28527	2.932058497	3.247215128	2.87850223	2.442778498	1.942700894	2.346218488
ENSMUSG00000046668.8	Cxxc5	-0.76468	2.128573	-2.31498	0.035463	0.20128	-3.81952	2.892257488	3.04790632	1.65047427	2.086213402	1.55392905	1.540656848
ENSMUSG00000007039.12	Ddah2	-0.76367	3.504041	-3.40078	0.004037	0.063738	-2.09746	3.793082083	4.434615096	3.44364329	3.416208778	3.148773062	2.787923938
ENSMUSG00000020453.17	Patz1	-0.76353	3.679349	-3.65393	0.002412	0.046328	-1.63403	4.094329925	4.474916461	3.61546782	3.653085446	3.234786977	3.003505827
ENSMUSG00000021913.7	Ogdhl	-0.76349	1.828182	-2.59648	0.020456	0.158008	-3.27333	2.0361669	2.274871471	2.2021167	2.164925378	1.329055639	0.961953338
ENSMUSG00000040183.13	Ankrd6	-0.76305	1.596507	-2.5681	0.021637	0.162204	-3.28263	2.107425583	2.478885363	1.43792963	1.126106129	1.257796956	1.170900228
ENSMUSG00000006398.15	Cdc20	-0.76254	2.38324	-3.14953	0.006731	0.086101	-2.36239	2.809201749	2.88995396	2.55868179	2.524655505	1.583372827	1.933571536
ENSMUSG00000038047.18	Haus6	-0.7617	2.929206	-2.89227	0.011328	0.113949	-2.95409	3.150238667	3.422301835	3.24855227	3.405442347	2.153688552	2.195012684
ENSMUSG00000045991.18	Onecut2	-0.76103	2.867967	-3.3957	0.004079	0.064099	-1.98036	3.116090444	3.784871914	2.87850223	2.69347457	2.388644435	2.346218488
ENSMUSG00000052504.6	Epha3	-0.76078	2.989912	-3.44553	0.003686	0.060104	-1.90713	3.383437843	3.319818877	3.31239875	3.132648975	2.661375339	2.129795025
ENSMUSG00000090386.9	Mir99ahg	-0.76055	2.87423	-3.92511	0.001392	0.034465	-0.95923	3.232232039	3.388943238	3.1117014	2.728450708	2.605067899	2.178982574
ENSMUSG00000028832.11	Stmn1	-0.75903	3.749856	-4.21031	0.000785	0.026059	-0.55027	3.994480997	4.58211552	3.848667	3.499564524	3.168335328	3.405973231
ENSMUSG00000069273.2	Hist1h3e	-0.75891	4.041726	-3.11993	0.007148	0.089222	-2.75499	4.503875621	4.951230301	3.81379654	4.135909304	3.481493213	3.364051781
ENSMUSG00000058006.12	Mdn1	-0.75853	4.623926	-3.45623	0.003606	0.059306	-2.18053	4.963607332	5.138508869	4.86791748	4.668926962	4.548340811	3.556253703
ENSMUSG00000045411.16	2410002F23Ri	-0.75778	3.035165	-3.61024	0.002636	0.048857	-1.59603	3.411866251	3.758993282	3.06798002	2.937203453	2.688725828	2.346218488
ENSMUSG00000054728.16	Phactr1	-0.75767	1.111304	-2.22259	0.042332	0.212176	-3.77562	1.347095996	2.120837843	1.07535956	0.899335267	0.380280962	0.844913917

ENSMUSG00000022814.6	Umps	-0.75692	2.736317	-4.22843	0.000757	0.025525	-0.36006	3.183597264	3.069122806	3.08270153	2.463687488	2.230140613	2.388653755
ENSMUSG00000079560.13	Hoxa3	-0.75625	0.976836	-2.13918	0.049581	0.222531	-3.88453	1.565276166	1.462943819	1.01523856	0.70229842	1.062540664	0.052718802
ENSMUSG00000038351.14	Sgsm2	-0.75605	2.052753	-2.36703	0.03207	0.19298	-3.71468	2.072236155	2.414034219	2.66032206	2.215111135	2.030831804	0.923985488
ENSMUSG00000020175.13	Rab36	-0.75527	0.94998	-2.26035	0.039386	0.208097	-3.69159	1.347095996	1.699011178	1.01523856	0.554199781	0.239418426	0.844913917
ENSMUSG00000060981.6	Hist1h4h	-0.75492	3.575156	-2.60311	0.02019	0.157605	-3.64257	3.703929706	4.647047756	3.52134564	3.824526679	2.531445035	3.222643803
ENSMUSG00000033152.13	Podxl2	-0.75402	1.789241	-2.70406	0.01652	0.142834	-3.077	2.332298994	2.571003565	1.6894684	1.17763643	1.583372827	1.381667324
ENSMUSG00000042807.15	Hecw2	-0.75376	0.977333	-2.29947	0.036538	0.202683	-3.63353	1.224239248	1.699011178	1.13307505	0.77101117	0.786906222	0.249755649
ENSMUSG00000045136.5	Tubb2b	-0.75293	5.048678	-4.31246	0.00064	0.023329	-0.51156	5.374437845	5.808718656	5.09182677	4.916499059	4.75976008	4.34082523
ENSMUSG00000097347.1	Gm17275	-0.75218	1.333778	-2.62503	0.019331	0.154133	-3.14497	1.999172692	1.328642728	1.76443046	1.017171757	0.931296131	0.961953338
ENSMUSG00000048126.16	Col6a3	-0.75104	2.563424	-3.46059	0.003574	0.058949	-1.80269	2.721070838	3.388943238	2.77353267	2.086213402	2.09356756	2.317218617
ENSMUSG00000018166.8	Erbb3	-0.74977	2.702317	-2.61337	0.019783	0.155703	-3.42007	2.830418235	3.919457955	2.53746531	2.602134258	2.21140405	2.113019312
ENSMUSG00000047747.9	Rnf150	-0.74935	2.725074	-4.11295	0.000954	0.028907	-0.57835	3.008510557	3.283973678	3.03807854	2.287260921	2.302744362	2.429876417
ENSMUSG00000048696.10	Mex3d	-0.74916	3.358192	-3.98217	0.001241	0.032515	-0.92904	3.975560135	3.907728662	3.33716666	3.052105547	3.078137519	2.798454606
ENSMUSG00000027715.9	Ccna2	-0.7481	2.858371	-3.46243	0.003561	0.05885	-1.84996	3.369010772	3.170763068	3.08270153	3.010082333	2.354895644	2.162772347
ENSMUSG00000040785.17	Ttc3	-0.74542	5.896324	-3.81556	0.001737	0.038858	-1.53739	6.410986291	6.24138605	6.14977412	5.980464536	5.672549083	4.922782741
ENSMUSG00000026153.15	Fam135a	-0.74423	3.29728	-4.01016	0.001173	0.031531	-0.86373	3.680753383	3.692189912	3.57439188	3.316289969	2.948369644	2.571683744
ENSMUSG00000022240.9	Ctnnd2	-0.74375	2.176446	-3.19345	0.006156	0.081955	-2.24663	2.240180792	2.88995396	2.53746531	1.719785847	1.987439209	1.683851484
ENSMUSG00000048644.7	Ctxn1	-0.74366	1.698387	-2.24218	0.04078	0.209905	-3.85148	2.240180792	2.739275048	1.34329276	1.410835606	1.020105398	1.436633784
ENSMUSG00000012443.3	Kif11	-0.74362	3.52288	-3.18922	0.006209	0.082254	-2.51463	4.022404999	4.053239764	3.55340685	3.606841261	3.342033493	2.559352942
ENSMUSG00000001228.14	Uhrf1	-0.74347	4.224597	-3.45236	0.003635	0.059702	-2.13164	4.706800741	4.813441066	4.23872684	4.471450888	3.441856241	3.675306375
ENSMUSG00000042595.7	Fam199x	-0.74204	2.254186	-2.65056	0.018376	0.151282	-3.25808	2.932058497	2.913605228	2.03050532	2.3105307	1.748432074	1.58998456
ENSMUSG00000074165.10	Zfp788	-0.74198	1.511956	-2.49491	0.02499	0.17357	-3.39396	2.175329648	1.643516065	1.76443046	1.648527164	0.735375921	1.104557733
ENSMUSG00000036782.13	Klhl13	-0.7415	1.943807	-2.92439	0.010618	0.110192	-2.70663	1.999172692	2.685336241	2.28082867	1.719785847	1.29386621	1.683851484
ENSMUSG00000060678.4	Hist1h4c	-0.74079	2.580709	-2.32065	0.035078	0.200218	-3.91157	2.871937504	3.63642664	2.33101443	2.90698884	1.987439209	1.750449233
ENSMUSG00000038252.13	Ncapd2	-0.74021	4.677476	-3.65294	0.002417	0.04633	-1.79424	5.139653916	5.326441819	4.6578664	4.785615583	4.282691135	3.872586335
ENSMUSG00000052516.17	Robo2	-0.73921	3.107423	-2.72323	0.0159	0.139769	-3.31054	3.30981498	3.63642664	3.40901694	3.010082333	3.289406562	1.989787546
ENSMUSG00000032332.17	Col12a1	-0.73853	3.043981	-2.97579	0.009571	0.103779	-2.82122	2.892257488	3.650570823	3.61546782	3.024226515	2.902383439	2.178982574
ENSMUSG00000050947.9	Amigo1	-0.73848	1.446722	-2.58763	0.020818	0.158953	-3.22535	2.0361669	1.803980737	1.65047427	1.07266687	0.735375921	1.381667324
ENSMUSG00000001870.15	Ltbp1	-0.73768	2.094929	-2.81738	0.013169	0.125327	-2.92655	2.674900657	2.510249534	2.2021167	1.684596418	2.173184728	1.324523417
ENSMUSG00000033705.16	Stard9	-0.73677	2.575688	-2.92385	0.010629	0.110226	-2.82101	3.199991702	3.283973678	2.37951295	2.355973671	2.320338422	1.914335318
ENSMUSG00000047193.15	Dync2h1	-0.73563	3.975138	-3.43842	0.003739	0.060677	-2.11588	4.220226186	4.46694575	4.27763333	4.027740999	3.863909291	2.994374816
ENSMUSG00000046591.10	Ticrr	-0.73561	2.437796	-2.66372	0.017902	0.149529	-3.27169	2.871937504	3.026373159	2.4493344	2.675663294	2.113887544	1.489582663
ENSMUSG00000030683.11	Sez6l2	-0.73337	0.736836	-2.13888	0.049609	0.222531	-3.8499	1.158650906	1.328642728	0.81820172	0.70229842	0.163469573	0.249755649
ENSMUSG00000038418.7	Egr1	-0.73309	2.202526	-3.26037	0.005373	0.075998	-2.1255	2.577876203	2.816580774	2.35546747	1.684596418	2.05204829	1.728589799
ENSMUSG00000036768.5	Kif15	-0.7327	3.276056	-3.23177	0.005695	0.07862	-2.37942	3.956387826	3.56354776	3.33716666	3.426895457	2.902383439	2.469953857
ENSMUSG00000023411.11	Nfatc4	-0.73252	3.130677	-2.98432	0.009407	0.102716	-2.82398	3.771305141	3.486790561	3.15413667	3.383665405	2.71556744	2.272595625
ENSMUSG00000027699.19	Ect2	-0.73176	2.695116	-2.69439	0.016841	0.14459	-3.27072	3.116090444	3.319818877	2.67981823	2.966798235	2.3377205	1.750449233
ENSMUSG00000034557.14	Zfyve9	-0.7314	2.596949	-2.90975	0.010936	0.11185	-2.85156	3.133265588	3.04790632	2.6405588	2.779374643	2.230140613	1.750449233
ENSMUSG00000036202.15	Rif1	-0.73136	4.719889	-3.22207	0.005808	0.07959	-2.65641	5.065541502	4.951230301	5.18345724	4.838601865	4.645759727	3.634746011
ENSMUSG00000034813.16	Grip1	-0.73133	1.873622	-2.25102	0.040096	0.208709	-3.87292	2.0361669	2.712557709	1.93638314	1.787689911	1.965243463	0.803691254
ENSMUSG00000024827.9	Gldc	-0.7295	1.264753	-2.32274	0.034937	0.200218	-3.63978	1.513745865	1.803980737	1.5267389	1.453270872	0.445869304	0.844913917
ENSMUSG00000021936.13	Mapk8	-0.7293	3.66649	-4.70069	0.000298	0.016849	0.413824	4.283132937	3.954084298	3.848667	3.361554703	3.39280806	3.15869117
ENSMUSG00000039953.13	Clstn1	-0.72905	3.859304	-4.45032	0.000487	0.02053	-0.09917	4.07668206	4.490726976	4.08635855	3.766811182	3.497048028	3.238198617
ENSMUSG00000068735.14	Trp53i11	-0.72845	4.178481	-3.65373	0.002413	0.046328	-1.72391	4.358094995	5.045232186	4.23872684	4.148877437	3.47365246	3.806302467
ENSMUSG00000051236.13	Msrb3	-0.7275	1.082758	-2.16505	0.047218	0.219095	-3.86148	1.089938156	1.643516065	1.65047427	0.630148634	0.311568212	1.170900228
ENSMUSG00000081683.5	Fzd10	-0.72715	2.869147	-2.83316	0.012758	0.122723	-3.05185	3.425873109	3.283973678	2.92825526	3.221263362	2.009298643	2.346218488
ENSMUSG00000048330.14	Ric3	-0.72649	1.168985	-2.32077	0.03507	0.200218	-3.62765	1.089938156	1.993458536	1.5267389	0.899335267	0.786906222	0.71753461
ENSMUSG00000016933.17	Plcg1	-0.72592	4.397395	-3.88973	0.001495	0.035893	-1.28655	4.934509976	4.788074346	4.53479117	4.230442789	4.352759899	3.543789738
ENSMUSG00000043015.15	Nemp2	-0.72518	1.635132	-2.75719	0.014857	0.134251	-2.95874	1.961204842	2.07961518	1.90359321	1.648527164	1.182834898	1.0350168
ENSMUSG00000024298.14	Zfp871	-0.72435	3.673428	-4.02165	0.001146	0.03138	-0.90766	4.067776506	4.084832889	3.91597693	3.509653342	3.57240171	2.889923652
ENSMUSG00000034949.18	Zfr2	-0.72368	0.950472	-2.26669	0.038911	0.207284	-3.68169	1.404811494	1.585800567	1.01523856	0.389140535	0.681936662	0.625904135
ENSMUSG00000108621.1	Gm37494	-0.72332	3.337723	-3.37763	0.004232	0.065579	-2.10844	3.715379726	3.678449855	3.61546782	3.634765262	2.71556744	2.666710351
ENSMUSG00000046709.16	Mapk10	-0.7223	2.347433	-3.26911	0.005278	0.07552	-2.13141	2.721070838	3.04790632	2.40316422	1.944659258	2.07295728	1.894839142
ENSMUSG00000028476.13	Reck	-0.72188	2.032236	-2.181	0.045814	0.217545	-4.02282	2.698170436	2.380481659	1.99980852	2.287260921	1.942700894	0.884991356
ENSMUSG00000020492.11	Ska2	-0.72164	2.296511	-2.53575	0.023062	0.167189	-3.47159	2.951554672	2.65759125	2.28082867	2.583151155	1.896530713	1.409412315
ENSMUSG00000047534.16	Mis18bp1	-0.72071	2.320147	-2.28569	0.037519	0.204554	-3.91148	2.698170436	2.65759125	2.53746531	2.952076729	1.722200532	1.35337828
ENSMUSG00000020674.16	Pxdn	-0.72041	4.350528	-3.21361	0.005909	0.080014	-2.62386	4.98268457	4.922375438	4.23872684	3.984991352	4.455853392	3.518534037
ENSMUSG00000020330.16	Hmmr	-0.71973	1.826671	-2.20839	0.043493	0.213784	-3.93326	2.332298994	1.803980737	2.25506558	2.139162282	1.668261725	0.761255988
ENSMUSG00000052534.15	Pbx1	-0.71935	4.845453	-5.36599	8.34E-05	0.009818	1.53938	5.214185583	5.391050901	5.01331881	4.659895978	4.264628989	4.529637847
ENSMUSG00000024998.17	Plce1	-0.71915	1.79488	-2.37012	0.031878	0.192604	-3.65711	2.271544963	2.07961518	1.99980852	2.031246943	1.583372827	0.803691254
ENSMUSG00000019841.15	Rev3l	-0.7183	4.173397	-3.12071	0.007136	0.089222	-2.77716	4.605830264	4.709182983	4.27122135	3.856499069	4.390727749	3.206919451
ENSMUSG00000068747.14	Sort1	-0.71765	3.988722	-4.41491	0.000522	0.020975	-0.18825	4.450062575	4.490726976	4.10089465	3.910800454	3.333394574	3.646451512
ENSMUSG00000070407.5	Hs3st3b1	-0.71654	1.342058	-2.38723	0.030837	0.189038	-3.54652	1.663123489	1.853733772	1.5267389	1.410835606	1.020105398	0.577809846
ENSMUSG00000026941.16	Mamdc4	-0.71639	1.51397	-2.3966	0.030281	0.187577	-3.56013	1.999172692	1.752450437	1.76443046	1.787689911	0.97638402	0.803691254
ENSMUSG00000037640.15	Zfp60	-0.7157	2.978176	-3.09232	0.007559	0.091718	-2.58508	3.596572296	3.56354776	2.844354	3.024226515	2.437831985	2.402525929
ENSMUSG00000004631.15	Sgce	-0.71519	1.757531	-2.22356	0.042255	0.212176	-3.8939	2.332298994	1.585800567	2.25506558	2.190236466	1.182834898	0.998947545
ENSMUSG00000048763.11	Hoxb3	-0.71505	1.575283	-2.28096	0.037861	0.205029	-3.76359	1.999172692	2.346130154	1.43792963	1.719785847	1.103763327	0.844913917
ENSMUSG00000022261.5	Sdc2	-0.7147	1.42497	-2.36816	0.031999	0.192814	-3.59207	2.107425583	1.462943819	1.72743625	1.07266687	1.461810848	0.71753461
ENSMUSG00000068154.5	Insm1	-0.71358	2.942978	-3.47465	0.003474	0.058192	-1.84194	3.216201929	3.705800343	2.99203117	2.828562193	2.485397668	2.429876417
ENSMUSG00000020627.9	Klhl29	-0.71355	1.057312	-2.2553	0.039769	0.208663	-3.71535	1.089938156	1.525679575	1.56917417	1.07266687	0.508605059	0.577809846
ENSMUSG00000050332.7	Amer1	-0.713	3.086385	-3.66168	0.002374	0.046124	-1.50541	3.546167343	3.607715344	3.15413667	3.065845604	2.661375339	2.483069357
ENSMUSG00000078154.3	Gm12184	-0.71233	1.681232	-2.76482	0.014632	0.133032	-2.95222	2.240180792	1.853733772	1.99980852	1.494493535	1.062540664	1.436633784
ENSMUSG00000043668.13	Tox3	-0.71084	2.08263	-2.51303	0.024116	0.170977	-3.46793	2.602750871	2.629302206	2.03050532	2.355973671	1.523871816	1.35337828
ENSMUSG00000058571.10	Gpc6	-0.71057	2.949903	-2.40246	0.029938	0.187288	-3.85628	3.584135193	3.4549065	2.87850223	2.504617752	3.324703614	1.95255464
ENSMUSG00000015709.8	Arnt2	-0.71029	3.331181	-3.55682	0.002939	0.052859	-1.7569	3.760292148	4.115748966	3.23543677	3.052105547	2.914018139	2.909485918
ENSMUSG00000036459.15	Wtip	-0.70988	1.504715	-2.37295	0.031704	0.191895	-3.59803	1.961204842	1.853733772	1.6894684	1.534570974	1.363407143	0.625904135
ENSMUSG00000040690.15	Col16a1	-0.70906	1.251385	-2.50775	0.024368	0.171379	-3.33191	1.404811494	1.803980737	1.61039683	0.95945626	0.884753545	0.844913917
ENSMUSG00000031561.15	Tenm3	-0.70873	4.914875	-4.0442	0.001095	0.030728	-1.03522	5.451774973	5.330840283	5.00561414	5.043451082	4.331226738	4.326343853
ENSMUSG00000061751.15	Kalrn	-0.70872	4.614624	-4.30738	0.000646	0.023443	-0.48329	4.784954202	5.330840283	4.80716344	4.193369356	4.552068708	4.019347344
ENSMUSG00000031565.17	Fgfr1	-0.70834	3.810583	-3.298	0.004977	0.072879	-2.36132	4.313586734	4.544737911	3.64552505	3.724124782	3.535216789	3.10030826
ENSMUSG00000013629.16	Cad	-0.70814	4.088395	-2.77614	0.014304	0.131287	-3.42718	4.618082525	4.794457963	3.89944156	4.402738138	3.234786977	3.58086317
ENSMUSG00000047003.14	Zfp41	-0.70808	3.14958	-2.54365	0.022706	0.166062	-3.6509	3.520288711	3.79763921	3.16800884	3.519672098	2.05204829	2.83982504
ENSMUSG00000034206.15	Polq	-0.70748	2.192034	-2.31982	0.035134	0.200368	-3.82499	2.651249389	2.414034219	2.4493344	2.355973671	2.21140405	1.070206228
ENSMUSG00000040661.6	Rad54l2	-0.70699	4.309979	-5.05255	0.000151	0.011837	0.998683	4.729565633	4.781662358	4.46907833	4.205833321	3.851861663	3.82187127
ENSMUSG00000020086.6	H2afy2	-0.70686	3.138035	-3.07044	0.007902	0.093843	-2.66107	3.657917866	3.835276868	3.00754417	3.145646315	2.405227319	2.777315838
ENSMUSG00000001436.15	Slc19a1	-0.70669	1.591855	-2.5237	0.023615	0.169371	-3.35816	1.922210711	1.853733772	1.96844435	1.787689911	1.020105398	0.998947545
ENSMUSG00000024975.12	Pdcd4	-0.70628	2.718917	-3.49877	0.003307	0.056389	-1.75541	3.06330303	3.319818877	2.82697193	2.602134258	2.354895644	2.146377909
ENSMUSG00000022048.8	Dpysl2	-0.70622	5.846235	-4.33646	0.00061	0.022923	-0.49565	5.981499604	6.653207488	5.9654687	5.624356996	5.29275259	5.560126712
ENSMUSG00000032402.12	Smad3	-0.70493	3.084928	-2.79913	0.01366	0.127556	-3.16373	3.150238667	4.042553086	3.12598563	3.221263362	2.3377205	2.631804736
ENSMUSG00000033257.14	Ttll4	-0.70422	4.424397	-3.61891	0.00259	0.048656	-1.83036	4.827783174	4.898868685	4.56131205	4.595044833	4.070360169	3.593012253
ENSMUSG00000033883.11	D3Ertd254e	-0.70395	1.862993	-2.87376	0.011758	0.116967	-2.78552	2.271544963	1.993458536	2.30613976	1.820479846	1.461810848	1.324523417
ENSMUSG00000006678.6	Pola1	-0.70297	2.953124	-3.08451	0.00768	0.092428	-2.59463	3.467095772	3.247215128	3.12598563	2.981371036	2.818202352	2.07887109
ENSMUSG00000066687.4	Zbtb16	-0.70046	1.855949	-2.44035	0.027807	0.180696	-3.54933	2.072236155	2.274871471	2.2021167	1.719785847	1.942700894	0.923985488
ENSMUSG00000025665.16	Rps6ka6	-0.70017	2.740429	-3.04246	0.008363	0.096914	-2.63098	3.383437843	3.110642075	2.73677412	2.795957527	2.371868722	2.043894951
ENSMUSG00000006651.7	Aplp1	-0.70016	2.899466	-3.13467	0.006937	0.087573	-2.48786	2.951554672	3.705800343	3.15413667	2.239564179	2.902383439	2.443359647
ENSMUSG00000029101.14	Rgs12	-0.69977	3.47978	-4.17117	0.000848	0.027304	-0.58097	3.856507464	3.883980059	3.71331514	3.361554703	3.244035054	2.819288109
ENSMUSG00000031465.6	Angpt2	-0.69866	0.948179	-2.15307	0.0483	0.220594	-3.85864	1.565276166	0.829071718	1.43792963	0.70229842	0.626441549	0.528056811
ENSMUSG00000018199.8	Trove2	-0.69831	3.868803	-4.96715	0.000178	0.012885	0.892814	4.187713372	4.450870987	4.04185029	3.519672098	3.481493213	3.531217152
ENSMUSG00000061143.15	Maml3	-0.69786	2.625548	-2.29739	0.036685	0.202683	-3.96223	3.008510557	3.438696273	2.51593215	2.11293074	2.970824495	1.706394053
ENSMUSG00000055485.6	Soga1	-0.69783	4.053728	-3.43504	0.003765	0.060876	-2.1368	4.306033339	4.800813458	4.09364491	4.18079677	3.342033493	3.599048643
ENSMUSG00000017405.14	Nek8	-0.69777	1.478853	-2.29597	0.036785	0.202884	-3.72086	2.107425583	1.948370647	1.43792963	1.494493535	0.681936662	1.202961437
ENSMUSG00000005871.14	Apc	-0.69748	5.273039	-6.03614	2.46E-05	0.006757	2.742514	5.66164456	5.605901773	5.59122027	5.060708406	4.994305928	4.724454024
ENSMUSG00000017146.12	Brca1	-0.69719	3.036962	-3.03795	0.00844	0.097299	-2.70112	3.339716465	3.150999813	3.54279875	3.350370965	2.421621757	2.416265986
ENSMUSG00000048349.9	Pou4f1	-0.69679	3.175698	-2.95524	0.009977	0.105999	-2.8894	3.425873109	4.185409844	3.02289214	2.981371036	2.71556744	2.723072794
ENSMUSG00000020363.6	Gfpt2	-0.69626	1.459154	-2.53522	0.023086	0.167273	-3.31682	1.961204842	1.853733772	1.61039683	1.322026339	0.836659257	1.170900228
ENSMUSG00000070436.11	Serpinh1	-0.69623	4.822348	-4.8607	0.000218	0.014275	0.579739	4.996828752	5.531423557	4.99397944	4.527140769	4.529555454	4.35516269
ENSMUSG00000027950.7	Chrnb2	-0.696	2.008157	-2.72263	0.015919	0.139769	-3.08201	1.961204842	2.65759125	2.47187697	1.719785847	1.363407143	1.875075887
ENSMUSG00000026196.7	Bard1	-0.69536	1.605555	-2.56157	0.021917	0.163576	-3.29542	1.840910608	2.037179913	1.93638314	1.684596418	0.931296131	1.202961437
ENSMUSG00000001467.5	Cyp51	-0.69393	3.8862	-4.0886	0.001001	0.02986	-0.81268	4.137527615	4.567280479	3.99592509	3.832586449	3.350620989	3.433259057
ENSMUSG00000038894.6	Irs2	-0.69387	2.620142	-2.72836	0.015738	0.139139	-3.19223	3.045269107	3.4549065	2.4493344	2.563914936	1.919800492	2.287623834
ENSMUSG00000030254.16	Rad18	-0.69326	1.923895	-2.86574	0.011949	0.118161	-2.80971	2.107425583	2.07961518	2.55868179	1.754137352	1.55392905	1.489582663
ENSMUSG00000043557.15	Mdga1	-0.69275	2.457321	-3.13373	0.00695	0.087573	-2.40503	2.674900657	3.150999813	2.60020106	2.355973671	1.848833971	2.113019312
ENSMUSG00000055639.16	Dach1	-0.69212	3.274055	-2.46504	0.026497	0.177602	-3.8231	3.520288711	3.99899672	3.2871982	3.350370965	3.324703614	2.162772347
ENSMUSG00000023017.9	Asic1	-0.69194	1.399369	-2.48331	0.025565	0.175411	-3.39533	1.513745865	2.07961518	1.6894684	0.899335267	1.143840766	1.070206228
ENSMUSG00000024426.17	Atat1	-0.69159	2.99451	-3.70554	0.002172	0.043866	-1.40371	3.027006901	3.650570823	3.36151652	2.602134258	2.741918767	2.583910046
ENSMUSG00000029651.16	Mtus2	-0.69071	1.585202	-2.18931	0.045097	0.216249	-3.91512	1.404811494	2.65759125	1.76443046	1.322026339	1.363407143	0.998947545

ENSMUSG00000033948.3	Zswim5	-0.68866	2.469885	-2.89893	0.011177	0.113194	-2.84653	2.698170436	3.170763068	2.55868179	2.378169417	2.284933085	1.728589799
ENSMUSG00000018451.6	6330403K07Ri	-0.68857	1.935802	-2.30114	0.036421	0.202683	-3.80205	2.526802018	2.478885363	1.80049971	1.974103035	1.799506258	1.0350168
ENSMUSG00000025026.14	Add3	-0.68763	2.266888	-2.87906	0.011633	0.116063	-2.84488	2.892257488	2.791269685	2.2021167	1.787689911	2.113887544	1.81410787
ENSMUSG00000061411.12	Nol4l	-0.68695	3.009125	-2.85974	0.012094	0.119071	-3.03296	3.425873109	3.942634277	2.77353267	2.7106863	2.546473245	2.655168729
ENSMUSG00000038587.8	Akap12	-0.6864	3.420045	-3.8124	0.001749	0.038925	-1.27275	3.596572296	4.214264707	3.53211207	3.010082333	3.118923134	3.048313623
ENSMUSG00000022360.7	Atad2	-0.68639	3.771541	-2.95685	0.009944	0.105823	-3.01737	4.290806865	4.165847577	3.81379654	4.076065446	3.39280806	2.889923652
ENSMUSG00000051934.4	Spats2	-0.68563	2.817643	-3.08373	0.007692	0.092428	-2.56827	3.533286052	3.110642075	2.80937787	2.844592302	2.320338422	2.287623834
ENSMUSG00000045095.16	Magi1	-0.6849	3.552392	-2.64513	0.018575	0.152181	-3.5592	3.907311834	4.084832889	3.60530795	3.925946997	3.244035054	2.546915839
ENSMUSG00000013236.16	Ptprs	-0.68459	6.039459	-3.10829	0.007318	0.090347	-2.95019	6.364472565	6.758177048	6.02384598	5.886810958	6.117036847	5.086410387
ENSMUSG00000046329.14	Slc25a23	-0.68302	1.987859	-2.51303	0.024116	0.170977	-3.44926	1.961204842	2.841455443	2.2021167	1.944659258	1.29386621	1.683851484
ENSMUSG00000017716.15	Birc5	-0.68287	1.930818	-2.85643	0.012175	0.119504	-2.82771	2.361742772	2.274871471	2.14715024	1.914602024	1.396959703	1.489582663
ENSMUSG00000026787.3	Gad2	-0.68253	1.062469	-2.23134	0.041632	0.211615	-3.7541	1.347095996	1.256492942	1.61039683	0.630148634	0.568726051	0.961953338
ENSMUSG00000092035.8	Peg10	-0.6823	5.28175	-2.79237	0.013846	0.128445	-3.54218	5.990952186	5.594972213	5.25993133	5.679019701	4.713904391	4.45172312
ENSMUSG00000044636.5	Csrnp2	-0.68209	2.245676	-2.51875	0.023847	0.170191	-3.49116	2.30224176	3.209495462	2.33101443	1.944659258	1.523871816	2.162772347
ENSMUSG00000005233.16	Spc25	-0.68189	1.911495	-2.45681	0.026926	0.178609	-3.5317	2.446631669	2.160915282	2.06056255	2.11293074	1.583372827	1.104557733
ENSMUSG00000002835.8	Chaf1a	-0.67958	3.200992	-3.91574	0.001419	0.034612	-1.03103	3.680753383	3.678449855	3.2744309	3.024226515	2.728803267	2.819288109
ENSMUSG00000028782.14	Adgrb2	-0.67938	1.489864	-2.4765	0.025909	0.176124	-3.42151	1.754753965	2.07961518	1.65047427	1.275483753	1.29386621	0.884991356
ENSMUSG00000032363.15	Adamts7	-0.67855	2.091856	-2.55077	0.022389	0.16509	-3.40341	2.271544963	2.88995396	2.11886119	2.139162282	1.722200532	1.409412315
ENSMUSG00000029469.14	Ift81	-0.67812	1.877838	-2.92226	0.010663	0.110463	-2.70013	2.072236155	2.274871471	2.28082867	1.648527164	1.55392905	1.436633784
ENSMUSG00000021175.15	Cdca7l	-0.67741	1.960986	-2.16098	0.047583	0.219824	-4.03971	2.271544963	2.07961518	2.40316422	2.139162282	1.987439209	0.884991356
ENSMUSG00000028613.14	Lrp8	-0.67615	2.82607	-3.07371	0.00785	0.093553	-2.58909	3.263767612	3.548519551	2.71803755	2.69347457	2.302744362	2.429876417
ENSMUSG00000027115.14	Kif18a	-0.67527	1.750618	-2.26733	0.038864	0.207284	-3.82033	1.882133271	2.037179913	2.25506558	1.611532956	1.872879445	0.844913917
ENSMUSG00000067928.6	Zfp760	-0.67501	2.035613	-2.32931	0.034496	0.199349	-3.77532	1.999172692	2.739275048	2.33101443	2.333431102	1.220802748	1.58998456
ENSMUSG00000027204.13	Fbn1	-0.67486	3.577606	-4.06963	0.00104	0.030085	-0.79647	4.013156922	4.125908832	3.61546782	3.426895457	3.158587352	3.125619349
ENSMUSG00000029661.16	Col1a2	-0.67476	6.101863	-5.57093	5.70E-05	0.008819	1.880073	6.357181608	6.675858962	6.28481292	5.882935347	5.696325643	5.714062418
ENSMUSG00000036306.11	Lzts1	-0.67356	2.557558	-2.29832	0.036619	0.202683	-3.94459	3.027006901	3.422301835	2.25506558	2.76259893	1.799506258	2.07887109
ENSMUSG00000030528.12	Blm	-0.67223	2.802617	-2.19221	0.044851	0.215717	-4.1844	3.324843189	3.247215128	2.77353267	2.828562193	3.003864757	1.637681302
ENSMUSG00000074221.11	Zfp568	-0.67148	3.229345	-3.1049	0.007369	0.090645	-2.61454	3.30981498	3.919457955	3.44364329	3.372652412	2.500910667	2.829593117
ENSMUSG00000017485.10	Top2b	-0.67055	5.533692	-5.79768	3.77E-05	0.008043	2.304141	5.750586331	5.969681153	5.88376562	5.379545378	5.079429678	5.13914634
ENSMUSG00000062115.15	Rai1	-0.67053	4.005455	-3.54602	0.003004	0.053603	-1.90825	4.443192585	4.574717068	3.99592509	3.887778236	3.869895586	3.261220835
ENSMUSG00000039328.9	Rnf122	-0.67027	2.224006	-2.51016	0.024253	0.171081	-3.50177	2.651249389	2.765506589	2.28082867	2.239564179	1.29386621	2.113019312
ENSMUSG00000015880.13	Ncapg	-0.66954	2.648804	-2.13648	0.049835	0.222753	-4.23984	3.199991702	2.629302206	2.9604956	2.952076729	2.661375339	1.489582663
ENSMUSG00000040209.12	Zfp704	-0.66948	3.160745	-3.684	0.002269	0.045041	-1.47546	3.439745284	3.502472184	3.48855571	3.233489664	2.633496307	2.666710351
ENSMUSG00000000823.16	Zfp512b	-0.66938	3.50464	-3.55072	0.002975	0.053262	-1.80305	3.738010701	4.074378531	3.67496883	3.625517186	2.902383439	3.012579411
ENSMUSG00000063810.6	Alms1	-0.66917	3.487384	-2.48516	0.025472	0.175276	-3.83796	3.760292148	4.306481078	3.37353905	3.559065536	3.441856241	2.483069357
ENSMUSG00000022704.15	Qtrt2	-0.66628	1.868705	-2.72727	0.015772	0.139132	-3.04927	2.208119583	2.414034219	2.03050532	1.322026339	1.77419517	1.463351122
ENSMUSG00000060548.12	Tnfrsf19	-0.66593	1.988862	-2.22542	0.042105	0.212176	-3.93963	2.240180792	2.380481659	2.22883404	2.3105307	1.77419517	0.998947545
ENSMUSG00000038535.17	Zfp280d	-0.66583	3.848133	-4.29658	0.00066	0.023696	-0.39733	4.111764519	4.146016406	4.23213918	3.848572153	3.481493213	3.268813985
ENSMUSG00000028906.15	Epb41	-0.6639	5.360686	-5.21735	0.00011	0.010497	1.230764	5.649758231	5.946938691	5.48440406	5.15371059	4.963844825	4.965462124
ENSMUSG00000094936.7	Rbm4	-0.6632	1.251608	-2.14925	0.048649	0.220972	-3.91528	1.347095996	1.752450437	1.61039683	1.367114228	0.508605059	0.923985488
ENSMUSG00000061482.6	Hist1h4d	-0.66285	3.804083	-2.40187	0.029972	0.187415	-4.06168	4.022404999	4.749167853	3.61546782	4.187096759	3.158587352	3.091771578
ENSMUSG00000030770.14	Parva	-0.66237	2.005138	-2.18738	0.045263	0.216614	-4.00642	2.332298994	3.069122806	1.6894684	1.820479846	1.55392905	1.565531517
ENSMUSG00000020121.15	Srgap1	-0.66154	2.552208	-2.15822	0.047832	0.220095	-4.18069	2.577876203	3.771990623	2.35546747	2.355973671	2.437831985	1.81410787
ENSMUSG00000028456.17	Unc13b	-0.66131	2.816011	-3.29114	0.005047	0.073296	-2.17159	3.06330303	3.228478566	3.1117014	2.620870821	2.741918767	2.129795025
ENSMUSG00000004880.11	Lbr	-0.66115	3.14706	-2.72627	0.015804	0.139518	-3.31395	3.726739589	3.745877783	2.94446549	3.293113647	2.702209057	2.469953857
ENSMUSG00000037907.16	Ankrd13b	-0.66113	3.433807	-3.87475	0.001541	0.036133	-1.15285	3.927143006	3.919457955	3.45500315	3.281384354	3.02547857	2.994374816
ENSMUSG00000075031.3	Hist1h2bb	-0.66109	3.190353	-2.67211	0.017605	0.148024	-3.42467	3.657198655	3.954084298	2.9604956	3.339099852	2.576068027	2.655168729
ENSMUSG00000022120.3	Rnf219	-0.66024	2.421798	-3.08617	0.007654	0.092285	-2.48805	2.871937504	2.913605228	2.49407271	2.239564179	1.848833971	2.162772347
ENSMUSG00000034653.11	Ythdc2	-0.65992	2.161756	-3.02261	0.008706	0.098507	-2.56166	2.500570476	2.571003565	2.37951295	2.058991933	1.799506258	1.660951082
ENSMUSG00000037098.17	Rab11fip3	-0.6595	3.222242	-3.12422	0.007086	0.089029	-2.57583	3.771305141	3.79763921	3.09727433	3.079456036	3.003864757	2.583910046
ENSMUSG00000063804.7	Lin28b	-0.65937	2.779412	-2.46649	0.026421	0.177364	-3.70165	3.294628576	3.004513725	2.92825526	2.922175243	2.754916107	1.771982394
ENSMUSG00000016624.15	Phf21b	-0.65826	3.446997	-3.05336	0.008181	0.095707	-2.76131	3.782234701	4.125908832	3.44364329	3.079456036	3.527663395	2.723072794
ENSMUSG00000030930.13	Chst15	-0.65684	2.531529	-2.90616	0.011015	0.112165	-2.84522	2.787668587	3.319818877	2.53746531	2.239564179	1.987439209	2.317218617
ENSMUSG00000021767.16	Kat6b	-0.65577	3.820512	-2.84981	0.012338	0.120323	-3.23266	4.372631088	4.13599765	3.87427764	3.918393603	3.751677733	2.87009248
ENSMUSG00000040093.15	Bmf	-0.65544	1.838941	-2.58781	0.02081	0.158953	-3.29046	2.30224176	2.199909414	2.06056255	1.322026339	1.29386621	1.855038134
ENSMUSG00000097195.8	Snhg5	-0.65476	2.551709	-3.06692	0.007959	0.094099	-2.5479	2.743613407	2.685336241	3.12598563	2.463687488	2.320338422	1.971291202
ENSMUSG00000039716.12	Dock3	-0.65389	1.750751	-2.13485	0.049987	0.222777	-4.03693	2.107425583	2.120837843	1.96844435	1.410835606	2.05204829	0.844913917
ENSMUSG00000017861.11	Mybl2	-0.65371	1.863075	-2.56963	0.021571	0.161802	-3.32642	2.072236155	2.07961518	2.33101443	1.974103035	1.396959703	1.324523417
ENSMUSG00000076258.1	Gm23935	-0.65357	15.27642	-2.26502	0.039036	0.207621	-4.27189	15.887465	16.02189195	14.9036155	15.56300573	14.90618725	14.37633485
ENSMUSG00000001517.14	Foxm1	-0.65346	3.045604	-2.45028	0.027273	0.17995	-3.79411	3.30981498	3.822839764	2.94446549	3.269558919	2.780564695	2.146377909
ENSMUSG00000050334.12	C130071C03Ri	-0.65286	2.535803	-3.21565	0.005885	0.080014	-2.26407	2.809201749	2.841455443	2.89527794	2.524655505	1.965243463	2.178982574
ENSMUSG00000023150.14	Ivns1abp	-0.65248	5.0711	-3.82889	0.001691	0.038362	-1.4778	5.381642329	5.561677773	5.23378892	5.241078819	4.475616647	4.53279473
ENSMUSG00000020527.14	Myo19	-0.65161	1.633294	-2.21189	0.043204	0.212903	-3.8878	1.513745865	2.237877264	2.06056255	1.820479846	1.062540664	1.104557733
ENSMUSG00000061436.15	Hipk2	-0.65154	5.17331	-5.34182	8.73E-05	0.009929	1.474039	5.478900473	5.730978906	5.29512076	4.858474011	4.871388287	4.804997451
ENSMUSG00000039741.15	Bahcc1	-0.65101	4.60515	-3.96528	0.001284	0.03302	-1.16201	5.033882347	5.11319778	4.63307722	4.641662622	4.185237388	4.023841724
ENSMUSG00000031955.9	Bcar1	-0.65067	2.65636	-2.9139	0.010845	0.111598	-2.85579	3.24808599	3.150999813	2.55868179	2.463687488	2.437831985	2.07887109
ENSMUSG00000026604.17	Ptpn14	-0.6504	2.190292	-2.14016	0.04949	0.222483	-4.12554	1.754753965	3.228478566	2.55868179	2.11293074	1.872879445	1.614030034
ENSMUSG00000022718.11	Dgcr8	-0.65027	3.83216	-4.06201	0.001056	0.030224	-0.85576	4.128990933	4.324237549	4.0113965	3.800073636	3.216111053	3.51215042
ENSMUSG00000048895.13	Cdk5r1	-0.65001	2.188449	-2.345	0.033467	0.196453	-3.7813	2.500570476	3.110642075	1.96844435	2.139162282	1.77419517	1.637681302
ENSMUSG00000074733.14	Zfp950	-0.64975	2.400883	-3.03577	0.008477	0.097312	-2.57854	2.743613407	2.571003565	2.82697193	2.3105307	1.77419517	2.178982574
ENSMUSG00000030243.16	Recql	-0.64872	2.021127	-2.4822	0.025621	0.175594	-3.50945	2.208119583	2.540946331	2.28082867	2.058991933	1.182834898	1.855038134
ENSMUSG00000032035.15	Ets1	-0.64866	2.168558	-2.41248	0.029359	0.185875	-3.66082	2.970790891	2.478885363	2.06056255	2.031246943	1.461810848	2.008049752
ENSMUSG00000023033.14	Scn8a	-0.64861	2.550913	-2.71789	0.01607	0.140485	-3.19656	2.787668587	2.982317978	2.80937787	2.378169417	2.619352125	1.728589799
ENSMUSG00000052331.14	Ankrd44	-0.64668	2.875191	-3.66215	0.002372	0.046124	-1.46706	3.294628576	3.247215128	3.06798002	2.620870821	2.388644435	2.631804736
ENSMUSG00000025571.13	Tnrc6c	-0.64604	4.604583	-4.79767	0.000246	0.015137	0.477744	4.843521822	5.148510261	4.78932464	4.429554803	4.335559159	4.081029198
ENSMUSG00000038146.7	Notch3	-0.64529	4.486696	-3.15308	0.006682	0.085893	-2.76283	4.695282157	5.138508869	4.56655822	4.717611476	4.059924716	3.742290709
ENSMUSG00000033439.12	Trmt13	-0.64521	1.401861	-2.33404	0.034183	0.198521	-3.64434	1.709666075	1.803980737	1.65047427	1.322026339	0.786906222	1.138110293
ENSMUSG00000040022.14	Rab11fip2	-0.64474	2.051082	-2.27361	0.038399	0.206263	-3.87231	2.361742772	2.120837843	2.49407271	2.400028851	1.695453194	1.234325608
ENSMUSG00000030279.15	C2cd5	-0.64301	3.214687	-3.22501	0.005774	0.0793	-2.37928	3.558934639	3.56354776	3.42065164	3.293113647	2.981922204	2.469953857
ENSMUSG00000038740.9	Mvb12b	-0.64207	3.709245	-3.49536	0.00333	0.05671	-1.95252	3.771305141	4.450870987	3.86579117	3.68013677	3.280445694	3.206919451
ENSMUSG00000032254.9	Kif23	-0.64137	2.5536	-2.43894	0.027883	0.181019	-3.69847	2.892257488	2.88995396	2.73677412	2.844592302	2.320338422	1.637681302
ENSMUSG00000056763.16	Cspp1	-0.64033	4.061745	-4.07913	0.001021	0.030012	-0.85992	4.283132937	4.214264707	4.58735418	4.034744387	3.751677733	3.499297818
ENSMUSG00000040563.13	Plppr2	-0.63998	1.470627	-2.38491	0.030976	0.189631	-3.572	1.663123489	1.752450437	1.93638314	1.275483753	0.931296131	1.265022405
ENSMUSG00000045333.15	Zfp423	-0.63974	2.935504	-2.35766	0.032657	0.194496	-3.93174	3.24808599	3.931092655	2.66032206	2.563914936	2.937009782	2.272595625
ENSMUSG00000034488.14	Edil3	-0.63959	2.376229	-3.00364	0.009047	0.100413	-2.63397	2.577876203	2.739275048	2.71803755	2.378169417	2.009298643	1.83471815
ENSMUSG00000027002.13	Nckap1	-0.63936	4.006923	-3.29662	0.004991	0.072879	-2.4014	4.244138805	4.654085307	4.05683947	4.142407941	3.608652236	3.335411733
ENSMUSG00000068923.14	Syt11	-0.63805	3.916125	-3.61842	0.002593	0.048656	-1.74856	4.401271136	4.418173949	3.89110226	3.895493201	3.315959981	3.574750047
ENSMUSG00000040537.17	Adam22	-0.63804	1.977889	-2.31833	0.035235	0.200644	-3.7815	2.30224176	1.901828061	2.57959078	2.164925378	1.257796956	1.660951082
ENSMUSG00000074794.9	Arrdc3	-0.63778	3.540248	-3.36543	0.004339	0.066555	-2.17392	4.343410949	3.622142414	3.60530795	3.245613223	3.324703614	3.10030826
ENSMUSG00000044617.7	Zbtb39	-0.63754	3.095167	-3.55945	0.002923	0.052789	-1.70582	3.467095772	3.533333147	3.22220094	2.995798107	2.818202352	2.534370585
ENSMUSG00000072082.6	Ccnf	-0.63722	2.870156	-2.96666	0.009749	0.104827	-2.80084	3.098708366	3.337412937	3.06798002	3.052105547	2.453862094	2.210866635
ENSMUSG00000000028.14	Cdc45	-0.63681	1.818434	-2.16952	0.046821	0.218613	-3.99479	2.240180792	1.525679575	2.51593215	1.944659258	1.220802748	1.463351122
ENSMUSG00000049932.3	H2afx	-0.63679	3.413123	-3.25986	0.005379	0.075998	-2.35378	3.63325296	4.165847577	3.42065164	3.350370965	2.842759456	3.065854204
ENSMUSG00000020032.14	Nuak1	-0.63668	2.347389	-3.08374	0.007692	0.092428	-2.47944	2.602750871	2.88995396	2.51593215	2.139162282	1.965243463	1.971291202
ENSMUSG00000058290.3	Espl1	-0.63525	3.005612	-2.52998	0.023325	0.16819	-3.641	3.520288711	3.502472184	2.89527794	3.32773999	2.485397668	2.302497111
ENSMUSG00000003992.14	Ssbp2	-0.63483	2.972869	-2.65437	0.018237	0.150647	-3.4063	3.520288711	3.247215128	3.02289214	3.20893256	2.675115396	2.162772347
ENSMUSG00000062510.12	Nsl1	-0.63429	1.609451	-2.15581	0.04805	0.220331	-3.9739	2.332298994	2.07961518	1.39138705	1.494493535	1.220802748	1.138110293
ENSMUSG00000029838.11	Ptn	-0.63377	3.615817	-2.54476	0.022656	0.166012	-3.75894	3.936957296	4.125908832	3.65540657	4.089580253	3.067758373	2.819288109
ENSMUSG00000032481.16	Smarcc1	-0.63367	5.089365	-4.95586	0.000182	0.012958	0.74454	5.363563207	5.655839343	5.20380111	4.897415513	4.71057253	4.704995415
ENSMUSG00000017550.14	Atad5	-0.63346	3.232889	-2.20094	0.044113	0.21442	-4.27682	3.584135193	3.533333147	3.40901694	3.529621758	3.262355237	2.07887109
ENSMUSG00000047246.6	Hist1h2be	-0.63335	2.960001	-2.3482	0.03326	0.196152	-3.95412	3.354437971	3.732641955	2.73677412	3.257635752	2.248636956	2.429876417
ENSMUSG00000033760.16	Rbm4b	-0.63156	2.621056	-2.87191	0.011802	0.117221	-2.92674	3.232232039	2.791269685	2.75527046	2.657629371	1.987439209	2.302497111
ENSMUSG00000041594.17	Tmtc4	-0.62995	2.232254	-2.84288	0.012511	0.121569	-2.90474	2.332298994	2.865908486	2.4493344	2.058991933	1.872879445	1.81410787
ENSMUSG00000031016.8	Wee1	-0.62965	2.308915	-3.02302	0.008699	0.098507	-2.5859	2.473853138	2.600447343	2.77353267	2.11293074	1.848833971	2.043894951
ENSMUSG00000068740.13	Celsr2	-0.62889	5.074148	-3.14594	0.00678	0.086485	-2.83978	5.413624563	5.770372345	4.9783191	5.15371059	4.791644141	4.33721849
ENSMUSG00000022546.4	Gpt	-0.62846	1.389413	-2.19667	0.044472	0.214943	-3.8651	1.404811494	1.643516065	1.99980852	1.322026339	0.931296131	1.0350168
ENSMUSG00000058230.12	Arhgap35	-0.62705	4.840732	-3.39213	0.004109	0.064415	-2.33161	5.14812792	5.44073254	4.86366173	4.889710848	4.621127749	4.081029198
ENSMUSG00000063450.14	Syne2	-0.62612	5.501193	-3.19006	0.006199	0.082246	-2.77719	5.769933106	6.093918718	5.57308922	5.610361462	5.314863292	4.644993508
ENSMUSG00000022246.13	Rai14	-0.62555	4.095991	-3.72876	0.002072	0.042576	-1.56092	4.408343185	4.709182983	4.10810814	4.041713942	3.777383447	3.531217152
ENSMUSG00000047888.9	Tnrc6b	-0.62511	4.794208	-4.30345	0.000651	0.023563	-0.5089	5.181534797	5.221389141	4.90566456	4.802127294	4.399031053	4.255499631
ENSMUSG00000026434.12	Nucks1	-0.625	5.476653	-4.31794	0.000633	0.023329	-0.52305	5.756140505	6.049241531	5.56262538	5.422897223	4.929864175	5.13914634
ENSMUSG00000033209.17	Ttc28	-0.6248	3.444581	-3.52447	0.003139	0.054506	-1.84253	3.966005828	3.822839764	3.46627426	3.405442347	3.078137519	2.928786474
ENSMUSG00000040721.9	Zfhx2	-0.62451	3.594121	-3.75687	0.001957	0.04175	-1.41455	3.825143293	4.270298741	3.65540657	3.372652412	3.187635884	3.25358751
ENSMUSG00000046785.8	Epm2aip1	-0.62407	3.170584	-2.88523	0.01149	0.115317	-3.02084	3.57158994	3.84760767	3.03807854	3.106297648	2.925559761	2.534370585
ENSMUSG00000040084.9	Bub1b	-0.62281	3.141415	-3.29992	0.004958	0.072785	-2.21902	3.439745284	3.678449855	3.22220094	3.106297648	2.854883015	2.546915839
ENSMUSG00000035877.17	Zhx3	-0.62235	2.804556	-2.99021	0.009296	0.101832	-2.74261	2.743613407	3.371970159	3.19535933	2.795957527	2.388644435	2.331791418

ENSMUSG00000020801.8	Med31	-0.62218	2.077282	-2.37393	0.031644	0.191705	-3.70757	2.107425583	2.237877264	2.71803755	2.031246943	1.987439209	1.381667324
ENSMUSG00000020899.15	Pfas	-0.62212	3.647532	-2.74278	0.015291	0.13661	-3.39784	3.994480997	4.185409844	3.64552505	3.832586449	3.449871234	2.777315838
ENSMUSG00000078532.9	Nkain1	-0.62164	2.802936	-3.44547	0.003686	0.060104	-1.87241	3.133265588	3.150999813	3.03807854	2.602134258	2.590640828	2.302497111
ENSMUSG00000051627.2	Hist1h1e	-0.62148	4.459949	-2.39335	0.030473	0.188438	-4.20204	4.822498571	5.356953124	4.13660674	4.726289367	3.869895586	3.847451639
ENSMUSG00000039801.6	2410089E03Ri	-0.62138	4.102784	-2.97953	0.009499	0.103548	-3.03888	4.394164249	4.418173949	4.37054279	4.129381261	4.121434353	3.183006831
ENSMUSG00000031822.18	Gse1	-0.62128	3.667617	-2.79399	0.013801	0.128301	-3.30599	4.040725182	4.46694575	3.477458	3.316289969	3.664822641	3.039462705
ENSMUSG00000022885.15	St6gal1	-0.62017	2.848847	-3.42439	0.003848	0.061912	-1.9214	3.263767612	3.170763068	3.03807854	2.71106863	2.3377205	2.571683744
ENSMUSG00000059878.12	Zfp422	-0.62004	3.321602	-3.04395	0.008338	0.096751	-2.75187	3.621129401	3.976715272	3.36151652	3.052105547	2.619352125	3.298793142
ENSMUSG00000090673.1	Gm340	-0.61886	2.758492	-3.08276	0.007708	0.092428	-2.55817	2.721070838	3.302007601	3.20884256	2.263609653	2.546473245	2.508947988
ENSMUSG00000021244.15	Ylpm1	-0.61823	4.725212	-4.10784	0.000963	0.028986	-0.89064	4.843521822	5.272586918	4.97437731	4.743490107	4.300529941	4.216767237
ENSMUSG00000001506.10	Col1a1	-0.61783	5.6102	-4.45739	0.00048	0.020447	-0.25105	5.730976581	6.269153291	5.76328863	5.300396323	5.188833638	5.408553312
ENSMUSG00000022816.11	Fstl1	-0.61764	4.242977	-4.13101	0.00092	0.028565	-0.78506	4.490609366	4.910669937	4.29037245	3.910800454	3.881794169	3.973616255
ENSMUSG00000025764.14	Jade1	-0.61658	2.692144	-2.91095	0.010909	0.111729	-2.86832	2.912295242	2.88995396	3.12598563	2.828562193	2.09356756	2.302497111
ENSMUSG00000064288.4	Hist1h4k	-0.6165	3.932084	-2.79211	0.013853	0.128445	-3.3658	4.267661528	4.709182983	3.75066856	4.076065446	3.376080974	3.41284322
ENSMUSG00000031558.15	Slit2	-0.61643	4.390312	-2.19537	0.044582	0.214964	-4.54124	4.630231608	4.939757516	4.46907833	4.704495976	4.407286841	3.191021824
ENSMUSG00000030172.15	Erc1	-0.61618	3.320046	-3.75311	0.001972	0.042003	-1.37001	3.467095772	3.859833972	3.55340685	3.132648975	3.057304016	2.849984906
ENSMUSG00000071337.11	Tia1	-0.61586	4.673924	-4.1368	0.000909	0.028367	-0.82774	5.047535714	4.904781378	4.96646126	4.609189016	4.514349042	4.001228487
ENSMUSG00000030677.8	Kif22	-0.61564	2.403584	-2.3648	0.032208	0.193215	-3.79413	2.932058497	2.629302206	2.49407271	2.76259893	1.748432074	1.855038134
ENSMUSG00000024151.13	Msh2	-0.61554	3.111988	-2.68268	0.017239	0.14641	-3.38653	3.480579001	3.63642664	3.12598563	3.32773999	2.302744362	2.798454606
ENSMUSG00000029730.16	Mcm7	-0.61526	4.263342	-3.33698	0.004597	0.069395	-2.36614	4.706800741	4.715924561	4.26478073	4.434858835	3.770999831	3.686688643
ENSMUSG00000029833.17	Trim24	-0.61459	3.820489	-4.07153	0.001036	0.030035	-0.83474	4.013156922	4.155966066	4.18516356	3.616209443	3.698850045	3.25358751
ENSMUSG00000026020.9	Nop58	-0.61435	3.906574	-3.6881	0.00225	0.044897	-1.60837	4.244138805	4.242553751	4.10810814	4.027740999	3.481493213	3.335411733
ENSMUSG00000016552.13	Foxred2	-0.61394	1.466741	-2.26825	0.038796	0.207079	-3.76392	1.615029201	1.993458536	1.76443046	0.899335267	1.29386621	1.234325608
ENSMUSG00000037606.17	Osbpl5	-0.61355	1.510868	-2.2548	0.039807	0.208663	-3.79401	1.798475342	2.037179913	1.61039683	1.494493535	1.020105398	1.104557733
ENSMUSG00000025880.11	Smad7	-0.61208	2.785773	-2.61379	0.019767	0.155682	-3.43784	3.216201929	3.388943238	2.66032206	2.922175243	2.284933085	2.242061257
ENSMUSG00000034730.16	Adgrb1	-0.61114	1.435758	-2.25975	0.039432	0.208097	-3.77187	1.709666075	1.525679575	1.96844435	1.07266687	1.103763327	1.234325608
ENSMUSG00000034329.16	Brip1	-0.60999	2.27192	-2.6427	0.018665	0.152281	-3.2755	2.743613407	2.765506589	2.22883404	2.11293074	2.05204829	1.728589799
ENSMUSG00000027379.13	Bub1	-0.60842	2.807062	-2.46157	0.026677	0.177985	-3.71653	3.199991702	2.913605228	3.1117014	2.922175243	2.780564695	1.914335318
ENSMUSG00000063160.12	Numbl	-0.60739	2.629915	-3.01867	0.008776	0.098962	-2.65387	3.081114306	3.150999813	2.62052105	2.263609653	2.302744362	2.360502714
ENSMUSG00000033767.14	Tmem131l	-0.60735	4.297535	-3.77042	0.001904	0.041006	-1.50972	4.624169855	4.632868821	4.52404479	4.135909304	4.26916577	3.599048643
ENSMUSG00000071855.4	Ccdc112	-0.60585	2.006341	-2.47398	0.026037	0.176459	-3.52086	2.271544963	2.540946331	2.2021167	1.275483753	1.872879445	1.875075887
ENSMUSG00000028654.13	Mycl	-0.6058	2.75553	-2.78035	0.014184	0.130453	-3.12516	3.081114306	3.319818877	2.77353267	2.828562193	2.113887544	2.416265986
ENSMUSG00000031826.19	Usp10	-0.60559	3.80318	-4.41661	0.00052	0.020975	-0.15559	4.085532977	4.13599765	4.07903521	3.689042323	3.376080974	3.453390012
ENSMUSG00000022893.14	Adamts1	-0.60289	2.694029	-2.94645	0.010155	0.107283	-2.80228	3.06330303	3.170763068	2.73677412	2.675663294	2.354895644	2.162772347
ENSMUSG00000069310.3	Hist1h3c	-0.60103	3.432416	-2.37418	0.031628	0.191705	-4.02403	3.814535193	4.233185568	3.15413667	3.662159029	2.890654146	2.83982504
ENSMUSG00000037784.14	Dzip1l	-0.60085	2.30636	-2.46925	0.026279	0.176849	-3.59073	2.851327224	2.685336241	2.25506558	2.333431102	2.05204829	1.660951082
ENSMUSG00000024665.7	Fads2	-0.59967	2.82659	-2.46677	0.026407	0.177356	-3.71219	3.383437843	3.337412937	2.6405588	2.90698884	2.561346521	2.129795025
ENSMUSG00000070047.12	Fat1	-0.5996	5.530384	-2.55198	0.022336	0.164957	-4.00862	5.896035828	6.159679569	5.4336295	5.523411266	5.627325283	4.542224143
ENSMUSG00000064061.13	Dzip3	-0.59827	3.770252	-3.61301	0.002621	0.048717	-1.73173	4.228241179	4.053239764	3.89944156	3.653085446	3.637010791	3.150494017
ENSMUSG00000029629.16	Phf14	-0.59804	4.387881	-4.5285	0.000417	0.018974	-0.02142	4.671965366	4.807141078	4.57178538	4.272524377	4.116407536	3.887459612
ENSMUSG00000026274.11	Pask	-0.59792	2.1293	-2.17603	0.046248	0.217853	-4.05209	2.446631669	2.380481659	2.37951295	2.086213402	2.248636956	1.234325608
ENSMUSG00000030091.17	Nup210	-0.5979	2.667316	-2.43836	0.027915	0.181137	-3.72575	3.167014379	3.405718951	2.37951295	2.524655505	2.284933085	2.242061257
ENSMUSG00000028799.15	Zfp362	-0.59789	3.118954	-3.00805	0.008967	0.099771	-2.7758	3.45348534	3.664577681	3.12598563	3.038233373	2.948369644	2.483069357
ENSMUSG00000034543.14	Morc2a	-0.59756	4.247125	-4.61997	0.000348	0.018054	0.175002	4.510463281	4.639975707	4.48578927	4.096290474	3.758147229	3.992083004
ENSMUSG00000036916.13	Zfp280c	-0.59711	2.588694	-2.59406	0.020554	0.158152	-3.4295	2.970790891	2.913605228	2.73677412	2.442778498	2.633496307	1.83471815
ENSMUSG00000020070.18	Rufy2	-0.59623	2.659614	-2.42826	0.028471	0.182623	-3.74119	2.765809153	2.913605228	3.08270153	2.844592302	2.516258632	1.83471815
ENSMUSG00000026458.13	Ppfia4	-0.59572	1.598998	-2.31235	0.035643	0.20128	-3.71581	1.663123489	1.901828061	2.09000633	1.410835606	1.29386621	1.234325608
ENSMUSG00000002870.8	Mcm2	-0.59566	3.950019	-2.92082	0.010694	0.110646	-3.12309	4.328575908	4.618549149	3.79604007	4.034744387	3.57240171	3.349802826
ENSMUSG00000038943.16	Prc1	-0.59489	2.510563	-2.29953	0.036534	0.202683	-3.93099	2.743613407	3.004513725	2.60020106	2.891640875	2.009298643	1.81410787
ENSMUSG00000069769.13	Msi2	-0.59388	4.772722	-3.69221	0.002231	0.044692	-1.72563	5.285055249	5.192677844	4.73444531	4.566333537	4.676823739	4.180996106
ENSMUSG00000024378.9	Stard4	-0.59361	2.325167	-2.77215	0.014418	0.132159	-3.05165	2.698170436	2.865908486	2.35546747	1.914602024	2.07295728	2.043894951
ENSMUSG00000039985.14	Fam60a	-0.5935	2.595575	-2.51569	0.02399	0.170562	-3.57181	2.851327224	3.283973678	2.53746531	2.71106863	2.09356756	2.096046233
ENSMUSG00000028633.7	Ctps	-0.59251	2.831365	-2.62376	0.01938	0.154432	-3.42995	2.787668587	3.607715344	2.97634955	2.876127876	2.437831985	2.302497111
ENSMUSG00000037111.9	Setd7	-0.59216	3.18766	-3.42319	0.003857	0.06192	-1.98897	3.533286052	3.533333147	3.40901694	2.922175243	2.619352125	3.108794726
ENSMUSG00000044330.8	Gm9790	-0.59185	2.573809	-2.61875	0.019574	0.155099	-3.38196	2.912295242	2.936875008	2.844354	1.719785847	2.546473245	2.483069357
ENSMUSG00000046179.17	E2f8	-0.59175	2.45986	-2.41224	0.029373	0.185875	-3.72425	2.332298994	2.685336241	3.23543677	2.058991933	2.030831804	2.416265986
ENSMUSG00000033436.13	Armcx2	-0.59167	3.664579	-3.26361	0.005338	0.075795	-2.3986	3.985051584	4.2047104	3.67496883	3.724124782	3.298312115	3.10030826
ENSMUSG00000027306.15	Nusap1	-0.59124	1.896555	-2.35746	0.03267	0.194496	-3.69843	2.107425583	2.274871471	2.14715024	2.031246943	1.329055639	1.489582663
ENSMUSG00000039089.14	L3mbtl3	-0.59096	3.037791	-3.2428	0.005568	0.077675	-2.30802	3.183597264	3.588943238	3.33716666	2.966798235	2.830533154	2.469953857
ENSMUSG00000039262.16	Prrc2b	-0.59079	6.007308	-4.16659	0.000856	0.027359	-0.83544	6.262792489	6.595886179	6.05030086	5.930647865	5.651852565	5.552370269
ENSMUSG00000038195.6	Rilp	-0.59034	1.456919	-2.2546	0.039823	0.208663	-3.78404	1.663123489	1.699011178	1.87004065	1.227389465	1.143840766	1.138110293
ENSMUSG00000032009.7	Sesn3	-0.59032	2.377072	-2.74937	0.015091	0.135634	-3.10341	2.500570476	2.936875008	2.62052105	2.058991933	1.872879445	2.272595625
ENSMUSG00000023015.13	Racgap1	-0.58995	3.382492	-2.71866	0.016046	0.140453	-3.38215	3.680753383	3.705800343	3.56393752	3.587920399	3.197190191	2.559352942
ENSMUSG00000043909.16	Trp53bp1	-0.58947	5.135972	-4.14097	0.000901	0.028196	-0.85825	5.438018539	5.655839343	5.20380111	4.862415804	5.061233283	4.59452372
ENSMUSG00000023495.12	Pcbp4	-0.58928	4.613803	-3.58104	0.002797	0.05121	-1.93023	5.038447841	5.244886322	4.46907833	4.380920028	4.223142887	4.326343853
ENSMUSG00000008305.18	Tle1	-0.58914	3.932757	-4.26894	0.000698	0.024305	-0.46512	4.372631088	4.29752021	4.02670375	3.625517186	3.705560266	3.56861091
ENSMUSG00000022686.14	B3gnt5	-0.5889	2.44553	-2.63316	0.019022	0.153491	-3.32798	2.871937504	2.571003565	2.69905445	2.583151155	2.07295728	1.875075887
ENSMUSG00000080316.10	Spaca6	-0.58837	3.468067	-2.84338	0.012499	0.121569	-3.16695	3.782234701	3.784871914	3.64552505	3.791829642	2.914018139	2.889923652
ENSMUSG00000106106.2	#N/A	-0.58763	16.32211	-2.53359	0.02316	0.167539	-3.7638	16.65865825	17.06559307	16.1195088	16.12790054	16.37291419	15.58809717
ENSMUSG00000005533.9	Igf1r	-0.58756	4.162601	-2.67507	0.017502	0.147524	-3.63163	4.283132937	4.844534836	4.26478073	3.715433822	4.427721957	3.440000635
ENSMUSG00000041642.18	Kif21b	-0.58678	4.257679	-4.43478	0.000502	0.020658	-0.1881	4.477219989	4.788074346	4.41193443	3.887778236	4.054678546	3.926387978
ENSMUSG00000030852.15	Tacc2	-0.58659	3.023697	-3.11399	0.007234	0.090084	-2.55291	3.411866251	3.593142542	2.97634955	2.828562193	2.688725828	2.643534029
ENSMUSG00000002297.15	Dbf4	-0.58595	3.192256	-3.29136	0.005045	0.073296	-2.24593	3.703929706	3.4549065	3.2871982	3.132648975	2.675115396	2.899737941
ENSMUSG00000032024.10	Clmp	-0.58537	2.861238	-2.32086	0.035064	0.200218	-3.978	3.045269107	3.883980059	2.6405588	2.504617752	2.437831985	2.655168729
ENSMUSG00000020914.17	Top2a	-0.58474	5.351999	-2.9437	0.010212	0.107613	-3.25369	5.649758231	5.864392254	5.40755599	5.631304177	4.955424402	4.603559418
ENSMUSG00000041540.16	Sox5	-0.58451	2.110799	-2.56011	0.021981	0.163601	-3.39055	2.526802018	2.510249534	2.22883404	1.534570974	2.009298643	1.855038134
ENSMUSG00000030729.16	Pgm2l1	-0.58412	3.024815	-3.35977	0.004389	0.067156	-2.07969	3.493937384	3.405718951	3.08270153	2.675663294	2.767797399	2.723072794
ENSMUSG00000029587.16	Zfp12	-0.58263	2.758205	-2.44171	0.027733	0.180541	-3.74091	3.263767612	3.354795016	2.60020106	2.215111135	2.842759456	2.272595625
ENSMUSG00000066551.12	Hmgb1	-0.58224	4.330782	-4.11332	0.000953	0.028907	-0.83227	4.735201167	4.709182983	4.41775175	4.272524377	3.839712581	4.010316359
ENSMUSG00000033697.15	Arhgap39	-0.58178	3.290742	-3.64247	0.002469	0.047032	-1.58076	3.546167343	3.388943238	3.76898874	3.065845604	3.036165249	2.938340781
ENSMUSG00000067586.3	S1pr3	-0.58121	2.582854	-3.02606	0.008646	0.098298	-2.63054	2.809201749	2.982317978	2.80937787	2.463687488	2.302744362	2.129795025
ENSMUSG00000027750.16	Postn	-0.58117	3.575655	-2.68407	0.017191	0.146277	-3.49142	3.715379726	3.942634277	3.89110226	3.840601442	2.728803267	3.335411733
ENSMUSG00000024370.16	Cdc23	-0.57991	3.458075	-3.80804	0.001764	0.039193	-1.28792	3.63325296	3.919457955	3.68465139	3.316289969	3.12894189	3.065854204
ENSMUSG00000021846.8	Peli2	-0.57754	2.604068	-2.55102	0.022378	0.16509	-3.51048	3.008510557	3.247215128	2.4493344	2.504617752	2.284933085	2.129795025
ENSMUSG00000026605.14	Cenpf	-0.57745	4.296141	-2.73453	0.015545	0.138146	-3.54239	4.689488193	4.529511436	4.46907833	4.664418536	3.899460044	3.524889532
ENSMUSG00000054752.16	Fsd1l	-0.57651	2.946281	-2.63969	0.018777	0.152709	-3.42695	3.369010772	3.228478566	3.06798002	3.145646315	2.21140405	2.655168729
ENSMUSG00000038886.10	Man2a2	-0.57639	3.820627	-3.66801	0.002344	0.045917	-1.63217	4.049798765	4.063847864	4.17832613	3.616209443	3.777383447	3.238198617
ENSMUSG00000049606.16	Zfp644	-0.57611	4.405679	-3.82791	0.001695	0.038362	-1.41048	4.671965366	4.880983807	4.52942799	4.369885978	3.808884591	4.172925064
ENSMUSG00000031403.14	Dkc1	-0.57371	2.714719	-2.54551	0.022623	0.165978	-3.54507	2.500570476	3.337412937	3.14012981	2.69347457	2.284933085	2.331791418
ENSMUSG00000060261.15	Gtf2i	-0.57173	5.267205	-3.97021	0.001271	0.032923	-1.20695	5.58881168	5.598624608	5.46205791	5.362946594	4.737014416	4.853776485
ENSMUSG00000036181.2	Hist1h1c	-0.57161	4.904704	-3.00179	0.009081	0.100476	-3.10789	5.307925399	5.504420455	4.76214591	5.011857957	4.287171558	4.554701584
ENSMUSG00000037235.13	Mxd4	-0.57056	3.455686	-3.43774	0.003745	0.060688	-2.01436	3.793082083	3.517985183	3.86579117	3.405442347	2.937009782	3.21480305
ENSMUSG00000038267.14	Slc22a23	-0.57026	2.044847	-2.29245	0.037034	0.203267	-3.839	2.208119583	2.120837843	2.57959078	1.914602024	2.009298643	1.436633784
ENSMUSG00000019960.7	Dusp6	-0.56965	2.73367	-2.34928	0.033191	0.196035	-3.89755	3.216201929	3.388943238	2.47187697	2.76259893	2.320338422	2.242061257
ENSMUSG00000060534.15	Dcc	-0.5695	4.354106	-2.5826	0.021025	0.159596	-3.83772	4.581009092	5.018214635	4.33409382	4.027740999	4.588827258	3.574750047
ENSMUSG00000055612.15	Cdca7	-0.56943	2.647734	-2.92725	0.010556	0.109812	-2.82887	2.932058497	3.209495462	2.69905445	2.333431102	2.3377205	2.374646897
ENSMUSG00000041491.7	Cep78	-0.5689	2.054688	-2.20954	0.043397	0.213471	-3.97997	2.446631669	2.310940726	2.2021167	2.3105307	1.396959703	1.660951082
ENSMUSG00000007850.16	Hnrnph1	-0.56851	6.628864	-4.63932	0.000336	0.017796	0.104611	6.974964841	6.91140432	6.85564823	6.608602435	6.207272787	6.215294347
ENSMUSG00000099597.1	#N/A	-0.56755	1.755971	-2.26695	0.038892	0.207284	-3.82191	1.999172692	2.310940726	1.83568914	1.453270872	1.61222769	1.324523417
ENSMUSG00000005370.3	Msh6	-0.56665	3.782634	-3.50147	0.003289	0.056198	-1.95423	4.111764519	4.125908832	3.93232493	3.864382668	3.262355237	3.399070371
ENSMUSG00000005893.14	Nr2c2	-0.56631	3.133056	-3.17629	0.006375	0.083375	-2.45595	3.150238667	3.622142414	3.46627426	3.038233373	2.661375339	2.860073724
ENSMUSG00000021215.14	Net1	-0.56525	3.103242	-3.47339	0.003483	0.058241	-1.87445	3.24808599	3.502472184	3.39728765	2.90698884	2.830533154	2.734085786
ENSMUSG00000075015.3	Gm10801	-0.56478	5.811597	-2.15997	0.047674	0.219843	-4.72164	6.201010195	6.312053487	5.77466641	5.231967031	6.379756632	4.970127269
ENSMUSG00000069135.11	Fgfr1op	-0.56477	2.82686	-3.18388	0.006277	0.082773	-2.37885	3.045269107	3.265711472	3.02289214	2.563914936	2.633496307	2.429876417
ENSMUSG00000040591.18	1110051M20F	-0.56319	2.186463	-2.46957	0.026263	0.176849	-3.56457	2.208119583	2.540946331	2.60020106	2.190236466	1.872879445	1.706394053
ENSMUSG00000028999.15	Rint1	-0.56315	2.334079	-2.66758	0.017765	0.148886	-3.24304	2.698170436	2.712557709	2.4493344	2.190236466	1.824380927	2.129795025
ENSMUSG00000001555.10	Fkbp10	-0.56211	1.817608	-2.19937	0.044245	0.214663	-3.94598	1.999172692	2.478885363	1.83568914	1.684596418	1.55392905	1.35337828
ENSMUSG00000028986.12	Klhl7	-0.56145	3.584452	-3.70768	0.002162	0.043866	-1.5092	3.680753383	4.105517043	3.81379654	3.394594965	3.289406562	3.222643803
ENSMUSG00000029127.15	Zbtb49	-0.55884	2.212555	-2.23911	0.041019	0.210441	-3.96602	2.602750871	2.816580774	2.11886119	1.719785847	2.266899163	1.750449233
ENSMUSG00000032547.12	Ryk	-0.55848	2.621086	-2.31954	0.035153	0.200388	-3.92234	3.008510557	3.209495462	2.47187697	2.69347457	2.371868722	1.971291202
ENSMUSG00000043467.19	Zbtb37	-0.55837	3.072996	-2.36504	0.032194	0.193215	-3.9518	3.57158994	3.63642664	2.844354	3.106297648	2.890654146	2.388653755
ENSMUSG00000095139.2	Pou3f2	-0.5582	3.97092	-2.67541	0.01749	0.147515	-3.59332	4.415380735	4.306481078	3.98812672	4.027740999	3.945538053	3.142250023
ENSMUSG00000004113.18	Cacna1b	-0.55779	2.777751	-2.1388	0.049617	0.222531	-4.26808	2.989773994	3.705800343	2.57959078	2.190236466	2.854883015	2.346218488
ENSMUSG00000036591.15	Arhgap21	-0.55726	4.572871	-3.7921	0.001822	0.039821	-1.50341	4.763053288	4.928192765	4.85511241	4.319161005	4.588827258	3.982879177
ENSMUSG00000048388.3	Fam171b	-0.55674	2.514165	-2.22243	0.042345	0.212176	-4.06333	2.970790891	3.170763068	2.25506558	2.524655505	2.192420947	1.971291202
ENSMUSG00000042590.16	Ipo11	-0.55557	3.359064	-2.97778	0.009533	0.103577	-2.88611	3.520288711	3.664577681	3.66522086	3.361554703	3.253224226	2.689520234

ENSMUSG00000048732.5	Klhl11	-0.55537	1.94275	-2.23205	0.041575	0.211556	-3.91879	2.30224176	2.540946331	1.90359321	1.410835606	1.748432074	1.750449233
ENSMUSG00000036564.16	Ndrg4	-0.55472	2.037273	-2.5324	0.023214	0.167585	-3.42456	2.30224176	2.414034219	2.22883404	1.820479846	1.77419517	1.683851484
ENSMUSG00000035133.8	Arhgap5	-0.55444	4.107484	-3.19427	0.006146	0.081899	-2.62042	4.536517173	4.458930757	4.1295347	4.230442789	3.764587842	3.524889532
ENSMUSG00000079036.10	Alkbh1	-0.55358	3.160208	-3.12635	0.007055	0.088728	-2.55763	3.493937384	3.405718951	3.37353905	3.221263362	2.647503165	2.819288109
ENSMUSG00000027778.15	Ift80	-0.55295	2.541433	-2.31558	0.035422	0.20128	-3.91033	2.970790891	2.88995396	2.53746531	2.728450708	2.266899163	1.855038134
ENSMUSG00000025747.12	Tyms	-0.55166	2.679344	-2.81267	0.013294	0.126069	-3.04858	2.787668587	3.110642075	2.94446549	2.675663294	2.21140405	2.346218488
ENSMUSG00000018548.15	Trim37	-0.55012	3.259848	-2.87366	0.01176	0.116967	-3.06207	3.533286052	3.486790561	3.52134564	3.468867727	2.688725828	2.860073724
ENSMUSG00000074657.4	Kif5a	-0.55007	2.49188	-2.18114	0.045802	0.217545	-4.12749	2.787668587	3.110642075	2.426434	2.164925378	2.647503165	1.81410787
ENSMUSG00000028483.13	Snapc3	-0.54968	3.162539	-2.90318	0.011082	0.112483	-2.98413	3.57158994	3.170763068	3.48855571	3.245613223	2.866905544	2.631804736
ENSMUSG00000035024.15	Ncapd3	-0.54952	3.243646	-2.22832	0.041873	0.212176	-4.23193	3.520288711	3.692189912	3.26154961	3.643954434	2.914018139	2.429876417
ENSMUSG00000034205.16	Loxl2	-0.54932	1.881521	-2.34331	0.033576	0.196627	-3.71937	2.107425583	2.380481659	1.99980852	1.648527164	1.61222769	1.540656848
ENSMUSG00000003534.17	Ddr1	-0.54888	4.278356	-2.89969	0.01116	0.113105	-3.22372	4.517020997	4.874972569	4.25831124	4.290190252	4.161030528	3.56861091
ENSMUSG00000028184.12	Adgrl2	-0.5485	5.121334	-3.92322	0.001397	0.034469	-1.29216	5.515384361	5.29527127	5.36000663	5.191803761	4.720545116	4.644993508
ENSMUSG00000037544.13	Dlgap5	-0.54768	2.374855	-2.51547	0.024001	0.170552	-3.52345	2.390597634	2.816580774	2.69905445	2.400028851	2.009298643	1.933571536
ENSMUSG00000032640.10	Chsy1	-0.54728	2.774087	-2.19641	0.044494	0.214943	-4.1698	3.045269107	3.405718951	2.67981823	3.052105547	2.07295728	2.388653755
ENSMUSG00000029916.11	Agk	-0.54691	2.470151	-2.37782	0.031406	0.190953	-3.78613	2.473853138	2.816580774	2.87850223	2.400028851	2.437831985	1.81410787
ENSMUSG00000025321.14	Itgb8	-0.54614	3.560444	-2.52119	0.023732	0.169665	-3.78845	3.877044395	3.678449855	3.88271448	3.824526679	2.728803267	3.37112383
ENSMUSG00000063894.14	Zkscan8	-0.54591	2.470533	-2.43647	0.028018	0.181318	-3.68366	2.627203915	2.765506589	2.77353267	2.675663294	1.919800492	2.061489011
ENSMUSG00000056394.17	Lig1	-0.54571	3.959787	-3.58027	0.002802	0.051221	-1.83128	4.146014081	4.359108008	4.16455328	3.970455258	3.692108467	3.426485828
ENSMUSG00000032740.16	Ccdc88a	-0.54548	4.638491	-4.11021	0.000959	0.028977	-0.87641	4.874493209	4.89293166	4.94244914	4.627834768	4.365527195	4.127708698
ENSMUSG00000018401.17	Mtmr4	-0.54505	4.283986	-3.47004	0.003506	0.058449	-2.10531	4.477219989	4.904781378	4.30927265	4.020703448	4.1804284	3.811510758
ENSMUSG00000048402.14	Gli2	-0.54502	2.756586	-2.30806	0.035939	0.202085	-3.97425	3.116090444	3.283973678	2.66032206	2.844592302	2.590640828	2.043894951
ENSMUSG00000029635.15	Cdk8	-0.54443	4.17492	-3.95447	0.001312	0.033365	-1.12439	4.436289725	4.359108008	4.52404479	4.096290474	3.664822641	3.968962396
ENSMUSG00000045672.15	Col27a1	-0.54392	2.847656	-2.80887	0.013395	0.126604	-3.0919	3.167014379	3.354795016	2.844354	2.795957527	2.453862094	2.469953857
ENSMUSG00000021597.16	Slf1	-0.54295	3.012402	-2.95383	0.010005	0.106218	-2.85532	3.279280612	3.422301835	3.14012981	3.024226515	2.485397668	2.723072794
ENSMUSG00000041879.13	Ipo9	-0.54238	5.11808	-4.2879	0.000672	0.023982	-0.56456	5.381642329	5.508309124	5.27282702	5.094615843	4.762980382	4.688105133
ENSMUSG00000060098.11	Prmt7	-0.54221	2.466344	-2.64307	0.018652	0.152281	-3.31465	2.871937504	2.88995396	2.49407271	2.058991933	2.320338422	2.162772347
ENSMUSG00000036875.15	Dna2	-0.54146	2.518435	-2.14784	0.048778	0.221249	-4.18909	2.871937504	3.090031796	2.37951295	2.76259893	2.07295728	1.933571536
ENSMUSG00000001761.7	Smo	-0.54038	3.667308	-3.1338	0.006949	0.087573	-2.65186	3.975560135	3.919457955	3.86579117	3.840601442	3.187635884	3.21480305
ENSMUSG00000078584.9	AU022252	-0.53841	3.212457	-3.12154	0.007124	0.089222	-2.57849	3.480579001	3.719283573	3.31239875	2.71106863	2.959640757	3.091771578
ENSMUSG00000028354.13	Fmn2	-0.53589	2.798207	-2.61873	0.019574	0.155099	-3.43115	2.951554672	3.228478566	2.99203117	2.657629371	2.780564695	2.178982574
ENSMUSG00000035455.12	Fignl1	-0.53571	2.285802	-2.40893	0.029563	0.186335	-3.69142	2.627203915	2.629302206	2.40316422	2.263609653	1.695483194	2.096046233
ENSMUSG00000030231.10	Plekha5	-0.53564	3.877817	-3.28543	0.005106	0.073913	-2.39818	4.306033339	4.2047104	3.90773294	3.848572153	3.67169263	3.328161994
ENSMUSG00000022914.15	Brwd1	-0.53555	4.912837	-3.25971	0.00538	0.075998	-2.60094	5.122555108	5.123375565	5.27282702	4.87417697	4.895063814	4.189022246
ENSMUSG00000048490.13	Nrip1	-0.53472	3.594651	-3.72113	0.002104	0.043105	-1.48439	4.040725182	3.732641955	3.80494562	3.361554703	3.307163033	3.32087564
ENSMUSG00000004891.16	Nes	-0.53439	4.987798	-3.71237	0.002142	0.043616	-1.70453	5.34156489	5.44073254	4.98617052	4.946517204	4.48734594	4.724454024
ENSMUSG00000089855.1	#N/A	-0.53351	4.831732	-2.33843	0.033895	0.197603	-4.34837	5.113929103	4.933986728	5.19705164	5.13428006	4.743549852	3.867594306
ENSMUSG00000021488.7	Nsd1	-0.53309	6.133392	-3.74285	0.002013	0.042282	-1.68454	6.493106099	6.507337939	6.19958639	6.194151516	5.630854814	5.775318107
ENSMUSG00000061046.9	Haghl	-0.53275	2.873335	-2.95964	0.009889	0.105743	-2.8146	3.183597264	3.209495462	3.06798002	2.355973671	2.767797399	2.655168729
ENSMUSG00000020167.14	Tcf3	-0.53267	4.67493	-4.43608	0.000501	0.020653	-0.23498	4.973177484	5.012749875	4.83351477	4.580760607	4.304955389	4.344422975
ENSMUSG00000003282.9	Plag1	-0.53124	1.938289	-2.173	0.046514	0.217853	-4.01437	2.240180792	1.993458536	2.30613976	1.883905227	1.824380927	1.381667324
ENSMUSG00000021540.16	Smad5	-0.53033	4.270205	-3.59012	0.002746	0.050549	-1.8642	4.283132937	4.838369463	4.49131685	4.15531805	3.851861663	4.001228487
ENSMUSG00000048285.8	Frmd6	-0.53017	3.044431	-3.14414	0.006805	0.086635	-2.49899	3.116090444	3.388943238	3.42065164	2.812351965	2.688725828	2.83982504
ENSMUSG00000024913.16	Lrp5	-0.52997	3.486073	-3.03057	0.008567	0.097851	-2.81301	3.680753383	4.074378531	3.51049826	3.458488581	3.02547857	3.166842012
ENSMUSG00000034235.16	Usp54	-0.5293	3.016847	-2.64228	0.018681	0.152281	-3.43814	3.008510557	3.371970159	3.42065164	2.844592302	3.02547857	2.429876417
ENSMUSG00000026455.14	Klhl12	-0.52881	2.727922	-2.87844	0.011648	0.116121	-2.93621	2.830418235	3.090031796	3.03807854	2.620870821	2.371868722	2.416265986
ENSMUSG00000022533.13	Atp13a3	-0.52817	4.516813	-4.20334	0.000796	0.026202	-0.67744	4.784954202	4.742580193	4.80272438	4.429554803	4.044128627	4.296937586
ENSMUSG00000036792.12	Mbd5	-0.52743	3.455655	-3.25644	0.005416	0.076344	-2.36823	3.669024091	3.63642664	3.79604007	3.468867727	3.187635884	2.975937567
ENSMUSG00000031134.16	Rbmx	-0.52737	4.162446	-3.56882	0.002868	0.052286	-1.88913	4.483930211	4.521837508	4.25831124	4.10296963	3.984683344	3.622944759
ENSMUSG00000005225.15	Plekha8	-0.52697	2.766078	-2.35961	0.032534	0.194203	-3.88692	2.970790891	3.502472184	2.6405588	2.745625852	2.405227319	2.331791418
ENSMUSG00000032198.8	Dock6	-0.52572	3.235411	-3.10485	0.00737	0.090645	-2.615	3.703929706	3.388943238	3.37353905	3.132648975	3.02547857	2.787923938
ENSMUSG00000089824.10	Rbm12	-0.52545	3.257994	-2.79601	0.013745	0.128071	-3.20792	3.57158994	3.354795016	3.57439188	3.405442347	2.675115396	2.966629825
ENSMUSG00000049823.9	Zbtb12	-0.52533	2.393828	-2.51746	0.023907	0.170415	-3.52412	2.602750871	2.936875008	2.4493344	2.239564179	2.07295728	2.061489011
ENSMUSG00000038718.15	Pbx3	-0.52512	3.209119	-2.50682	0.024412	0.171439	-3.73064	3.279280612	3.622142414	3.44364329	3.499564524	2.754916107	2.655168729
ENSMUSG00000053774.8	Ubxn7	-0.52448	4.774431	-4.03617	0.001113	0.030999	-1.03797	5.047535714	5.207104915	4.8593934	4.682368317	4.326881267	4.523303281
ENSMUSG00000036473.15	Tbc1d24	-0.5236	3.183179	-3.22021	0.00583	0.07959	-2.38149	3.507173211	3.56354776	3.2744309	2.952076729	2.992935197	2.808908963
ENSMUSG00000000561.14	Wdr77	-0.52264	2.593345	-2.52934	0.023355	0.16819	-3.54654	2.892257488	2.791269685	2.87850223	2.463687488	1.987439209	2.546915839
ENSMUSG00000063785.12	Utp14a	-0.52173	2.660585	-2.45331	0.027112	0.179305	-3.69704	2.970790891	2.571003565	3.20884256	2.139162282	2.590640828	2.483069357
ENSMUSG00000029290.12	Zfp326	-0.52118	3.452099	-2.83609	0.012683	0.122354	-3.17667	3.45348534	3.919457955	3.72274456	3.587920399	3.02547857	3.003505827
ENSMUSG00000063052.9	Lrrc40	-0.5204	3.085808	-3.24856	0.005504	0.077092	-2.30657	3.397722069	3.388943238	3.2744309	2.745625852	2.818202352	2.889923652
ENSMUSG00000029033.16	Acap3	-0.52021	2.697556	-2.81181	0.013317	0.126119	-3.05387	3.008510557	3.026373159	2.82697193	2.583151155	2.437831985	2.302497111
ENSMUSG00000032657.15	Fam189b	-0.52007	2.020501	-2.23891	0.041035	0.210441	-3.92358	2.446631669	2.274871471	2.11886119	1.944659258	1.523871816	1.81410787
ENSMUSG00000042207.17	Kdm5b	-0.52001	5.512705	-2.80436	0.013517	0.126608	-3.53018	5.952764479	5.789672901	5.57308922	5.368500769	5.662237937	4.72996576
ENSMUSG00000038538.17	Ubn2	-0.51974	5.126463	-4.35792	0.000585	0.022349	-0.42613	5.441469964	5.395257012	5.3201845	4.893568324	5.040152432	4.668146583
ENSMUSG00000038290.15	Smg6	-0.51943	3.787046	-3.54282	0.003024	0.05369	-1.87324	4.111764519	4.2047104	3.84002808	3.529621758	3.644014179	3.392134323
ENSMUSG00000038545.13	Cul7	-0.51881	4.352033	-3.08292	0.007705	0.092428	-2.87955	4.735201167	4.60408592	4.46907833	4.324885993	4.309367304	3.669581387
ENSMUSG00000064339.1	mt-Rnr2	-0.51827	12.07972	-2.27684	0.038162	0.20566	-4.35045	12.36403217	12.86017872	11.7838628	11.73402483	12.21454069	11.52170287
ENSMUSG00000042197.13	Zfp451	-0.51825	4.759224	-3.85771	0.001595	0.037037	-1.39271	4.996828752	4.996230285	5.03996565	4.814388233	4.407286841	4.300646315
ENSMUSG00000021704.7	Mtx3	-0.51746	2.695112	-2.41573	0.029174	0.185276	-3.77094	2.830418235	2.816580774	3.12598563	2.891640875	2.248636956	2.257409221
ENSMUSG00000093930.1	Hmgcs1	-0.51557	5.222125	-4.17268	0.000846	0.027304	-0.79991	5.40657842	5.730978906	5.30770706	5.046919101	4.946954544	4.893611839
ENSMUSG00000010663.4	Fads1	-0.51545	3.981227	-3.66477	0.002359	0.046085	-1.66745	4.275417972	4.393155489	4.04936435	3.895493201	3.650983734	3.622944759
ENSMUSG00000037674.15	Rfx7	-0.5137	3.585436	-3.01375	0.008864	0.099093	-2.86665	3.782234701	4.010009712	3.70382369	3.68013677	3.253224226	3.083184082
ENSMUSG00000020241.13	Col6a2	-0.51362	2.949305	-2.96452	0.009791	0.105112	-2.82129	3.150238667	3.209495462	3.26154961	2.563914936	2.890654146	2.619979301
ENSMUSG00000003929.10	Zfp81	-0.51279	2.164495	-2.21529	0.042925	0.212526	-3.99439	2.30224176	2.478885363	2.4493344	2.239564179	1.583372827	1.933571536
ENSMUSG00000041936.18	Agrn	-0.51206	6.010686	-3.74676	0.001998	0.042282	-1.67625	6.180502457	6.534286995	6.08727136	5.845588295	5.898728268	5.517737628
ENSMUSG00000046982.10	Tshz1	-0.51206	3.548466	-3.16891	0.006471	0.084387	-2.55832	3.692388084	3.987899011	3.71331514	3.578366092	3.168335328	3.150494017
ENSMUSG00000031229.16	Atrx	-0.5097	6.030342	-3.85339	0.001609	0.037161	-1.4624	6.314528133	6.164616084	6.37578216	6.0091937	5.838949855	5.478983556
ENSMUSG00000029394.11	Cdk2ap1	-0.50834	2.413554	-2.58747	0.020824	0.158953	-3.4033	2.674900657	2.600447343	2.73677412	2.031246943	2.21140405	2.226548258
ENSMUSG00000033105.12	Lss	-0.50795	3.49546	-3.05475	0.008158	0.095707	-2.76775	3.703929706	3.607715344	3.87427764	3.468867727	3.342033493	2.975937567
ENSMUSG00000022865.13	Cxadr	-0.50708	3.299258	-2.82411	0.012992	0.124439	-3.16478	3.793082083	3.650570823	3.22220094	3.233489664	2.948369644	2.94783223
ENSMUSG00000023191.9	P3h3	-0.5038	2.719461	-2.52251	0.023671	0.169529	-3.58683	3.116090444	2.841455443	2.91186082	2.620870821	2.647503165	2.178982574
ENSMUSG00000024978.10	Gpam	-0.50372	2.023036	-2.1415	0.049366	0.222295	-4.08459	2.208119583	2.446824154	2.14715024	2.086213402	1.61222769	1.637681302
ENSMUSG00000026974.11	Zmynd19	-0.50294	2.758992	-2.51266	0.024134	0.170977	-3.61368	3.027006901	3.069122806	2.91186082	2.844592302	2.192420947	2.508947988
ENSMUSG00000055013.14	Agap1	-0.50245	4.433624	-3.37802	0.004229	0.065579	-2.31008	4.660164114	5.029082446	4.38842767	4.272524377	4.111363143	4.140181643
ENSMUSG00000001313.12	Rnd2	-0.50231	2.632759	-2.2819	0.037793	0.204907	-3.98994	2.989773994	2.816580774	2.91186082	2.031246943	2.248636956	2.798454606
ENSMUSG00000001518.12	Itfg2	-0.50226	2.37616	-2.26023	0.039396	0.208097	-3.96624	2.698170436	2.274871471	2.82697193	2.421562012	2.009298643	2.026083675
ENSMUSG00000023832.13	Acat2	-0.502	2.993591	-2.72254	0.015922	0.139769	-3.28472	3.24808599	3.502472184	2.99203117	2.922175243	2.688725828	2.608056133
ENSMUSG00000026565.18	Pou2f1	-0.5011	3.447678	-3.06721	0.007954	0.094099	-2.73364	3.760292148	3.84760767	3.48855571	3.416208778	3.014712139	3.15869117
ENSMUSG00000074505.4	Fat3	-0.50093	3.939519	-2.38064	0.031234	0.190341	-4.12735	4.422384123	4.074378531	4.00368153	4.236530119	3.685335238	3.21480305
ENSMUSG00000040651.8	Fam208a	-0.50063	4.241936	-3.14041	0.006857	0.087016	-2.74883	4.618082525	4.434615096	4.40022893	4.341925896	3.650983734	4.005779579
ENSMUSG00000016181.9	Diexf	-0.49978	2.527227	-2.45387	0.027081	0.179194	-3.66532	2.871937504	2.629302206	2.77353267	2.583151155	2.192420947	2.113019312
ENSMUSG00000028568.16	Btf3l4	-0.49944	2.687851	-2.61671	0.019653	0.155404	-3.41001	3.150238667	2.95977541	2.71803755	2.504617752	2.3377205	2.45671803
ENSMUSG00000096210.1	H1f0	-0.49905	4.428899	-3.51543	0.003197	0.05514	-2.03611	4.817194539	4.800813458	4.42354572	4.248628261	4.304955389	3.97825515
ENSMUSG00000029177.9	Cenpa	-0.49794	3.167646	-2.58871	0.020773	0.158924	-3.5713	3.546167343	3.283973678	3.34939296	3.304748347	2.925559761	2.596033605
ENSMUSG00000045328.11	Cenpe	-0.49724	4.111444	-2.29203	0.037065	0.203267	-4.3202	4.372631088	4.333034497	4.30927265	4.301848457	4.121434353	3.230442174
ENSMUSG00000032477.13	Cdc25a	-0.49689	3.649919	-2.76812	0.014535	0.132596	-3.34993	3.877044395	4.185409844	3.64552505	3.68013677	3.078137519	3.433259057
ENSMUSG00000009575.14	Cbx5	-0.4968	6.140977	-2.91804	0.010754	0.111096	-3.32235	6.435424541	6.639089259	6.09455312	6.299669707	5.58063786	5.796485952
ENSMUSG00000069729.13	Arid1b	-0.49628	5.013942	-3.91085	0.001433	0.034704	-1.30897	5.468788083	5.244886322	5.06982845	4.893568324	4.782152692	4.624425206
ENSMUSG00000032235.15	Ice2	-0.49582	2.412064	-2.29265	0.03702	0.203267	-3.9195	2.552565114	2.600447343	2.79156659	2.421562012	1.848833971	2.257409221
ENSMUSG00000034462.9	Pkd2	-0.49523	3.519065	-2.83013	0.012836	0.123295	-3.20257	3.782234701	3.607715344	3.84002808	3.689042323	3.078137519	3.117231563
ENSMUSG00000024238.13	Zeb1	-0.49498	4.076795	-3.79427	0.001814	0.039776	-1.42642	4.443192585	4.315386631	4.20548355	3.955771213	3.815103113	3.725833933
ENSMUSG00000066037.14	Hnrnpr	-0.49476	4.920532	-3.61377	0.002617	0.048717	-1.89586	5.210144418	5.09262424	5.18345724	4.997591388	4.435815639	4.603559418
ENSMUSG00000022351.14	Sqle	-0.49469	3.327358	-3.15885	0.006605	0.08563	-2.53054	3.621129401	3.664577681	3.44364329	3.20893256	2.925559761	3.10030826
ENSMUSG00000022377.16	Asap1	-0.49463	4.360662	-4.07339	0.001032	0.030012	-0.91511	4.718228089	4.647047756	4.46346473	4.148877437	4.131435745	4.054916357
ENSMUSG00000054942.13	Miga1	-0.49432	2.562904	-2.42134	0.028857	0.184162	-3.7311	2.912295242	3.026373159	2.51593215	2.442778498	2.192420947	2.287623834
ENSMUSG00000099998.1	Gm6501	-0.4941	2.82273	-2.81986	0.013103	0.125115	-3.06558	3.06330303	3.090031796	3.05310675	2.54441876	2.71556744	2.469953857
ENSMUSG00000028556.15	Dock7	-0.49249	4.902612	-3.31479	0.00481	0.071288	-2.49057	5.277350584	5.216643429	4.94647902	4.842598252	4.813550994	4.319048287
ENSMUSG00000044950.7	Pwwp2a	-0.49189	3.145262	-2.53244	0.023213	0.167585	-3.66898	3.339716465	3.593142542	3.23543677	3.233489664	2.531445035	2.938340781
ENSMUSG00000032855.5	Pkd1	-0.49017	4.566377	-2.62902	0.019179	0.153861	-3.78214	4.689488193	4.838369463	4.86791748	4.682368317	4.529555454	3.79056382
ENSMUSG00000064337.1	mt-Rnr1	-0.49013	11.33729	-2.39102	0.030611	0.188597	-4.17122	11.59320641	12.00436315	11.1452066	11.10798472	11.45054235	10.72244649
ENSMUSG00000024063.12	Lbh	-0.49006	3.616489	-2.8342	0.012731	0.122621	-3.21746	3.726739589	4.270298741	3.63557539	3.405442347	3.187635884	3.473243927
ENSMUSG00000033904.15	Ccp110	-0.48572	3.225701	-2.68711	0.017087	0.145861	-3.40371	3.232232039	3.692189912	3.45500315	3.183950203	3.067758373	2.723072794
ENSMUSG00000058454.14	Dhcr7	-0.48305	2.388128	-2.29881	0.036584	0.202683	-3.90351	2.892257488	2.510249534	2.47187697	2.3105307	2.030831804	2.113019312
ENSMUSG00000021375.9	Kif13a	-0.48188	3.120423	-2.78758	0.01398	0.129196	-3.19203	3.216201929	3.371970159	3.46627426	2.922175243	3.067758373	2.678160372
ENSMUSG00000064302.13	Clasp1	-0.48169	5.484224	-3.66285	0.002369	0.046124	-1.83281	5.693835846	5.97811799	5.50640937	5.390506077	5.254368768	5.082107042
ENSMUSG00000041684.11	Bivm	-0.4816	2.262151	-2.19553	0.044568	0.214964	-4.04961	2.500570476	2.65759125	2.40316422	1.914602024	1.824380927	2.272595625

ENSMUSG00000041215.15	Yeats2	-0.4803	3.932657	-2.20674	0.043629	0.213843	-4.43242	4.058815639	4.35046909	4.05683947	4.109618006	3.928430797	3.091771578
ENSMUSG00000026918.16	Brd3	-0.48009	4.844779	-3.44123	0.003718	0.060404	-2.23339	4.963607332	5.386832491	4.90979836	4.793895061	4.491234608	4.523303281
ENSMUSG00000039630.9	Hnrnpu	-0.47794	5.027585	-4.00819	0.001177	0.031531	-1.11522	5.257906961	5.197502916	5.33255495	4.994002593	4.631735849	4.751804512
ENSMUSG00000042460.5	C1galt1	-0.47711	2.671364	-2.50677	0.024415	0.171439	-3.60394	2.787668587	2.865908486	3.05310675	2.563914936	2.248636956	2.508947988
ENSMUSG00000031078.15	Cttn	-0.47649	4.230972	-3.88091	0.001522	0.036216	-1.27872	4.574736495	4.5063661	4.33409382	4.020703448	3.962444829	3.98748843
ENSMUSG00000015749.12	Anp32e	-0.47567	4.983443	-3.81224	0.001749	0.038925	-1.50376	5.269604552	5.254179076	5.13130596	4.994002593	4.66310002	4.588468328
ENSMUSG00000036555.14	Iqce	-0.47507	3.275669	-2.21525	0.042929	0.212526	-4.26196	3.45348534	3.719283573	3.33716666	3.559065536	2.590640828	2.994374816
ENSMUSG00000041415.9	Dicer1	-0.47377	5.245779	-4.39541	0.000543	0.02148	-0.35865	5.488942474	5.557930511	5.40169732	5.101302738	4.926995993	4.997805721
ENSMUSG00000031503.13	Col4a2	-0.47241	4.441813	-3.06467	0.007995	0.094444	-2.92859	4.618082525	5.029082446	4.40609355	4.369885978	4.022794485	4.204941801
ENSMUSG00000028884.14	Rpa2	-0.47069	3.241842	-3.09344	0.007542	0.091676	-2.63809	3.439745284	3.517985183	3.48855571	2.922175243	3.02547857	3.057110571
ENSMUSG00000022521.10	Crebbp	-0.47029	5.426727	-3.97092	0.001269	0.032923	-1.21293	5.649758231	5.811868647	5.52539248	5.29165269	5.261217016	5.020472252
ENSMUSG00000040010.10	Slc7a5	-0.47012	2.994196	-2.46243	0.026632	0.1778	-3.75883	3.383437843	3.371970159	2.92825526	2.966798235	2.767797399	2.546915839
ENSMUSG00000037369.16	Kdm6a	-0.46933	4.30908	-2.61853	0.019582	0.155099	-3.7621	4.422384123	4.681897157	4.51321776	4.142407941	4.459827761	3.634746011
ENSMUSG00000074102.4	Rbm15b	-0.46896	4.373803	-2.72058	0.015984	0.140192	-3.58109	4.689488193	4.880983807	4.26478073	4.440143438	3.839712581	4.127708698
ENSMUSG00000052539.15	Magi3	-0.4689	2.389592	-2.13843	0.049652	0.222533	-4.17313	2.743613407	2.510249534	2.55868179	2.54441876	2.009298643	1.971291202
ENSMUSG00000037395.15	Rcor3	-0.46819	2.765977	-2.49443	0.025013	0.173643	-3.64755	3.216201929	2.841455443	2.91186082	2.71106863	2.485397668	2.429876417
ENSMUSG00000080746.3	Rpsa-ps12	-0.46813	2.987795	-2.51552	0.023998	0.170562	-3.66186	3.279280612	2.95977541	3.42065164	2.657629371	2.633496307	2.975937567
ENSMUSG00000089788.1	#N/A	-0.468	2.219565	-2.18633	0.045353	0.216675	-4.05506	2.500570476	2.600447343	2.30613976	1.883905227	1.896530713	2.129795025
ENSMUSG00000038335.13	Tsr1	-0.46761	2.610103	-2.2797	0.037953	0.205148	-3.98767	2.698170436	3.069122806	2.75527046	2.463687488	2.561346521	2.113019312
ENSMUSG00000009621.18	Vav2	-0.46761	3.522841	-2.99265	0.00925	0.101526	-2.89337	3.669024091	3.96544416	3.64552505	3.437503556	3.09867445	3.32087564
ENSMUSG00000026496.11	Parp1	-0.46655	5.249191	-3.60091	0.002686	0.049724	-1.94549	5.557344892	5.623935696	5.26639358	5.231967031	4.909666115	4.905838141
ENSMUSG00000021690.9	Jmy	-0.46641	2.984878	-2.15907	0.047755	0.220003	-4.28465	3.439745284	3.110642075	3.09727433	2.602134258	3.216111053	2.443359647
ENSMUSG00000022702.15	Hira	-0.46581	2.378715	-2.21906	0.042618	0.212361	-4.03668	2.721070838	2.712557709	2.426434	2.031246943	2.354895644	2.026083675
ENSMUSG00000024533.15	Spire1	-0.46568	3.836642	-3.01347	0.008869	0.099093	-2.91986	4.220226186	4.063847864	3.89944156	3.872223421	3.565041005	3.399070371
ENSMUSG00000028278.14	Rragd	-0.46531	3.284271	-2.74733	0.015153	0.136008	-3.30527	3.507173211	3.705800343	3.36151652	3.158527607	2.805765249	3.166842012
ENSMUSG00000037426.17	Depdc5	-0.46346	3.634579	-3.2094	0.00596	0.080458	-2.4974	3.793082083	4.084832889	3.74142048	3.426895457	3.376080974	3.385164769
ENSMUSG00000072235.5	Tuba1a	-0.46333	6.362753	-2.72422	0.015868	0.139769	-3.69393	6.43888217	7.020258595	6.32406167	6.367807665	5.84199834	6.183509075
ENSMUSG00000073664.11	Nbeal1	-0.46318	3.815472	-2.49619	0.024927	0.173405	-3.89117	4.283132937	3.907728662	3.90773294	3.791829642	3.82746032	3.174947061
ENSMUSG00000000881.12	Dlg3	-0.46284	3.210424	-2.98518	0.009391	0.102622	-2.83848	3.558934639	3.388943238	3.37353905	3.024226515	2.959640757	2.957261643
ENSMUSG00000020156.16	Mum1	-0.46251	3.640934	-2.8149	0.013234	0.125684	-3.25843	3.866812472	3.871957531	3.83133712	3.791829642	3.253224226	3.230442174
ENSMUSG00000004865.15	Srpk1	-0.46214	4.708082	-3.67605	0.002306	0.045505	-1.7502	4.953973273	4.928192765	4.92212916	4.717611476	4.291638109	4.434947407
ENSMUSG00000038533.15	Cbfa2t2	-0.46136	4.458906	-3.0534	0.00818	0.095707	-2.95266	4.689488193	4.794457963	4.56655822	4.604489679	4.017411289	4.081029198
ENSMUSG00000045414.7	1190002N15R	-0.46096	2.804034	-2.31162	0.035694	0.20128	-3.97908	3.339716465	3.090031796	2.69905445	2.657629371	2.453862094	2.583910046
ENSMUSG00000032601.13	Prkar2a	-0.45963	3.409968	-2.57467	0.021358	0.161151	-3.65439	3.645275488	3.835276868	3.42065164	3.529621758	3.02547857	3.003505827
ENSMUSG00000029267.17	Mtf2	-0.4571	4.276481	-3.34737	0.004501	0.068326	-2.34613	4.542957786	4.521837508	4.4293165	4.272524377	4.075549725	3.816700314
ENSMUSG00000039967.14	Zfp292	-0.45667	5.148155	-2.41969	0.02895	0.184552	-4.22994	5.563693395	5.235533323	5.31707524	5.046919101	5.338799957	4.386910316
ENSMUSG00000075703.15	Selenoi	-0.45666	3.663085	-3.21491	0.005893	0.080014	-2.49224	3.956387826	3.907728662	3.79604007	3.578366092	3.433796471	3.306191594
ENSMUSG00000051786.14	Tubgcp6	-0.45663	3.705003	-3.25148	0.005471	0.076896	-2.42948	3.814535193	4.095212035	3.89110226	3.578366092	3.417540581	3.433259057
ENSMUSG00000078786.9	BC024978	-0.45639	2.356088	-2.28302	0.037712	0.204879	-3.92287	2.500570476	2.571003565	2.66032206	2.239564179	2.05204829	2.113019312
ENSMUSG00000024037.12	Wdr4	-0.45615	2.843549	-2.64693	0.018509	0.152003	-3.38927	3.027006901	3.069122806	3.09727433	2.728450708	2.605067899	2.534370585
ENSMUSG00000021493.15	Pdlim7	-0.45599	2.93072	-2.34035	0.033769	0.197192	-3.9597	3.30981498	3.388943238	2.80937787	2.71106863	2.818202352	2.546915839
ENSMUSG00000028080.15	Lrba	-0.45482	3.421695	-2.66953	0.017696	0.148505	-3.48185	3.63325296	3.771990623	3.53211207	3.257635752	3.425691422	2.909485918
ENSMUSG00000059474.13	Mbtd1	-0.45437	4.533792	-2.61425	0.019749	0.155682	-3.80499	4.677829988	4.979519345	4.60785464	4.781457958	4.04941323	4.106677785
ENSMUSG00000048668.15	Rhno1	-0.45348	2.552951	-2.16583	0.047149	0.219041	-4.1671	2.787668587	3.090031796	2.51593215	2.333431102	2.133925297	2.45671803
ENSMUSG00000039242.9	B3galnt2	-0.45282	2.383165	-2.28747	0.037391	0.204379	-3.92145	2.500570476	2.629302206	2.67981823	2.239564179	2.153688552	2.096046233
ENSMUSG00000033964.12	Zbtb41	-0.45271	3.311033	-2.40594	0.029735	0.186632	-3.93644	3.546167343	3.705800343	3.36151652	3.383665405	2.702209057	3.166842012
ENSMUSG00000027284.16	Cdan1	-0.45243	2.624392	-2.38938	0.030709	0.188787	-3.80067	2.912295242	2.791269685	2.82697193	2.583151155	2.230410613	2.402525929
ENSMUSG00000054008.8	Ndst1	-0.44956	4.089232	-2.40375	0.029862	0.186992	-4.11624	4.450062575	4.35046909	4.13660674	4.193369356	3.367644136	4.037241465
ENSMUSG00000020069.16	Hnrnph3	-0.44914	4.177769	-3.5859	0.00277	0.050885	-1.85691	4.52354904	4.315386631	4.36453156	3.925946997	4.054678546	3.88251887
ENSMUSG00000028282.12	Casp8ap2	-0.44905	3.896792	-2.72502	0.015843	0.139769	-3.48317	4.146014081	3.987899011	4.17145614	4.089580253	3.47365246	3.51215042
ENSMUSG00000026782.15	Abi2	-0.44805	3.662396	-2.79682	0.013723	0.127971	-3.2975	3.856507464	4.031786655	3.75066856	3.671175903	3.489291584	3.174947061
ENSMUSG00000006763.12	Saal1	-0.44733	2.799095	-2.21712	0.042776	0.212429	-4.13981	2.787668587	3.283973678	2.97634955	2.891640875	2.371868722	2.483069357
ENSMUSG00000056919.9	Cep162	-0.44657	3.154701	-2.3905	0.030642	0.188644	-3.92497	3.30981498	3.170763068	3.57439188	3.171294903	3.046773348	2.655168729
ENSMUSG00000029687.16	Ezh2	-0.44615	4.64771	-2.93203	0.010455	0.109063	-3.21418	4.924678966	4.850673973	4.81158889	4.838601865	4.209044777	4.251672852
ENSMUSG00000021811.6	Dnajc9	-0.44609	2.792714	-2.45587	0.026976	0.178849	-3.7226	2.912295242	3.228478566	2.91186082	2.657629371	2.576068027	2.469953857
ENSMUSG00000014850.15	Msh3	-0.44565	3.453851	-3.03232	0.008537	0.097743	-2.80152	3.57158994	3.63642664	3.81379654	3.183950203	3.271428821	3.245913583
ENSMUSG00000036568.6	Bicral	-0.44553	2.783072	-2.19773	0.044383	0.214943	-4.16903	3.294628576	2.685336241	3.05310675	2.333431102	2.576068027	2.755862729
ENSMUSG00000033014.12	Trim33	-0.44527	4.701543	-3.77188	0.001898	0.041006	-1.55776	4.958798344	4.951230301	4.85511241	4.655359197	4.394885375	4.393871464
ENSMUSG00000024135.9	Srbd1	-0.4452	2.656679	-2.34762	0.033298	0.196152	-3.88101	2.932058497	2.913605228	2.77353267	2.657629371	2.302744362	2.360502714
ENSMUSG00000029461.15	Fam168a	-0.44455	3.913657	-3.03114	0.008557	0.097851	-2.90089	4.171178004	4.233185568	3.98812672	3.910800454	3.698850045	3.479801643
ENSMUSG00000036893.16	Ehmt1	-0.44339	4.809383	-3.36165	0.004372	0.066975	-2.3879	5.105251212	5.163383538	4.82478437	4.747758439	4.628208475	4.386910316
ENSMUSG00000040363.14	Bcor	-0.44329	3.962642	-2.43031	0.028357	0.182324	-4.04246	4.443192585	4.146016406	3.94848974	3.840601442	4.033500991	3.364051781
ENSMUSG00000037343.7	Taf2	-0.44315	4.011889	-2.97497	0.009587	0.103779	-3.02909	4.477219989	4.223756155	3.99592509	3.918393603	3.821294947	3.634746011
ENSMUSG00000023050.8	Map3k12	-0.44289	3.355214	-2.46809	0.026339	0.177074	-3.83523	3.749194439	3.607715344	3.34939296	3.350370965	3.234786977	2.83982504
ENSMUSG00000024985.18	Tcf7l2	-0.44264	3.66163	-2.83888	0.012612	0.122107	-3.21766	4.058815639	3.871957531	3.72274456	3.578366092	3.20668164	3.531217152
ENSMUSG00000046658.16	Zfp316	-0.44172	2.654957	-2.15566	0.048064	0.220331	-4.20827	2.602750871	3.190259244	2.82697193	2.657629371	2.320338422	2.331791418
ENSMUSG00000022016.15	Akap11	-0.44076	4.343239	-2.54523	0.022635	0.165979	-3.90365	4.763053288	4.559805359	4.34023296	4.49195134	4.1512329	3.75315852
ENSMUSG00000054717.6	Hmgb2	-0.43932	3.588937	-2.31161	0.035694	0.20128	-4.17018	3.657198655	4.010009712	3.71331514	3.766811182	3.401099434	2.985185644
ENSMUSG00000061759.15	Armt1	-0.43879	2.424575	-2.15592	0.048041	0.220331	-4.15256	2.892257488	2.571003565	2.47187697	2.263609653	2.153688552	2.195012684
ENSMUSG00000035401.9	Emsy	-0.43863	4.224239	-3.38579	0.004162	0.065001	-2.26154	4.470478411	4.482843377	4.36453156	4.161730039	4.038824595	3.827023758
ENSMUSG00000043154.15	Ppp2r3a	-0.43577	3.477167	-2.66527	0.017846	0.149302	-3.50197	3.657198655	3.533333147	3.84002808	3.53950327	3.078137519	3.21480305
ENSMUSG00000022994.8	Adcy6	-0.4357	3.756751	-2.32854	0.034548	0.199437	-4.17927	3.927143006	3.976715272	3.97240236	3.741351197	3.839712581	3.083184082
ENSMUSG00000039108.14	Lsm14b	-0.43538	4.534336	-3.48432	0.003406	0.057409	-2.11172	4.689488193	4.868936179	4.69187089	4.512164561	4.199568914	4.243988706
ENSMUSG00000018378.13	Cuedc1	-0.43527	2.684117	-2.28613	0.037487	0.204486	-3.99357	2.743613407	2.913605228	3.00754417	2.620870821	2.531445035	2.287623834
ENSMUSG00000051674.14	Dcun1d4	-0.43472	3.140011	-2.20245	0.043986	0.214334	-4.24932	3.425873109	3.228478566	3.38546221	3.233489664	2.500910667	3.065854204
ENSMUSG00000028034.15	Fubp1	-0.43427	6.016457	-3.92191	0.001401	0.034494	-1.32464	6.239189589	6.198706666	6.26235807	5.984087124	5.687878927	5.726523393
ENSMUSG00000026048.16	Ercc5	-0.43268	2.591898	-2.18865	0.045155	0.216249	-4.13773	2.830418235	3.026373159	2.57959078	2.484297768	2.388644435	2.242061257
ENSMUSG00000064127.13	Med14	-0.43186	3.781847	-2.68052	0.017313	0.14661	-3.54321	3.887204261	4.29752021	3.86579117	3.416208778	3.433796471	3.79056382
ENSMUSG00000031530.6	Dusp4	-0.43172	3.723367	-3.06162	0.008045	0.094798	-2.80303	3.825143293	4.155966066	3.85725449	3.509653342	3.47365246	3.518534037
ENSMUSG00000022019.15	Tdrd3	-0.43116	3.073763	-2.50289	0.024601	0.171946	-3.70452	3.232232039	3.405718951	3.20884256	3.038233373	2.902383439	2.655168729
ENSMUSG00000025261.17	Huwe1	-0.43068	6.940094	-2.90798	0.010975	0.112023	-3.33	7.174811345	7.254757891	7.04137936	6.983634798	6.85827449	6.327707674
ENSMUSG00000022390.10	Zc3h7b	-0.42973	4.516998	-3.41403	0.00393	0.062557	-2.24928	4.581009092	4.951230301	4.67253801	4.319161005	4.361283972	4.216767237
ENSMUSG00000025959.13	Klf7	-0.42957	2.721813	-2.32736	0.034627	0.199596	-3.93127	2.932058497	2.913605228	2.92825526	2.728450708	2.453862094	2.374646897
ENSMUSG00000037029.7	Zfp146	-0.42922	3.370656	-2.71796	0.016068	0.140485	-3.37914	3.680753383	3.438696273	3.59507603	3.372652412	3.088442528	3.048313623
ENSMUSG00000009418.15	Nav1	-0.42922	4.405201	-3.32367	0.004724	0.07043	-2.41297	4.735201167	4.735962314	4.41193443	4.135909304	4.126443716	4.285753847
ENSMUSG00000025477.13	Inpp5a	-0.42791	3.147446	-2.36621	0.032121	0.193081	-3.96607	3.533286052	3.090031796	3.39728765	3.221263362	2.854883015	2.787923938
ENSMUSG00000029920.9	Smarcad1	-0.42771	4.927629	-3.30397	0.004917	0.072585	-2.51383	5.21821546	5.216643429	4.98617052	4.953925154	4.537098975	4.653719513
ENSMUSG00000055322.14	Tns1	-0.42763	3.296773	-2.62239	0.019433	0.154577	-3.54012	3.584135193	3.650570823	3.34939296	2.922175243	3.036165249	3.238198617
ENSMUSG00000030259.12	Rassf8	-0.42734	3.075102	-2.54207	0.022777	0.166062	-3.63427	3.354437971	3.4549065	3.08270153	2.860446254	2.878828711	2.819288109
ENSMUSG00000023927.15	Satb1	-0.42588	3.414879	-2.32104	0.035051	0.200218	-4.11201	3.946705272	3.719283573	3.23543677	3.32773999	3.168335328	3.091771578
ENSMUSG00000032898.9	Fbxo21	-0.42465	3.708539	-2.99641	0.00918	0.100966	-2.9255	3.887204261	3.919457955	3.93232493	3.697893241	3.367644136	3.446710856
ENSMUSG00000029729.12	Zkscan1	-0.4235	4.360931	-3.49182	0.003354	0.056904	-2.0724	4.562108887	4.618549149	4.53479117	4.266587352	4.006584258	4.176966229
ENSMUSG00000063108.14	Zfp26	-0.4229	3.713746	-2.31296	0.035602	0.20128	-4.19714	3.927143006	4.105517043	3.72274456	3.933461051	3.187635884	3.405973231
ENSMUSG00000032621.7	Srek1	-0.42263	4.512544	-2.89445	0.011278	0.113704	-3.26844	4.757525714	4.559805359	4.81600081	4.599774985	4.309367304	4.032788699
ENSMUSG00000037461.10	Ints7	-0.42224	3.793184	-2.54159	0.022798	0.166062	-3.80267	4.040725182	4.155966066	3.79604007	3.887778236	3.57240171	3.306191594
ENSMUSG00000033054.7	Npat	-0.42074	3.273893	-2.28649	0.037461	0.204486	-4.13779	3.425873109	3.548519551	3.48855571	2.860446254	3.449871234	2.87009248
ENSMUSG00000032228.16	Tcf12	-0.4188	5.401154	-2.94615	0.010162	0.107283	-3.25067	5.664600904	5.824400278	5.34482023	5.406792553	5.210224896	4.956086331
ENSMUSG00000035967.15	Ints6l	-0.41877	3.045406	-2.2171	0.042778	0.212429	-4.20048	3.507173211	3.247215128	2.99203117	3.092939265	2.754916107	2.678160372
ENSMUSG00000037664.12	Cdkn1c	-0.41708	5.420368	-3.14065	0.006853	0.087016	-2.87047	5.58881168	5.92383197	5.38100257	5.314852306	5.127693344	5.186017674
ENSMUSG00000040681.14	Hmgn1	-0.41668	4.865896	-2.78155	0.01415	0.130303	-3.52861	5.222234112	5.272586918	4.73444531	4.878076148	4.533332145	4.554701584
ENSMUSG00000045039.9	Megf8	-0.41625	5.081892	-3.3747	0.004258	0.065754	-2.38591	5.265715884	5.378358487	5.22053789	5.117885622	4.75976008	4.749092679
ENSMUSG00000061273.3	Mmgt1	-0.41509	2.917574	-2.35823	0.032621	0.194379	-3.9242	3.279280612	2.936875008	3.12598563	2.812351965	2.754916107	2.596033605
ENSMUSG00000032349.13	Elovl5	-0.41491	3.560371	-2.54723	0.022546	0.165758	-3.73974	3.558934639	4.115748966	3.64552505	3.458488581	3.307163033	3.276367379
ENSMUSG00000043384.15	Gprasp1	-0.41456	3.975068	-2.46541	0.026477	0.177559	-3.9811	4.228241179	4.426417943	3.89944156	3.895493201	3.934155785	3.466656267
ENSMUSG00000029478.16	Ncor2	-0.41403	5.069556	-3.06152	0.008046	0.094798	-3.00442	5.156552441	5.616749155	5.06982845	4.90125247	4.816653565	4.85629868
ENSMUSG00000024843.15	Chka	-0.41271	3.754392	-2.85639	0.012176	0.119504	-3.20349	4.040725182	3.732641955	4.07167451	3.625517186	3.615794305	3.440000635
ENSMUSG00000026042.16	Col5a2	-0.4122	4.213345	-3.21093	0.005941	0.080289	-2.60487	4.321100787	4.639975707	4.31551809	3.999382444	4.01200793	3.992083004
ENSMUSG00000054199.16	Gon4l	-0.41128	3.834964	-2.94842	0.010115	0.10713	-3.04413	3.975560135	4.020939272	4.09364491	3.81642163	3.62997324	3.473243927
ENSMUSG00000035941.15	Ibtk	-0.41097	3.747272	-2.56445	0.021793	0.162831	-3.75016	3.877044395	4.074378531	3.88271448	3.732763701	3.67853006	3.238198617
ENSMUSG00000034021.15	Pds5b	-0.41077	4.414144	-2.84181	0.012538	0.121742	-3.35508	4.642279235	4.58211552	4.60275677	4.541963109	4.156140031	3.959609397
ENSMUSG00000019777.16	Hdac2	-0.40977	4.868053	-3.3653	0.00434	0.066555	-2.38625	4.944274446	5.254179076	5.0247992	4.764706593	4.518165697	4.702194064
ENSMUSG00000035958.2	Tdp2	-0.40932	2.861751	-2.23918	0.041014	0.210441	-4.11678	3.008510557	2.95977541	3.1817489	2.795957527	2.767797399	2.45671803
ENSMUSG00000004798.14	Ulk2	-0.40901	3.818909	-2.6673	0.017774	0.148886	-3.57511	4.049798765	4.105517043	3.91597693	3.808270789	3.324703614	3.709187267
ENSMUSG00000039740.6	Alg2	-0.40836	3.268394	-2.1752	0.04632	0.217853	-4.32826	3.199991702	3.883980059	3.36151652	3.196495457	2.842759456	3.125619349
ENSMUSG00000034832.15	Tet3	-0.40628	5.630576	-3.31376	0.00482	0.071309	-2.53545	6.011996733	5.900349132	5.59122027	5.485576186	5.470701157	5.323612337
ENSMUSG00000037656.8	Slc20a2	-0.40513	2.816504	-2.35703	0.032697	0.194499	-3.90222	3.008510557	3.04790632	3.00754417	2.563914936	2.675115396	2.596033605

ENSMUSG00000024350.5	Dnajc18	-0.40195	3.215639	-2.21677	0.042805	0.212429	-4.2432	3.520288711	3.04790632	3.64552505	2.90698884	3.197190191	2.975937567
ENSMUSG00000041570.14	Camsap2	-0.40069	4.46138	-3.11189	0.007265	0.090174	-2.83785	4.677829988	4.735962314	4.56131205	4.471450888	4.227811807	4.093910489
ENSMUSG00000020898.18	Ctc1	-0.40043	3.527891	-2.21668	0.042812	0.212429	-4.32062	3.749194439	3.835276868	3.56393752	3.634765262	3.417540581	2.966629825
ENSMUSG00000021693.19	Kif2a	-0.40031	3.914159	-2.39447	0.030407	0.188388	-4.09605	4.252022405	4.195092397	3.87427764	4.069260269	3.601474634	3.492828323
ENSMUSG00000058761.4	Rnf169	-0.39968	3.56391	-2.7882	0.013962	0.129196	-3.29103	3.738010701	3.664577681	3.86579117	3.458488581	3.271428821	3.385164769
ENSMUSG00000025758.10	Plk4	-0.39941	3.285699	-2.36972	0.031903	0.192665	-3.99334	3.439745284	3.4549065	3.51049826	3.394594965	3.014712139	2.899737941
ENSMUSG00000046574.7	Prr12	-0.39838	5.061593	-3.14012	0.006861	0.087016	-2.84904	5.28120806	5.339597185	5.155878	5.094615843	4.850348611	4.647908044
ENSMUSG00000075595.9	Zfp652	-0.39665	4.608609	-3.24651	0.005527	0.077252	-2.59285	4.768559764	4.990681451	4.67253801	4.476603376	4.304955389	4.438318191
ENSMUSG00000022498.16	Txndc11	-0.39319	2.820881	-2.15203	0.048394	0.22073	-4.25433	2.871937504	3.04790632	3.09727433	2.76259893	2.702209057	2.443359647
ENSMUSG00000055897.13	Ppp4r1l-ps	-0.39226	3.48972	-2.18592	0.045389	0.216675	-4.36359	3.877044395	3.319818877	3.7870792	3.529621758	3.367644136	3.057110571
ENSMUSG00000032525.14	Nktr	-0.39223	5.805978	-3.32127	0.004747	0.070695	-2.52474	5.945490371	6.086134373	5.97536377	5.661027947	5.776587038	5.391264968
ENSMUSG00000032423.12	Syncrip	-0.39191	5.323405	-3.73501	0.002046	0.042346	-1.68029	5.508819184	5.523759821	5.52269585	5.222797328	5.11514809	5.047210223
ENSMUSG00000057133.14	Chd6	-0.39172	4.954823	-2.63341	0.019013	0.153491	-3.81779	5.214185583	5.29976565	4.93435546	5.018938684	4.816653565	4.445036225
ENSMUSG00000029202.12	Pds5a	-0.39139	5.4894	-3.02913	0.008592	0.097998	-3.09243	5.842216806	5.580269461	5.62681279	5.518423552	5.310468178	5.058206427
ENSMUSG00000027804.13	Ppid	-0.39054	3.46073	-2.43792	0.027939	0.181144	-3.91424	3.877044395	3.533333147	3.55340685	3.372652412	3.078137519	3.349802826
ENSMUSG00000022526.7	Zfp251	-0.3898	2.730127	-2.17412	0.046415	0.217853	-4.19507	2.951554672	2.95977541	2.86152915	2.639367164	2.421621757	2.546915839
ENSMUSG00000024188.15	Luc7l	-0.38878	4.675857	-2.83731	0.012652	0.122318	-3.39985	4.80116443	4.933986728	4.8593934	4.726289367	4.533332145	4.200978351
ENSMUSG00000001016.12	Ilf2	-0.38846	4.553405	-3.00094	0.009097	0.100476	-3.06739	4.843521822	4.715924561	4.66766403	4.575967596	4.348478902	4.168872547
ENSMUSG00000026355.11	Mcm6	-0.38745	4.572302	-3.26397	0.005334	0.075795	-2.55352	4.718228089	4.838369463	4.73909917	4.507137744	4.23710456	4.393871464
ENSMUSG00000056342.16	Usp34	-0.38736	5.477867	-3.19812	0.006098	0.081642	-2.75922	5.807865301	5.638202265	5.56524846	5.475316978	5.277070968	5.103496538
ENSMUSG00000025326.11	Ube3a	-0.38705	5.083453	-3.32639	0.004698	0.070353	-2.48186	5.395944256	5.207104915	5.22053789	5.053830228	4.847317738	4.775984325
ENSMUSG00000015488.14	Cacfd1	-0.38409	4.258003	-2.86086	0.012067	0.119064	-3.2927	4.408343185	4.418173949	4.49682332	4.324885993	3.916912213	3.982879177
ENSMUSG00000028496.17	Mllt3	-0.38405	3.966773	-2.73226	0.015616	0.13868	-3.48331	3.946705272	4.324237549	4.19874197	3.918393603	3.664822641	3.747734848
ENSMUSG00000034610.14	Zcchc11	-0.38291	5.081262	-2.17618	0.046234	0.217853	-4.65706	5.455193683	5.272586918	5.0881836	4.912702491	5.303850299	4.45505498
ENSMUSG00000030978.9	Rrm1	-0.38241	4.420311	-2.54345	0.022715	0.166062	-3.91876	4.677829988	4.762253612	4.38842767	4.541963109	4.070360169	4.081029198
ENSMUSG00000022142.7	Nup155	-0.38151	4.105484	-2.22144	0.042425	0.212176	-4.44119	4.456900005	4.223756155	4.16455328	4.324885993	3.875857144	3.5869505
ENSMUSG00000042323.16	Pbrm1	-0.38046	5.358837	-3.32581	0.004703	0.070356	-2.50025	5.563693395	5.620346901	5.46205791	5.343336845	5.034833735	5.128752238
ENSMUSG00000028580.15	Pum1	-0.3803	5.312914	-3.27817	0.005182	0.07461	-2.59251	5.554160134	5.527596778	5.42499058	5.30619611	4.961043474	5.103496538
ENSMUSG00000028901.13	Gmeb1	-0.3793	3.293756	-2.22973	0.04176	0.211919	-4.2406	3.771305141	3.422301835	3.2744309	3.132648975	2.970824495	3.191021824
ENSMUSG00000034781.5	Gna11	-0.37905	3.408177	-2.4315	0.028291	0.182324	-3.91265	3.608903098	3.56354776	3.58477102	3.426895457	3.225479235	3.039462705
ENSMUSG00000025134.2	Alyref	-0.37824	4.467519	-3.11196	0.007264	0.090174	-2.83852	4.599664891	4.844534836	4.54013449	4.29603113	4.227811807	4.296937586
ENSMUSG00000028433.14	Ubap2	-0.37789	4.657325	-3.2762	0.005203	0.07483	-2.53984	4.768559764	4.968270204	4.80272438	4.561492274	4.431774474	4.411128787
ENSMUSG00000022194.15	Pabpn1	-0.37691	3.945743	-2.65869	0.018082	0.150179	-3.61722	4.067776506	4.175661867	4.15064768	3.749887879	3.968036678	3.562445537
ENSMUSG00000015882.17	Lcorl	-0.37635	3.60789	-2.60177	0.020243	0.157832	-3.64948	3.782234701	3.883980059	3.72274456	3.54931756	3.262355237	3.446710856
ENSMUSG00000033237.18	Arid2	-0.37633	5.250424	-2.56473	0.021781	0.162831	-3.9693	5.560522636	5.452890155	5.29512076	5.340513566	5.140130447	4.713366976
ENSMUSG00000055296.14	Tmem245	-0.37619	3.771873	-2.42687	0.028547	0.182941	-4.00636	3.985051584	4.165847577	3.73211274	3.800073636	3.481493213	3.466656267
ENSMUSG00000041859.10	Mcm3	-0.37489	4.226146	-2.62696	0.019257	0.154094	-3.73085	4.401271136	4.674994296	4.17832613	4.161730039	4.033500991	3.907055092
ENSMUSG00000034636.9	Zyg11b	-0.37452	4.415115	-3.03052	0.008568	0.097851	-2.98971	4.555753392	4.611335659	4.62304104	4.418887878	4.170762066	4.110908565
ENSMUSG00000041773.7	Enc1	-0.37413	4.810359	-2.8074	0.013435	0.126604	-3.47285	5.185656786	5.123375565	4.70144104	4.575967596	4.610441071	4.665272688
ENSMUSG00000085438.1	1700020I14Ril	-0.37366	5.11454	-2.63012	0.019137	0.153774	-3.83739	5.388811015	5.158442796	5.34482023	5.191803761	4.603272385	5.000088467
ENSMUSG00000028431.11	Ikbkap	-0.37356	4.521036	-2.40637	0.029711	0.186632	-4.18477	4.795781233	4.715924561	4.59250667	4.571158608	4.491234608	3.959609397
ENSMUSG00000051329.13	Nup160	-0.37296	3.89661	-2.26429	0.039091	0.207745	-4.32294	4.283132937	4.020939272	3.92417408	4.027740999	3.425691422	3.697981813
ENSMUSG00000041852.14	Tcf20	-0.37294	5.553901	-3.03655	0.008464	0.097312	-3.08035	5.699612411	5.932540417	5.59122027	5.505878298	5.402132513	5.192020575
ENSMUSG00000046434.15	Hnrnpa1	-0.37245	5.367144	-2.96041	0.009873	0.105736	-3.22081	5.582572989	5.638202265	5.43649768	5.436181802	5.10249279	5.006915112
ENSMUSG00000037234.16	Hook3	-0.37194	4.630024	-3.22434	0.005782	0.079328	-2.63889	4.843521822	4.762253612	4.83351477	4.55663471	4.322522669	4.461695706
ENSMUSG00000036676.14	Tmtc3	-0.37169	3.261607	-2.33538	0.034095	0.198317	-4.04829	3.493937384	3.47093661	3.38546221	3.145646315	2.890654146	3.183006831
ENSMUSG00000004099.15	Dnmt1	-0.37163	5.45368	-2.8803	0.011604	0.115859	-3.38052	5.502223995	5.876477728	5.54146724	5.436181802	5.279321662	5.086410387
ENSMUSG00000022191.15	Drosha	-0.37111	4.544268	-2.67087	0.017649	0.148202	-3.69912	4.695282157	4.922375438	4.57178538	4.517173924	4.443864168	4.115126975
ENSMUSG00000039219.17	Arid4b	-0.37108	4.757967	-3.25018	0.005486	0.076935	-2.60281	5.061061079	4.819713663	4.94244914	4.632458795	4.506685305	4.585431074
ENSMUSG00000039585.15	Myo9a	-0.36975	4.485133	-2.151	0.048489	0.220866	-4.63051	4.654227089	4.819713663	4.5454581	4.193369356	4.733735562	3.964293476
ENSMUSG00000020721.16	Helz	-0.36956	4.323383	-2.64085	0.018734	0.152448	-3.72104	4.542957786	4.442765937	4.50777362	4.408141497	4.141368281	3.897290621
ENSMUSG00000026623.16	Lpgat1	-0.36885	4.050216	-2.69561	0.0168	0.144352	-3.56897	4.290806865	4.35046909	4.07167451	3.992204842	3.698850045	3.897290621
ENSMUSG00000037236.14	Matr3	-0.36873	5.761747	-3.49246	0.00335	0.056901	-2.182	5.967203583	5.842995961	6.02575179	5.645098976	5.58610262	5.50332657
ENSMUSG00000040390.13	Map3k10	-0.36778	3.421805	-2.43474	0.028113	0.181717	-3.91039	3.467095772	3.664577681	3.69426939	3.196495457	3.158587352	3.349802826
ENSMUSG00000036197.15	Gxylt1	-0.36738	3.936434	-2.44283	0.027672	0.18028	-4.01291	4.179469378	4.053239764	4.10810814	3.984991352	3.465768861	3.827023758
ENSMUSG00000020576.9	Nbas	-0.36735	3.976098	-2.35919	0.03256	0.194277	-4.17157	4.187713372	4.185409844	4.07167451	4.135909304	3.732093337	3.543789738
ENSMUSG00000024236.17	Svil	-0.36647	3.808375	-2.70358	0.016536	0.142878	-3.50403	3.946705272	4.084832889	3.94042998	3.68013677	3.698850045	3.499297818
ENSMUSG00000036054.15	Sugp2	-0.36516	4.042326	-2.84774	0.01239	0.120737	-3.27827	4.29844019	4.155966066	4.20548355	3.992204842	3.764587842	3.837273854
ENSMUSG00000038241.16	Cep250	-0.36332	4.802824	-2.5722	0.021462	0.161255	-3.91641	5.126848846	4.945505313	4.8721607	4.743490107	4.813550994	4.315386623
ENSMUSG00000058013.11	Sep-11	-0.36305	5.671261	-3.30187	0.004938	0.072641	-2.56	5.911051003	5.964029059	5.68585715	5.53580563	5.476597739	5.454222736
ENSMUSG00000000631.19	Myo18a	-0.36296	4.879992	-3.41123	0.003952	0.062723	-2.29544	5.033882347	5.108081835	5.04373248	4.70888107	4.717228574	4.668146583
ENSMUSG00000025151.16	Maged1	-0.36257	5.769196	-3.25887	0.005389	0.076047	-2.64789	5.831733962	6.132222606	5.89005645	5.586729565	5.636132972	5.538302708
ENSMUSG00000018068.13	Ints2	-0.36213	3.918951	-2.62246	0.01943	0.154577	-3.67895	4.07668206	4.146016406	4.05683947	3.933461051	3.732093337	3.56861091
ENSMUSG00000054280.14	Prr14l	-0.36188	4.256163	-2.44072	0.027786	0.180652	-4.07913	4.195910525	4.611335659	4.48578927	4.290190252	4.10630105	3.847451639
ENSMUSG00000057691.8	Zfp746	-0.36169	3.690544	-2.49858	0.024811	0.172863	-3.85767	4.03159417	3.79763921	3.7870792	3.56874809	3.35915767	3.599048643
ENSMUSG00000027312.14	Atrn	-0.36044	3.691614	-2.3532	0.03294	0.19504	-4.11961	3.645275488	4.063847864	3.89110226	3.689042323	3.489291584	3.37112383
ENSMUSG00000028676.17	Srsf10	-0.36039	4.983497	-3.22021	0.00583	0.07959	-2.68406	5.277350584	5.061203143	5.14539833	4.90125247	4.769399503	4.746375738
ENSMUSG00000050989.9	Selenon	-0.36017	4.383231	-2.97364	0.009613	0.103942	-3.09548	4.599664891	4.681897157	4.41775175	4.242591871	4.209044777	4.148437431
ENSMUSG00000032913.13	Lrig2	-0.36012	4.128652	-2.80537	0.01349	0.126608	-3.37566	4.328575908	4.409882576	4.19196874	4.062422838	3.833599457	3.945465215
ENSMUSG00000017376.15	Nlk	-0.35997	3.559617	-2.22375	0.042239	0.212176	-4.31553	3.846128318	3.745877783	3.60530795	3.671175903	3.168335328	3.32087564
ENSMUSG00000030201.15	Lrp6	-0.35993	5.0914	-2.85996	0.012089	0.119071	-3.39763	5.427614443	5.317604456	5.06982845	5.011857957	4.983303434	4.738194081
ENSMUSG00000029253.12	Cenpc1	-0.35851	3.362017	-2.16074	0.047605	0.219824	-4.37473	3.507173211	3.422301835	3.65540657	3.416208778	2.948369644	3.222643803
ENSMUSG00000038456.9	Dennd2a	-0.35698	3.945425	-2.39847	0.030171	0.18787	-4.09524	4.204061366	4.35046909	3.84002808	3.864382668	3.698850045	3.714757525
ENSMUSG00000063077.14	Kif1b	-0.35695	5.87888	-2.97482	0.00959	0.103779	-3.20933	6.002681478	6.248378123	5.92315198	5.82956931	5.729626488	5.539872562
ENSMUSG00000057406.16	Nsd2	-0.35587	5.998597	-2.74201	0.015314	0.136729	-3.66018	6.149183679	6.220204176	6.16022216	6.13183274	5.607756986	5.722381682
ENSMUSG00000028693.15	Nasp	-0.35574	5.804327	-2.62884	0.019186	0.153861	-3.87179	6.028155086	5.821277561	6.09273612	5.902210117	5.362345948	5.619236983
ENSMUSG00000073678.4	Pgap1	-0.35513	3.454015	-2.45938	0.026791	0.178198	-3.87333	3.621129401	3.664577681	3.60530795	3.361554703	3.187635884	3.283881433
ENSMUSG00000034377.10	Tulp4	-0.35373	5.276299	-2.96681	0.009746	0.104827	-3.20313	5.322973202	5.645282991	5.39286446	5.216651653	5.107568239	4.972454197
ENSMUSG00000079225.2	Gm9531	-0.35342	5.057525	-3.09558	0.00751	0.091445	-2.9359	5.345254649	5.133482052	5.21720603	4.950225933	4.952606637	4.746375738
ENSMUSG00000031508.13	Ankrd10	-0.35314	3.592212	-2.20411	0.043848	0.214195	-4.35667	3.83567396	3.593142542	3.82259348	3.653085446	3.465768861	3.183006831
ENSMUSG00000021733.9	Slc4a7	-0.35209	4.492839	-2.53961	0.022887	0.166552	-3.93613	4.624169855	4.567280479	4.78483026	4.609189016	4.131435745	4.24013123
ENSMUSG00000042699.11	Dhx9	-0.35194	6.375898	-2.92993	0.0105	0.109327	-3.29758	6.51783862	6.61223932	6.52741164	6.44606557	6.028826669	6.123003401
ENSMUSG00000028649.17	Macf1	-0.35152	6.832741	-2.83393	0.012738	0.122621	-3.47555	7.054877689	7.159061315	6.81214112	6.76101616	6.745586119	6.463764679
ENSMUSG00000047412.15	Zbtb44	-0.35089	3.284386	-2.27322	0.038427	0.206263	-4.16238	3.467095772	3.548519551	3.37353905	3.079456036	3.216111053	3.021596284
ENSMUSG00000030451.15	Herc2	-0.35021	5.383114	-3.08351	0.007696	0.092428	-2.9806	5.58881168	5.587639567	5.49544867	5.379545378	5.191226168	5.056013881
ENSMUSG00000030872.14	Gga2	-0.34909	4.128111	-2.25838	0.039535	0.208294	-4.38101	4.365381349	4.46694575	4.07903521	4.055552849	4.126443716	3.675306375
ENSMUSG00000038766.16	Gabpb2	-0.34835	3.928596	-2.69909	0.016684	0.143634	-3.5373	4.111764519	3.99899672	4.17832613	3.800073636	3.790066562	3.692346278
ENSMUSG00000028911.16	Srsf4	-0.34827	3.26696	-2.13846	0.049649	0.222533	-4.38844	3.520288711	3.265711472	3.4995687	3.171294903	3.216111053	2.928786474
ENSMUSG00000054843.8	Atrnl1	-0.34819	4.520746	-3.0155	0.008833	0.099056	-3.03406	4.587254535	4.735962314	4.76214591	4.301848457	4.407286841	4.329977847
ENSMUSG00000035666.14	Gtf3c4	-0.34795	4.571637	-2.2377	0.04113	0.210668	-4.49238	4.859090625	4.788074346	4.58218323	4.717611476	4.054678546	4.428182119
ENSMUSG00000003233.11	Dvl3	-0.34397	3.901696	-2.27684	0.038161	0.20566	-4.30208	3.946705272	4.409882576	3.89110226	3.749887879	3.783738942	3.628857452
ENSMUSG00000041235.11	Chd7	-0.34293	5.732675	-2.1727	0.04654	0.217853	-4.69679	5.947919151	6.049241531	5.7168584	5.750951421	5.794007278	5.137073502
ENSMUSG00000022607.14	Ptk2	-0.34287	4.327759	-2.61267	0.019811	0.155828	-3.7744	4.456900005	4.654085307	4.38842767	4.242591871	4.250932414	3.973616255
ENSMUSG00000063317.7	Usp31	-0.34257	3.945026	-2.3848	0.030983	0.189631	-4.11901	4.120403437	4.2047104	4.0113965	3.925946997	3.863909291	3.543789738
ENSMUSG00000027677.17	Ttc14	-0.34136	4.162633	-2.44781	0.027404	0.180155	-4.04826	4.328575908	4.165847577	4.46346473	4.218190528	3.962444829	3.837273854
ENSMUSG00000029207.16	Apbb2	-0.34102	3.787989	-2.47015	0.026233	0.176849	-3.93086	3.917261495	3.976715272	3.98028596	3.689042323	3.449871234	3.714757525
ENSMUSG00000015176.9	Nolc1	-0.34102	3.822939	-2.27844	0.038045	0.205277	-4.28191	3.956387826	4.270298741	3.7870792	3.689042323	3.520070246	3.714757525
ENSMUSG00000048537.15	Phldb1	-0.33977	4.405628	-2.59901	0.020354	0.158008	-3.81269	4.574736495	4.788074346	4.37652909	4.336268261	4.185237388	4.172925064
ENSMUSG00000055531.12	Cpsf6	-0.3391	5.885813	-2.99614	0.009185	0.100966	-3.1677	6.050928961	6.008637677	6.1054073	5.888744866	5.719716695	5.541440708
ENSMUSG00000026933.17	Camsap1	-0.33875	4.98037	-2.65422	0.018243	0.150647	-3.78048	5.139653916	5.286240285	5.02098255	5.039974707	4.753297829	4.642073072
ENSMUSG00000030753.15	Thap12	-0.33781	3.643066	-2.25128	0.040076	0.208688	-4.28756	3.645275488	4.010009712	3.7870792	3.587920399	3.315959981	3.51215042
ENSMUSG00000004568.12	Arhgef18	-0.33426	3.477133	-2.14907	0.048666	0.220972	-4.42344	3.703929706	3.784871914	3.46627426	3.383665405	3.13889155	3.385164769
ENSMUSG00000031983.6	2310022B05Ri	-0.33414	3.842926	-2.31502	0.035461	0.20128	-4.22179	4.00384918	4.214264707	3.848667	3.529621758	3.808884591	3.652268839
ENSMUSG00000022812.13	Gsk3b	-0.33294	5.824081	-2.73713	0.015465	0.137704	-3.66699	5.974369301	6.127173799	5.87110106	5.880993631	5.56042142	5.530427716
ENSMUSG00000032185.14	Carm1	-0.3328	4.083089	-2.61187	0.019842	0.155892	-3.73146	4.187713372	4.384718652	4.17832613	3.999382444	3.911118249	3.837273854
ENSMUSG00000043940.14	Wdfy3	-0.33131	6.010557	-3.15514	0.006655	0.085817	-2.85663	6.130060785	6.246051195	6.15326521	5.941867844	5.845040398	5.747055453
ENSMUSG00000032624.15	Eml4	-0.33088	4.816837	-2.71193	0.016263	0.141339	-3.65493	5.056566698	5.007264336	4.88060989	4.822504682	4.471685595	4.662393056
ENSMUSG00000023169.14	Slc38a1	-0.33022	5.59418	-2.85923	0.012107	0.119106	-3.4265	5.742214769	5.900349132	5.63682286	5.584344945	5.317055838	5.384291202
ENSMUSG00000028484.16	Psip1	-0.33007	4.955256	-2.90357	0.011073	0.11248	-3.30147	5.020098531	5.158442796	5.1766117	4.924062353	4.687031529	4.765287742
ENSMUSG00000038759.15	Nup205	-0.32982	5.093588	-2.71222	0.016253	0.141339	-3.68019	5.352605977	5.187836581	5.22717862	5.053830228	5.029495357	4.71058185
ENSMUSG00000040423.10	Rc3h1	-0.32699	4.599328	-2.35667	0.03272	0.194499	-4.28498	4.510463281	4.968270204	4.80716344	4.613873095	4.264628989	4.431568728
ENSMUSG00000042354.6	Gnl3	-0.32495	3.566681	-2.25425	0.039849	0.208688	-4.26397	3.866812472	3.650570823	3.67496883	3.405442347	3.315959981	3.486329686
ENSMUSG00000067336.6	Bmpr2	-0.32109	5.454209	-2.79185	0.01386	0.128445	-3.55046	5.640778772	5.714242117	5.49269544	5.340513566	5.130189356	5.406833771
ENSMUSG00000051451.5	Crebzf	-0.32046	4.471297	-2.68408	0.017191	0.146277	-3.66292	4.648265531	4.762253612	4.49682332	4.29603113	4.361283972	4.263122873
ENSMUSG00000041702.6	Btbd7	-0.32045	3.974652	-2.45835	0.026846	0.178426	-3.99186	4.154450918	4.125908832	4.11528574	3.848572153	3.922683001	3.681008734
ENSMUSG00000016921.14	Srsf6	-0.32	6.083073	-3.01468	0.008847	0.099056	-3.13312	6.215194108	6.222573134	6.29116492	6.025105939	5.792432283	5.951965183

ENSMUSG00000039512.11	Uhrf1bp1	-0.31976	4.140317	-2.4275	0.028513	0.182805	-4.08085	4.313586734	4.34177813	4.23213918	4.135909304	4.017411289	3.801075306
ENSMUSG00000002718.14	Cse1l	-0.31944	4.709472	-2.27324	0.038426	0.206263	-4.44778	4.817194539	4.962612569	4.81600081	4.878076148	4.331226738	4.45172312
ENSMUSG00000002221.11	Paxip1	-0.31883	3.827294	-2.3874	0.030827	0.189038	-4.08843	3.907311834	4.063847864	3.98028596	3.758374344	3.685335238	3.56861091
ENSMUSG00000024542.8	Cep192	-0.31795	4.545036	-2.14259	0.049264	0.222208	-4.65259	4.859090625	4.537144761	4.6870619	4.618542015	4.48734594	4.081029198
ENSMUSG00000046841.4	Ckap4	-0.31664	3.832476	-2.43399	0.028154	0.181809	-4.00579	4.03159417	4.010009712	3.93232493	3.724124782	3.615794305	3.681008734
ENSMUSG00000005846.11	Rsl1d1	-0.31637	4.284487	-2.51108	0.024209	0.170977	-3.95493	4.483930211	4.529511436	4.32173662	4.218190528	3.984683344	4.168872547
ENSMUSG00000036698.10	Ago2	-0.3159	5.523812	-2.48324	0.025569	0.175411	-4.13404	5.690938867	5.900349132	5.45924014	5.480455701	5.210224896	5.401662815
ENSMUSG00000035342.13	Lzts2	-0.31574	3.74218	-2.13669	0.049815	0.222753	-4.50716	3.856507464	3.942634277	3.87427764	3.848572153	3.457841945	3.473243927
ENSMUSG00000001151.9	Pcnt	-0.31544	4.201697	-2.30123	0.036415	0.202683	-4.31859	4.328575908	4.442765937	4.29037245	4.28432563	4.006584258	3.857558126
ENSMUSG00000066324.2	Impad1	-0.31536	4.821246	-2.50437	0.02453	0.171946	-4.04348	5.105251212	4.89293166	4.93029153	4.842598252	4.459827761	4.696574992
ENSMUSG00000040945.13	Rcc2	-0.31445	5.857994	-2.79355	0.013813	0.128301	-3.5596	5.967203583	6.20351165	5.87744724	5.761543943	5.662237937	5.676020404
ENSMUSG00000020994.4	Pnn	-0.3135	4.898588	-2.82605	0.012941	0.124043	-3.44519	5.042998933	4.996230285	5.11707457	4.858474011	4.697167601	4.679585256
ENSMUSG00000079003.1	Samd1	-0.31251	3.653586	-2.18328	0.045616	0.217113	-4.40692	3.985051584	3.732641955	3.70382369	3.56874809	3.504762993	3.426485828
ENSMUSG00000030016.14	Zfp638	-0.31203	5.393249	-2.77968	0.014203	0.130538	-3.57069	5.601208706	5.472927908	5.56786679	5.406792553	5.184036643	5.126664401
ENSMUSG00000026014.15	Raph1	-0.30971	4.377661	-2.54702	0.022556	0.165758	-3.90393	4.497257742	4.529511436	4.56131205	4.353175037	4.08072068	4.243988706
ENSMUSG00000043535.13	Setx	-0.30846	4.761242	-2.45013	0.027281	0.17995	-4.13599	5.14389714	4.715924561	4.87639148	4.686821083	4.540855995	4.603559418
ENSMUSG00000049658.13	Bdp1	-0.30762	4.426176	-2.15897	0.047764	0.220003	-4.60625	4.683670867	4.574717068	4.47467018	4.502093351	3.984683344	4.33721849
ENSMUSG00000025986.6	Slc39a10	-0.3074	4.182425	-2.32781	0.034597	0.199507	-4.26788	4.244138805	4.521837508	4.24528456	4.17446915	3.939858144	3.968962396
ENSMUSG00000054766.13	Set	-0.3058	5.992429	-2.76005	0.014772	0.133664	-3.62539	6.007346624	6.285107427	6.14452158	5.938137538	5.768598673	5.810860749
ENSMUSG00000050953.9	Gja1	-0.30506	4.768681	-2.58292	0.021012	0.159596	-3.89178	4.779510064	5.029082446	4.95450516	4.70888107	4.495112823	4.644993508
ENSMUSG00000075376.10	Rc3h2	-0.30476	5.098477	-2.52911	0.023366	0.16819	-4.02389	5.265715884	5.369834415	5.11707457	5.13428006	4.865408195	4.838550011
ENSMUSG00000020029.5	Nudt4	-0.30376	4.328617	-2.23722	0.041168	0.21069	-4.4543	4.542957786	4.654085307	4.25831124	4.301848457	4.033500991	4.180996106
ENSMUSG00000021327.18	Zkscan3	-0.30364	4.423216	-2.36181	0.032395	0.193723	-4.24883	4.677829988	4.384718652	4.64304406	4.391870328	4.126443716	4.315386623
ENSMUSG00000022031.5	Elp3	-0.30219	3.917089	-2.39961	0.030104	0.187803	-4.08584	4.049798765	4.053239764	4.10089465	3.749887879	3.758147229	3.79056382
ENSMUSG00000028330.8	Ncbp1	-0.30153	4.070092	-2.29382	0.036937	0.203223	-4.30608	4.228241179	4.279429753	4.15064768	4.076065446	3.764587842	3.92157899
ENSMUSG00000049470.13	Aff4	-0.30086	5.749961	-2.92629	0.010577	0.109854	-3.30093	5.943057495	5.92383197	5.83460237	5.67005187	5.569645844	5.558578756
ENSMUSG00000039100.9	Mar-06	-0.30006	5.68221	-2.81893	0.013128	0.125115	-3.50679	5.794432289	5.849141636	5.8518933	5.663289225	5.432785827	5.501716419
ENSMUSG00000021068.15	Nin	-0.29943	4.053239	-2.32197	0.034989	0.200218	-4.25273	4.259863158	4.214264707	4.1295347	4.034744387	3.783738942	3.897290621
ENSMUSG00000048578.11	Mlec	-0.29815	5.38256	-2.70323	0.016547	0.142887	-3.71579	5.582572989	5.561677773	5.45075367	5.343336845	5.094845978	5.262172167
ENSMUSG00000034252.14	Senp6	-0.29805	4.849669	-2.40122	0.03001	0.187565	-4.23478	4.94913201	5.076999232	4.9624869	4.90125247	4.707232957	4.500910669
ENSMUSG00000018474.17	Chd3	-0.2968	6.199012	-2.25867	0.039514	0.208294	-4.55134	6.425001653	6.412997428	6.20632403	6.120356696	6.267466148	5.76192873
ENSMUSG00000039982.7	Dtx4	-0.29663	4.842503	-2.16272	0.047427	0.219621	-4.65606	5.061061079	4.904781378	4.99008027	5.001171279	4.455853392	4.642073072
ENSMUSG00000032777.9	Gtf3c1	-0.29639	5.201747	-2.27227	0.038497	0.206554	-4.49707	5.28120806	5.561677773	5.21051914	5.166520397	5.162250566	4.82830901
ENSMUSG00000005410.5	Mcm5	-0.29503	4.294063	-2.39344	0.030467	0.188438	-4.17022	4.510463281	4.376232186	4.42354572	4.29603113	4.059924716	4.098178821
ENSMUSG00000032555.9	Topbp1	-0.29262	4.744427	-2.58484	0.020933	0.159238	-3.88518	4.869377264	4.990681451	4.81600081	4.673421343	4.510522262	4.606558785
ENSMUSG00000045482.15	Trrap	-0.29225	6.13259	-2.27602	0.038222	0.205878	-4.52058	6.333228266	6.296396178	6.20800354	5.987700639	6.249211163	5.720998465
ENSMUSG00000029863.13	Casp2	-0.29158	4.213869	-2.40685	0.029683	0.186632	-4.13151	4.422384123	4.26110957	4.38842767	4.089580253	4.006584258	4.115126975
ENSMUSG00000033364.13	Usp37	-0.29138	3.870409	-2.26048	0.039377	0.208097	-4.32225	3.994480997	4.010009712	4.0342969	3.766811182	3.764587842	3.652268839
ENSMUSG00000045098.18	Kmt5b	-0.29052	4.715805	-2.58256	0.021027	0.159596	-3.88587	4.884670994	4.788074346	4.90979836	4.522165953	4.521972283	4.668146583
ENSMUSG00000049739.10	Zfp646	-0.29042	4.59461	-2.40929	0.029542	0.186335	-4.18876	4.853919669	4.755725569	4.60785464	4.566333537	4.435815639	4.34801177
ENSMUSG00000021670.14	Hmgcr	-0.28866	5.451921	-2.65823	0.018098	0.150179	-3.80428	5.601208706	5.580269461	5.60403292	5.459790066	5.207863692	5.258363069
ENSMUSG00000044791.16	Setd2	-0.28823	5.815082	-2.64629	0.018533	0.152011	-3.83873	5.860380473	5.941196614	6.07443918	5.761543943	5.56411827	5.688812857
ENSMUSG00000034525.6	Ice1	-0.28672	4.617444	-2.25162	0.040051	0.208688	-4.4734	4.774045303	4.781662358	4.72039297	4.541963109	4.638764828	4.247835895
ENSMUSG00000021555.14	Naa35	-0.28654	4.535974	-2.49312	0.025078	0.173779	-4.02726	4.712525729	4.58211552	4.73909917	4.364337144	4.352759899	4.465004641
ENSMUSG00000022000.10	Zc3h13	-0.28652	4.809747	-2.47062	0.026208	0.176849	-4.10378	4.968400344	4.788074346	5.0881836	4.721956946	4.652720874	4.639146711
ENSMUSG00000026491.13	Ahctf1	-0.28485	4.843691	-2.17386	0.046438	0.217853	-4.63695	5.056566698	5.11319778	4.78932464	4.912702491	4.638764828	4.551592326
ENSMUSG00000046311.13	Zfp62	-0.28309	4.175318	-2.33249	0.034285	0.198803	-4.2575	4.379844578	4.242553751	4.32173662	4.076065446	3.968306678	4.063673259
ENSMUSG00000030541.16	Idh2	-0.283	4.627955	-2.45925	0.026798	0.178198	-4.1024	4.827783174	4.838369463	4.64800178	4.541963109	4.423658025	4.487957091
ENSMUSG00000015697.14	Setdb1	-0.28252	4.723448	-2.3939	0.03044	0.18841	-4.2331	4.899804298	4.813441066	4.86791748	4.75201418	4.555786996	4.45172312
ENSMUSG00000022100.13	Xpo7	-0.28213	4.748977	-2.292	0.037066	0.203267	-4.4188	4.822498571	4.874972569	4.95850155	4.822504682	4.463791212	4.551592326
ENSMUSG00000022682.7	Rrn3	-0.27948	4.12026	-2.23472	0.041364	0.211294	-4.41949	4.162838703	4.315386631	4.30300005	4.006524513	3.887706862	4.046105979
ENSMUSG00000024384.3	Iws1	-0.27664	4.860776	-2.59543	0.020499	0.158008	-3.87816	4.992129415	5.029082446	4.9783191	4.726289367	4.687031529	4.751804512
ENSMUSG00000057230.13	Aak1	-0.27394	4.438545	-2.21537	0.042919	0.212526	-4.50985	4.701052945	4.5063661	4.50777362	4.455882086	4.227811807	4.232385198
ENSMUSG00000038416.14	Cdc16	-0.27205	4.290266	-2.22959	0.041771	0.211919	-4.46016	4.365381349	4.537144761	4.37652909	4.260625794	4.091007319	4.110908565
ENSMUSG00000003119.14	Cdk12	-0.27126	4.702846	-2.27904	0.038001	0.205148	-4.43583	4.80116443	4.844534836	4.8593934	4.75201418	4.439845516	4.520125538
ENSMUSG00000063273.11	Naa15	-0.27006	5.427247	-2.47787	0.025839	0.176099	-4.13904	5.475537546	5.594972213	5.61420174	5.404090876	5.17681111	5.297865662
ENSMUSG00000022119.15	Rbm26	-0.26956	4.889185	-2.48075	0.025694	0.175641	-4.0934	5.033882347	4.996230285	5.03618896	4.850557969	4.693796817	4.724454024
ENSMUSG00000041530.16	Ago1	-0.26853	5.847863	-2.45512	0.027016	0.179024	-4.1948	5.913538409	6.035833488	5.99689693	5.831581437	5.562271029	5.747055453
ENSMUSG00000027665.13	Pik3ca	-0.26686	4.848543	-2.33022	0.034436	0.199179	-4.36206	5.07446082	4.874972569	4.99397944	4.713252877	4.6140121	4.820580268
ENSMUSG00000005609.15	Ctr9	-0.26618	4.453175	-2.28096	0.037861	0.205029	-4.39723	4.562108887	4.695604576	4.50777362	4.369885978	4.264628989	4.319048287
ENSMUSG00000024120.12	Lrpprc	-0.26583	4.498247	-2.17581	0.046267	0.217853	-4.58749	4.630231608	4.5063661	4.73444531	4.502093351	4.357028231	4.259316287
ENSMUSG00000001435.14	Col18a1	-0.26471	5.025253	-2.39444	0.030408	0.188388	-4.26395	5.181534797	5.143518232	5.14890002	4.90125247	4.769399503	5.006915112
ENSMUSG00000029328.15	Hnrnpdl	-0.26462	6.182885	-2.5733	0.021416	0.161195	-3.97883	6.338791364	6.224938208	6.3817468	6.070112673	6.078783743	6.002936829
ENSMUSG00000035623.14	Rsf1	-0.26381	4.947352	-2.27149	0.038555	0.206554	-4.47664	5.052058273	5.082226393	5.09546076	5.004742308	4.700530528	4.749092679
ENSMUSG00000035469.17	Rcbtb1	-0.2635	4.707862	-2.22224	0.042361	0.212176	-4.53636	4.919738225	4.916534559	4.6870619	4.677901766	4.491234608	4.554701584
ENSMUSG00000014355.10	Anapc1	-0.26177	5.915524	-2.37016	0.031876	0.192604	-4.35033	6.053186701	6.049241531	6.03713406	5.871245655	5.882534583	5.599801419
ENSMUSG00000035621.13	Midn	-0.26116	6.094585	-2.24032	0.040925	0.210409	-4.58333	6.144955998	6.41714013	6.11439049	6.002064865	5.831300341	6.057658603
ENSMUSG00000026349.14	Ccnt2	-0.25978	5.16665	-2.24436	0.04061	0.209578	-4.54335	5.427614443	5.173214547	5.28242369	5.107958782	5.112625896	4.896065402
ENSMUSG00000028820.13	Sfpq	-0.25879	7.103385	-2.463	0.026602	0.177749	-4.16735	7.164405999	7.282271383	7.25303333	7.060279479	6.944474772	6.915847418
ENSMUSG00000024294.13	Mib1	-0.25851	5.700854	-2.14978	0.048601	0.220972	-4.73496	5.857799622	6.027728438	5.60912629	5.651947225	5.499945976	5.558578756
ENSMUSG00000042446.16	Zmym4	-0.25662	4.957331	-2.31208	0.035662	0.20128	-4.40551	5.139653916	5.071753062	5.04373248	4.920285662	4.707232957	4.86132988
ENSMUSG00000030980.16	Knop1	-0.2528	4.757839	-2.27075	0.03861	0.206554	-4.45719	4.859090625	4.922375438	4.8721607	4.682368317	4.525768851	4.685270761
ENSMUSG00000048154.16	Kmt2d	-0.24893	6.468118	-2.15947	0.047719	0.219973	-4.72217	6.669759972	6.679312453	6.43003623	6.44475164	6.393327669	6.191521286
ENSMUSG00000066232.3	Ipo7	-0.24843	5.056204	-2.29094	0.037142	0.20357	-4.45196	5.257906961	5.182979018	5.1027014	4.953925154	4.85337313	4.986337496
ENSMUSG00000037503.12	Fam168b	-0.22245	6.299099	-2.2292	0.041802	0.211996	-4.60233	6.425001653	6.427445138	6.37876756	6.214340259	6.172579444	6.176461702
ENSMUSG00000025579.14	Gaa	0.222415	5.875888	2.153772	0.048236	0.220594	-4.72644	5.834361823	5.697308882	5.76100227	5.954848478	5.958938886	6.048865006
ENSMUSG00000044496.6	2510039O18R	0.229899	6.551566	2.15809	0.047844	0.220095	-4.72066	6.368104272	6.381542075	6.55933981	6.637643171	6.624229844	6.738535911
ENSMUSG00000021270.13	Hsp90aa1	0.232035	7.212906	2.223741	0.04224	0.212176	-4.59303	7.106882297	7.118931617	7.06288461	7.23918527	7.361549539	7.388000212
ENSMUSG00000029621.14	Arpc1a	0.234726	5.367818	2.251275	0.040077	0.208688	-4.53588	5.197952557	5.277152412	5.27603301	5.520919565	5.426707047	5.508146268
ENSMUSG00000021610.7	Clptm1l	0.235081	6.516395	2.15187	0.048409	0.22073	-4.73176	6.428484323	6.294145483	6.47257578	6.622614962	6.524346308	6.756200399
ENSMUSG00000055401.14	Fbxo6	0.236069	5.83167	2.221922	0.042386	0.212176	-4.60677	5.713953434	5.652329135	5.77239803	5.95853588	5.845040398	6.047762028
ENSMUSG00000040907.15	Atp1a3	0.237755	5.479058	2.173192	0.046497	0.217853	-4.67844	5.385231125	5.230833987	5.45924014	5.548094418	5.567805672	5.683141321
ENSMUSG00000068040.10	Tm9sf4	0.237888	7.10412	2.213314	0.043087	0.21274	-4.61404	6.997415094	6.866666005	7.09228152	7.21857499	7.180729432	7.269050628
ENSMUSG00000047123.8	Ticam1	0.238388	5.338163	2.174434	0.046388	0.217853	-4.66806	5.139653916	5.207104915	5.30770706	5.449345169	5.366586054	5.558578756
ENSMUSG00000034485.9	Uaca	0.238415	5.349846	2.282286	0.037765	0.204879	-4.47982	5.288892206	5.163383538	5.23708274	5.490678561	5.48831909	5.430722821
ENSMUSG00000028081.6	Rps3a1	0.23935	6.637546	2.161407	0.047545	0.219824	-4.71374	6.516202911	6.404676164	6.63120075	6.701748508	6.684910854	6.886534511
ENSMUSG00000000374.7	Trappc10	0.239773	5.201252	2.168727	0.046891	0.218712	-4.66795	5.100892613	4.962612569	5.17317671	5.351773682	5.238261957	5.380791637
ENSMUSG00000001289.12	Pfdn5	0.239943	6.192074	2.152732	0.04833	0.220594	-4.73162	6.032738712	5.958354735	6.22303175	6.335559495	6.208454356	6.394305422
ENSMUSG00000003808.18	Farsa	0.240331	5.880889	2.148049	0.04876	0.221249	-4.73611	5.739413419	5.627515586	5.91082996	5.978649825	5.929147664	6.099776195
ENSMUSG00000063524.13	Eno1	0.240544	7.660241	2.211451	0.04324	0.212931	-4.60208	7.562505177	7.434104136	7.62335573	7.717339449	7.767997759	7.856141172
ENSMUSG00000028063.15	Lmna	0.24455	5.995078	2.185226	0.045449	0.216816	-4.67372	5.933284757	5.74422993	5.93739591	6.044318869	6.044790262	6.266445416
ENSMUSG00000029617.10	Ccz1	0.244616	5.857652	2.27541	0.038266	0.205896	-4.51299	5.664600904	5.680174536	5.85832434	5.919339943	5.960342286	6.063127507
ENSMUSG00000044768.16	D1Ertd622e	0.244714	6.244084	2.297299	0.036691	0.202683	-4.47833	6.127920287	6.022299664	6.21303036	6.35247453	6.293309508	6.455470922
ENSMUSG00000066315.9	Gm12918	0.244731	5.002408	2.165732	0.047158	0.219041	-4.65526	4.919738225	4.886970102	4.83786024	5.067553947	5.040152432	5.262172167
ENSMUSG00000022285.16	Ywhaz	0.244986	7.906951	2.160182	0.047655	0.21983	-4.68386	7.836001808	7.675426794	7.84138132	7.973875319	7.943233287	8.171786443
ENSMUSG00000017774.19	Myo1c	0.245049	7.033731	2.144605	0.049077	0.221815	-4.73487	6.920356744	6.772799082	7.0394941	7.055122387	7.126756224	7.287857412
ENSMUSG00000030629.14	Zfand6	0.245139	5.053235	2.138265	0.049667	0.222533	-4.70733	5.015474505	4.762253612	5.00561414	5.156923721	5.122688325	5.256454742
ENSMUSG00000079339.6	Ifit1bl1	0.245472	5.415789	2.137438	0.049745	0.222657	-4.73653	5.273482767	5.168307416	5.4307556	5.515923213	5.438839102	5.667428646
ENSMUSG00000022969.13	Il10rb	0.245985	6.812633	2.151692	0.048425	0.22073	-4.72747	6.671230609	6.538096093	6.85885897	6.85204554	6.935940195	7.019628653
ENSMUSG00000021065.15	Fut8	0.245988	5.720916	2.180182	0.045885	0.21765	-4.67629	5.585695707	5.464946172	5.73793711	5.813370458	5.76217598	5.96136771
ENSMUSG00000034647.14	Ankrd12	0.246148	5.429748	2.13945	0.049556	0.222531	-4.73383	5.322973202	5.123375565	5.46487018	5.543191457	5.501874713	5.622203966
ENSMUSG00000019054.13	Fis1	0.246176	6.324127	2.182785	0.045659	0.217117	-4.67958	6.215194108	6.051908251	6.33332459	6.396635071	6.378707399	6.568995372
ENSMUSG00000020946.13	Gosr2	0.246242	6.63141	2.350359	0.033122	0.195711	-4.38048	6.476379013	6.441749597	6.60594505	6.721414477	6.720130965	6.822838764
ENSMUSG00000054604.7	Cggbp1	0.246822	6.933837	2.221938	0.042385	0.212176	-4.6029	6.848080104	6.675858962	6.90618193	6.95807547	7.054350026	7.160478106
ENSMUSG00000042492.12	Tbc1d10b	0.246918	7.078386	2.160851	0.047595	0.219824	-4.70578	6.863600057	6.852969431	7.14933713	7.191412097	7.151540086	7.261458727
ENSMUSG00000048832.8	Vps37c	0.247482	5.922666	2.219488	0.042583	0.212347	-4.61282	5.693835846	5.750810078	5.94949406	6.087055851	5.939146581	6.11565341
ENSMUSG00000030682.4	Cdipt	0.247534	6.14602	2.206916	0.043615	0.213843	-4.63736	6.021252226	5.882482711	6.16022216	6.283590873	6.164078691	6.364494802
ENSMUSG00000001052.15	Sec24b	0.247645	6.557708	2.24858	0.040284	0.209198	-4.56262	6.325777274	6.373036847	6.60339499	6.72249921	6.644450764	6.677090784
ENSMUSG00000071068.6	Treml2	0.247728	5.180733	2.23529	0.04132	0.211226	-4.55018	4.987414722	5.001757859	5.1766117	5.262118495	5.263492561	5.393003155
ENSMUSG00000079316.10	Rab9	0.247994	5.915574	2.162499	0.047447	0.219624	-4.71177	5.802507096	5.638202265	5.92927375	5.978649825	5.967338881	6.177470579
ENSMUSG00000042659.14	Arrdc4	0.248086	5.282572	2.216867	0.042797	0.212429	-4.59023	5.193865599	5.09262424	5.18686787	5.382293369	5.290522767	5.549255977
ENSMUSG00000018001.18	Cyth3	0.248323	5.076494	2.333071	0.034247	0.198772	-4.3681	4.968400344	4.990681451	4.90151888	5.204281202	5.154914812	5.239165247
ENSMUSG00000027363.15	Usp8	0.248558	6.423595	2.18301	0.04564	0.217113	-4.67863	6.245126614	6.164616084	6.48648138	6.51904796	6.495596867	6.63070021
ENSMUSG00000034575.11	Papd7	0.248559	6.052254	2.212498	0.043154	0.212903	-4.62684	5.885938183	5.808718656	6.08544517	6.081993758	6.177414622	6.274011189
ENSMUSG00000056220.14	Pla2g4a	0.248607	6.306516	2.135521	0.049925	0.222753	-4.76124	6.24315032	6.014118007	6.28640354	6.389823467	6.319791843	6.585811081
ENSMUSG00000034266.5	Batf	0.248765	6.902707	2.179598	0.045936	0.217758	-4.67735	6.72885964	6.66544861	6.93992474	6.957154209	6.972564149	7.152291129

ENSMUSG00000029265.4	Dr1	0.248775	6.354469	2.219648	0.04257	0.212347	-4.61512	6.284094413	6.062526109	6.34099852	6.41016255	6.452366815	6.576663182
ENSMUSG00000029310.13	Nudt9	0.249014	5.71207	2.156305	0.048006	0.220331	-4.71725	5.505525358	5.488760111	5.76328863	5.784577299	5.765390901	5.964877918
ENSMUSG00000071660.10	Ttc9c	0.249334	5.984581	2.304766	0.036168	0.202683	-4.46311	5.786312033	5.780054898	6.01043438	6.087055851	6.081365748	6.162262831
ENSMUSG00000070427.3	Il18bp	0.249503	7.61635	2.148991	0.048673	0.220972	-4.71163	7.5001568	7.328092815	7.64769243	7.69706577	7.688937475	7.836156291
ENSMUSG00000031608.13	Galnt7	0.249628	5.859364	2.192207	0.044851	0.215717	-4.65904	5.842216806	5.569143223	5.78820236	5.926888397	5.916189238	6.113546517
ENSMUSG00000035158.15	Mitf	0.249636	5.980146	2.140694	0.04944	0.222432	-4.75041	5.823821538	5.687052713	6.05030086	6.08537046	6.036830545	6.197501377
ENSMUSG00000069682.5	Gm10275	0.250687	6.906733	2.189918	0.045046	0.216249	-4.65933	6.857153688	6.626621446	6.86099551	7.079032647	6.891757827	7.124835062
ENSMUSG00000017831.7	Rab5a	0.250843	6.368865	2.334544	0.03415	0.198465	-4.4114	6.286015446	6.167078021	6.27523218	6.44475164	6.410883946	6.629226192
ENSMUSG00000028525.16	Pde4b	0.250877	6.665474	2.200612	0.04414	0.214453	-4.64521	6.447490161	6.4679445	6.7032284	6.726830006	6.724267135	6.923083521
ENSMUSG00000040940.18	Arhgef1	0.251301	6.391885	2.147496	0.04881	0.221249	-4.7402	6.247100206	6.104232935	6.44578008	6.543805239	6.39747782	6.612911811
ENSMUSG00000003308.14	Keap1	0.251415	6.57602	2.14293	0.049232	0.222208	-4.74631	6.524362973	6.264562372	6.55933981	6.581358616	6.693374915	6.833118532
ENSMUSG00000057531.13	Dtnbp1	0.251778	6.409148	2.206703	0.043632	0.213843	-4.63744	6.227241736	6.176883972	6.44293032	6.43552047	6.505243811	6.667069543
ENSMUSG00000058126.6	Tpm3-rs7	0.251795	7.411967	2.21233	0.043168	0.212903	-4.60759	7.29319662	7.154105724	7.41084106	7.465956132	7.483690769	7.664013556
ENSMUSG00000036057.11	Ptpn23	0.251815	6.205252	2.300523	0.036464	0.202683	-4.47241	6.046402856	5.986505775	6.20295915	6.232728548	6.434301528	6.328616172
ENSMUSG00000024566.8	Atp9b	0.251855	6.415654	2.274758	0.038314	0.206068	-4.51771	6.303190589	6.132222606	6.43291156	6.571760609	6.481004413	6.572834371
ENSMUSG00000040712.16	Camta2	0.252037	6.328414	2.344545	0.033496	0.196453	-4.39365	6.270575084	6.070438533	6.26397363	6.387089795	6.448371811	6.530032835
ENSMUSG00000029518.15	Rab35	0.252125	6.161635	2.301304	0.036409	0.202683	-4.47088	5.97912675	5.92383197	6.20127376	6.268816601	6.266331954	6.330431454
ENSMUSG00000043131.14	Mob1a	0.252497	7.513014	2.149687	0.048609	0.220972	-4.7134	7.27299856	7.312609546	7.57471736	7.544725423	7.628870515	7.74416445
ENSMUSG00000066568.12	Lsm14a	0.252645	6.582692	2.196619	0.044476	0.214943	-4.6533	6.489776161	6.282839038	6.59443412	6.622614962	6.682361922	6.824127748
ENSMUSG00000032905.4	Atg12	0.253012	5.522294	2.207005	0.043607	0.213843	-4.62149	5.417134772	5.235533323	5.52808407	5.591487014	5.591546759	5.769977258
ENSMUSG00000030720.16	Cln3	0.253067	6.345251	2.13575	0.049903	0.222753	-4.76061	6.188740567	6.080921377	6.38323411	6.395275321	6.378707399	6.644628776
ENSMUSG00000040462.12	Os9	0.253252	7.116186	2.307835	0.035955	0.202085	-4.44682	6.94731234	6.899609098	7.12198246	7.213954666	7.179524928	7.334733616
ENSMUSG00000017466.9	Timp2	0.253522	7.169303	2.209233	0.043423	0.213518	-4.6195	7.058253732	6.877229719	7.192393	7.221647006	7.279602753	7.386692238
ENSMUSG00000002233.13	Rhoc	0.254602	7.430933	2.14958	0.048619	0.220972	-4.71589	7.26230468	7.16646293	7.48267068	7.50177749	7.478802758	7.693580934
ENSMUSG00000001418.13	Glmp	0.254661	6.75778	2.143619	0.049169	0.222153	-4.74232	6.569225511	6.477894161	6.84381397	6.847080996	6.818589289	6.990074678
ENSMUSG00000057193.7	Slc44a2	0.255033	6.041134	2.152883	0.048317	0.220594	-4.7301	5.94062051	5.697308882	6.09818026	6.093777797	6.228394361	6.188521919
ENSMUSG00000024835.14	Coro1b	0.25542	8.127424	2.171634	0.046634	0.217969	-4.65757	7.988299901	7.872334756	8.13858953	8.184540706	8.182684609	8.398097002
ENSMUSG00000062526.3	Mppe1	0.255558	5.407265	2.151032	0.048486	0.220866	-4.71236	5.326710756	5.071753062	5.4307556	5.488129629	5.480515446	5.645722694
ENSMUSG00000022214.14	Dcaf11	0.255737	5.53068	2.246331	0.040457	0.209437	-4.55295	5.261816706	5.352633674	5.58864403	5.687932131	5.611334657	5.681719947
ENSMUSG00000038774.8	Ascc3	0.255819	6.552863	2.441033	0.02777	0.18063	-4.21698	6.387916875	6.355874473	6.53009948	6.622614962	6.707650826	6.713019129
ENSMUSG00000025337.10	Sbds	0.255856	7.722684	2.210776	0.043296	0.213127	-4.60144	7.509230384	7.49710083	7.77891005	7.802383796	7.812968518	7.935510944
ENSMUSG00000025510.13	Cd151	0.255989	5.382192	2.155963	0.048037	0.220331	-4.7023	5.307925399	5.034485804	5.41047642	5.43088264	5.558569436	5.550813963
ENSMUSG00000031443.7	F7	0.256142	5.886836	2.251726	0.040042	0.208688	-4.5553	5.742214769	5.605901773	5.92315198	5.951151628	5.983993554	6.114600348
ENSMUSG00000034271.15	Jdp2	0.256211	5.742166	2.225398	0.042107	0.212176	-4.59793	5.610437064	5.448848989	5.77693124	5.780416676	5.869148656	5.967213322
ENSMUSG00000002897.5	Il17ra	0.256226	7.528789	2.135264	0.049949	0.222753	-4.73774	7.372179045	7.226709473	7.60435179	7.575067131	7.656405367	7.738023135
ENSMUSG00000026315.13	Serpinb8	0.256559	7.100185	2.169041	0.046863	0.218658	-4.69107	6.887836883	6.845303658	7.18303035	7.170102757	7.185537418	7.32929717
ENSMUSG00000019428.16	Fkbp8	0.256869	7.395952	2.329111	0.03451	0.199349	-4.40147	7.271060105	7.132852466	7.39986159	7.498615071	7.502597326	7.570724186
ENSMUSG00000035356.16	Nfkbiz	0.257249	6.429345	2.135463	0.04993	0.222753	-4.7606	6.325777274	6.088733824	6.48509684	6.511537166	6.487832597	6.677090784
ENSMUSG00000024981.5	Acsl5	0.257412	7.718105	2.179403	0.045953	0.217758	-4.65615	7.576699613	7.446317501	7.74518466	7.746959088	7.811025232	7.982446303
ENSMUSG00000042079.13	Hnrnpf	0.257491	6.375368	2.400396	0.030058	0.187691	-4.2925	6.27251419	6.224938208	6.24118948	6.496397245	6.377657402	6.639512831
ENSMUSG00000005625.15	Psmd4	0.257767	6.593033	2.383108	0.031085	0.189975	-4.32184	6.440607882	6.342867367	6.6084906	6.701748508	6.710989434	6.753496823
ENSMUSG00000026656.15	Fcgr2b	0.257811	7.407618	2.173706	0.046451	0.217853	-4.67488	7.253495795	7.111281426	7.47292506	7.511223366	7.467003517	7.629779125
ENSMUSG00000023952.13	Gtpbp2	0.25784	7.287403	2.271148	0.03858	0.206554	-4.50757	7.111222865	7.025695041	7.33985003	7.375068698	7.415239835	7.457341179
ENSMUSG00000030966.12	Trim21	0.258114	6.951959	2.197532	0.044399	0.214943	-4.64509	6.740114266	6.693044336	7.03571618	7.019380077	7.058942511	7.164554238
ENSMUSG00000020178.5	Adora2a	0.258636	6.054766	2.138816	0.049616	0.222531	-4.75442	5.916021533	5.74422993	6.11080392	6.073517265	6.148157446	6.335863635
ENSMUSG00000019189.13	Rnf145	0.25874	5.910759	2.535904	0.023055	0.167189	-4.04096	5.850029253	5.737619632	5.75641865	6.073517265	6.039488667	6.007482544
ENSMUSG00000035778.17	Ggta1	0.258896	7.62159	2.247439	0.040372	0.20925	-4.54009	7.431524777	7.387360468	7.65817359	7.703122896	7.687243425	7.862114422
ENSMUSG00000063229.14	Ldha	0.259075	9.571772	2.145429	0.049001	0.22162	-4.65936	9.488004	9.269582941	9.57023554	9.65130658	9.672067391	9.779435402
ENSMUSG00000018217.12	Pmp22	0.259114	7.069637	2.176397	0.046215	0.217853	-4.67907	6.973773516	6.733472762	7.11394266	7.174072751	7.13423619	7.288324455
ENSMUSG00000018820.13	Zfyve27	0.259131	4.947167	2.193445	0.044745	0.215501	-4.60081	4.642279235	4.844534836	4.9624869	5.039974707	5.040152432	5.15357341
ENSMUSG00000028452.7	Vcp	0.259164	6.087574	2.215107	0.04294	0.212526	-4.62248	5.933284757	5.824400278	6.11259832	6.209706331	6.080075323	6.365380434
ENSMUSG00000027131.3	Emc4	0.259164	5.374267	2.21477	0.042968	0.212526	-4.5997	5.315468919	5.082226393	5.32947227	5.495762954	5.381329051	5.641342038
ENSMUSG00000034765.6	Dusp5	0.259195	5.115202	2.222194	0.042364	0.212176	-4.5671	4.996828752	4.794457963	5.15239323	5.238047946	5.235946234	5.273539488
ENSMUSG00000035227.6	Spcs2	0.25926	5.740021	2.301184	0.036418	0.202683	-4.46391	5.595023509	5.476902277	5.75412136	5.780416676	5.857144884	5.976517312
ENSMUSG00000005779.12	Psmb4	0.259275	7.277697	2.348998	0.033209	0.196054	-4.36898	7.098161787	7.063187138	7.28282217	7.346506444	7.373186372	7.502320436
ENSMUSG00000037966.15	Ninj1	0.259284	8.411072	2.147211	0.048837	0.221249	-4.69127	8.220979486	8.138195205	8.48665435	8.492110359	8.504161745	8.624332722
ENSMUSG00000026663.6	Atf6	0.259299	5.704373	2.332319	0.034297	0.198803	-4.40712	5.547769448	5.444796473	5.72626476	5.821492619	5.773396998	5.912518702
ENSMUSG00000033940.8	Brk1	0.25956	6.468265	2.147217	0.048836	0.221249	-4.74009	6.270575084	6.222573134	6.51931796	6.503987066	6.506204967	6.786932736
ENSMUSG00000042165.8	Gm9774	0.259782	5.012427	2.307723	0.035962	0.202085	-4.40657	4.822498571	4.825959106	4.99397944	5.101302738	5.089725493	5.241096566
ENSMUSG00000022749.6	Tbc1d23	0.259793	5.472329	2.294097	0.036918	0.203223	-4.46545	5.326710756	5.230833987	5.46487018	5.477888628	5.623787096	5.709884727
ENSMUSG00000028670.14	Lypla2	0.26001	5.815729	2.340356	0.033769	0.197192	-4.39595	5.705365938	5.527596778	5.81930091	5.875152752	5.961744322	6.005211477
ENSMUSG00000027562.12	Car2	0.26007	6.829971	2.255437	0.039759	0.208663	-4.54613	6.643028882	6.583037665	6.87269023	6.878562805	6.915827615	7.08667892
ENSMUSG00000082292.3	Gm12250	0.260112	7.627403	2.221604	0.042412	0.212176	-4.58527	7.452202755	7.373569895	7.66674832	7.694856859	7.691897284	7.885145746
ENSMUSG00000035828.10	Pim3	0.260241	4.973099	2.24556	0.040517	0.209437	-4.51225	4.827783174	4.722634782	4.96646126	5.185524339	4.997043491	5.13914634
ENSMUSG00000027351.14	Spred1	0.260249	7.947158	2.247925	0.040334	0.209217	-4.52977	7.802842568	7.67715499	7.97215531	8.03670798	8.018253738	8.175830795
ENSMUSG00000028857.16	Tmem222	0.260492	4.508494	2.172646	0.046545	0.217853	-4.57796	4.483930211	4.279429753	4.37054279	4.585537748	4.5960679	4.735456518
ENSMUSG00000045502.6	Hcar2	0.26052	7.727079	2.243784	0.040655	0.209664	-4.54348	7.579835056	7.460436964	7.75095661	7.81209723	7.783937101	7.975212558
ENSMUSG00000037096.6	Gm9762	0.260565	4.948961	2.298236	0.036625	0.202683	-4.4168	4.859090625	4.715924561	4.87639148	4.983182329	5.179223644	5.079950547
ENSMUSG00000062488.9	Ifit3b	0.260884	6.646646	2.293895	0.036932	0.203223	-4.48115	6.611203436	6.360184245	6.57504277	6.686265239	6.734966006	6.912215711
ENSMUSG00000030965.18	Abraxas2	0.260942	4.897566	2.205099	0.043766	0.214027	-4.57484	4.746406622	4.611335659	4.93029153	5.011857957	4.977770561	5.107736644
ENSMUSG00000017765.16	Slc12a4	0.260971	5.676236	2.407019	0.029673	0.186632	-4.27133	5.495598519	5.46894256	5.66888407	5.742421048	5.786115053	5.895452402
ENSMUSG00000020219.6	Timm13	0.261036	5.444668	2.44556	0.027525	0.180155	-4.19084	5.315468919	5.202311004	5.41920243	5.557850635	5.591546759	5.581625493
ENSMUSG00000015355.13	Cd48	0.261051	8.001746	2.165798	0.047152	0.219041	-4.67136	7.834689969	7.715504134	8.06450783	8.064349143	8.086355272	8.245071765
ENSMUSG00000024847.14	Aip	0.261068	6.170712	2.292106	0.037059	0.203267	-4.48716	6.053186701	5.867423127	6.19620573	6.214340259	6.319791843	6.373326755
ENSMUSG00000079477.9	Rab7	0.261151	8.202754	2.145212	0.049021	0.221636	-4.70089	8.086359974	7.890326509	8.24098442	8.2819363	8.258224907	8.458690414
ENSMUSG00000037075.6	Rnf139	0.261365	6.15488	2.231883	0.041589	0.211556	-4.59352	5.903562949	5.941196614	6.22469193	6.247875092	6.207272787	6.404681458
ENSMUSG00000016831.11	Tox4	0.261403	6.693519	2.312818	0.035611	0.20128	-4.44668	6.550169273	6.447836927	6.68886715	6.764179966	6.739059922	6.971000307
ENSMUSG00000048758.13	Rpl29	0.261455	6.428015	2.265604	0.038992	0.207552	-4.53374	6.194888424	6.224938208	6.4697785	6.492587309	6.486859118	6.699036029
ENSMUSG00000034226.7	Rhov	0.26158	5.54458	2.207502	0.043566	0.213843	-4.62155	5.538130024	5.207104915	5.48993694	5.562704113	5.692952889	5.776650235
ENSMUSG00000028480.14	Glipr2	0.261605	7.986267	2.161476	0.047539	0.219824	-4.67928	7.865849498	7.671963878	8.0293184	8.014738695	8.116879545	8.218851259
ENSMUSG00000044080.9	S100a1	0.261623	5.235514	2.174918	0.046345	0.217853	-4.65931	5.015474505	4.968270204	5.3201845	5.300396323	5.349550343	5.459209014
ENSMUSG00000025314.16	Ptprj	0.261983	7.179894	2.222495	0.04234	0.212176	-4.59603	7.041293956	6.893675126	7.21261212	7.303661571	7.192122345	7.436001623
ENSMUSG00000036138.16	Acaa1a	0.261993	6.915809	2.186266	0.045359	0.216675	-4.66544	6.795106919	6.590393678	6.9689397	7.006079168	6.969779695	7.164554238
ENSMUSG00000029119.9	Man2b2	0.262068	5.782205	2.256109	0.039708	0.208663	-4.54502	5.699612411	5.43257017	5.81489909	5.919339943	5.885492441	5.941313572
ENSMUSG00000060450.13	Rnf14	0.262176	6.571019	2.378219	0.031381	0.190935	-4.33091	6.393273422	6.347216112	6.57764339	6.636492696	6.658352179	6.813134501
ENSMUSG00000036275.13	9530068E07Ri	0.262179	7.865241	2.135313	0.049944	0.222753	-4.72786	7.686945999	7.590852186	7.92494018	7.90865766	7.924301884	8.155750677
ENSMUSG00000034484.7	Snx2	0.262279	8.295819	2.147254	0.048833	0.221249	-4.69462	8.162577322	7.993805296	8.33850897	8.362424792	8.355829038	8.561769598
ENSMUSG00000026712.3	Mrc1	0.262533	6.798824	2.203458	0.043902	0.214299	-4.63788	6.592697207	6.513155265	6.89684071	6.888261632	6.897629909	7.004358904
ENSMUSG00000026466.16	Tor1aip1	0.262659	8.155676	2.158855	0.047775	0.220003	-4.6788	7.980610241	7.868558065	8.22489932	8.212604279	8.248206151	8.399178482
ENSMUSG00000031765.7	Mt1	0.262925	7.52682	2.22166	0.042408	0.212176	-4.58805	7.348475465	7.252441238	7.58574025	7.566031433	7.648156411	7.760077934
ENSMUSG00000019818.15	Cd164	0.263013	8.59844	2.162689	0.04743	0.219621	-4.65904	8.484139403	8.293300567	8.62414215	8.66371282	8.669442019	8.855904878
ENSMUSG00000058594.15	Fbxo18	0.263313	5.70509	2.189297	0.045099	0.216249	-4.65959	5.670495478	5.352633674	5.69067015	5.707786046	5.879570647	5.929385475
ENSMUSG00000002325.14	Irf9	0.263468	6.200604	2.222057	0.042376	0.212176	-4.61085	6.132198111	5.852204684	6.21803972	6.247875092	6.315411741	6.437897274
ENSMUSG00000050721.8	Plekho2	0.263972	8.616665	2.199335	0.044248	0.214663	-4.59501	8.450276736	8.345315149	8.65924822	8.68473556	8.698534736	8.861879104
ENSMUSG00000022150.16	Dab2	0.264279	7.854141	2.14949	0.048627	0.220972	-4.70383	7.72921268	7.530945765	7.90695764	7.922883199	7.91564677	8.11920183
ENSMUSG00000073987.4	Ggh	0.264511	5.012393	2.217475	0.042747	0.212429	-4.56554	4.889733087	4.729313938	5.01331881	5.018938684	5.164687551	5.258363069
ENSMUSG00000004043.14	Stat5a	0.264533	6.771166	2.227815	0.041913	0.212176	-4.59578	6.586475332	6.491709404	6.83840251	6.898855568	6.79361369	7.017939974
ENSMUSG00000020611.14	Gna13	0.264746	8.92383	2.161418	0.047544	0.219824	-4.65149	8.799316221	8.611561628	8.96459964	8.982616544	9.031249191	9.153637495
ENSMUSG00000020590.15	Snx13	0.265092	5.637566	2.179363	0.045956	0.217758	-4.67438	5.448348142	5.356953124	5.70263286	5.731686661	5.658784447	5.926987976
ENSMUSG00000064289.15	Tank	0.265264	7.768406	2.212059	0.04319	0.212903	-4.59786	7.582963699	7.489252079	7.83650369	7.86118515	7.831492074	8.009041831
ENSMUSG00000030403.9	Vasp	0.265281	8.077114	2.166284	0.047109	0.219041	-4.6683	7.895721344	7.770777084	8.16908701	8.17109628	8.175754357	8.2802467
ENSMUSG00000001783.3	Rtcb	0.265459	5.745526	2.28842	0.037323	0.204088	-4.48666	5.719650164	5.391050901	5.72156924	5.888744866	5.825151392	5.926987976
ENSMUSG00000082016.3	Pgam1-ps2	0.265544	6.537605	2.337674	0.033944	0.197603	-4.40429	6.51783862	6.23670577	6.45853484	6.660462073	6.573777671	6.778313668
ENSMUSG00000020407.13	Upp1	0.265666	8.590743	2.211714	0.043218	0.212903	-4.57421	8.436938273	8.307318313	8.63073125	8.68640422	8.651309607	8.831757461
ENSMUSG00000038147.13	Cd84	0.265776	7.165968	2.142551	0.049268	0.222208	-4.73509	7.138059484	6.792067416	7.16930255	7.223180565	7.247775217	7.425424633
ENSMUSG00000038312.10	Edem2	0.26585	7.05953	2.252369	0.039993	0.208688	-4.54649	6.925295371	6.769562524	7.084074	7.081571114	7.161945653	7.334733616
ENSMUSG00000042349.13	Ikbke	0.265888	7.762227	2.205626	0.043722	0.214017	-4.60927	7.634384337	7.44530366	7.80910125	7.811587624	7.869335425	8.003648042
ENSMUSG00000011179.7	Odc1	0.265953	6.871105	2.447524	0.02742	0.180155	-4.1999	6.724616417	6.630194687	6.85885897	6.933929382	6.993963368	7.08506697
ENSMUSG00000079442.12	St6galnac4	0.266042	7.781641	2.156078	0.048026	0.220331	-4.6946	7.6396524	7.458428338	7.84893613	7.842348802	7.859215153	8.041264948
ENSMUSG00000053846.4	Lipg	0.266066	8.291728	2.138827	0.049615	0.222531	-4.70921	8.16623236	7.954440651	8.35679287	8.341396435	8.408701059	8.52280722
ENSMUSG00000031444.16	F10	0.266101	8.46442	2.160216	0.047652	0.21983	-4.66729	8.299629257	8.163691773	8.53194446	8.527295956	8.552417704	8.711539843
ENSMUSG00000012519.14	Mlkl	0.266143	7.566125	2.213274	0.04309	0.21274	-4.60159	7.405253428	7.267433408	7.62775419	7.655643164	7.624451167	7.816214586
ENSMUSG00000031596.15	Slc7a2	0.266189	8.892762	2.175259	0.046315	0.217853	-4.62852	8.762535977	8.583268109	8.93448227	8.971253998	8.97543	9.12959956
ENSMUSG00000018930.3	Ccl4	0.266335	8.214717	2.188763	0.045145	0.216249	-4.6253	8.052904676	7.908832343	8.28461397	8.30166702	8.300391426	8.439895539
ENSMUSG00000030921.16	Trim30a	0.266572	9.294101	2.146118	0.048937	0.221481	-4.66663	9.151424606	9.003308681	9.32802499	9.337773236	9.379166532	9.564905211
ENSMUSG00000031278.12	Acsl4	0.266817	5.269615	2.204283	0.043833	0.214195	-4.61072	5.14389714	4.962612569	5.29195695	5.406792553	5.272559017	5.539872562

ENSMUSG00000063268.12	Parp10	0.266851	7.719059	2.270807	0.038606	0.206554	-4.49605	7.569619852	7.423846752	7.76471577	7.796730193	7.8191695	7.940273792
ENSMUSG00000039621.13	Prex1	0.266868	8.274872	2.171224	0.04667	0.217984	-4.65391	8.167795975	7.941556164	8.31649151	8.363815845	8.339743821	8.519827265
ENSMUSG00000022024.9	Sugt1	0.266941	5.637575	2.290272	0.03719	0.203612	-4.47966	5.505525358	5.420239368	5.58347771	5.581956376	5.809663373	5.924586485
ENSMUSG00000029007.8	Agtrap	0.266955	6.625938	2.297128	0.036703	0.202683	-4.47564	6.456047096	6.336319572	6.68404813	6.730069592	6.69253074	6.856613645
ENSMUSG00000028563.16	Tm2d1	0.267073	4.273723	2.156333	0.048003	0.220331	-4.56482	4.275417972	4.010009712	4.1295347	4.386405567	4.419582612	4.421384956
ENSMUSG00000040990.17	Sh3kbp1	0.267199	6.681514	2.14154	0.049362	0.222295	-4.74711	6.56764707	6.320924814	6.75354647	6.736527021	6.759357036	6.951080546
ENSMUSG00000028382.15	Ptbp3	0.267213	7.042874	2.425573	0.02862	0.183246	-4.23624	6.936345855	6.784070194	7.00706191	7.132240915	7.112941411	7.284583873
ENSMUSG00000043895.5	S1pr2	0.267272	6.616393	2.251291	0.040075	0.208688	-4.55696	6.442331532	6.303127271	6.70203707	6.72249921	6.706814966	6.821548628
ENSMUSG00000031103.12	Elf4	0.267381	6.309471	2.356537	0.032728	0.194499	-4.372	6.180502457	6.00313645	6.34099852	6.385721014	6.43631999	6.510149734
ENSMUSG00000032046.15	Abhd12	0.267382	7.10171	2.300844	0.036442	0.202683	-4.45959	6.937568478	6.806351642	7.16152287	7.227007344	7.178319417	7.299488361
ENSMUSG00000021583.6	Erap1	0.267638	5.761675	2.409161	0.02955	0.186335	-4.27013	5.64377814	5.492691163	5.74257974	5.94744528	5.784531414	5.959022817
ENSMUSG00000010755.16	Cars	0.267692	5.571243	2.15611	0.048023	0.220331	-4.7118	5.560522636	5.197502916	5.54678594	5.557850635	5.766995679	5.797798686
ENSMUSG00000023048.13	Prr13	0.267773	7.02101	2.238818	0.041042	0.210441	-4.57126	6.851975772	6.713400159	7.09682115	7.128972276	7.082977158	7.251912323
ENSMUSG00000022867.9	Usp25	0.267898	8.252901	2.262422	0.039231	0.208097	-4.49523	8.110410003	7.974959955	8.27182368	8.304567275	8.34539407	8.510249834
ENSMUSG00000060703.12	Cd302	0.26791	5.157286	2.146671	0.048886	0.221375	-4.70149	4.968400344	4.874972569	5.21720603	5.1728829	5.254368768	5.455886745
ENSMUSG00000045322.10	Tlr9	0.26803	6.206451	2.261596	0.039293	0.208097	-4.54127	5.883402691	6.059878962	6.2720244	6.311251867	6.274252653	6.437897274
ENSMUSG00000009291.12	Pttg1ip	0.268142	6.683875	2.184318	0.045527	0.216939	-4.67314	6.551766943	6.358030968	6.73735573	6.700648054	6.780961858	6.974487185
ENSMUSG00000018585.9	Atox1	0.268314	5.732007	2.222132	0.042369	0.212176	-4.60311	5.610437064	5.403632623	5.77239803	5.817437255	5.7749929	6.01314462
ENSMUSG00000053175.16	Bcl3	0.268539	6.980017	2.252471	0.039985	0.208688	-4.54816	6.840257066	6.654962592	7.04232107	7.057703237	7.075225924	7.2096343
ENSMUSG00000005087.17	Cd44	0.268582	7.617239	2.165104	0.047214	0.219095	-4.6838	7.394609457	7.337959309	7.71715328	7.659612846	7.724473632	7.869624401
ENSMUSG00000037447.16	Arid5a	0.268659	7.388239	2.304125	0.036212	0.202683	-4.44628	7.219723767	7.110002441	7.43255246	7.445737199	7.496806269	7.624610155
ENSMUSG00000002768.7	Mea1	0.268741	4.695994	2.298008	0.036641	0.202683	-4.38613	4.549369775	4.5063661	4.62806786	4.721956946	4.825921445	4.94428025
ENSMUSG00000026749.11	Nek6	0.268929	7.509985	2.321964	0.034989	0.200218	-4.41107	7.342026626	7.245468876	7.5404678	7.642522478	7.543901387	7.745525644
ENSMUSG00000027555.8	Car13	0.268968	8.06748	2.182133	0.045716	0.217275	-4.64115	7.918190767	7.768346941	8.11282246	8.113137534	8.133769826	8.358613715
ENSMUSG00000014361.5	Mertk	0.269	7.409762	2.303686	0.036243	0.202683	-4.44647	7.295105579	7.118931617	7.41157006	7.449017544	7.505964738	7.677982162
ENSMUSG00000020440.13	Arf5	0.269018	7.568368	2.342207	0.033648	0.196741	-4.37317	7.364017958	7.324788932	7.61388505	7.664700726	7.657703533	7.785113761
ENSMUSG00000090841.1	Myl6	0.269159	7.059418	2.201981	0.044026	0.214334	-4.63481	6.863600057	6.756543191	7.1545722	7.105050541	7.177716283	7.299024919
ENSMUSG00000017707.9	Serinc3	0.269318	8.464187	2.180267	0.045878	0.21765	-4.63264	8.306743152	8.150066734	8.53328479	8.541194859	8.550322455	8.703507874
ENSMUSG00000024789.12	Jak2	0.269406	8.77096	2.165789	0.047153	0.219041	-4.64853	8.597539445	8.485558226	8.82628867	8.830701477	8.835225784	9.050449061
ENSMUSG00000035969.15	Rusc2	0.269484	6.470389	2.255299	0.039769	0.208663	-4.55155	6.278315921	6.164616084	6.56196887	6.54625776	6.582005992	6.689166589
ENSMUSG00000004451.14	Ralb	0.269624	5.815192	2.404072	0.029844	0.186992	-4.28074	5.742214769	5.496611532	5.79715627	5.915550853	5.921962913	6.017658335
ENSMUSG00000021457.14	Syk	0.269717	8.8117	2.169081	0.04686	0.218658	-4.64162	8.683853014	8.493427044	8.85417424	8.874786911	8.888536395	9.075424918
ENSMUSG00000066363.12	Serpina3f	0.269773	7.642492	2.163623	0.047346	0.219385	-4.68563	7.477640294	7.321477465	7.72479905	7.700372809	7.719509515	7.911154738
ENSMUSG00000038462.3	Uqcrfs1	0.269793	6.231878	2.555844	0.022166	0.164159	-4.00679	6.095425782	6.019577597	6.17403623	6.344041802	6.294422698	6.463764679
ENSMUSG00000010045.2	Tmem115	0.269921	4.989414	2.313681	0.035552	0.20128	-4.39307	4.848730113	4.688767146	5.01331881	5.094615843	5.105032746	5.186017674
ENSMUSG00000026827.12	Gpd2	0.269949	6.177197	2.405961	0.029734	0.186632	-4.28239	5.976749987	5.955509182	6.19111985	6.208158374	6.302191075	6.429453029
ENSMUSG00000044701.5	Il27	0.270054	9.270377	2.186323	0.045354	0.216675	-4.59799	9.105659156	8.984587877	9.3167834	9.349932335	9.339271966	9.526028574
ENSMUSG00000020707.6	Rnf135	0.270167	6.15248	2.171689	0.046629	0.217969	-4.69835	5.993305685	5.824400278	6.22966103	6.231205111	6.185837386	6.450471685
ENSMUSG00000052310.9	Slc39a1	0.270262	7.813595	2.282412	0.037756	0.204879	-4.47276	7.670127772	7.525199876	7.84084018	7.893327534	7.876043047	8.076033665
ENSMUSG00000031729.5	Ist1	0.270274	7.496812	2.391798	0.030565	0.188589	-4.28579	7.371274534	7.211285777	7.50333508	7.567239468	7.634155925	7.693580934
ENSMUSG00000052837.5	Junb	0.270325	8.899725	2.20206	0.044019	0.214334	-4.58181	8.735728372	8.601130276	8.95837683	8.992317267	8.977164113	9.133633799
ENSMUSG00000018167.10	Stard3	0.270361	7.644916	2.301243	0.036414	0.202683	-4.44419	7.510052433	7.339051433	7.68133033	7.724936882	7.742938411	7.871184072
ENSMUSG00000000290.13	Itgb2	0.270395	9.403864	2.141184	0.049395	0.222351	-4.67176	9.262426648	9.089869613	9.45456108	9.4584072	9.492872001	9.665047923
ENSMUSG00000029780.14	Nt5c3	0.27043	7.460614	2.245852	0.040494	0.209437	-4.54744	7.287454543	7.206506659	7.48197674	7.529311868	7.479292305	7.779144666
ENSMUSG00000043801.6	Oaz1-ps	0.270466	7.127659	2.230301	0.041715	0.211874	-4.58363	7.059377327	6.806351642	7.11214993	7.272894632	7.105984078	7.409197612
ENSMUSG00000062593.15	Gm49339	0.270569	8.923753	2.155142	0.048111	0.220397	-4.6623	8.739588662	8.6472682	8.97868809	8.960950625	9.000543419	9.215478541
ENSMUSG00000029713.15	Gnb2	0.270802	8.321622	2.290803	0.037152	0.20357	-4.44298	8.151556317	8.048240239	8.35981779	8.39680495	8.417157378	8.556156745
ENSMUSG00000020901.13	Pik3r5	0.270841	8.172584	2.217281	0.042763	0.212429	-4.57687	8.030162215	7.860214379	8.22199317	8.224903872	8.270156291	8.428076159
ENSMUSG00000097715.1	Gpr137b-ps	0.270936	5.193339	2.306746	0.03603	0.202184	-4.42431	5.118248552	4.979519345	5.07351821	5.30619611	5.179223644	5.50332657
ENSMUSG00000041836.9	Ptpre	0.27106	5.290848	2.355353	0.032803	0.194686	-4.34451	5.092135712	5.039869001	5.32638298	5.343336845	5.436824164	5.506541491
ENSMUSG00000041362.16	Shtn1	0.271458	6.080546	2.23078	0.041677	0.21176	-4.59479	5.911051003	5.74422993	6.17403623	6.149684087	6.193017965	6.311256111
ENSMUSG00000054342.8	Kcnn4	0.271557	7.358573	2.329797	0.034464	0.199257	-4.40125	7.219723767	7.057890409	7.39175654	7.449017544	7.439090771	7.593957106
ENSMUSG00000024401.14	Tnf	0.271567	8.549859	2.135001	0.049974	0.222777	-4.70809	8.405032478	8.224642786	8.61309301	8.576417618	8.648047558	8.831917654
ENSMUSG00000036966.15	Spryd3	0.271599	5.526567	2.445101	0.02755	0.180155	-4.19553	5.345254649	5.277152412	5.54412904	5.591487014	5.709738361	5.691640286
ENSMUSG00000022365.5	Derl1	0.271659	5.855445	2.20141	0.044074	0.214361	-4.64254	5.769933106	5.500521277	5.88166257	5.888744866	5.9377224	6.154086004
ENSMUSG00000043279.9	Trim56	0.27169	6.455485	2.154533	0.048166	0.220397	-4.72746	6.274450694	6.122107262	6.55933981	6.507767055	6.530028082	6.739219329
ENSMUSG00000078763.2	Slfn1	0.271751	8.210287	2.310074	0.0358	0.201625	-4.41199	8.030735167	7.942993469	8.25038711	8.279358744	8.318561282	8.439685326
ENSMUSG00000040774.15	Cept1	0.271901	7.600759	2.269081	0.038734	0.206831	-4.5025	7.421073575	7.304805149	7.67040767	7.717883451	7.661591037	7.828790684
ENSMUSG00000033352.11	Map2k4	0.271914	6.938711	2.49886	0.024797	0.172857	-4.10415	6.776097015	6.704953443	6.92672617	7.017613692	7.041810512	7.165062946
ENSMUSG00000034361.9	Cpne2	0.27195	6.017816	2.154468	0.048172	0.220397	-4.72684	5.813203679	5.66979539	6.15674788	6.138349725	6.105667037	6.223132438
ENSMUSG00000032667.17	Pon2	0.271976	5.44841	2.182026	0.045725	0.217275	-4.66081	5.292718986	5.158442796	5.47884989	5.459790066	5.513393298	5.787263212
ENSMUSG00000049401.10	Ogfr	0.272124	8.534464	2.286395	0.037468	0.204486	-4.44453	8.366516525	8.263699946	8.56573976	8.610214958	8.616573328	8.784037833
ENSMUSG00000009772.15	Nuak2	0.272449	5.466038	2.383467	0.031063	0.189975	-4.30457	5.348934995	5.168307416	5.46487018	5.569953852	5.534274775	5.709884727
ENSMUSG00000025178.8	Pi4k2a	0.272532	8.878528	2.253162	0.039932	0.208688	-4.49301	8.69582415	8.604313027	8.92787314	8.969199202	8.958500043	9.115456019
ENSMUSG00000062081.6	Gm6055	0.27255	4.90607	2.237674	0.041132	0.210668	-4.51866	4.562108887	4.807141078	4.93435546	5.060708406	4.974996146	5.097112921
ENSMUSG00000073491.10	Ifi213	0.272633	7.893598	2.156749	0.047965	0.220331	-4.69016	7.765464958	7.558399451	7.94874089	7.948604537	7.947485369	8.192893914
ENSMUSG00000033450.7	Tagap	0.27265	5.50921	2.207623	0.043556	0.213843	-4.61933	5.352605977	5.211882055	5.54678594	5.520919565	5.591546759	5.831517139
ENSMUSG00000068245.14	Phf11d	0.273007	8.192794	2.230055	0.041734	0.211893	-4.55392	7.993011708	7.911037175	8.26578898	8.25182485	8.291219957	8.443883787
ENSMUSG00000079523.8	Tmsb10	0.273064	8.435611	2.278974	0.038006	0.205148	-4.46059	8.259618778	8.193583775	8.444534	8.548859003	8.443738754	8.723331875
ENSMUSG00000028228.5	Cpne3	0.273139	6.294137	2.356943	0.032702	0.194499	-4.37123	6.163883756	5.964029059	6.34252842	6.442120184	6.37450282	6.477755557
ENSMUSG00000045658.16	Pid1	0.27318	6.748774	2.506043	0.02445	0.171594	-4.09455	6.5660669	6.513155265	6.7569924	6.859953446	6.840093792	6.956380249
ENSMUSG00000028098.13	Rnf115	0.273205	6.429249	2.196773	0.044463	0.214943	-4.65442	6.293674097	6.093918718	6.48648138	6.408815496	6.586557084	6.70604452
ENSMUSG00000021486.6	Prelid1	0.273254	7.070837	2.33879	0.033871	0.197603	-4.3925	6.933897496	6.779250496	7.08955086	7.168511695	7.112310311	7.341500491
ENSMUSG00000020089.8	Ppa1	0.27329	6.364018	2.575394	0.021327	0.161052	-3.97009	6.136463288	6.20351165	6.34099852	6.477246145	6.455355825	6.570532198
ENSMUSG00000027010.16	Slc25a12	0.273313	6.371138	2.193308	0.044757	0.215501	-4.66078	6.235217956	6.014118007	6.45146279	6.507767055	6.391248108	6.627012337
ENSMUSG00000020577.15	Tspan13	0.273367	5.624001	2.391935	0.030557	0.188589	-4.29657	5.420636461	5.361259679	5.67375393	5.701198386	5.749244173	5.837912074
ENSMUSG00000028859.14	Csf3r	0.273459	8.259419	2.2141	0.043023	0.212719	-4.57986	8.098981578	7.970033674	8.29926276	8.308906754	8.319108328	8.560223404
ENSMUSG00000022663.3	Atg3	0.273513	6.333322	2.441532	0.027742	0.180541	-4.21761	6.251039307	6.030435184	6.30535548	6.384350933	6.468237117	6.560513442
ENSMUSG00000040859.14	Bsdc1	0.273599	5.088705	2.190728	0.044977	0.216014	-4.61935	5.056566698	4.768752249	5.0247992	5.053830228	5.247487856	5.380791637
ENSMUSG00000071074.9	Yipf3	0.273607	5.789158	2.405619	0.029754	0.186663	-4.27723	5.673433758	5.480865727	5.79715627	5.907942688	5.855637366	6.019909908
ENSMUSG00000029560.12	Snx8	0.273608	6.479061	2.298954	0.036574	0.202683	-4.47407	6.344333094	6.144767859	6.53546017	6.560885972	6.568265935	6.720652454
ENSMUSG00000004266.15	Ptpn6	0.273658	8.93633	2.21788	0.042714	0.212429	-4.55313	8.720183081	8.669578362	9.00983273	9.004630847	9.041396171	9.172355866
ENSMUSG00000036469.16	Mar-01	0.273765	7.323707	2.27755	0.03811	0.205544	-4.49523	7.165449917	7.003825252	7.39249525	7.409825904	7.415751429	7.55489569
ENSMUSG00000025076.9	Casp7	0.273881	5.97955	2.483264	0.025567	0.175411	-4.13918	5.903562949	5.687052713	5.93334055	6.046052877	6.101857101	6.205436468
ENSMUSG00000046031.6	Fam26f	0.273937	9.294712	2.171476	0.046648	0.217969	-4.62294	9.097204775	8.998823973	9.37867436	9.36529237	9.402842326	9.525434403
ENSMUSG00000005125.12	Ndrg1	0.273944	6.71484	2.225929	0.042064	0.211556	-4.59998	6.650504003	6.336319572	6.74547381	6.784057877	6.804593755	6.968088124
ENSMUSG00000027514.14	Zbp1	0.273948	9.017776	2.172652	0.046544	0.217853	-4.62926	8.833869463	8.711503955	9.09851984	9.094511114	9.10495344	9.26330106
ENSMUSG00000029313.18	Aff1	0.273976	7.484713	2.298036	0.036639	0.202683	-4.45444	7.318756847	7.216049116	7.50811448	7.532407781	7.547173865	7.785775471
ENSMUSG00000068566.12	Myadm	0.274192	7.398665	2.232352	0.041552	0.211556	-4.57288	7.285535427	7.035159803	7.46592337	7.489723239	7.507405512	7.608243193
ENSMUSG00000073555.1	Gm4951	0.274295	6.972518	2.346563	0.033366	0.196286	-4.38081	6.809869509	6.689623599	7.00609689	7.038668903	7.047103598	7.243747915
ENSMUSG00000021877.11	Arf4	0.274381	8.101365	2.355457	0.032797	0.194686	-4.33394	7.966904237	7.817756405	8.10878256	8.195909829	8.172730788	8.346106334
ENSMUSG00000066152.11	Slc31a2	0.274418	8.540126	2.206881	0.043617	0.213843	-4.58414	8.395277011	8.210390897	8.60482995	8.630293128	8.619907242	8.780058211
ENSMUSG00000005667.8	Mthfd2	0.274642	5.737642	2.285016	0.037567	0.204554	-4.49229	5.534902517	5.448848989	5.8104838	5.855510583	5.776587038	5.999518119
ENSMUSG00000028656.14	Cap1	0.274645	9.106763	2.173707	0.046451	0.217853	-4.62476	8.955030137	8.788674012	9.16574207	9.176746335	9.181556946	9.37283139
ENSMUSG00000020198.7	Ap3d1	0.274717	7.031132	2.298378	0.036615	0.202683	-4.46566	6.812537461	6.748346038	7.12109136	7.085791992	7.215817315	7.203209132
ENSMUSG00000002820.5	Atg4d	0.274771	4.499727	2.243317	0.040691	0.209754	-4.45298	4.436289725	4.288503337	4.35849517	4.677901766	4.49898064	4.738194081
ENSMUSG00000024816.12	Frmd8	0.274814	6.303401	2.337993	0.033923	0.197603	-4.40525	6.086635534	6.073066394	6.33486265	6.335559495	6.381852811	6.608430228
ENSMUSG00000029490.3	Mfsd7a	0.274976	5.37753	2.516307	0.023961	0.170562	-4.05655	5.253986591	5.148510261	5.31395927	5.422897223	5.509563979	5.616263885
ENSMUSG00000037405.7	Icam1	0.275083	9.693128	2.149461	0.04863	0.220972	-4.64869	9.532982763	9.379553156	9.75519867	9.752346131	9.782698162	9.955976096
ENSMUSG00000014867.9	Surf4	0.275159	7.35181	2.324796	0.034798	0.200154	-4.41037	7.16753549	7.084181706	7.39101746	7.409825904	7.425945395	7.632356682
ENSMUSG00000057596.13	Trim30d	0.275285	7.56648	2.355582	0.032789	0.194686	-4.34919	7.413185186	7.278860755	7.59539688	7.657345794	7.637230162	7.816862188
ENSMUSG00000030339.7	Ltbr	0.275386	6.057605	2.517328	0.023913	0.170415	-4.07722	5.993305685	5.780054898	5.98323124	6.157725887	6.132058536	6.299256659
ENSMUSG00000013736.16	Trnt1	0.275396	4.641328	2.261233	0.03932	0.208097	-4.4427	4.394164249	4.409882576	4.69187089	4.75201418	4.83206712	4.767969332
ENSMUSG00000025854.15	Fam20c	0.275636	7.943622	2.302931	0.036296	0.202683	-4.43251	7.872896424	7.609074027	7.93612984	7.99736735	8.062377306	8.183885643
ENSMUSG00000027641.13	Rbl1	0.275647	6.341258	2.532114	0.023228	0.167585	-4.05072	6.215194108	6.059878962	6.33332459	6.455229725	6.448371811	6.535551348
ENSMUSG00000021453.2	Gadd45g	0.275729	5.748115	2.257657	0.03959	0.208339	-4.54101	5.631743075	5.378358487	5.81269314	5.817437255	5.866157064	5.982301983
ENSMUSG00000021895.9	Arhgef3	0.275803	5.716853	2.237375	0.041155	0.21069	-4.57574	5.550968329	5.399450896	5.77919252	5.720871805	5.846559024	6.004074601
ENSMUSG00000016529.5	Il10	0.275825	6.138418	2.460365	0.02674	0.17813	-4.18286	6.053186701	5.839913278	6.1054073	6.279174456	6.178620889	6.374206984
ENSMUSG00000059323.13	Tonsl	0.275844	5.663052	2.571335	0.021499	0.161349	-3.96796	5.525176446	5.403632623	5.64180197	5.819466362	5.760565829	5.827666527
ENSMUSG00000035678.7	Tnfsf9	0.275865	5.02524	2.492766	0.025095	0.173779	-4.07218	4.919738225	4.794457963	4.94244914	5.15371059	5.092288008	5.248796091
ENSMUSG00000034731.9	Dgkh	0.275967	4.852429	2.18691	0.045304	0.216675	-4.60071	4.618082525	4.632868821	4.884816	4.90507925	4.903842918	5.169886744
ENSMUSG00000027366.12	Sppl2a	0.27598	8.71646	2.221279	0.042438	0.212176	-4.55376	8.53631544	8.422044188	8.77792099	8.779504938	8.799016129	8.983960792
ENSMUSG00000025825.12	Iscu	0.276055	4.629072	2.217975	0.042706	0.212429	-4.51663	4.477219989	4.306481078	4.67253801	4.704495976	4.772598385	4.841098943
ENSMUSG00000001348.14	Acp5	0.276271	5.62016	2.307369	0.035987	0.202139	-4.44838	5.385231125	5.391050901	5.66399771	5.740280551	5.629091128	5.911306354
ENSMUSG00000035004.2	Igsf6	0.276281	6.429905	2.23945	0.040993	0.210441	-4.5797	6.165971593	6.162149938	6.54479372	6.506508159	6.51864207	6.681364384
ENSMUSG00000018567.14	Gabarap	0.276311	8.720819	2.3281	0.034577	0.199481	-4.36477	8.580030795	8.43946021	8.7290455	8.792733626	8.809955309	8.973688853

ENSMUSG00000022564.6	Grina	0.276319	9.009129	2.239571	0.040983	0.210441	-4.51298	8.857315198	8.707281224	9.049013	9.064883772	9.107172362	9.269109783
ENSMUSG00000018899.16	Irf1	0.276368	10.04334	2.137512	0.049738	0.222657	-4.65828	9.865448069	9.735236638	10.1159009	10.10520649	10.1386204	10.29963316
ENSMUSG00000040007.8	Bahd1	0.276395	6.845567	2.483598	0.025551	0.175411	-4.13418	6.666814192	6.592226836	6.86312888	6.969998637	6.915104097	7.06612659
ENSMUSG00000029152.13	Ociad1	0.276457	7.50276	2.369215	0.031934	0.19275	-4.32643	7.310286989	7.245468876	7.53866683	7.591783834	7.567115002	7.763441253
ENSMUSG00000008036.11	Ap2s1	0.276562	7.024911	2.4164	0.029136	0.185133	-4.25335	6.894147895	6.718444553	7.04795844	7.137943274	7.109150662	7.241820145
ENSMUSG00000027882.18	Stxbp3	0.276738	7.440192	2.421811	0.028831	0.184162	-4.23284	7.291285133	7.165231962	7.44968935	7.540426201	7.508365229	7.686157101
ENSMUSG00000032725.9	Folr2	0.276924	8.08307	2.244926	0.040566	0.209514	-4.53104	7.95849725	7.766724568	8.10878256	8.120562428	8.165144028	8.378709746
ENSMUSG00000033220.6	Rac2	0.276998	8.068857	2.187629	0.045242	0.21659	-4.63157	7.914470081	7.746289452	8.13064199	8.091050783	8.16453536	8.366154916
ENSMUSG00000026773.19	Pfkfb3	0.277065	9.054545	2.249718	0.040196	0.208938	-4.4938	8.876247634	8.760420583	9.11248624	9.117988668	9.171746763	9.288382825
ENSMUSG00000020400.17	Tnip1	0.277087	8.560276	2.246915	0.040412	0.209379	-4.51346	8.353293248	8.271157233	8.6425784	8.661734977	8.64521447	8.787676201
ENSMUSG00000033917.14	Gde1	0.277133	5.658873	2.184073	0.045548	0.216939	-4.66673	5.534902517	5.290762844	5.72626476	5.769962319	5.679382465	5.951965183
ENSMUSG00000040829.14	Zmynd15	0.277138	5.488893	2.142278	0.049293	0.222208	-4.73132	5.33415694	5.087434684	5.61673279	5.560279415	5.67596982	5.658785415
ENSMUSG00000022684.14	Bfar	0.27737	6.940068	2.233773	0.041439	0.211469	-4.58189	6.837639931	6.571932837	6.9944661	7.019380077	7.010315222	7.206672393
ENSMUSG00000042726.14	Trafd1	0.277463	9.142802	2.177248	0.046141	0.217853	-4.61755	8.963757355	8.826153841	9.22396584	9.232633381	9.214421241	9.395877429
ENSMUSG00000069793.11	Slfn9	0.277547	5.97791	2.391281	0.030595	0.188589	-4.30712	5.903562949	5.627515586	5.98126839	6.061565877	6.098037077	6.195510766
ENSMUSG00000021624.9	Cd180	0.277629	7.963379	2.29491	0.03686	0.203131	-4.4462	7.773703068	7.696882995	8.00343977	8.031465781	8.018927344	8.255857872
ENSMUSG00000078153.3	Psme2b	0.277655	8.859457	2.193112	0.044774	0.215501	-4.59857	8.683124288	8.555348611	8.9243018	8.927946738	8.921512676	9.144508924
ENSMUSG00000071715.11	Ncf4	0.277722	7.25792	2.367138	0.032063	0.19298	-4.33681	7.077236836	6.976013402	7.30496317	7.374378743	7.310193038	7.504733979
ENSMUSG00000056629.15	Fkbp2	0.277733	4.500581	2.201616	0.044056	0.214355	-4.52534	4.252022405	4.270298741	4.5454581	4.682368317	4.548340811	4.704995415
ENSMUSG00000034640.9	Tiparp	0.277774	7.413934	2.360939	0.03245	0.19391	-4.3438	7.316878949	7.099729536	7.40865184	7.470482745	7.505964738	7.681897695
ENSMUSG00000034116.17	Vav1	0.278187	8.785246	2.175444	0.046299	0.217853	-4.63141	8.631554228	8.452637913	8.85551429	8.855634181	8.854033899	9.062103665
ENSMUSG00000038034.15	Igsf8	0.278222	7.935432	2.164816	0.047239	0.219117	-4.675	7.792744334	7.56027369	8.03831456	8.010304405	8.05648407	8.15447033
ENSMUSG00000028599.10	Tnfrsf1b	0.278303	10.60267	2.148949	0.048677	0.220972	-4.62088	10.45705987	10.2844698	10.6500496	10.66445381	10.70553412	10.85446659
ENSMUSG00000078616.1	Trim30c	0.278383	7.381048	2.431171	0.028309	0.182362	-4.21741	7.242655823	7.085483778	7.39839133	7.474351427	7.479781686	7.605622152
ENSMUSG00000030751.17	Psma1	0.278481	6.041871	2.258553	0.039522	0.208294	-4.54537	5.865528364	5.683617723	6.15326521	6.139974381	6.174999058	6.23384075
ENSMUSG00000015316.11	Slamf1	0.278509	8.163	2.270011	0.038665	0.206683	-4.48447	7.991835198	7.869314194	8.21114735	8.259692071	8.201498742	8.444512503
ENSMUSG00000021929.8	Kpna3	0.278531	7.954257	2.382252	0.031137	0.190179	-4.28993	7.78529378	7.674561769	7.98641519	8.059206604	8.030664816	8.189397378
ENSMUSG00000034947.13	Tmem106a	0.278551	8.517586	2.264731	0.039058	0.207653	-4.48334	8.360148004	8.208001834	8.56770328	8.577916678	8.615014861	8.776733452
ENSMUSG00000024121.13	Atp6v0c	0.278599	7.160701	2.259902	0.039421	0.208097	-4.53069	7.068334717	6.787274404	7.20926189	7.203116015	7.324431176	7.371785061
ENSMUSG00000066149.11	Cdc26	0.278609	4.355997	2.225238	0.042119	0.212176	-4.45995	4.244138805	4.084832889	4.30927265	4.497031258	4.403164853	4.597541912
ENSMUSG00000022534.13	Mefv	0.27873	7.072059	2.488323	0.025315	0.174569	-4.12054	6.867454097	6.843765603	7.08681503	7.165324298	7.153381824	7.315615749
ENSMUSG00000021591.7	Glrx	0.278787	6.532268	2.248133	0.040318	0.209217	-4.56346	6.299391522	6.224938208	6.65232586	6.59089319	6.667832288	6.758224761
ENSMUSG00000071379.1	Hpcal1	0.278809	6.813182	2.136561	0.049827	0.222753	-4.75331	6.671230609	6.441749597	6.90721612	6.871734621	6.861282456	7.125880683
ENSMUSG00000029404.15	Arl6ip4	0.278824	4.325391	2.172866	0.046525	0.217853	-4.54476	4.220226186	3.976715272	4.33409382	4.527140769	4.455853392	4.438318191
ENSMUSG00000025357.7	Dgka	0.278885	6.037243	2.222084	0.042373	0.212176	-4.60945	5.802507096	5.737619632	6.14802539	6.148070334	6.07230844	6.31492826
ENSMUSG00000038722.17	Bud31	0.278901	5.123592	2.271823	0.038531	0.206554	-4.48	4.953973273	4.807141078	5.18003853	5.144028037	5.294978972	5.3613908
ENSMUSG00000033467.12	Crlf2	0.278943	7.373118	2.284058	0.037637	0.204554	-4.48236	7.276867674	7.010695241	7.41448239	7.431877717	7.504041465	7.600741837
ENSMUSG00000031021.13	Tmem9b	0.279026	5.32923	2.250243	0.040156	0.208938	-4.53387	5.234223337	4.933986728	5.38694571	5.500829492	5.400065614	5.519330007
ENSMUSG00000055413.12	H2-Q5	0.279545	5.785781	2.155531	0.048076	0.220331	-4.72028	5.753366091	5.356953124	5.81930091	5.873200526	5.846559024	6.065309277
ENSMUSG00000020476.14	Dbnl	0.279597	8.569375	2.280596	0.037887	0.205058	-4.45376	8.398832091	8.267146561	8.62414215	8.66371282	8.640626158	8.821790616
ENSMUSG00000029919.5	Hpgds	0.279661	5.868895	2.477501	0.025858	0.176099	-4.1476	5.719650164	5.587639567	5.87533495	6.032122099	5.903113363	6.095512594
ENSMUSG00000004567.15	Mcoln1	0.279839	5.514711	2.643976	0.018618	0.152281	-3.82549	5.392382044	5.26800693	5.45924014	5.683482798	5.667402722	5.6177512
ENSMUSG00000055994.15	Nod2	0.279958	4.437307	2.201859	0.044036	0.214334	-4.5148	4.195910525	4.288503337	4.40609355	4.517173924	4.483446761	4.732713751
ENSMUSG00000004730.14	Adgre1	0.279986	9.991221	2.184361	0.045523	0.216939	-4.57926	9.839684973	9.676399116	10.0383736	10.04989278	10.08261475	10.26035815
ENSMUSG00000021038.16	Vipas39	0.280125	7.537642	2.25337	0.039916	0.208688	-4.53203	7.390151187	7.201711656	7.60179891	7.598298478	7.617351931	7.816538423
ENSMUSG00000035199.6	Arl6ip5	0.280146	6.305028	2.324087	0.034846	0.200218	-4.4301	6.163883756	5.969681153	6.35773884	6.384350933	6.375555114	6.5789556
ENSMUSG00000006589.7	Aprt	0.280178	5.825317	2.396789	0.03027	0.187973	-4.29401	5.756140505	5.460938683	5.83242636	5.964049371	5.916189238	6.022157972
ENSMUSG00000058587.7	Tmod3	0.280355	7.077965	2.222737	0.042321	0.212176	-4.59807	6.90918263	6.731810671	7.17274676	7.143623184	7.159504028	7.350921107
ENSMUSG00000042608.15	Stk40	0.280419	6.881454	2.439469	0.027854	0.180919	-4.21435	6.77199067	6.556992173	6.89475664	6.984539309	6.978809592	7.101637566
ENSMUSG00000038866.15	Zcchc2	0.280427	6.707487	2.457597	0.026885	0.178511	-4.18433	6.481417534	6.475909711	6.74315902	6.769437607	6.816266108	6.958729437
ENSMUSG00000050029.7	Rap2c	0.280455	7.280895	2.266899	0.038896	0.207284	-4.51521	7.153925059	6.918727661	7.35053388	7.350020072	7.394625874	7.517538457
ENSMUSG00000055720.9	Ubl7	0.280473	5.815183	2.4677	0.026359	0.17712	-4.16438	5.664600904	5.531423557	5.82368934	5.841600193	5.990876654	6.038907716
ENSMUSG00000057948.12	Unc13d	0.280476	5.093356	2.272013	0.038516	0.206554	-4.47689	4.944274446	4.735962314	5.1628224	5.24712153	5.164687551	5.30526886
ENSMUSG00000031557.15	Plekha2	0.280511	6.633204	2.342306	0.033641	0.196741	-4.39469	6.378944862	6.368765356	6.73036083	6.737600455	6.727567554	6.855983647
ENSMUSG00000016534.15	Lamp2	0.280536	9.273399	2.219871	0.042552	0.212347	-4.53952	9.093918765	8.973026636	9.33322841	9.332987292	9.360951944	9.546281397
ENSMUSG00000026786.14	Apbb1ip	0.280625	6.172065	2.166835	0.047059	0.219041	-4.70668	6.032738712	5.780054898	6.27683339	6.211252628	6.287730633	6.443778974
ENSMUSG00000029826.14	Zc3hav1	0.280657	7.790408	2.185012	0.045467	0.216816	-4.64429	7.708592242	7.404154488	7.83867356	7.840351647	7.886046365	8.064627828
ENSMUSG00000048234.13	Rnf149	0.280923	8.817689	2.18892	0.045131	0.216249	-4.60709	8.692931165	8.449101894	8.89201673	8.920640147	8.903751744	9.047693064
ENSMUSG00000024052.17	Lpin2	0.281092	5.916214	2.494993	0.024986	0.17357	-4.11637	5.823821538	5.587639567	5.91082996	6.002064865	6.096761484	6.076168907
ENSMUSG00000039232.11	Stx11	0.281101	7.602576	2.302492	0.036326	0.202683	-4.44317	7.442762123	7.285673967	7.65878776	7.689874407	7.663746244	7.874609423
ENSMUSG00000034422.14	Parp14	0.281103	9.820367	2.202008	0.044023	0.214334	-4.55395	9.646304014	9.518346776	9.87592934	9.90847895	9.881470119	10.09167124
ENSMUSG00000026005.15	Rpe	0.281205	5.599644	2.28025	0.037913	0.205058	-4.49561	5.403042401	5.29976565	5.66644296	5.687932131	5.637888076	5.902791265
ENSMUSG00000030595.15	Nfkbib	0.281226	7.55303	2.252731	0.039965	0.208688	-4.53272	7.376693113	7.221981259	7.63962572	7.591783834	7.685547384	7.80254736
ENSMUSG00000000386.14	Mx1	0.28136	9.743191	2.180396	0.045867	0.21765	-4.59382	9.579932929	9.417786354	9.81096738	9.803088942	9.844612996	10.00275897
ENSMUSG00000046822.12	Slc39a3	0.281378	4.665233	2.188716	0.045149	0.216249	-4.5725	4.630231608	4.26110957	4.66277353	4.781457958	4.75976008	4.896065402
ENSMUSG00000032932.14	Hspa13	0.281501	4.392203	2.203263	0.043919	0.214299	-4.50458	4.321100787	4.13599765	4.29037245	4.546870239	4.390727749	4.668146583
ENSMUSG00000022587.14	Ly6e	0.281683	10.00113	2.210358	0.04333	0.213218	-4.53381	9.837394331	9.693791542	10.0508274	10.08605539	10.07478058	10.26392454
ENSMUSG00000022842.16	Ece2	0.28177	4.446805	2.389651	0.030692	0.188787	-4.18363	4.343410949	4.195092397	4.37054279	4.561492274	4.588827258	4.621462792
ENSMUSG00000026626.11	Ppp2r5a	0.281787	7.208562	2.303917	0.036227	0.202683	-4.45141	7.011416288	6.898127892	7.29472566	7.318085036	7.27001483	7.459001597
ENSMUSG00000027007.16	Ssfa2	0.28182	6.801525	2.507045	0.024401	0.171439	-4.09173	6.635514829	6.522798985	6.82314152	6.900773408	6.900557011	7.026363674
ENSMUSG00000009927.8	Rps25	0.28186	6.426149	2.233426	0.041467	0.211469	-4.59027	6.325777274	6.312053487	6.21637186	6.589704809	6.30440295	6.808583409
ENSMUSG00000031805.17	Jak3	0.281867	5.983343	2.374102	0.031633	0.191705	-4.33821	5.947919151	5.616749155	5.95750349	6.087055851	6.057959637	6.232870545
ENSMUSG00000069874.6	Irgm2	0.281948	8.326936	2.203153	0.043928	0.214299	-4.59694	8.155239348	7.99726832	8.40627643	8.371788471	8.417668292	8.613377843
ENSMUSG00000043716.13	Rpl7	0.281983	7.859869	2.281119	0.037849	0.205029	-4.47369	7.808199529	7.616298806	7.7318209	8.031028071	7.779968745	8.191895769
ENSMUSG00000037731.5	Themis2	0.282046	9.804483	2.196182	0.044513	0.214943	-4.56456	9.653204198	9.473798237	9.86479339	9.881175778	9.896851398	10.05707631
ENSMUSG00000019487.10	Trip10	0.282234	5.825177	2.205315	0.043748	0.214017	-4.63485	5.619606767	5.46894256	5.9555053	5.890676184	5.943410707	6.072919592
ENSMUSG00000000594.7	Gm2a	0.282248	7.081019	2.396747	0.030272	0.187973	-4.28769	6.924062298	6.774414642	7.12109136	7.133056919	7.202833267	7.330658203
ENSMUSG00000064193.8	Gm4735	0.282259	5.636404	2.38929	0.030714	0.188787	-4.30174	5.601208706	5.480865727	5.40462963	5.807248688	5.587919617	5.936554161
ENSMUSG00000058927.5	Gm10053	0.282326	5.702437	2.361954	0.032387	0.193723	-4.35336	5.515384361	5.391050901	5.77012606	5.767862326	5.815878547	5.954321566
ENSMUSG00000000555.6	Itga5	0.282389	8.467137	2.351737	0.033033	0.195344	-4.3299	8.307689027	8.179020169	8.49183397	8.547023318	8.543082106	8.734171495
ENSMUSG00000041238.15	Rbbp8	0.282487	5.348361	2.241713	0.040816	0.210011	-4.55013	5.092135712	5.055899111	5.46205791	5.459790066	5.414472104	5.605809528
ENSMUSG00000031792.14	Usb1	0.28258	7.11011	2.410018	0.029501	0.186203	-4.2629	6.891626802	6.843765603	7.17188648	7.224712495	7.181331307	7.347339551
ENSMUSG00000031216.13	Stard8	0.282631	5.412402	2.378099	0.031389	0.190935	-4.31114	5.242161156	5.182979018	5.3839772	5.420225565	5.498014657	5.747055453
ENSMUSG00000036381.13	P2ry14	0.282898	6.77419	2.36753	0.032038	0.192964	-4.34701	6.569225511	6.489743879	6.83840251	6.861923668	6.837805011	7.048037852
ENSMUSG00000000530.16	Acvrl1	0.283032	6.010037	2.574514	0.021364	0.161151	-3.97025	5.842216806	5.737619632	6.02193766	6.102136425	6.146925426	6.209387706
ENSMUSG00000006699.17	Cdc42	0.28323	9.055076	2.318222	0.035242	0.200644	-4.37247	8.904344771	8.749783906	9.08738542	9.140887447	9.14422671	9.303825905
ENSMUSG00000032724.5	Abtb2	0.283236	6.59576	2.620719	0.019497	0.154813	-3.8831	6.481417534	6.331937807	6.5474494	6.670613996	6.715151857	6.827987804
ENSMUSG00000040952.16	Rps19	0.283325	6.13231	2.311651	0.035691	0.20128	-4.4519	5.955181055	5.888462803	6.125097	6.292383353	6.063193911	6.46954207
ENSMUSG00000042167.5	Papd4	0.283385	5.664218	2.248116	0.04032	0.209217	-4.5548	5.482255579	5.317604456	5.75871228	5.843595622	5.674260466	5.908878595
ENSMUSG00000032661.9	Oas3	0.283395	9.554061	2.153	0.048306	0.220594	-4.64688	9.41965247	9.190107946	9.62908679	9.63296365	9.638162118	9.814391645
ENSMUSG00000031246.14	Sh3bgrl	0.283489	8.526045	2.36225	0.032368	0.193723	-4.30928	8.382764787	8.226414413	8.54462758	8.613726947	8.602710828	8.786023535
ENSMUSG00000013465.18	Nelfb	0.283672	5.534953	2.508848	0.024315	0.171189	-4.07851	5.388811015	5.244886322	5.53880052	5.649668085	5.614903478	5.772650154
ENSMUSG00000000204.15	Slfn4	0.283695	8.383949	2.330688	0.034405	0.199086	-4.37007	8.251776693	8.068793687	8.40627643	8.437338403	8.485519512	8.653991352
ENSMUSG00000032688.7	Malt1	0.283845	7.824976	2.224428	0.042184	0.212176	-4.57457	7.665707972	7.475413171	7.90954034	7.900054517	7.895247258	8.103894578
ENSMUSG00000000791.8	Il12rb1	0.284104	5.940854	2.197448	0.044406	0.214943	-4.65104	5.649758231	5.662834242	6.07811718	6.098798783	5.956127984	6.199489245
ENSMUSG00000099764.1	Rps10-ps2	0.284363	7.053029	2.226482	0.04202	0.212176	-4.59209	6.99271767	6.698160281	7.04137936	7.167715506	7.02715359	7.391047547
ENSMUSG00000052712.16	BC004004	0.284368	5.109511	2.659191	0.018064	0.150179	-3.76964	4.914780505	4.939757516	5.04373248	5.25013342	5.221973292	5.286689144
ENSMUSG00000004040.16	Stat3	0.284383	9.418834	2.284369	0.037614	0.204554	-4.42171	9.228865479	9.133954061	9.46829024	9.518033159	9.483873748	9.679985751
ENSMUSG00000025877.14	Hk3	0.284473	10.76554	2.139586	0.049544	0.222531	-4.63165	10.58457426	10.44257543	10.8442528	10.84038287	10.85810981	11.0233333
ENSMUSG00000027367.16	Stard7	0.284485	6.050932	2.381568	0.031178	0.190335	-4.32549	6.002681478	5.687052713	6.03145415	6.151296037	6.114517955	6.318591087
ENSMUSG00000034118.15	Tpst1	0.284646	5.440371	2.31154	0.035699	0.20128	-4.43212	5.185656786	5.143518232	5.5547274	5.533335261	5.644887217	5.580100445
ENSMUSG00000029561.17	Oasl2	0.284673	10.05485	2.175341	0.046308	0.217853	-4.59286	9.871537766	9.73233028	10.1348972	10.11277563	10.16548629	10.3120889
ENSMUSG00000020300.14	Cpeb4	0.284692	7.552962	2.509028	0.024307	0.171189	-4.06976	7.372179045	7.295833834	7.56426537	7.626386715	7.668047022	7.79105826
ENSMUSG00000026875.14	Traf1	0.284693	8.688425	2.314699	0.035482	0.20128	-4.38965	8.496220951	8.390260888	8.75311515	8.769306399	8.792333789	8.929310614
ENSMUSG00000038497.6	Tmco3	0.284714	6.449575	2.284857	0.037579	0.204554	-4.49925	6.380743734	6.059878962	6.47815411	6.538887654	6.512915188	6.726868
ENSMUSG00000030654.9	Arl6ip1	0.284816	7.581407	2.477166	0.025875	0.176099	-4.12765	7.420199214	7.311497214	7.58574025	7.669208271	7.656405367	7.84539008
ENSMUSG00000052270.7	Fpr2	0.284879	6.311024	2.262598	0.039218	0.208097	-4.53939	6.229239929	5.966857874	6.30535548	6.362250817	6.333936025	6.668505418
ENSMUSG00000024066.8	Xdh	0.285049	8.14584	2.239406	0.040996	0.210441	-4.53889	7.940315597	7.841070056	8.22945433	8.209899708	8.211256708	8.443045071
ENSMUSG00000025525.12	Apool	0.285217	5.052315	2.228813	0.041833	0.212074	-4.54894	4.833048491	4.781662358	5.1027014	5.194933253	5.034833735	5.366707864
ENSMUSG00000020078.15	Vps26a	0.285282	6.756314	2.368211	0.031996	0.192814	-4.34607	6.580226507	6.419207028	6.84165182	6.876615191	6.856768152	6.963416369
ENSMUSG00000025017.9	Pik3ap1	0.285286	9.250903	2.22439	0.042188	0.212176	-4.5323	9.117304777	8.911587857	9.29660141	9.298124433	9.349349508	9.532449782
ENSMUSG00000026628.13	Atf3	0.2853	9.440278	2.171499	0.046646	0.217969	-4.61847	9.290687273	9.095050434	9.50802928	9.488846972	9.527840881	9.731211592
ENSMUSG00000001750.15	Tcirg1	0.285378	9.077346	2.216006	0.042867	0.212526	-4.55217	8.936498739	8.724098942	9.14506961	9.162829235	9.15728773	9.338290182
ENSMUSG00000003283.13	Hck	0.285388	9.076296	2.199406	0.044242	0.214663	-4.58112	8.906531619	8.730354768	9.16574207	9.15762564	9.165676402	9.331848055
ENSMUSG00000043998.10	Mgat2	0.285927	4.79269	2.618296	0.019591	0.155099	-3.81305	4.666076807	4.639975707	4.64304406	4.946517204	4.895063814	4.965462124
ENSMUSG00000004535.12	Tax1bp1	0.285965	9.826527	2.186046	0.045378	0.216675	-4.58145	9.675541957	9.486420971	9.88972948	9.886145578	9.917860567	10.10346336

ENSMUSG00000040033.15	Stat2	0.285972	9.246141	2.261249	0.039319	0.208097	-4.46775	9.040582953	8.944967429	9.32551293	9.337950189	9.335761922	9.492068276
ENSMUSG00000024014.7	Pim1	0.286067	8.179975	2.140635	0.049446	0.222432	-4.70941	8.0432829	7.774010922	8.29610803	8.254826947	8.295674012	8.415945446
ENSMUSG00000021814.16	Anxa7	0.28607	8.25539	2.253824	0.039882	0.208688	-4.51031	8.069171628	7.920552702	8.34920272	8.341043352	8.345931036	8.506441133
ENSMUSG00000034974.12	Dapk3	0.286081	5.011476	2.421345	0.028857	0.184162	-4.20107	4.806527614	4.762253612	5.02860579	5.043451082	5.179223644	5.248796091
ENSMUSG00000031779.3	Ccl22	0.286084	4.743028	2.173982	0.046427	0.217853	-4.60865	4.463705183	4.46694575	4.85081867	4.885843031	4.788487257	5.002367606
ENSMUSG00000026979.16	Psd4	0.286164	7.442579	2.275865	0.038233	0.205878	-4.49494	7.273966812	7.107441064	7.51762605	7.482057806	7.543901387	7.730481407
ENSMUSG00000070003.6	Ssbp4	0.286185	6.181208	2.555556	0.022179	0.164159	-4.00698	6.073348916	5.852204684	6.18601597	6.321310539	6.317603454	6.336767013
ENSMUSG00000025198.16	Erlin1	0.28619	5.467839	2.541384	0.022807	0.166062	-4.01496	5.399497694	5.221389141	5.35091399	5.472740736	5.629091128	5.733399932
ENSMUSG00000025792.9	Slc25a10	0.286249	4.980655	2.176906	0.046171	0.217853	-4.632	4.874493209	4.654085307	4.97437731	5.025984827	5.002503081	5.352485247
ENSMUSG00000028779.16	Pef1	0.286353	5.477842	2.59543	0.020499	0.158008	-3.91471	5.269604552	5.29527127	5.43649768	5.503356104	5.718058425	5.644263953
ENSMUSG00000031712.8	Il15	0.286556	7.634591	2.366063	0.03213	0.193081	-4.32837	7.510052433	7.312609546	7.65201742	7.723853981	7.684274043	7.924736696
ENSMUSG00000037020.16	Wdr62	0.286652	5.598639	2.154753	0.048146	0.220397	-4.71491	5.550968329	5.178105043	5.62681279	5.707786046	5.602373789	5.92578773
ENSMUSG00000096472.1	Cdkn2d	0.286863	5.975421	2.393933	0.030438	0.18841	-4.30213	5.813203679	5.641746972	6.03524324	6.035617425	6.096761484	6.229956009
ENSMUSG00000037049.8	Smpd1	0.286908	4.06259	2.164021	0.047311	0.219371	-4.5086	3.956387826	3.719283573	4.05683947	4.224329665	4.213759471	4.204941801
ENSMUSG00000044786.5	Zfp36	0.286929	9.081715	2.26307	0.039182	0.208097	-4.46947	8.93435688	8.747318112	9.13406922	9.138654487	9.187261382	9.348627696
ENSMUSG00000078920.2	Ifi47	0.287158	9.658995	2.182981	0.045642	0.217113	-4.59193	9.47606352	9.321339322	9.75030842	9.720261054	9.767847491	9.918150325
ENSMUSG00000021451.15	Sema4d	0.287288	8.047957	2.273401	0.038414	0.206263	-4.48185	7.895721344	7.698585792	8.11997671	8.109410707	8.149234601	8.314813647
ENSMUSG00000043257.15	Pigv	0.28731	4.141782	2.142259	0.049295	0.222208	-4.56206	3.887204261	3.895903227	4.19196874	4.29603113	4.213759471	4.365823046
ENSMUSG00000021054.5	Sgpp1	0.287321	7.454374	2.402703	0.029923	0.187287	-4.26717	7.277833333	7.168921721	7.4854431	7.513105143	7.531681059	7.749262318
ENSMUSG00000025279.7	Dnase1l3	0.287381	5.545926	2.137585	0.049731	0.222657	-4.74204	5.475537546	5.108081835	5.61166625	5.562704113	5.665683181	5.851881941
ENSMUSG00000036067.12	Slc2a6	0.287617	9.705335	2.27061	0.03862	0.206554	-4.43732	9.53863367	9.393024042	9.75419321	9.798338584	9.778527516	9.969290112
ENSMUSG00000035208.15	Slfn8	0.287642	8.969276	2.287161	0.037413	0.204415	-4.43024	8.820677509	8.643295047	9.01295857	9.002176524	9.102889953	9.233658886
ENSMUSG00000046229.9	Scand1	0.287804	5.168513	2.255979	0.039718	0.208663	-4.51145	5.139653916	4.735962314	5.18345724	5.231967031	5.372922939	5.347115408
ENSMUSG00000004056.15	Akt2	0.287863	6.930685	2.537367	0.022989	0.167019	-4.03299	6.864885881	6.633759099	6.86099551	7.045619571	6.978116988	7.200730273
ENSMUSG00000057411.8	Fam173a	0.2879	4.474305	2.444	0.027609	0.180155	-4.0896	4.372631088	4.251861493	4.36453156	4.541963109	4.635254619	4.679585256
ENSMUSG00000032175.10	Tyk2	0.287909	6.93876	2.573959	0.021388	0.161176	-3.96472	6.78699047	6.663706225	6.93384809	7.067980716	7.000799178	7.17923442
ENSMUSG00000030745.9	Il21r	0.287968	4.866553	2.300858	0.036441	0.202683	-4.40178	4.599664891	4.632868821	4.92212916	5.032996725	4.889181257	5.122479641
ENSMUSG00000020346.16	Mgat1	0.288167	7.494326	2.406557	0.0297	0.186632	-4.25907	7.407902196	7.130331373	7.51423625	7.581657527	7.648156411	7.683673976
ENSMUSG00000046879.6	Irgm1	0.288196	9.99713	2.168475	0.046914	0.21874	-4.60651	9.859171274	9.639421741	10.061666	10.0566822	10.08832219	10.27751463
ENSMUSG00000014226.10	Cacybp	0.288263	6.579705	2.491843	0.025141	0.173779	-4.12314	6.433692614	6.264562372	6.60721839	6.702848123	6.647067503	6.822838764
ENSMUSG00000026672.11	Optn	0.288411	5.727558	2.380959	0.031215	0.190335	-4.31979	5.591920938	5.348301253	5.80161248	5.845588295	5.897263603	5.880661721
ENSMUSG00000028530.14	Jak1	0.288484	8.366473	2.243076	0.04071	0.209754	-4.52593	8.248332331	7.989638665	8.43057579	8.454740261	8.445493553	8.630055512
ENSMUSG00000002205.15	Vrk3	0.288502	5.061627	2.259483	0.039452	0.208101	-4.49589	4.80116443	4.807141078	5.13484198	5.046919101	5.245186907	5.334507506
ENSMUSG00000051439.6	Cd14	0.288534	8.341677	2.353467	0.032923	0.19504	-4.33047	8.167274958	8.018555496	8.40810402	8.419724078	8.475984635	8.560416769
ENSMUSG00000051007.14	Gatd1	0.288556	4.440912	2.302245	0.036344	0.202683	-4.33874	4.450062575	4.155966066	4.27763333	4.609189016	4.48734594	4.665272688
ENSMUSG00000036986.16	Pml	0.288683	7.969883	2.373096	0.031695	0.191895	-4.306	7.779169122	7.669361301	8.0293184	8.056628444	8.053528399	8.231292569
ENSMUSG00000032812.16	Arap1	0.28871	8.978817	2.260392	0.039384	0.208097	-4.47727	8.828936606	8.629078999	9.04713769	9.066806796	9.068978549	9.231960385
ENSMUSG00000061731.9	Ext1	0.289161	5.580798	2.247521	0.040365	0.20925	-4.5524	5.378044584	5.202311904	5.7168584	5.753076156	5.660512225	5.773984747
ENSMUSG00000003541.6	Ier3	0.289209	6.146935	2.44277	0.027675	0.18028	-4.21494	5.906063288	5.885475855	6.2113567	6.180007334	6.30440295	6.394305422
ENSMUSG00000005354.15	Txn2	0.289245	5.385493	2.266332	0.038938	0.207346	-4.50896	5.222234112	5.001757859	5.48440406	5.596228826	5.426707047	5.581625493
ENSMUSG00000022175.8	Lrp10	0.28925	7.872268	2.244761	0.040579	0.209514	-4.53751	7.768216225	7.487283201	7.92952826	7.98927871	7.912025091	8.147279312
ENSMUSG00000026111.15	Unc50	0.289281	5.633789	2.611986	0.019838	0.155892	-3.89065	5.554160134	5.335225378	5.57308922	5.7055935	5.770199889	5.864465493
ENSMUSG00000000957.9	Mmp14	0.289565	9.156793	2.242956	0.040719	0.209754	-4.5026	9.032881716	8.799425584	9.20453742	9.190922367	9.287940231	9.42505303
ENSMUSG00000067212.8	H2-T23	0.289779	10.72556	2.206487	0.04365	0.213852	-4.51755	10.56897899	10.39203781	10.782613	10.81600883	10.81396344	10.97975597
ENSMUSG00000028955.3	Vamp3	0.289842	6.906737	2.370129	0.031878	0.192604	-4.33971	6.697448908	6.631977993	6.95500531	6.983634798	6.940213795	7.232142463
ENSMUSG00000020638.7	Cmpk2	0.289851	10.82467	2.216562	0.042821	0.212429	-4.49682	10.65770878	10.5111582	10.8714654	10.90648185	10.90445819	11.096763
ENSMUSG00000060036.13	Rpl3	0.290272	5.220676	2.195723	0.044552	0.214964	-4.6214	5.07446082	5.061203143	5.0881836	5.523411266	5.063846862	5.512949919
ENSMUSG00000062082.16	Cd200r4	0.290282	6.668599	2.454401	0.027054	0.179098	-4.19076	6.449205613	6.394206633	6.72567865	6.76101616	6.739877313	6.941610514
ENSMUSG00000018848.4	Rars	0.290287	7.114909	2.511534	0.024187	0.170977	-4.07666	6.92282817	6.842225907	7.14496002	7.246744601	7.166208579	7.36648671
ENSMUSG00000032316.7	Clk3	0.290485	5.068191	2.36069	0.032466	0.19391	-4.31613	4.757525714	4.844534836	5.155878	5.166520397	5.205498618	5.279189737
ENSMUSG00000004843.6	Chmp2b	0.290519	5.816132	2.156006	0.048033	0.220331	-4.72005	5.775413435	5.348301253	5.87955644	5.902210117	5.89579745	6.095512594
ENSMUSG00000078921.3	Tgtp2	0.290579	8.587473	2.381158	0.031203	0.190335	-4.27339	8.403706065	8.3022876	8.62130902	8.677482317	8.649570765	8.870482429
ENSMUSG00000042677.7	Zc3h12a	0.290628	6.830655	2.251519	0.040058	0.208688	-4.55287	6.662384214	6.441749597	6.95200178	6.923590807	6.938078577	7.06612659
ENSMUSG00000033880.11	Lgals3bp	0.290634	10.68536	2.22514	0.042127	0.212176	-4.48635	10.52197074	10.36132687	10.7380461	10.75906829	10.7870298	10.94474324
ENSMUSG00000045302.13	Preb	0.290644	5.220145	2.68377	0.017201	0.146277	-3.73201	5.042998933	5.007264336	5.16973352	5.300396323	5.426707047	5.373766936
ENSMUSG00000023206.16	Il15ra	0.290721	7.091265	2.407559	0.029642	0.186632	-4.2678	6.87129787	6.795253933	7.17188648	7.192195319	7.175905369	7.341050352
ENSMUSG00000048779.4	P2ry6	0.290735	7.152756	2.459956	0.026761	0.17813	-4.17069	6.988004902	6.829848823	7.20506312	7.21857499	7.317877184	7.357167516
ENSMUSG00000035392.17	Dennd1a	0.290857	7.743734	2.594815	0.020524	0.158008	-3.90491	7.582963699	7.469441331	7.7440275	7.871979042	7.850159368	7.943835637
ENSMUSG00000071014.10	Ndufb6	0.290899	4.094271	2.175807	0.046267	0.217853	-4.49515	3.793082083	3.931092655	4.11528574	4.168113655	4.24633784	4.311715641
ENSMUSG00000035929.11	H2-Q4	0.290924	8.689711	2.256791	0.039656	0.208602	-4.49223	8.555156126	8.317881445	8.76143124	8.756389803	8.808397638	8.939010193
ENSMUSG00000063889.16	Crem	0.291048	5.671779	2.397892	0.030205	0.187925	-4.28714	5.444913152	5.369834415	5.75641865	5.738136873	5.795580555	5.92578773
ENSMUSG00000028037.13	Ifi44	0.291227	8.373763	2.411679	0.029405	0.185875	-4.22439	8.221481467	8.067476457	8.39599895	8.429388738	8.486980839	8.641250633
ENSMUSG00000024235.10	Map3k8	0.291284	5.675092	2.411522	0.029414	0.185875	-4.26253	5.505525358	5.416105569	5.66154832	5.696789841	5.739468676	6.031115362
ENSMUSG00000018965.11	Ywhah	0.291316	7.351094	2.397844	0.030208	0.187925	-4.2788	7.19744232	7.043223327	7.37615567	7.456208155	7.375292113	7.658243489
ENSMUSG00000015340.10	Cybb	0.29138	10.42354	2.236757	0.041204	0.210715	-4.47441	10.2334147	10.11646567	10.4847072	10.50118506	10.50935427	10.69611308
ENSMUSG00000030830.18	Itgal	0.291429	8.624773	2.289785	0.037225	0.203719	-4.43583	8.426090891	8.316773171	8.6950181	8.68139244	8.70921676	8.92014823
ENSMUSG00000015027.10	Galns	0.291614	5.659133	2.392424	0.030527	0.188589	-4.29657	5.508819184	5.281703505	5.74026029	5.78665312	5.789277126	5.848085373
ENSMUSG00000032802.8	Srxn1	0.291644	6.154529	2.502714	0.02461	0.171946	-4.1048	6.030448719	5.796049464	6.19620573	6.298215378	6.303297436	6.302959439
ENSMUSG00000091649.3	Phf11b	0.291675	8.77345	2.282507	0.037749	0.204879	-4.44432	8.571393052	8.46269333	8.84960875	8.830701477	8.866250645	9.0600538
ENSMUSG00000002602.16	Axl	0.291758	8.690894	2.312003	0.035667	0.20128	-4.39437	8.474906123	8.395519487	8.76542881	8.747625245	8.794892457	8.966994538
ENSMUSG00000027078.14	Ube2l6	0.291878	8.119071	2.320747	0.035071	0.200218	-4.39561	7.954275313	7.782866778	8.18324381	8.174270964	8.221241813	8.398529691
ENSMUSG00000029401.8	Rilpl2	0.291917	7.113895	2.374455	0.031611	0.191705	-4.32722	7.027581108	6.726812877	7.15021097	7.194542438	7.243170561	7.341050352
ENSMUSG00000058818.13	Pirb	0.291933	9.358335	2.282177	0.037773	0.204879	-4.4274	9.19986434	9.028574842	9.40947319	9.415118971	9.458152409	9.63882628
ENSMUSG00000024414.13	Mrpl27	0.291986	5.037979	2.171444	0.046651	0.217969	-4.647	4.811870936	4.654085307	5.19027045	5.101302738	5.219631254	5.250714569
ENSMUSG00000051185.9	Fam174a	0.292105	5.660892	2.529298	0.023357	0.16819	-4.04574	5.403042401	5.428471602	5.70739031	5.819466362	5.721373061	5.885608816
ENSMUSG00000032180.10	Tmed1	0.292152	4.929364	2.312596	0.035627	0.20128	-4.38814	4.822498571	4.632868821	4.88900988	4.90507925	5.066455714	5.260268875
ENSMUSG00000044811.13	Cd300c2	0.292183	6.811918	2.280414	0.037901	0.205058	-4.50212	6.562901359	6.503446648	6.93080015	6.91981288	6.854505692	7.100042252
ENSMUSG00000024259.8	Slc25a46	0.292284	4.862619	2.449959	0.02729	0.17995	-4.13274	4.746406622	4.639975707	4.76214591	4.897415513	4.949783358	5.179989691
ENSMUSG00000017561.16	Crlf3	0.292301	6.467273	2.46561	0.026467	0.177559	-4.17254	6.253004832	6.184194943	6.52337048	6.50146156	6.612673999	6.728933912
ENSMUSG00000037321.17	Tap1	0.292306	9.848675	2.232524	0.041538	0.211556	-4.49984	9.665246797	9.516658567	9.92717232	9.932113751	9.933396742	10.11746157
ENSMUSG00000002603.15	Tgfb1	0.292671	7.256167	2.316845	0.035336	0.201006	-4.42708	7.091586637	6.883231579	7.3566036	7.362598747	7.358891662	7.484089001
ENSMUSG00000052406.14	Rexo4	0.29302	5.209267	2.488881	0.025287	0.174552	-4.09484	5.006181751	4.910669937	5.25993133	5.340513566	5.312667408	5.425636934
ENSMUSG00000023947.7	Nfkbie	0.29303	7.855433	2.372575	0.031727	0.191948	-4.31027	7.671598034	7.523279483	7.9341018	7.975241077	7.936475087	8.091905353
ENSMUSG00000066861.14	Oas1g	0.293057	7.794786	2.446055	0.027498	0.180155	-4.17858	7.610053726	7.511703131	7.82341546	7.869532966	7.867466649	8.086544659
ENSMUSG00000009585.17	Apobec3	0.293109	7.807896	2.309995	0.035805	0.201625	-4.42387	7.618463737	7.465446327	7.90177833	7.885600948	7.904389842	8.07169921
ENSMUSG00000022808.4	Snx4	0.293117	5.945011	2.242291	0.040771	0.209905	-4.57245	5.964807079	5.500521277	5.92315198	6.003850379	6.048753712	6.228983187
ENSMUSG00000003458.12	Ncstn	0.293259	7.153923	2.528893	0.023375	0.16819	-4.04358	6.936345855	6.88023377	7.20590385	7.236150412	7.291359355	7.373546862
ENSMUSG00000073131.12	Vma21	0.293434	6.10368	2.613731	0.019769	0.155682	-3.89772	6.009673552	5.855261242	6.00271425	6.168909625	6.162860198	6.422661866
ENSMUSG00000032434.8	Cmtm6	0.293475	7.61514	2.41216	0.029378	0.185875	-4.24549	7.476799562	7.273158396	7.6563295	7.685987236	7.723647465	7.874920416
ENSMUSG00000021996.16	Esd	0.293499	8.741562	2.433166	0.028199	0.182013	-4.17452	8.618654305	8.430266159	8.73604677	8.819339626	8.830437227	9.01462724
ENSMUSG00000048120.16	Entpd1	0.293546	6.43511	2.595735	0.020486	0.158008	-3.93148	6.213176353	6.201111159	6.44862424	6.531479705	6.50812536	6.708140446
ENSMUSG00000019794.13	Katna1	0.293658	6.456866	2.396523	0.030285	0.187973	-4.29869	6.245126614	6.164616084	6.51796459	6.571760609	6.476107276	6.765623213
ENSMUSG00000032381.5	Fam96a	0.29385	5.019079	2.343231	0.033581	0.196627	-4.3425	4.914780505	4.639975707	5.0474895	5.074367159	5.154914812	5.282944313
ENSMUSG00000023460.12	Rab12	0.294059	6.761416	2.702838	0.01656	0.142907	-3.72546	6.608135503	6.50149706	6.73269624	6.864873966	6.859779257	7.001513351
ENSMUSG00000023903.7	Mmp25	0.294064	5.764857	2.493436	0.025062	0.173779	-4.11545	5.750586331	5.436657128	5.66154832	5.902210117	5.789277126	6.048865006
ENSMUSG00000028337.14	Coro2a	0.294185	7.026134	2.481664	0.025648	0.175594	-4.13384	6.831076248	6.730146662	7.07581952	7.105050541	7.119865354	7.294847228
ENSMUSG00000056116.18	H2-T22	0.294391	8.756793	2.384675	0.03099	0.189631	-4.26204	8.594054672	8.440478164	8.795342	8.843470988	8.839998499	9.027413187
ENSMUSG00000061207.11	Stk19	0.294394	4.963899	2.422348	0.0288	0.184122	-4.19389	4.869377264	4.611335659	4.95450516	5.13753671	5.010653922	5.199985785
ENSMUSG00000044533.15	Rps2	0.294398	7.525194	2.573865	0.021392	0.161176	-3.95017	7.409665343	7.302567545	7.42246112	7.735721422	7.490020525	7.790728652
ENSMUSG00000024164.15	C3	0.294666	10.23093	2.258079	0.039558	0.208334	-4.44308	10.0338109	9.912596896	10.3058947	10.31866795	10.32609905	10.48849068
ENSMUSG00000040811.15	Eml2	0.294717	5.075014	2.331973	0.03432	0.19885	-4.36826	4.977938858	4.781662358	5.01715577	5.13428006	5.094845978	5.444198179
ENSMUSG00000098050.1	Gm5345	0.294726	3.933839	2.200884	0.044118	0.21442	-4.418	3.83567396	3.650570823	3.86579117	4.0136314	4.08072068	4.156646244
ENSMUSG00000038352.15	Arl5c	0.295274	6.188469	2.532086	0.023229	0.167585	-4.0505	5.964807079	5.906255773	6.24773604	6.244858478	6.36075269	6.406403565
ENSMUSG00000029009.17	Mthfr	0.295336	8.469228	2.412767	0.029343	0.185875	-4.21962	8.289610501	8.164308046	8.51168867	8.548859003	8.559379972	8.741523477
ENSMUSG00000030530.15	Furin	0.295344	9.0516	2.305999	0.036082	0.202334	-4.39434	8.894934834	8.703046097	9.11517386	9.12107663	9.159580391	9.31578757
ENSMUSG00000017715.11	Pgs1	0.295489	7.18529	2.298762	0.036588	0.202683	-4.46116	7.002097273	6.80160593	7.31122726	7.283958298	7.350353402	7.362500139
ENSMUSG00000062270.13	Morf4l1	0.295611	4.774826	2.262053	0.039259	0.208097	-4.45877	4.671965366	4.418173949	4.77579927	4.897415513	4.788487257	5.097112921
ENSMUSG00000059456.13	Ptk2b	0.295685	8.692448	2.326399	0.034691	0.199707	-4.36864	8.511489891	8.360990896	8.76257454	8.764311641	8.787598138	8.967723687
ENSMUSG00000036534.5	Slc38a7	0.295836	5.640099	2.764753	0.014633	0.133032	-3.6014	5.557344892	5.382601711	5.53345225	5.709975265	5.800290119	5.85692854
ENSMUSG00000024772.9	Ehd1	0.296045	9.294648	2.315744	0.035411	0.20128	-4.36971	9.1167652	8.964559885	9.35991221	9.376361474	9.387797228	9.562493806
ENSMUSG00000079227.9	Ccr5	0.296068	8.684579	2.254911	0.039799	0.208663	-4.49569	8.518451533	8.317327414	8.77565771	8.765627728	8.7696997	8.960708594
ENSMUSG00000031539.5	Ap3m2	0.29621	5.046741	2.546046	0.022599	0.165893	-3.97516	4.934509976	4.781662358	4.97437731	5.121179448	5.125193005	5.343524379
ENSMUSG00000000278.10	Scpep1	0.2963	8.53066	2.337471	0.033957	0.197603	-4.35361	8.349164706	8.192375741	8.60737748	8.592822225	8.65586412	8.78635422
ENSMUSG00000053581.13	Zfand2a	0.296362	6.352773	2.63611	0.018911	0.152876	-3.85608	6.256927867	6.062526109	6.29116492	6.397993541	6.472177563	6.635847446
ENSMUSG00000037894.13	H2afz	0.296436	4.803299	2.390424	0.030646	0.188644	-4.23321	4.677829988	4.544737911	4.72977639	5.097963165	4.769399503	5.000088467
ENSMUSG00000017830.15	Dhx58	0.296513	8.142326	2.432271	0.028249	0.182193	-4.19367	7.96630536	7.827127123	8.19005803	8.235200708	8.228539962	8.406726231

ENSMUSG00000020826.6	Nos2	0.296515	12.17331	2.166695	0.047072	0.219041	-4.53887	11.98222181	11.85728742	12.2372498	12.26028739	12.26231079	12.44049317
ENSMUSG00000020023.17	Tmcc3	0.29664	6.064903	2.309633	0.03583	0.201681	-4.45471	5.918500391	5.662834242	6.16195617	6.123644891	6.196594897	6.325888959
ENSMUSG00000102037.1	Bcl2a1a	0.296819	5.44308	2.58931	0.020748	0.158889	-3.92413	5.273482767	5.207104915	5.39875904	5.548094418	5.478557922	5.752481675
ENSMUSG00000036810.16	Cnep1r1	0.296988	5.155001	2.514773	0.024034	0.170639	-4.04253	5.052058273	4.807141078	5.14890002	5.259131548	5.268032911	5.39473925
ENSMUSG00000019578.11	Ubxn6	0.297112	5.513819	2.642437	0.018675	0.152281	-3.82795	5.395944256	5.197502916	5.49544867	5.591487014	5.708068575	5.694462184
ENSMUSG00000056145.5	AI504432	0.297252	5.232008	2.248868	0.040262	0.209164	-4.52894	5.14812792	4.844534836	5.24691934	5.204281202	5.383422949	5.56476064
ENSMUSG00000090272.7	Mndal	0.297258	8.898714	2.396209	0.030304	0.188001	-4.237	8.732914386	8.582807184	8.93549638	8.982163757	8.983389717	9.175515238
ENSMUSG00000039853.18	Trim14	0.297267	5.703848	2.462826	0.026611	0.177749	-4.1697	5.502223995	5.374102747	5.78145026	5.867327947	5.768598673	5.929385475
ENSMUSG00000046722.14	Cdc42se1	0.297457	6.830342	2.324929	0.034789	0.200154	-4.42245	6.68002309	6.443781562	6.92059356	6.9179202	6.914380216	7.105353118
ENSMUSG00000022971.18	Ifnar2	0.297618	6.850307	2.432166	0.028254	0.182193	-4.22825	6.731681539	6.473922527	6.89892177	6.965424458	6.934512844	7.09737947
ENSMUSG00000064120.13	Mocs1	0.297639	5.673097	2.536135	0.023044	0.167189	-4.03337	5.531667773	5.348301253	5.68585715	5.82956931	5.711406217	5.931778997
ENSMUSG00000050957.4	Insl6	0.297738	4.71011	2.420729	0.028891	0.184266	-4.16621	4.636267997	4.35046909	4.68223683	4.814388233	4.825921445	4.951375486
ENSMUSG00000070031.11	Sp140	0.297843	7.550546	2.37564	0.031539	0.191501	-4.31348	7.299866953	7.243137247	7.66369177	7.628124205	7.700320575	7.768136766
ENSMUSG00000002108.10	Nr1h3	0.297868	6.230075	2.450759	0.027247	0.179934	-4.2005	6.023556851	5.906255773	6.31005481	6.366420458	6.286612265	6.487550412
ENSMUSG00000060938.14	Rpl26	0.297876	7.089708	2.301859	0.036371	0.202683	-4.45797	6.980906757	6.892187811	6.94698196	7.222413989	6.97742405	7.518334976
ENSMUSG00000026107.11	Nabp1	0.297895	4.994394	2.251616	0.040051	0.208688	-4.50256	4.929602845	4.574717068	5.01715577	5.011857957	5.142605053	5.290424279
ENSMUSG00000021981.9	Cab39l	0.297988	5.238299	2.728297	0.01574	0.139139	-3.64874	5.087737248	5.029082446	5.14890002	5.274004824	5.440851229	5.449219165
ENSMUSG00000029298.15	Gbp9	0.298196	8.712318	2.376899	0.031462	0.191207	-4.27739	8.50861354	8.406500546	8.77537456	8.772975714	8.822163546	8.988279164
ENSMUSG00000000708.14	Kat2b	0.298331	5.329274	2.215467	0.042911	0.212526	-4.59438	5.169097694	4.898868685	5.45641686	5.404090876	5.448871792	5.598295474
ENSMUSG00000053559.12	Smagp	0.298471	4.928162	2.391713	0.03057	0.188589	-4.24553	4.671965366	4.647047756	5.00174633	5.107958782	4.98053965	5.159712547
ENSMUSG00000040253.15	Gbp7	0.298522	7.902042	2.317056	0.035322	0.201006	-4.40854	7.771647946	7.517502918	7.97067207	7.962902463	8.013191645	8.176335542
ENSMUSG00000052423.14	B4galt3	0.298652	4.008368	2.301454	0.036399	0.202683	-4.25805	3.803848514	3.810294511	3.95650474	4.096290474	4.218458808	4.164808614
ENSMUSG00000029502.14	Golga3	0.298723	6.824449	2.542738	0.022747	0.166062	-4.0251	6.635514829	6.507337939	6.88218862	6.898855568	7.001480981	7.021315358
ENSMUSG00000024677.13	Ms4a6b	0.298742	8.202165	2.552073	0.022332	0.164957	-3.97142	8.015763941	7.927829868	8.21532847	8.280095658	8.314451818	8.459520085
ENSMUSG00000046223.10	Plaur	0.298815	7.642022	2.404411	0.029824	0.186992	-4.25879	7.476799562	7.294708486	7.70768713	7.720057413	7.742938411	7.909941242
ENSMUSG00000025791.18	Pgm2	0.298928	6.730964	2.559349	0.022014	0.163601	-3.99581	6.637020773	6.370902682	6.73619227	6.858967325	6.833982287	6.94871886
ENSMUSG00000073409.12	H2-Q6	0.299028	8.689261	2.437801	0.027945	0.181144	-4.16761	8.526192643	8.359915261	8.73488225	8.794539898	8.793121555	8.92691299
ENSMUSG00000026766.16	Mmadhc	0.299112	5.336451	2.745093	0.01522	0.136501	-3.62342	5.122555108	5.128437658	5.30457076	5.488129629	5.42264025	5.552370269
ENSMUSG00000069792.5	Wfdc17	0.299125	7.493294	2.447271	0.027433	0.180155	-4.1849	7.328109846	7.16646293	7.53713131	7.552675861	7.593583026	7.781800646
ENSMUSG00000032504.15	Pdcd6ip	0.299276	7.80895	2.572531	0.021448	0.161239	-3.94465	7.623790148	7.516537905	7.83921552	7.930882507	7.908394297	8.034877644
ENSMUSG00000026177.11	Slc11a1	0.299503	7.556923	2.475832	0.025942	0.176177	-4.13078	7.445342973	7.206506659	7.57080673	7.656778474	7.634595503	7.827505861
ENSMUSG00000053835.17	H2-T24	0.299955	7.444145	2.485162	0.025472	0.175276	-4.11674	7.245620266	7.145392268	7.49235091	7.545338553	7.507405512	7.728761866
ENSMUSG00000032487.8	Ptgs2	0.29998	10.34803	2.321129	0.035045	0.200218	-4.32777	10.1649933	10.03440153	10.3954924	10.41432205	10.45787299	10.62106889
ENSMUSG00000029484.12	Anxa3	0.300441	6.960094	2.299109	0.036563	0.202683	-4.46585	6.797802286	6.564481846	7.06751755	7.037797711	7.055006991	7.237956859
ENSMUSG00000028923.14	Necap2	0.300547	7.479185	2.28518	0.037556	0.204554	-4.4774	7.297964288	7.106158669	7.58380114	7.537963738	7.569415951	7.779809119
ENSMUSG00000020828.13	Pld2	0.30063	5.20005	2.232206	0.041563	0.211556	-4.55547	5.156552441	4.742580193	5.23378892	5.311972674	5.252078775	5.50332657
ENSMUSG00000039316.14	Rftn1	0.300682	7.271963	2.50383	0.024556	0.171946	-4.08693	7.099254738	6.933263754	7.33370926	7.365379177	7.407544075	7.492625683
ENSMUSG00000049502.16	Dtx3l	0.300733	7.796635	2.358378	0.032612	0.194379	-4.33754	7.554558613	7.495142642	7.88979488	7.842348802	7.907667041	8.090299236
ENSMUSG00000021807.5	Rtraf	0.30078	6.257212	2.226963	0.041981	0.212176	-4.60195	6.044134467	5.870447646	6.40096331	6.348264328	6.314314634	6.56514613
ENSMUSG00000070034.13	Sp110	0.300858	7.930458	2.463103	0.026597	0.177749	-4.14346	7.736957767	7.613593752	7.99129989	8.047568427	8.007433052	8.185892347
ENSMUSG00000038507.6	Parp12	0.300859	8.51058	2.342959	0.033599	0.196627	-4.34437	8.358779656	8.135054814	8.58912743	8.62855825	8.600237379	8.751719845
ENSMUSG00000069892.9	9930111J21Ri	0.300889	5.435153	2.365491	0.032165	0.193208	-4.33475	5.197952557	5.216643429	5.4336295	5.619706888	5.362345948	5.780639256
ENSMUSG00000027580.17	Helz2	0.300966	9.61998	2.334767	0.034135	0.198465	-4.32595	9.454232989	9.284965757	9.67048381	9.708538964	9.696694061	9.904962823
ENSMUSG00000037344.13	Slc12a9	0.300976	6.15435	2.559242	0.022018	0.163601	-3.99984	5.923445366	5.849141636	6.23625999	6.329876789	6.307714416	6.279659594
ENSMUSG00000025059.16	Gk	0.301063	6.99388	2.32446	0.034821	0.200198	-4.41987	6.868736493	6.588558188	7.0730575	7.081571114	7.075225924	7.276131933
ENSMUSG00000021298.7	Gpr132	0.301129	6.101906	2.614856	0.019725	0.155682	-3.89549	5.918500391	5.780054898	6.15152072	6.209706331	6.27199403	6.279659594
ENSMUSG00000054676.15	1600014C10Ri	0.301201	7.488589	2.445604	0.027523	0.180155	-4.18808	7.279762715	7.162766869	7.57341499	7.61006844	7.57400687	7.731512149
ENSMUSG00000011008.13	Mcoln2	0.301502	7.05898	2.297352	0.036687	0.202683	-4.46673	6.917881076	6.64792933	7.15978834	7.153308445	7.144766857	7.330204668
ENSMUSG00000029772.17	Ahcyl2	0.301544	5.353677	2.484938	0.025483	0.175276	-4.11215	5.177400998	5.045232186	5.37802179	5.351773682	5.554858322	5.614775036
ENSMUSG00000026810.12	Dpm2	0.301738	5.152612	2.183966	0.045557	0.216939	-4.63554	4.90980569	4.755725569	5.3201845	5.259131548	5.245186907	5.425636934
ENSMUSG00000022744.12	Cldnd1	0.301933	4.691926	2.627429	0.01924	0.154043	-3.78238	4.503875621	4.498567729	4.61799665	4.75201418	4.86840134	4.910699797
ENSMUSG00000025509.15	Pnpla2	0.302024	6.628251	2.411407	0.029421	0.185875	-4.26972	6.412745675	6.273729647	6.74431688	6.735452788	6.756128626	6.847134668
ENSMUSG00000022964.14	Tmem50b	0.302133	6.301573	2.463539	0.026574	0.177749	-4.17706	6.194888424	5.949801182	6.30221406	6.295302312	6.463296375	6.60393468
ENSMUSG00000024725.12	Ostf1	0.302176	7.022079	2.468814	0.026301	0.176911	-4.15753	6.776097015	6.721797709	7.11573316	7.125696214	7.12362809	7.269523798
ENSMUSG00000035673.9	Sbno2	0.302261	8.410277	2.483934	0.025534	0.175411	-4.09131	8.255212852	8.08190025	8.44203861	8.533489783	8.485275813	8.663743599
ENSMUSG00000042901.10	Aida	0.302332	7.402136	2.586103	0.02088	0.159111	-3.93068	7.201519172	7.168921721	7.38137473	7.457511697	7.470947329	7.732542155
ENSMUSG00000026980.15	Ly75	0.302353	3.832667	2.17317	0.046499	0.217853	-4.44373	3.546167343	3.79763921	3.71331514	3.940936172	3.916912213	4.081029198
ENSMUSG00000020709.15	Adap2	0.30252	7.152581	2.462849	0.02661	0.177749	-4.16536	6.983276687	6.800020551	7.22178536	7.262491429	7.24086271	7.407048825
ENSMUSG00000061360.7	Phf5a	0.302734	4.709014	2.614538	0.019738	0.155682	-3.80861	4.52354904	4.498567729	4.64304406	4.893568324	4.762980382	4.932376758
ENSMUSG00000034612.7	Chst11	0.302763	5.810569	2.388853	0.03074	0.188787	-4.30766	5.613500112	5.43257017	5.92315198	5.884874454	5.971520608	6.037797096
ENSMUSG00000032299.9	Commd4	0.302808	4.673634	2.292684	0.037018	0.203267	-4.39082	4.562108887	4.35046909	4.64304406	4.773106588	4.683636951	5.029440082
ENSMUSG00000073155.12	1810058I24Ril	0.302882	5.385234	2.573206	0.02142	0.161195	-3.95058	5.269604552	5.012749875	5.40755599	5.550539665	5.524820645	5.546134948
ENSMUSG00000015839.6	Nfe2l2	0.302891	7.644032	2.534436	0.023122	0.167352	-4.02016	7.441900812	7.337959309	7.69935299	7.744291382	7.754309466	7.886380269
ENSMUSG00000027502.11	Rtf2	0.302898	7.123038	2.688269	0.017048	0.145787	-3.74555	6.94001062	6.851439531	7.12376302	7.249757278	7.199271788	7.373986977
ENSMUSG00000039997.16	Ifi203	0.302984	8.853526	2.344758	0.033482	0.196453	-4.33101	8.616747491	8.563781338	8.92659868	8.95703901	8.891481986	9.165507918
ENSMUSG00000030990.17	Pgap2	0.303093	7.009995	2.539089	0.022911	0.166634	-4.02804	6.823160206	6.718444553	7.03287622	7.075641062	7.085551676	7.324295701
ENSMUSG00000024778.12	Fas	0.303148	6.423614	2.447917	0.027399	0.180155	-4.20518	6.245126614	6.057226948	6.51117862	6.537655635	6.517689167	6.672804488
ENSMUSG00000014077.13	Chp1	0.303183	6.741569	2.436608	0.02801	0.181318	-4.22204	6.525989461	6.410921607	6.83188184	6.819973193	6.839331268	7.021315358
ENSMUSG00000065990.12	Aurkaip1	0.303232	5.157705	2.321797	0.035	0.200218	-4.39325	5.083325333	4.702409754	5.21386646	5.314852306	5.247487856	5.384291202
ENSMUSG00000079197.10	Psme2	0.303265	7.424441	2.362177	0.032373	0.193723	-4.34125	7.10361828	7.176273049	7.53913413	7.482057806	7.548107498	7.697454416
ENSMUSG00000066258.13	Trim12a	0.303571	5.46238	2.191112	0.044944	0.216011	-4.64498	5.193865599	5.102947684	5.62178159	5.586729565	5.48831909	5.780639256
ENSMUSG00000010307.5	Tmem86a	0.303803	4.333297	2.431042	0.028316	0.182324	-4.08859	4.259863158	4.053239764	4.2255213	4.434858835	4.431774474	4.59452372
ENSMUSG00000078485.2	Plekhn1	0.3039	5.733685	2.461252	0.026694	0.177985	-4.17347	5.64377814	5.343955783	5.74951579	5.77624402	5.87660061	6.012013981
ENSMUSG00000030689.15	Ino80e	0.3041	4.364371	2.398561	0.030166	0.18787	-4.15245	4.220226186	4.042553086	4.35849517	4.466279933	4.495112823	4.603559418
ENSMUSG00000044340.7	Phlpp1	0.304279	6.860881	2.628248	0.019208	0.153884	-3.86456	6.701772673	6.532378668	6.89266956	7.010526444	6.953663944	7.074274363
ENSMUSG00000074141.12	Il4i1	0.30435	6.220016	2.48093	0.025684	0.175641	-4.145	5.998001198	5.894418208	6.30692362	6.311251867	6.318698063	6.490800642
ENSMUSG00000022553.15	Maf1	0.304378	6.174299	2.474438	0.026013	0.176388	-4.15681	6.106338807	5.770372345	6.18601597	6.376103012	6.217872227	6.389089285
ENSMUSG00000019804.12	Snx3	0.304447	6.697894	2.509703	0.024274	0.171081	-4.08844	6.445772667	6.425390017	6.76385966	6.723583129	6.856014393	6.9727448
ENSMUSG00000025371.12	Chmp6	0.304562	4.900972	2.38089	0.031219	0.190335	-4.26188	4.779510064	4.574717068	4.88060989	5.015402665	4.912568923	5.243025303
ENSMUSG00000035725.13	Prkx	0.304991	7.772356	2.628624	0.019194	0.153861	-3.84071	7.582182174	7.493181792	7.78511157	7.881236489	7.859215153	8.03320673
ENSMUSG00000027533.10	Fabp5	0.305076	8.950213	2.331127	0.034376	0.199003	-4.35254	8.814368821	8.642410636	8.93726936	9.123131605	8.887799056	9.296299186
ENSMUSG00000038683.15	Pak1ip1	0.305175	4.535482	2.202716	0.043964	0.214334	-4.52781	4.379844578	4.165847577	4.58735418	4.541963109	4.666543208	4.871339946
ENSMUSG00000024965.8	Fermt3	0.305178	7.921377	2.38769	0.03081	0.189038	-4.28104	7.800156609	7.53381015	7.97413059	8.014295879	8.006754057	8.199116729
ENSMUSG00000003421.12	Nosip	0.305366	4.995804	2.722417	0.015926	0.139769	-3.63849	4.929602845	4.688767146	4.90151888	5.160129711	5.159809457	5.134997681
ENSMUSG00000002210.11	Smg9	0.305482	5.336025	2.817387	0.013168	0.125327	-3.48489	5.14389714	5.118295648	5.28242369	5.525898672	5.404196455	5.541440708
ENSMUSG00000032691.14	Nlrp3	0.305515	7.479526	2.541603	0.022798	0.166062	-4.01145	7.340178794	7.130331373	7.51083848	7.549011872	7.626662533	7.720133281
ENSMUSG00000040701.15	Ap1g2	0.30573	4.79861	2.35368	0.03291	0.19504	-4.29826	4.549369775	4.490726976	4.87639148	5.025984827	4.819749478	5.029440082
ENSMUSG00000060550.15	H2-Q7	0.30588	8.353824	2.472074	0.026134	0.176671	-4.11472	8.203807348	8.003480892	8.39747166	8.485096912	8.429622992	8.603465587
ENSMUSG00000028191.11	Bcl10	0.30591	5.754041	2.661786	0.017971	0.149791	-3.80065	5.637773157	5.411959891	5.74720747	5.907942688	5.831300341	5.988063553
ENSMUSG00000024101.14	Washc1	0.305924	6.29403	2.804465	0.013514	0.126608	-3.53589	6.119326397	6.027728438	6.27362918	6.393914288	6.410883946	6.538695332
ENSMUSG00000032590.14	Apeh	0.305929	5.560936	2.521875	0.0237	0.169529	-4.05517	5.352605977	5.254179076	5.60912629	5.645098976	5.641391891	5.863212065
ENSMUSG00000029428.13	Stx2	0.305993	6.986219	2.508296	0.024342	0.171285	-4.08558	6.760982929	6.674129112	7.06566616	7.142813136	7.039156669	7.234567976
ENSMUSG00000021322.7	Aoah	0.306099	7.47025	2.460188	0.026749	0.17813	-4.16188	7.2700899	7.135369161	7.5471177	7.530551031	7.600349901	7.738023135
ENSMUSG00000006411.12	Nectin4	0.306157	5.596921	2.700173	0.016648	0.143489	-3.72242	5.502223995	5.26800693	5.5547274	5.763653147	5.663961588	5.828951206
ENSMUSG00000030707.15	Coro1a	0.306175	6.606697	2.396615	0.03028	0.187973	-4.29683	6.467942176	6.215454558	6.6755759	6.71706737	6.659216585	6.904924754
ENSMUSG00000022488.8	Nckap1l	0.306313	9.372821	2.378966	0.031336	0.190874	-4.25403	9.199354778	9.041043903	9.41947427	9.453515874	9.448184273	9.675351012
ENSMUSG00000040242.14	Fgfr1op2	0.306314	5.524906	2.638246	0.018831	0.152835	-3.83623	5.388811015	5.254179076	5.46767698	5.530860655	5.672549083	5.8353575
ENSMUSG00000034216.12	Vps18	0.306351	6.592668	2.368588	0.031973	0.192814	-4.34771	6.412745675	6.191469051	6.71390629	6.772583019	6.667832288	6.797470614
ENSMUSG00000099974.1	Bcl2a1d	0.306362	7.59301	2.430077	0.02837	0.182324	-4.21349	7.411426338	7.260533299	7.64892946	7.720600392	7.614234996	7.902333679
ENSMUSG00000031402.12	Mpp1	0.306552	8.522543	2.443894	0.027615	0.180155	-4.16137	8.379169844	8.167385468	8.56246128	8.598150744	8.633166385	8.794925526
ENSMUSG00000047843.16	Bri3	0.306598	7.042315	2.566394	0.02171	0.162568	-3.97651	6.875131428	6.740102044	7.05076889	7.095871999	7.128629855	7.363386996
ENSMUSG00000009035.13	Tmem184b	0.306637	8.024022	2.548152	0.022505	0.165568	-3.98384	7.831405143	7.716344865	8.06543457	8.107751245	8.139665985	8.283530084
ENSMUSG00000030042.15	Pole4	0.306666	4.512052	2.452664	0.027146	0.179354	-4.07897	4.415380735	4.214264707	4.44078929	4.532098489	4.693796817	4.775984325
ENSMUSG00000002812.4	Flii	0.306705	7.834346	2.477086	0.025879	0.176099	-4.12057	7.657569671	7.485311632	7.90177833	7.915787963	7.967164379	8.078466085
ENSMUSG00000028854.9	Slc9a1	0.306748	5.468048	2.416371	0.029138	0.185133	-4.24421	5.285055249	5.09262424	5.5547274	5.567541318	5.558569436	5.749771115
ENSMUSG00000059070.14	Rpl18	0.30683	6.216732	2.598123	0.02039	0.158008	-3.92736	6.117169902	5.983715262	6.08727136	6.414196183	6.153075031	6.544962819
ENSMUSG00000026727.10	Rsu1	0.306838	5.482191	2.313448	0.035568	0.20128	-4.43039	5.31922594	5.09262424	5.56262538	5.596228826	5.503800875	5.818641595
ENSMUSG00000002379.6	Ndufa11	0.307386	4.546662	2.59082	0.020687	0.158806	-3.82929	4.415380735	4.251861493	4.49682332	4.726289367	4.659648595	4.72996576
ENSMUSG00000026390.7	Marco	0.307409	5.746244	2.205605	0.043723	0.214017	-4.63169	5.554160134	5.29527127	5.91700413	5.803153029	5.970128046	5.937745487
ENSMUSG00000068706.2	Gm10250	0.307722	4.957715	2.226353	0.04203	0.212176	-4.54261	4.763053288	4.611335659	5.01715577	5.241078819	4.880312202	5.233355729
ENSMUSG00000032870.8	Smap2	0.307804	5.570333	2.605368	0.020099	0.157268	-3.90003	5.420636461	5.240217402	5.58088759	5.622033816	5.711406217	5.846817627
ENSMUSG00000025225.14	Nfkb2	0.307911	9.084931	2.436361	0.028024	0.181318	-4.15856	8.904344771	8.748962443	9.14133888	9.194640151	9.171898195	9.348403754
ENSMUSG00000034959.7	Rubcnl	0.308144	6.14625	2.673847	0.017545	0.147698	-3.78453	5.925911512	5.879483344	6.16714573	6.191020327	6.295535031	6.418401098
ENSMUSG00000025044.15	Msr1	0.308302	9.136333	2.36626	0.032117	0.193081	-4.28398	8.942600874	8.814422959	9.18973933	9.184048637	9.222338892	9.464849704
ENSMUSG00000041355.13	Ssr2	0.308546	5.420431	2.706229	0.016448	0.14248	-3.70222	5.242161156	5.108081835	5.43936017	5.557850635	5.545538554	5.629594838
ENSMUSG00000024696.8	Lpxn	0.308547	7.740021	2.419404	0.028966	0.184566	-4.22874	7.54976958	7.385247402	7.82451069	7.876371554	7.790658356	8.013568381

ENSMUSG00000038508.7	Gdf15	0.30864	4.904706	2.481334	0.025664	0.175618	-4.07924	4.763053288	4.552291305	4.92212916	4.975923471	5.026818742	5.188021417
ENSMUSG00000055371.17	Stam2	0.308683	6.469234	2.681946	0.017264	0.146531	-3.76874	6.268633367	6.176883972	6.49751016	6.610947328	6.536628659	6.724799125
ENSMUSG00000022876.17	Samsn1	0.309353	6.444518	2.20309	0.043933	0.214299	-4.64296	6.219221173	6.043893258	6.60211827	6.472095956	6.548807325	6.780971177
ENSMUSG00000025647.16	Shisa5	0.30942	8.767014	2.497895	0.024844	0.173005	-4.0552	8.586280465	8.452637913	8.79868964	8.844467755	8.862690284	9.057316107
ENSMUSG00000081219.1	Bambi-ps1	0.309606	4.41892	2.589113	0.020757	0.158889	-3.81238	4.275417972	4.175661867	4.33409382	4.551760736	4.525768851	4.650816705
ENSMUSG00000041153.9	Osgin2	0.309727	4.476209	2.2983	0.03662	0.202683	-4.34999	4.259863158	4.105517043	4.57178538	4.627834768	4.570565051	4.72169024
ENSMUSG00000005881.13	Ergic3	0.309732	6.234106	2.599695	0.020326	0.158008	-3.92441	6.140715892	5.858311339	6.23461308	6.328452608	6.343647547	6.498894324
ENSMUSG00000028643.10	Svbp	0.310119	5.343362	2.698679	0.016698	0.143634	-3.7118	5.14389714	5.071753062	5.34176367	5.459790066	5.426707047	5.616263885
ENSMUSG00000044715.6	Gskip	0.3103	4.650137	2.653476	0.01827	0.150777	-3.72712	4.436289725	4.409882576	4.62806786	4.785615583	4.782152692	4.858816473
ENSMUSG00000025355.6	Mmp19	0.310363	5.375838	2.631842	0.019072	0.153655	-3.84006	5.139653916	5.097795196	5.41629962	5.451963491	5.515304153	5.634011255
ENSMUSG00000041481.16	Serpina3g	0.310541	8.859965	2.454767	0.027034	0.179059	-4.13161	8.665154544	8.533094587	8.91687577	8.935684273	8.965505557	9.143476723
ENSMUSG00000029217.16	Tec	0.310608	5.069111	2.391308	0.030594	0.188589	-4.2602	4.914780505	4.681897157	5.12776125	5.222797328	5.10249279	5.364937686
ENSMUSG00000006736.8	Tspan31	0.311537	5.775202	2.731049	0.015654	0.138906	-3.66979	5.690938867	5.428471602	5.7332795	5.95853588	5.845040398	5.9949472
ENSMUSG00000029925.13	Tbxas1	0.311566	5.002752	2.28413	0.037631	0.204554	-4.4455	4.889733087	4.567280479	5.06612923	5.053830228	5.105032746	5.334507506
ENSMUSG00000032754.14	Slc8b1	0.31157	6.774414	2.444519	0.027581	0.180155	-4.20698	6.693112147	6.355874473	6.80550039	6.840101808	6.89396266	7.057932541
ENSMUSG00000027255.14	Arfgap2	0.311698	4.454099	2.631716	0.019077	0.153655	-3.73846	4.259863158	4.288503337	4.34023296	4.632458795	4.680234367	4.523303281
ENSMUSG00000059288.13	Cdyl	0.311894	4.007817	2.301213	0.036416	0.202683	-4.25735	3.738010701	3.758993282	4.04185029	4.116235885	4.19480754	4.197003982
ENSMUSG00000050490.7	Gm8394	0.3121	5.547841	2.61787	0.019608	0.155139	-3.87548	5.403042401	5.277152412	5.48993694	5.679019701	5.543667354	5.894225621
ENSMUSG00000031488.13	Rab11fip1	0.312296	6.490844	2.482124	0.025624	0.175594	-4.14182	6.362653277	6.078307799	6.56065494	6.574166104	6.647938695	6.721344394
ENSMUSG00000046711.15	Hmga1	0.312399	7.871786	2.607893	0.019999	0.156849	-3.87677	7.76201848	7.570538705	7.81462353	8.014738695	7.877900754	8.190896932
ENSMUSG00000052776.10	Oas1a	0.312685	9.044903	2.448814	0.027351	0.180155	-4.13702	8.873375645	8.713189593	9.08006731	9.145343026	9.099550379	9.357890797
ENSMUSG00000040843.10	Tiprl	0.312738	4.740992	2.674597	0.017518	0.14757	-3.69978	4.497257742	4.567280479	4.6870619	4.81031282	4.886230959	4.997805721
ENSMUSG00000015396.3	Cd83	0.313014	8.871571	2.50261	0.024615	0.171946	-4.04345	8.697990088	8.546393598	8.90138913	8.939654138	8.975950453	9.168047986
ENSMUSG00000020143.15	Dock2	0.313695	6.435641	2.473144	0.026079	0.176568	-4.15875	6.223237028	6.078307799	6.53278232	6.605077934	6.47119346	6.703245208
ENSMUSG00000025737.5	Wdr24	0.31376	4.066054	2.519718	0.023801	0.170068	-3.87615	3.907311834	3.942634277	3.88271448	4.212025155	4.223142887	4.228496529
ENSMUSG00000031266.6	Gla	0.314423	8.398201	2.560861	0.021948	0.163601	-3.94941	8.216957333	8.081247742	8.42517108	8.453107514	8.526124267	8.686600066
ENSMUSG00000031887.13	Tradd	0.314918	4.455021	2.590277	0.020709	0.158886	-3.81555	4.195910525	4.251861493	4.4293165	4.686821083	4.5960679	4.570148145
ENSMUSG00000018819.10	Lsp1	0.31505	6.68187	2.546169	0.022593	0.165893	-4.02103	6.486438519	6.349385579	6.73502786	6.736527021	6.781755856	7.002082911
ENSMUSG00000042333.14	Tnfrsf14	0.315101	4.594987	2.176401	0.046215	0.217853	-4.5818	4.422384123	4.125908832	4.73444531	4.655359197	4.762980382	4.868843933
ENSMUSG00000021905.13	Dph3	0.315152	3.669084	2.222854	0.042311	0.212176	-4.31966	3.45348534	3.47093661	3.60530795	3.808270789	3.764587842	3.911912655
ENSMUSG00000003437.14	Paf1	0.315279	5.827206	2.918663	0.010741	0.111042	-3.31023	5.601208706	5.655839343	5.74951579	5.919339943	5.924841109	6.112491915
ENSMUSG00000104713.4	Gbp6	0.315322	7.688317	2.658599	0.018085	0.150179	-3.78665	7.523956064	7.379951159	7.68856644	7.785356082	7.765591596	7.986481413
ENSMUSG00000039911.13	Spsb1	0.315488	5.057981	2.518539	0.023856	0.170191	-4.0264	4.822498571	4.749167853	5.11707457	5.111275324	5.200756806	5.347115408
ENSMUSG00000045211.4	Nudt18	0.315892	4.101741	2.163746	0.047335	0.219385	-4.51531	3.749194439	3.79763921	4.2518126	4.260625794	4.26916577	4.282006585
ENSMUSG00000055200.7	Sertad3	0.316207	4.601943	2.219259	0.042602	0.212361	-4.50836	4.290806865	4.233185568	4.77579927	4.789761261	4.693796817	4.82830901
ENSMUSG00000025372.16	Baiap2	0.316693	5.030824	2.689139	0.017018	0.145717	-3.70468	4.843521822	4.762253612	5.00174633	5.222797328	5.058614961	5.296008912
ENSMUSG00000034674.18	Tdg	0.316803	3.819429	2.259977	0.039415	0.208097	-4.28972	3.546167343	3.705800343	3.74142048	3.848572153	4.006584258	4.068031857
ENSMUSG00000063234.4	Gpr84	0.316941	5.923962	2.572955	0.02143	0.161195	-3.97129	5.688036059	5.587639567	6.014279	6.016287482	6.054021432	6.183509075
ENSMUSG00000049047.11	Armcx3	0.317148	5.39245	2.705951	0.016458	0.14248	-3.70101	5.222234112	5.148510261	5.32638298	5.500829492	5.420602545	5.736141396
ENSMUSG00000021285.14	Ppp1r13b	0.317214	4.379688	2.296652	0.036737	0.202785	-4.33614	4.162838703	3.987899011	4.48024044	4.580760607	4.459827761	4.606558785
ENSMUSG00000030522.14	Mtmr10	0.317264	4.118041	2.37611	0.03151	0.191413	-4.14636	4.022404999	3.771990623	4.07167451	4.17446915	4.326881267	4.34082523
ENSMUSG00000083902.2	Gm15975	0.317376	4.820586	2.42331	0.028746	0.183865	-4.17486	4.630231608	4.482843377	4.85511241	5.004742308	4.819749478	5.130837058
ENSMUSG00000024608.10	Rps14	0.317803	8.210468	2.62894	0.019182	0.153861	-3.82758	8.008801394	7.902936263	8.24426188	8.327205296	8.296229803	8.483375326
ENSMUSG00000052160.6	Pld4	0.31788	6.87032	2.295033	0.036851	0.203131	-4.47491	6.792406506	6.38366057	6.9580026	6.969084961	6.966989856	7.151777897
ENSMUSG00000041143.16	Tmco4	0.318607	4.46501	2.527577	0.023436	0.168536	-3.93328	4.350771654	4.13599765	4.41775175	4.575967596	4.563194945	4.746375738
ENSMUSG00000016206.6	H2-M3	0.318712	6.312631	2.534617	0.023113	0.167352	-4.04569	6.130060785	5.929643438	6.39655139	6.44475164	6.391248108	6.583529538
ENSMUSG00000033192.4	Lpcat2	0.319884	4.567674	2.521978	0.023696	0.169529	-3.95924	4.415380735	4.195092397	4.58735418	4.798017049	4.666543208	4.743653671
ENSMUSG00000031109.16	Enox2	0.320103	4.707608	2.776524	0.014293	0.131275	-3.50156	4.587254535	4.418173949	4.62806786	4.830575724	4.856391322	4.925187233
ENSMUSG00000075327.9	Zbtb2	0.320128	4.293938	2.362621	0.032345	0.193723	-4.20343	4.085532977	3.919457955	4.37054279	4.408141497	4.459827761	4.520125538
ENSMUSG00000016283.13	H2-M2	0.320294	4.991512	2.807815	0.013424	0.126604	-3.47485	4.774045303	4.844534836	4.87639148	5.097963165	5.063846862	5.292288226
ENSMUSG00000096727.2	Psmb9	0.320595	7.942596	2.485758	0.025443	0.175264	-4.1015	7.749542538	7.573325624	8.0255137	8.012523254	8.068246568	8.226426403
ENSMUSG00000025287.15	Acot9	0.320615	5.52524	2.595898	0.02048	0.158008	-3.91538	5.40657842	5.182979018	5.49819666	5.508396091	5.709738361	5.845548766
ENSMUSG00000026536.9	Ifi211	0.320665	8.393874	2.571716	0.021483	0.161318	-3.92932	8.213933342	8.059547727	8.42841634	8.509496246	8.458711076	8.693140107
ENSMUSG00000052102.14	Gnpda1	0.320834	4.579103	2.589729	0.020731	0.158889	-3.83587	4.470478411	4.270298741	4.50777362	4.641662622	4.71057253	4.873831648
ENSMUSG00000028149.12	Rap1gds1	0.321003	6.960644	2.564578	0.021788	0.162831	-3.98162	6.795106919	6.594057668	7.01091552	7.055122387	7.035832485	7.272831649
ENSMUSG00000021196.13	Pfkp	0.321116	8.896376	2.445677	0.027519	0.180155	-4.14713	8.698350762	8.525919364	8.98506899	8.98329546	9.018337956	9.167286435
ENSMUSG00000024937.14	Ehbp1l1	0.321366	6.939566	2.434198	0.028142	0.181809	-4.2226	6.665339044	6.58672036	7.08498826	7.026424071	7.07716764	7.19675522
ENSMUSG00000009563.16	Tor2a	0.321554	4.310618	2.220791	0.042478	0.212294	-4.45647	4.111764519	3.871957531	4.42354572	4.590299122	4.378182496	4.487957091
ENSMUSG00000062070.12	Pgk1	0.322096	6.468853	2.541971	0.022781	0.166062	-4.03123	6.466248871	6.147263872	6.30692362	6.587325105	6.4493716	6.855983647
ENSMUSG00000020386.5	Sar1b	0.322323	4.972285	2.606225	0.020065	0.15715	-3.85468	4.843521822	4.661088694	4.92212916	4.979557466	5.127693344	5.299720026
ENSMUSG00000044876.14	Zfp444	0.322421	4.111736	2.525977	0.02351	0.168973	-3.8727	3.927143006	3.871957531	4.04185029	4.28432563	4.204314625	4.34082523
ENSMUSG00000020806.15	Rhbdf2	0.322543	7.794545	2.797583	0.013702	0.127864	-3.51931	7.578268186	7.530945765	7.79128654	7.918156921	7.878272008	8.070342018
ENSMUSG00000028420.13	Tmem38b	0.322572	4.446576	2.445535	0.027526	0.180155	-4.08016	4.470478411	4.010009712	4.35243341	4.655359197	4.59967464	4.591499201
ENSMUSG00000031555.13	Adam9	0.323023	7.671321	2.722614	0.015919	0.139769	-3.66588	7.464977588	7.373569895	7.6915708	7.770749029	7.76759701	7.959462775
ENSMUSG00000037280.12	Galnt6	0.323141	6.161295	2.497471	0.024865	0.173059	-4.11409	5.967203583	5.789672901	6.23625999	6.157725887	6.349014722	6.467893746
ENSMUSG00000027207.15	Galk2	0.323424	4.586941	2.286297	0.037475	0.204486	-4.38797	4.587254535	4.053239764	4.60275677	4.760488184	4.807325763	4.71058185
ENSMUSG00000037921.14	Ddx60	0.323428	6.594191	2.764091	0.014653	0.133036	-3.61101	6.378944862	6.291891272	6.62492815	6.706141949	6.686607643	6.876629678
ENSMUSG00000002983.16	Relb	0.323597	6.918803	2.657106	0.018139	0.150248	-3.80893	6.685855114	6.590393678	6.99543892	7.065418202	7.007602751	7.168111431
ENSMUSG00000036528.14	Ppfibp2	0.324122	5.211891	2.809739	0.013372	0.126544	-3.49036	4.996828752	4.962612569	5.18345724	5.332010427	5.288289493	5.508146268
ENSMUSG00000054277.7	Arfgap3	0.324428	4.869422	2.511647	0.024182	0.170977	-4.01918	4.52354904	4.742580193	4.85511241	4.916499059	4.961043474	5.217748328
ENSMUSG00000027995.10	Tlr2	0.324469	6.084409	2.766796	0.014574	0.132857	-3.60701	5.938179401	5.750810078	6.07259665	6.154514544	6.197785239	6.392568805
ENSMUSG00000032002.9	Dcun1d5	0.324818	4.718628	2.860253	0.012082	0.119071	-3.34253	4.510463281	4.537144761	4.61799665	4.87417697	4.813550994	4.958435998
ENSMUSG00000042396.9	Rbm7	0.325454	6.390127	2.843111	0.012505	0.121569	-3.46101	6.215194108	6.067805878	6.39655139	6.500197147	6.506204967	6.654806562
ENSMUSG00000024955.13	Esrra	0.325714	5.720079	2.48172	0.025645	0.175594	-4.13497	5.625687683	5.263412356	5.77239803	5.796987656	5.851105336	6.010882455
ENSMUSG00000061650.6	Med9	0.326742	3.899629	2.399244	0.030125	0.18785	-4.05946	3.726739589	3.63642664	3.84002808	3.992204842	4.033500991	4.168872547
ENSMUSG00000018761.14	Mpdu1	0.326892	5.220986	2.578101	0.021213	0.160463	-3.92941	5.139653916	4.794457963	5.22386208	5.379545378	5.288289493	5.500104469
ENSMUSG00000027219.13	Slc28a2	0.327162	9.650337	2.562379	0.021882	0.163405	-3.90984	9.438126521	9.33918789	9.68382184	9.759233465	9.722665776	9.958986148
ENSMUSG00000016308.12	Ube2a	0.327182	4.752864	2.257775	0.039581	0.208339	-4.46183	4.358094995	4.426417943	4.95450516	4.931586203	4.955424402	4.891154095
ENSMUSG00000018654.17	Ikzf1	0.327216	7.342238	2.680995	0.017297	0.14661	-3.75377	7.131664076	7.018896277	7.38583323	7.43254072	7.438587388	7.645904933
ENSMUSG00000068396.9	Rpl34-ps1	0.327423	6.117918	2.971975	0.009645	0.104125	-3.2108	6.005015937	5.888462803	5.96745315	6.298215378	6.130812686	6.417547432
ENSMUSG00000032333.5	Stoml1	0.327568	4.887924	2.503521	0.024571	0.171946	-4.03581	4.869377264	4.458930757	4.82915617	5.011857957	4.958236674	5.199985785
ENSMUSG00000036712.14	Cyld	0.327688	5.499402	2.75482	0.014927	0.134616	-3.61347	5.304138795	5.148510261	5.54412904	5.64051535	5.62024021	5.73887766
ENSMUSG00000038188.17	Scarf1	0.328117	5.921553	2.950248	0.010078	0.10682	-3.2506	5.834361823	5.594972213	5.83894457	6.056413389	6.022122683	6.182504412
ENSMUSG00000004267.16	Eno2	0.329161	4.161795	2.246053	0.040479	0.209437	-4.38392	3.985051584	3.692189912	4.27763333	4.29603113	4.322522669	4.397339483
ENSMUSG00000039007.9	Cpq	0.329325	4.645444	2.337851	0.033932	0.197603	-4.30488	4.408343185	4.233185568	4.77579927	4.691260149	4.85337313	4.910699797
ENSMUSG00000028048.11	Gba	0.329512	6.698774	2.555675	0.022174	0.164159	-4.003	6.50634952	6.309827106	6.78426747	6.789243702	6.795187397	7.007766176
ENSMUSG00000020589.16	Fam49a	0.329856	6.410402	2.895871	0.011246	0.113636	-3.35889	6.209132356	6.142267521	6.3817468	6.44475164	6.570105521	6.714410014
ENSMUSG00000002546.16	Golga2	0.330179	5.695881	2.883349	0.011533	0.115408	-3.37455	5.525176446	5.382601711	5.67860741	5.748823552	5.881053376	5.959022817
ENSMUSG00000047989.11	Ino80c	0.330307	4.005005	2.4135	0.029301	0.185875	-4.05659	3.738010701	4.020939272	3.77806232	4.199614799	4.04941323	4.243988706
ENSMUSG00000048170.14	Mcmbp	0.330522	7.349621	2.787731	0.013975	0.129196	-3.55032	7.1871995	7.021619629	7.34520184	7.496714286	7.402906837	7.644081506
ENSMUSG00000060739.7	Nsa2	0.330862	5.434497	2.374139	0.031631	0.191705	-4.31827	5.34156489	4.990681451	5.46205791	5.579563847	5.414472104	5.818641595
ENSMUSG00000023004.8	Tuba1b	0.33123	7.500643	2.477498	0.025858	0.176099	-4.12924	7.424565728	7.224347303	7.3566036	7.764969835	7.357827139	7.8755422
ENSMUSG00000037960.10	Card19	0.331653	6.174587	2.668093	0.017747	0.148838	-3.79525	6.005015937	5.773607088	6.2428289	6.306919447	6.288848135	6.430299681
ENSMUSG00000084106.4	Gm6136	0.331705	6.673218	2.663258	0.017918	0.149529	-3.80158	6.627961434	6.481854891	6.41120561	6.897895691	6.59290459	7.027483127
ENSMUSG00000027822.16	Slc33a1	0.331723	5.357789	3.038651	0.008428	0.097299	-3.05627	5.253986591	5.076999232	5.24036907	5.472740736	5.486372133	5.616263885
ENSMUSG00000032119.4	Hinfp	0.331965	3.680014	2.141701	0.049347	0.222295	-4.45952	3.493937384	3.265711472	3.75066856	3.848572153	3.770999831	3.950195367
ENSMUSG00000078922.9	Tgtp1	0.331969	6.835943	2.709541	0.01634	0.141901	-3.71122	6.668287834	6.473922527	6.86738619	6.982729719	6.888075602	7.135257322
ENSMUSG00000019039.13	Dalrd3	0.331969	4.048579	2.303849	0.036232	0.202683	-4.26028	4.094329925	3.650570823	3.89110226	4.129381261	4.361283972	4.164808614
ENSMUSG00000029084.5	Cd38	0.33201	6.752876	2.78608	0.014022	0.129406	-3.5666	6.512925919	6.449860343	6.79659828	6.845090385	6.870268906	7.042511337
ENSMUSG00000035007.5	Rundc1	0.332116	4.380128	2.413297	0.029313	0.185875	-4.12794	4.456900005	4.105517043	4.08635855	4.527140769	4.419582612	4.685270761
ENSMUSG00000039236.18	Isg20	0.332202	7.593792	2.636094	0.018912	0.152876	-3.83167	7.320632304	7.28340647	7.68072568	7.722228104	7.679595488	7.876163717
ENSMUSG00000019433.8	Gipc1	0.332304	5.406244	3.153545	0.006676	0.085893	-2.83337	5.238197706	5.187836581	5.29195695	5.495762954	5.614903478	5.608804225
ENSMUSG00000090523.2	Gypc	0.332651	5.540186	2.72851	0.015733	0.139139	-3.66545	5.374437845	5.153485076	5.58347771	5.638218064	5.696325643	5.795172022
ENSMUSG00000030793.4	Pycard	0.332662	5.044887	2.196297	0.044504	0.214943	-4.60309	4.695282157	4.632868821	5.28242369	5.160129711	5.169549207	5.329070206
ENSMUSG00000040522.5	Tlr8	0.332833	5.586986	2.819579	0.013111	0.125115	-3.49336	5.431090818	5.244886322	5.57829281	5.645098976	5.744364704	5.878181798
ENSMUSG00000011752.6	Pgam1	0.332927	5.812963	2.910662	0.010916	0.111729	-3.32509	5.634761266	5.504420455	5.794923	5.956693357	5.88401427	6.102965647
ENSMUSG00000018507.16	Trpv2	0.333061	6.397564	2.592311	0.020626	0.158429	-3.93771	6.258885391	5.97811799	6.45287998	6.543805239	6.45336384	6.698333304
ENSMUSG00000051506.16	Wdfy4	0.333158	4.404573	2.198918	0.044283	0.214756	-4.51065	4.328575908	3.84760767	4.49682332	4.618542015	4.467743803	4.668146583
ENSMUSG00000021585.8	Cast	0.333194	6.12659	2.870158	0.011843	0.117456	-3.40846	6.005015937	5.770372345	6.09999042	6.209706331	6.266331954	6.408123619
ENSMUSG00000068749.9	Psma5	0.333335	5.714049	2.715568	0.016145	0.140589	-3.69698	5.488942474	5.464946172	5.68344461	5.904123506	5.674260466	6.068575757
ENSMUSG00000059586.9	Nsmce2	0.334189	3.796778	2.294664	0.036877	0.203144	-4.22255	3.45348534	3.79763921	3.63557539	4.055552849	4.001140119	3.837273854
ENSMUSG00000037849.7	Ifi206	0.334425	8.999177	2.684559	0.017174	0.146277	-3.70003	8.804016104	8.668709922	9.02420351	9.103906411	9.101300644	9.292928402
ENSMUSG00000038775.14	Vill	0.334506	3.766385	2.3129	0.035606	0.20128	-4.18404	3.669024091	3.533333147	3.60530795	3.840601442	3.851861663	4.098178821
ENSMUSG00000049891.3	Gm7984	0.334857	3.80172	2.158635	0.047795	0.220019	-4.45877	3.846128318	3.388943238	3.64552505	4.082838675	3.796366551	4.050517894
ENSMUSG00000031662.7	Snx20	0.335086	6.967503	2.819068	0.013124	0.125115	-3.49926	6.80853368	6.595886179	6.99641109	7.121590648	7.086837214	7.195759743
ENSMUSG00000036114.3	Rpp25l	0.335338	3.433938	2.259699	0.039436	0.208097	-4.19978	3.294628576	3.247215128	3.26154961	3.587920399	3.542730843	3.669581387

ENSMUSG00000083892.3	Gm1848	0.335431	5.255794	2.735012	0.01553	0.138104	-3.63609	4.98268457	4.95693266	5.31395927	5.357370954	5.385513812	5.538302708
ENSMUSG00000026248.9	Mrpl44	0.335744	4.040699	2.491749	0.025145	0.173779	-3.92014	3.825143293	3.719283573	4.04936435	4.242591871	4.156140031	4.251672852
ENSMUSG00000040613.14	Apobec1	0.335865	7.243589	2.724245	0.015868	0.139769	-3.67422	7.099254738	6.86969211	7.25871643	7.364684572	7.316234023	7.552953451
ENSMUSG00000039217.12	Il18	0.336934	4.019587	2.153419	0.048268	0.220594	-4.51453	3.803848514	3.548519551	4.15761724	4.236530119	4.070360169	4.300646315
ENSMUSG00000004788.11	Eif2b2	0.337161	5.190195	2.663548	0.017908	0.149529	-3.76638	4.90980569	4.868936179	5.27282702	5.30619611	5.306059633	5.477345991
ENSMUSG00000062054.6	Iah1	0.337226	3.737208	2.142929	0.049233	0.222208	-4.47148	3.520288711	3.47093661	3.71331514	3.824526679	3.758147229	4.136035965
ENSMUSG00000036833.16	Pnpla7	0.337544	4.157502	2.284549	0.037601	0.204554	-4.31491	4.049798765	3.63642664	4.23872684	4.347561431	4.296090875	4.376405209
ENSMUSG00000027944.13	Hax1	0.337926	5.17776	2.464396	0.02653	0.177689	-4.13593	4.90980569	4.850673973	5.25344001	5.259131548	5.214935741	5.578573784
ENSMUSG00000000282.12	Mnt	0.338079	4.992026	2.844868	0.012461	0.121348	-3.40291	4.864243112	4.647047756	4.94647902	5.114584259	5.10249279	5.277308779
ENSMUSG00000022350.6	Washc5	0.338102	5.327337	2.95031	0.010077	0.10682	-3.22654	5.105251212	5.050575507	5.31083655	5.520919565	5.39176828	5.584670761
ENSMUSG00000083041.2	Gmfg-ps	0.338216	5.368021	2.82718	0.012912	0.123939	-3.46734	5.100892613	5.187836581	5.30457076	5.565124743	5.353828167	5.695871066
ENSMUSG00000045404.15	Kcnk13	0.338299	4.920777	2.448213	0.027383	0.180155	-4.14044	4.869377264	4.426417943	4.93840798	5.025984827	5.021450568	5.243025303
ENSMUSG00000020225.10	Tmbim4	0.338478	6.43994	3.044571	0.008328	0.096751	-3.06874	6.310758843	6.139762841	6.3592511	6.564519966	6.523397167	6.741949767
ENSMUSG00000042682.8	Selenok	0.339243	5.617094	2.93535	0.010385	0.108772	-3.27068	5.49891506	5.290762844	5.54678594	5.763653147	5.674260466	5.928187224
ENSMUSG00000015750.15	Aph1a	0.339265	4.854044	2.687818	0.017063	0.145787	-3.68772	4.80116443	4.589476225	4.66277353	4.964966241	4.880312202	5.225573134
ENSMUSG00000106990.1	Gm42547	0.340041	4.382419	2.510617	0.024231	0.171043	-3.94975	4.429353678	3.987899011	4.20548355	4.618542015	4.431774474	4.621462792
ENSMUSG00000019866.13	Crybg1	0.340109	6.738075	2.635279	0.018942	0.153031	-3.85328	6.6173198	6.305364008	6.77975729	6.823010447	6.85827449	7.044724484
ENSMUSG00000027678.17	Ncoa3	0.34189	6.603799	2.706629	0.016435	0.142472	-3.72027	6.395054527	6.208300684	6.69367014	6.694027644	6.81471524	6.817024043
ENSMUSG00000036376.2	Abt1	0.341893	3.452023	2.240864	0.040882	0.210271	-4.23549	3.216201929	3.170763068	3.43219326	3.625517186	3.698850045	3.56861091
ENSMUSG00000032596.14	Uba7	0.342021	7.979383	2.896414	0.011234	0.113597	-3.32375	7.798811752	7.643957999	7.98396662	8.118091707	8.095325093	8.236142376
ENSMUSG00000045980.13	Tmem104	0.342314	6.17081	2.435946	0.028047	0.181378	-4.22683	6.002681478	5.687052713	6.30221406	6.21895935	6.361815058	6.452140023
ENSMUSG00000003746.15	Man1a	0.342946	5.116249	2.625741	0.019304	0.154133	-3.83115	4.939400472	4.702409754	5.1766117	5.179217465	5.279321662	5.420533055
ENSMUSG00000025171.1	Ubtd1	0.343555	5.682534	2.642021	0.01869	0.152281	-3.83484	5.566857202	5.216643429	5.73793711	5.861431365	5.773396998	5.938935829
ENSMUSG00000053040.13	Aph1c	0.343621	3.804339	2.175795	0.046268	0.217853	-4.43007	3.726739589	3.422301835	3.74142048	3.903167129	3.82746032	4.204941801
ENSMUSG00000054091.9	1810037I17Ri	0.344359	5.578238	2.998245	0.009146	0.100849	-3.1464	5.385231125	5.230833987	5.59379192	5.738136873	5.739468676	5.781966483
ENSMUSG00000040483.15	Xaf1	0.344576	7.902484	2.807551	0.013431	0.126604	-3.49715	7.695643508	7.568677763	7.92698113	7.994227154	8.014205486	8.215171539
ENSMUSG00000059040.4	Eno1b	0.345043	4.685968	2.800066	0.013634	0.127406	-3.45248	4.450062575	4.367695504	4.70620242	4.793895061	4.924122098	4.873831648
ENSMUSG00000043091.8	Tuba1c	0.345185	5.392908	2.592979	0.020598	0.158344	-3.913	5.307925399	4.922375438	5.41629962	5.548094418	5.452865414	5.709884727
ENSMUSG00000024410.14	3110002H16R	0.345328	4.781046	2.637114	0.018873	0.152876	-3.7738	4.648265531	4.376232186	4.78483026	4.838601865	4.932726666	5.105618149
ENSMUSG00000044424.3	Gm9493	0.346028	4.487318	2.744848	0.015228	0.136501	-3.52913	4.313586734	4.333034497	4.29670007	4.747758439	4.475616647	4.757212935
ENSMUSG00000039745.7	Htatip2	0.346863	4.48989	2.472177	0.026129	0.176671	-4.03736	4.450062575	4.010009712	4.46346473	4.686821083	4.537098975	4.791881952
ENSMUSG00000024084.7	Qpct	0.347151	4.974677	3.088063	0.007625	0.092209	-2.92819	4.779510064	4.702409754	4.91392034	5.081148347	5.167120427	5.203951959
ENSMUSG00000106037.1	Gm4332	0.348204	6.70636	2.79346	0.013816	0.128101	-3.55311	6.476379013	6.327542693	6.79212653	6.819973193	6.882904647	6.939233261
ENSMUSG00000062611.4	Rps3a2	0.348944	5.761928	2.505131	0.024493	0.171812	-4.0928	5.616556671	5.500521277	5.64180197	6.06840736	5.58610262	6.158180211
ENSMUSG00000081895.3	Rpl17-ps10	0.349087	6.270473	3.092425	0.007558	0.091718	-2.97516	6.134332276	6.073066394	6.07811718	6.374723762	6.318698063	6.643899037
ENSMUSG00000071311.1	Gpr31b	0.349607	4.223758	2.166605	0.04708	0.219041	-4.53356	4.049798765	3.942634277	4.14364429	4.476603376	4.070360169	4.659507665
ENSMUSG00000036845.12	Lin37	0.350517	3.679118	2.43022	0.028362	0.182324	-3.95381	3.608903098	3.405718951	3.48855571	3.925946997	3.738651053	3.907055092
ENSMUSG00000068329.12	Htra2	0.350601	4.800209	2.657509	0.018124	0.15022	-3.7378	4.884670994	4.367695504	4.61293455	4.912702491	4.96943126	5.053817998
ENSMUSG00000062345.10	Serpinb2	0.351064	6.001692	3.069182	0.007923	0.093916	-3.019	5.756140505	5.730978906	5.98714895	6.122001731	6.098037077	6.315844839
ENSMUSG00000025521.14	Tmem192	0.351123	5.621205	2.743242	0.015277	0.13661	-3.64052	5.388811015	5.240217402	5.69785967	5.712161167	5.749244173	5.938935829
ENSMUSG00000094530.1	Gm21399	0.351624	7.492158	2.559771	0.021995	0.162008	-3.97727	7.238693733	7.199308165	7.51015796	7.597116183	7.452616136	7.955057147
ENSMUSG00000022210.6	Dhrs4	0.351816	3.401702	2.142237	0.049297	0.222208	-4.3917	2.989779994	3.265711472	3.39728765	3.625517186	3.67853006	3.453390012
ENSMUSG00000030538.15	Cib1	0.35332	4.45385	2.641493	0.01871	0.152348	-3.718	4.365381349	4.031786655	4.41775175	4.527140769	4.621127749	4.759909562
ENSMUSG00000053101.2	Gpr141	0.353386	5.055309	2.793673	0.01381	0.128301	-3.50681	4.89477748	4.688767146	5.03996565	5.1728829	5.125193005	5.410270806
ENSMUSG00000041453.12	Rpl21	0.353878	5.822323	2.490863	0.025189	0.173964	-4.12068	5.582572989	5.448848989	5.89631997	6.028618284	5.747619517	6.229956009
ENSMUSG00000041390.18	Mdfic	0.353981	8.005848	2.884144	0.011515	0.115391	-3.34673	7.820843155	7.631532373	8.03689784	8.184540706	8.108043148	8.253228705
ENSMUSG00000032518.5	Rpsa	0.354789	6.936847	2.502795	0.024606	0.171946	-4.09658	6.804518756	6.689623599	6.78539282	7.309448273	6.731682488	7.300414799
ENSMUSG00000023904.10	Hcfc1r1	0.355092	4.433	2.592873	0.020603	0.158344	-3.80527	4.187713372	4.084832889	4.47467018	4.55663471	4.518165697	4.775984325
ENSMUSG00000003617.16	Cp	0.3551	6.256516	2.87456	0.011739	0.116945	-3.4002	5.945490371	5.969681153	6.31785318	6.358069089	6.371341325	6.576663182
ENSMUSG00000039041.15	Adrm1	0.355294	4.7274	2.610623	0.019891	0.156094	-3.81597	4.350771654	4.490726976	4.78932464	5.00830452	4.743549852	4.981724559
ENSMUSG00000016498.9	Pdcd1lg2	0.356857	4.293731	2.581848	0.021057	0.159689	-3.80133	4.146014081	3.835276868	4.33409382	4.424231199	4.544603257	4.478165006
ENSMUSG00000072946.12	Ptgr2	0.356941	3.533555	2.22314	0.042288	0.212176	-4.28385	3.383437843	3.04790632	3.58477102	3.783538268	3.732093337	3.669581387
ENSMUSG00000003380.11	Rabac1	0.357203	6.293317	2.639019	0.018802	0.15282	-3.85006	6.086635534	5.830625509	6.42282274	6.473385228	6.410883946	6.535551348
ENSMUSG00000020437.12	Myo1g	0.357687	7.179273	2.861117	0.012061	0.119064	-3.4134	6.99036321	6.780858852	7.23172645	7.340866778	7.280726566	7.451097538
ENSMUSG00000005510.9	Ndufs3	0.359296	5.043676	2.853409	0.012249	0.119766	-3.39106	4.833048491	4.886970102	4.8721607	5.303299131	4.98053965	5.386037806
ENSMUSG00000047798.15	Cd300lf	0.359666	4.456873	2.548663	0.022483	0.165568	-3.89071	4.23621189	4.010009712	4.55604673	4.541963109	4.683636951	4.713366976
ENSMUSG00000056144.13	Trim34a	0.359872	5.539684	2.709289	0.016348	0.141901	-3.70137	5.392382044	5.118295648	5.55736487	5.723041271	5.541793724	5.90522928
ENSMUSG00000036918.15	Ttc7	0.360429	5.756769	2.907844	0.010978	0.112023	-3.32845	5.563693395	5.374102747	5.78370447	5.823516035	5.916189238	6.079410919
ENSMUSG00000031320.9	Rps4x	0.360663	7.725911	2.761863	0.014718	0.13336	-3.58954	7.560125801	7.442257857	7.63401454	7.92146694	7.644669007	8.152932412
ENSMUSG00000048100.13	Taf13	0.360778	3.353302	2.322685	0.03494	0.200218	-4.06962	3.133265588	3.228478566	3.16800884	3.529621758	3.425691422	3.634746011
ENSMUSG00000039450.11	Dcxr	0.361316	4.722407	2.457852	0.026872	0.17851	-4.09713	4.429353678	4.26110957	4.90151888	4.893568324	4.85337313	4.995519357
ENSMUSG00000029468.17	P2rx7	0.361711	3.863202	2.4571	0.026911	0.178596	-3.94495	3.856507464	3.422301835	3.75066856	4.034744387	3.962444829	4.152547676
ENSMUSG00000091971.3	Hspa1a	0.36234	4.915639	2.719685	0.016013	0.14035	-3.6333	4.779510064	4.567280479	4.84651211	5.060708406	4.912568923	5.32725321
ENSMUSG00000012428.9	Steap4	0.363746	3.801347	2.279271	0.037984	0.205148	-4.24711	3.608903098	3.283973678	3.90773294	4.089580253	4.001140119	3.916753918
ENSMUSG00000015341.16	Golga7	0.363954	6.034486	2.478558	0.025814	0.176099	-4.14758	5.786312033	5.554173491	6.20800354	6.154514544	6.127068665	6.376844452
ENSMUSG00000074506.4	Gm10705	0.365707	4.845716	2.917659	0.010763	0.111096	-3.24557	4.52354904	4.611335659	4.84219266	5.070964575	4.889181257	5.137073502
ENSMUSG00000027900.15	Dram2	0.365767	6.47109	3.204259	0.006022	0.080896	-2.75357	6.295582424	6.132222606	6.43434708	6.600365165	6.588373508	6.775651254
ENSMUSG00000039168.15	Dap	0.369827	6.114721	2.642575	0.01867	0.152281	-3.84248	5.873215922	5.645282991	6.26397363	6.280648098	6.221388139	6.403819633
ENSMUSG00000072109.11	A530040E14Ri	0.37053	6.249899	2.996144	0.009185	0.100849	-3.16364	6.115010178	5.915070637	6.16022216	6.423564365	6.236525514	6.648999479
ENSMUSG00000040997.13	Abhd4	0.370953	4.214296	3.01495	0.008842	0.099056	-2.96318	4.049798765	3.987899011	4.04936435	4.324885993	4.455853392	4.417974328
ENSMUSG00000020733.3	Slc9a3r1	0.372369	6.212048	3.178373	0.006348	0.083118	-2.80526	6.009673552	5.842995961	6.22136966	6.40070665	6.323068214	6.474475771
ENSMUSG00000025036.6	Sfxn2	0.372842	3.272971	2.31427	0.035512	0.20128	-4.0634	3.167014379	2.865908486	3.19535933	3.519672098	3.449871234	3.440000635
ENSMUSG00000010054.4	Tusc2	0.373077	3.787099	2.585599	0.020901	0.159164	-3.69309	3.57158994	3.47093661	3.75066856	3.880021792	3.951195688	4.098178821
ENSMUSG00000006205.13	Htra1	0.373186	3.903586	2.559447	0.022009	0.163601	-3.76592	3.749194439	3.517985183	3.86579117	4.069260269	3.97915577	4.24013123
ENSMUSG00000064090.14	Vrk2	0.374477	4.311033	2.884553	0.011505	0.115387	-3.23108	4.07668206	3.954084298	4.31551809	4.532098489	4.451868044	4.535944721
ENSMUSG00000031950.7	Gabarapl2	0.374903	4.092314	2.19798	0.044362	0.214943	-4.45251	3.645275488	3.931092655	4.13660674	4.193369356	4.065151878	4.582387413
ENSMUSG00000015656.16	Hspa8	0.376195	10.03148	2.765393	0.014615	0.133032	-3.51626	9.88316476	9.727334289	9.91976109	10.26020569	9.955380416	10.44301885
ENSMUSG00000030149.15	Klrk1	0.37642	4.494653	2.895304	0.011259	0.11368	-3.24121	4.336012497	4.35046909	4.23872684	4.764706593	4.475616647	4.802383873
ENSMUSG00000085939.3	Cd63-ps	0.377731	7.794693	2.87336	0.011767	0.116967	-3.37346	7.664231692	7.43000993	7.72362541	7.980237822	7.759558572	8.210497045
ENSMUSG00000082321.3	Gm14253	0.378282	5.036934	2.594939	0.020519	0.158008	-3.88015	4.94913201	4.559805359	5.01715577	5.188667467	5.029495357	5.477345991
ENSMUSG00000021367.7	Edn1	0.378638	5.847023	2.602569	0.020211	0.157682	-3.91324	5.508819184	5.420239368	6.03334994	5.971368068	5.972911828	6.175452118
ENSMUSG00000026480.12	Ncf2	0.379355	6.887605	3.001142	0.009093	0.100476	-3.1476	6.77199067	6.4679445	6.85350375	7.068833877	6.928789275	7.234567976
ENSMUSG00000027907.4	S100a11	0.379413	6.191316	2.637862	0.018845	0.152835	-3.85175	5.998001198	5.710871333	6.28957954	6.376103012	6.198974601	6.574367115
ENSMUSG00000092607.9	Scnm1	0.380421	4.329677	3.100535	0.007435	0.090943	-2.81586	4.137527615	4.095212035	4.18516356	4.461090377	4.471685595	4.627381549
ENSMUSG00000029022.18	Miip	0.382178	3.566954	2.601501	0.020254	0.157832	-3.61458	3.467095772	3.283973678	3.36151652	3.81642163	3.808884591	3.66383359
ENSMUSG00000083097.3	Gm14494	0.38365	3.567058	2.331513	0.03435	0.198941	-4.10405	3.558934639	3.354795016	3.24855227	3.587920399	3.67853006	3.973616255
ENSMUSG00000034105.8	Tldc1	0.384929	3.398832	2.476784	0.025894	0.176114	-3.80397	3.324843189	3.04790632	3.23543677	3.616209443	3.504762993	3.66383359
ENSMUSG00000020227.10	Irak3	0.386151	4.558955	2.932203	0.010452	0.109063	-3.17854	4.212166416	4.306481078	4.56131205	4.838601865	4.60686118	4.82830901
ENSMUSG00000021866.15	Anxa11	0.38702	5.112209	2.762646	0.014695	0.133241	-3.57003	4.811870936	4.661088694	5.25993133	5.262118495	5.389686468	5.28855792
ENSMUSG00000070372.11	Capza1	0.388844	6.023775	2.857378	0.012152	0.119462	-3.43137	5.981499604	5.623935696	5.87744724	6.198835595	5.983993554	6.476936309
ENSMUSG00000062353.6	Gm15772	0.391666	6.204188	3.280217	0.00516	0.074459	-2.60313	6.005015937	5.792864705	6.22303175	6.367807665	6.398513494	6.437897274
ENSMUSG00000037190.12	Cyb561d2	0.395854	3.59072	2.595137	0.020511	0.158008	-3.63163	3.279280612	3.405718951	3.4995687	3.715433822	3.698850045	3.945465215
ENSMUSG00000019505.7	Ubb	0.396302	9.381388	2.901155	0.011127	0.112857	-3.27477	9.165580354	9.026881538	9.35821159	9.588887228	9.351089146	9.797675668
ENSMUSG00000093798.1	Gm8355	0.396774	6.815225	3.280221	0.00516	0.074459	-2.59848	6.589589623	6.555113663	6.70441874	7.01407442	6.799898242	7.228253139
ENSMUSG00000013822.6	Elof1	0.397424	4.888228	3.329724	0.004666	0.070051	-2.44016	4.530047678	4.668058249	4.8593934	5.13428006	5.112625896	5.024963135
ENSMUSG00000020621.5	Rdh14	0.397583	2.970561	2.245308	0.040537	0.209457	-4.10994	2.970790891	2.540946331	2.79156659	3.158527607	3.13889155	3.222643803
ENSMUSG00000085783.3	Gm9816	0.398984	4.373111	3.05524	0.00815	0.095707	-2.91002	4.128990933	4.010009712	4.36453156	4.481737527	4.59967464	4.653719513
ENSMUSG00000029390.13	Tmed2	0.400612	5.159141	3.09705	0.007487	0.091338	-2.92578	5.006181751	4.868936179	4.99786811	5.320594383	5.152461249	5.608804225
ENSMUSG00000052906.6	Ubxn8	0.401371	3.264913	2.249647	0.040202	0.208938	-4.17265	3.06330303	2.765506589	3.33716666	3.316289969	3.601474634	3.505738432
ENSMUSG00000029762.6	Akr1b8	0.402025	3.691815	2.730786	0.015662	0.138906	-3.39977	3.45348534	3.302007601	3.68465139	3.940936172	3.857898053	3.911912655
ENSMUSG00000069049.11	Eif2s3y	0.403224	4.083735	2.559268	0.022017	0.163601	-3.80031	4.013156922	3.502472184	4.07903521	4.466279933	4.170762066	4.270706044
ENSMUSG00000026648.18	Dclre1c	0.403965	4.605185	3.076205	0.007811	0.093372	-2.90432	4.290806865	4.29752021	4.60275677	4.862415804	4.659648595	4.9179617
ENSMUSG00000068417.7	Pnp2	0.404775	4.446081	2.687687	0.017068	0.145787	-3.62699	4.244138805	3.919457955	4.53479117	4.575967596	4.581550092	4.820580268
ENSMUSG00000029759.9	Pon3	0.405474	3.53279	2.622385	0.019433	0.154577	-3.56844	3.369010772	3.319818877	3.32483586	3.529621758	3.821294947	3.832157909
ENSMUSG00000082693.3	Gm15190	0.405902	3.954062	2.234808	0.041358	0.211294	-4.3577	3.584135193	3.784871914	3.87427764	4.319161005	3.764587842	4.397339483
ENSMUSG00000020605.8	Hs1bp3	0.4076	4.524803	2.811768	0.013318	0.126119	-3.40386	4.443192585	3.987899011	4.50777362	4.571158608	4.844280484	4.794514608
ENSMUSG00000025226.11	Fbxl15	0.408832	2.870718	2.193148	0.044771	0.215501	-4.1745	2.674900657	2.510249534	2.80937787	3.024226515	2.959640757	3.245913583
ENSMUSG00000074342.3	I830077J02Rik	0.408953	5.274383	2.866901	0.011921	0.117973	-3.38239	4.987414722	4.800813458	5.40169732	5.433534654	5.39176828	5.631068479
ENSMUSG00000044968.16	Napepld	0.409599	3.184818	2.411955	0.02939	0.185875	-3.86856	2.765809153	3.004513725	3.14012981	3.519672098	3.350620989	3.328161994
ENSMUSG00000060438.5	Rps10-ps1	0.411931	4.292672	2.688886	0.017027	0.145717	-3.59883	3.927143006	4.020939272	4.29670007	4.522165953	4.264628989	4.724454024
ENSMUSG00000014856.12	Tmem208	0.413755	4.132472	3.011841	0.008898	0.099337	-2.95188	3.907311834	3.784871914	4.07167451	4.290190252	4.24633784	4.494448419
ENSMUSG00000069910.2	Spdl1	0.415547	4.172737	3.044358	0.008331	0.096751	-2.89569	4.120403437	3.719283573	4.0342969	4.413524693	4.300529941	4.448383546
ENSMUSG00000020865.16	Abcc3	0.417065	3.359653	2.5323	0.023219	0.167585	-3.69436	3.167014379	3.228478566	3.09727433	3.372652412	3.550205963	3.742290709
ENSMUSG00000042190.12	Cmklr1	0.417863	3.925478	2.69339	0.016875	0.144785	-3.5201	3.63325296	3.678449855	3.848667	3.918393603	4.101221132	4.372886439
ENSMUSG00000045128.8	Rpl18a	0.423984	6.4141	3.023013	0.008699	0.098507	-3.1103	6.095425782	6.027728438	6.48093521	6.710522051	6.379756632	6.790234099
ENSMUSG00000028893.8	Sesn2	0.425371	3.586074	2.752531	0.014996	0.134961	-3.33505	3.57158994	3.090031796	3.43219326	3.832586449	3.783738942	3.806302467

ENSMUSG00000034875.5	Nudt19	0.430104	3.702481	2.631792	0.019074	0.153655	-3.5858	3.558934639	3.265711472	3.61546782	3.977741613	3.698850045	4.098178821
ENSMUSG00000092558.3	Med20	0.430881	3.567416	2.791519	0.01387	0.128445	-3.25751	3.57158994	3.150999813	3.31239875	3.880021792	3.758147229	3.73134041
ENSMUSG00000028100.11	Nudt17	0.433012	3.125659	2.222616	0.04233	0.212176	-4.18344	2.721070838	2.685336241	3.2744309	3.269558919	3.557642552	3.245913583
ENSMUSG00000032715.9	Trib3	0.433964	3.710883	2.651074	0.018357	0.151219	-3.55164	3.30981498	3.354795016	3.79604007	3.848572153	3.857898053	4.098178821
ENSMUSG00000090877.3	Hspa1b	0.43397	4.876198	3.660126	0.002382	0.046186	-1.77914	4.636267997	4.596799566	4.73909917	5.081148347	4.966640746	5.237231339
ENSMUSG00000071640.3	Stxbp3-ps	0.434126	4.694949	3.501578	0.003288	0.056198	-2.07463	4.470478411	4.450870987	4.51321776	4.850557969	4.785323451	5.099243933
ENSMUSG00000035242.15	Oaz1	0.434468	5.503212	2.978024	0.009528	0.103577	-3.18043	5.164928052	5.197502916	5.48717315	5.784577299	5.406257448	5.978833965
ENSMUSG00000054648.7	Zfp869	0.435346	2.806315	2.254594	0.039823	0.208663	-4.05316	2.446631669	2.446824154	2.86152915	2.891640875	3.057304016	3.13395865
ENSMUSG00000068523.12	Gng5	0.436138	5.969653	3.297512	0.004982	0.072879	-2.56573	5.799820512	5.580269461	5.86897945	6.195714566	5.941990732	6.431145837
ENSMUSG00000020038.9	Cry1	0.439993	2.712741	2.260768	0.039355	0.208097	-4.02101	2.552565114	2.380481659	2.55868179	2.828562193	2.830533154	3.125619349
ENSMUSG00000055172.10	C1ra	0.440995	5.474573	3.467173	0.003527	0.058645	-2.21123	5.164928052	5.050575507	5.53345225	5.674542754	5.667402722	5.75653799
ENSMUSG00000042569.13	Dhrs7b	0.443242	3.357689	2.477557	0.025855	0.176099	-3.79028	3.116090444	3.170763068	3.1117014	3.791829642	3.280445694	3.675306375
ENSMUSG00000029330.8	Cds1	0.443622	4.124852	2.898259	0.011192	0.113261	-3.16752	3.907311834	3.593142542	4.17832613	4.218190528	4.373976385	4.478165006
ENSMUSG00000035372.1	1810055G02R	0.448418	2.62593	2.244199	0.040622	0.209578	-4.02849	2.332298994	2.510249534	2.37951295	2.891640875	2.647503165	2.994374816
ENSMUSG00000091498.4	Mpc1-ps	0.451385	4.94137	3.746303	0.001999	0.042282	-1.61276	4.671965366	4.552291305	4.90566456	5.198055971	5.10249279	5.217748328
ENSMUSG00000022335.16	Zfat	0.454903	3.130826	2.339468	0.033826	0.197441	-3.98458	2.809201749	3.069122806	2.89527794	3.024226515	3.376080974	3.611046176
ENSMUSG00000024217.9	Snrpc	0.457664	4.819471	3.320018	0.004759	0.070797	-2.44947	4.470478411	4.521837508	4.7667114	5.117885622	4.810441736	5.22946968
ENSMUSG00000028397.13	Kdm4c	0.459744	3.6384	2.752688	0.014991	0.134961	-3.34555	3.657198655	3.265711472	3.31239875	3.697893241	4.096123265	3.801075306
ENSMUSG00000097534.1	Gm16675	0.4608	2.902325	2.399692	0.030099	0.187803	-3.82155	2.390597634	2.685336241	2.89527794	3.316289969	3.078137519	3.048313623
ENSMUSG00000094242.2	Gm5456	0.465198	4.152333	2.471041	0.026187	0.176475	-3.97437	4.137527615	3.822839764	3.80494562	4.486853473	3.962444829	4.699387263
ENSMUSG00000040584.8	Abcb1a	0.465692	2.895337	2.559671	0.022	0.163601	-3.53556	2.577876203	2.65759125	2.75527046	3.119533475	2.992935197	3.268813985
ENSMUSG00000039164.2	Naif1	0.469028	3.03159	2.292606	0.037024	0.203267	-4.04073	2.787668587	2.600447343	3.02289214	2.922175243	3.350620989	3.505738432
ENSMUSG00000038260.9	Trpm4	0.478861	3.700522	2.683107	0.017224	0.146378	-3.48597	3.411866251	3.04790632	3.86579117	3.94837276	3.869895586	4.059301452
ENSMUSG00000009376.15	Met	0.484744	3.562444	3.18067	0.006318	0.08304	-2.50853	3.216201929	3.247215128	3.48855571	3.741351197	3.764587842	3.916753918
ENSMUSG00000043432.7	Leng9	0.487599	2.47629	2.160882	0.047592	0.219824	-4.12942	2.271544963	1.853733772	2.51593215	2.860446254	2.516258632	2.83982504
ENSMUSG00000026544.6	Dusp23	0.491932	3.0004	2.472728	0.026101	0.176623	-3.71466	2.851327224	2.540946331	2.87850223	3.132648975	3.067758373	3.531217152
ENSMUSG00000082394.1	Gm4596	0.49255	3.810176	2.567726	0.021653	0.162232	-3.72274	3.703929706	3.069122806	3.85725449	4.199614799	3.802639148	4.228496529
ENSMUSG00000084067.3	Gm14269	0.493038	4.019453	2.270678	0.038615	0.206554	-4.30561	3.782234701	3.976715272	3.59507603	4.358766886	3.738651053	4.665272688
ENSMUSG00000096255.1	Dynlt1b	0.494396	2.575319	2.151687	0.048426	0.22073	-4.17116	2.390597634	2.446824154	2.2021167	2.745625852	2.516258632	3.150494017
ENSMUSG00000052151.11	Plpp2	0.496168	3.281787	2.85611	0.012183	0.119504	-3.07188	3.369010772	2.88995396	2.86152915	3.437503556	3.527663395	3.605059881
ENSMUSG00000059883.16	Irak4	0.498934	4.394196	3.155663	0.006647	0.085817	-2.71117	4.111764519	3.758993282	4.51321776	4.627834768	4.746806502	4.606558785
ENSMUSG00000049832.6	Rbx1-ps	0.502804	3.9902	2.992126	0.00926	0.101526	-2.95873	3.715379726	3.692189912	3.80494562	4.307642421	3.916912213	4.504130972
ENSMUSG00000042111.9	Ccdc115	0.512034	3.505764	2.926543	0.010572	0.109854	-2.98354	3.232232039	2.913605228	3.56393752	3.643954434	3.916912213	3.763945075
ENSMUSG00000068457.14	Uty	0.512065	3.247395	2.219953	0.042546	0.212347	-4.21305	3.546167343	2.446824154	2.92825526	3.775198967	3.225479235	3.562445537
ENSMUSG00000083283.3	Gm15361	0.515878	3.712826	3.531191	0.003096	0.054188	-1.85068	3.294628576	3.422301835	3.63557539	3.925946997	3.97915577	4.019347344
ENSMUSG00000021403.2	Serpinb9b	0.518063	2.635921	2.419043	0.028987	0.184609	-3.72576	2.473853138	2.199909414	2.47187697	2.779374643	2.71556744	3.174947061
ENSMUSG00000028716.15	Pdzk1ip1	0.520714	3.78181	3.486254	0.003392	0.057392	-1.95196	3.467095772	3.4549065	3.63557539	4.034744387	3.893595422	4.204941801
ENSMUSG00000035399.12	Oser1	0.522102	3.839987	3.205398	0.006009	0.08079	-2.51379	3.383437843	3.56354776	3.77806232	4.122823545	3.887706862	4.304345535
ENSMUSG00000081087.1	Rps15a-ps7	0.523192	5.012529	3.150752	0.006714	0.085977	-2.80228	4.630231608	4.618549149	4.98617052	5.398672297	4.889181257	5.552370269
ENSMUSG00000070524.1	Fcrlb	0.524955	2.334491	2.418643	0.029009	0.184665	-3.65943	1.999172692	1.993458536	2.22883404	2.400028851	2.741918767	2.643534029
ENSMUSG00000066245.5	Gm10156	0.528323	3.492974	2.956998	0.009941	0.105823	-2.92358	3.397722069	3.13096206	3.16800884	3.775198967	3.489291584	3.996662992
ENSMUSG00000095419.1	Gm14328	0.531774	4.148586	3.2674	0.005297	0.075533	-2.4494	3.803848514	3.692189912	4.13660674	4.242591871	4.322522669	4.693757227
ENSMUSG00000062825.15	Actg1	0.53178	8.887975	4.020068	0.001149	0.03138	-1.06918	8.676549138	8.509037083	8.68148145	9.236435201	8.87762225	9.346723074
ENSMUSG00000001642.18	Akr1b3	0.532277	3.675513	3.501427	0.003289	0.056198	-1.90179	3.369010772	3.337412937	3.52134564	3.864382668	3.845799911	4.115126975
ENSMUSG00000019295.8	Tmem129	0.540372	3.521481	3.170989	0.006444	0.084113	-2.51676	3.467095772	3.004513725	3.2871982	3.634765262	3.738651053	3.996662992
ENSMUSG00000027935.14	Rab13	0.544943	2.956675	2.598763	0.020364	0.158008	-3.47629	2.851327224	2.571003565	2.67981823	2.828562193	3.376080974	3.433259057
ENSMUSG00000008668.14	Rps18	0.548848	6.161409	3.181253	0.006311	0.08304	-2.79687	5.826463837	5.805561773	6.02575179	6.615625711	5.923402729	6.771648398
ENSMUSG00000056019.12	Zfp709	0.54894	2.426308	2.587783	0.020811	0.158953	-3.37962	2.107425583	2.037179913	2.30613976	2.675663294	2.561346521	2.87009248
ENSMUSG00000108381.1	Gm9299	0.549137	2.111881	2.252174	0.040008	0.208688	-3.8919	1.840910608	1.699011178	1.90359321	2.71106863	2.3377205	2.178982574
ENSMUSG00000089844.7	A530032D15R	0.549842	3.784246	3.933746	0.001368	0.034312	-1.06882	3.45348534	3.438696273	3.61546782	4.109618006	4.096123265	3.992083004
ENSMUSG00000035274.13	Tpbg	0.554412	2.539182	2.604013	0.020153	0.157448	-3.37415	2.30224176	2.310940726	2.17489523	2.981371036	2.546473245	2.919168471
ENSMUSG00000045868.12	Gvin1	0.554598	4.298885	3.445974	0.003682	0.060104	-2.12108	4.212166416	3.771990623	4.05683947	4.756257403	4.282691135	4.713366976
ENSMUSG00000056054.9	S100a8	0.554991	3.428716	2.801342	0.013599	0.127169	-3.20077	3.199991702	2.65759125	3.52134564	3.872223421	3.520070246	3.801075306
ENSMUSG00000060019.5	Gm10073	0.559954	7.099599	3.928261	0.001383	0.034436	-1.29613	6.901684847	6.694751667	6.86206259	7.474995199	7.021114393	7.642986343
ENSMUSG00000028958.14	Tmub1	0.560759	3.480094	3.395942	0.004077	0.064099	-2.06953	3.133265588	3.13096206	3.32483586	3.800073636	3.565041005	3.926387978
ENSMUSG00000062647.16	Rpl7a	0.568017	5.702673	3.201594	0.006055	0.081173	-2.74659	5.307925399	5.424361357	5.51999417	6.100468569	5.478557922	6.384728051
ENSMUSG00000097567.1	Gm26637	0.571627	2.047937	2.322084	0.034981	0.200218	-3.76224	2.141777088	1.462943819	1.6894684	2.164925378	2.453862094	2.374646897
ENSMUSG00000067038.5	Rps12-ps3	0.577751	5.565251	3.240647	0.005593	0.077935	-2.66339	5.554160134	5.097795196	5.17317671	5.993103997	5.39176828	6.18149905
ENSMUSG00000026497.7	Mixl1	0.578055	2.615812	2.488264	0.025318	0.174569	-3.59746	2.446631669	2.199909414	2.37951295	2.620870821	2.767797399	3.276367379
ENSMUSG00000056071.12	S100a9	0.580403	2.813625	2.93335	0.010427	0.109061	-2.82318	2.208119583	2.478885363	2.844354	3.145646315	3.13889155	3.065854204
ENSMUSG00000083626.1	Gm4459	0.581544	2.727048	2.905761	0.011024	0.112165	-2.85733	2.651249389	2.037179913	2.57959078	3.038233373	3.02547857	3.030557152
ENSMUSG00000102644.5	Thap6	0.584865	2.182791	2.475807	0.025944	0.176177	-3.52428	1.840910608	1.525679575	2.25506558	2.442778498	2.485397668	2.546915839
ENSMUSG00000059970.6	Hspa2	0.587363	2.244541	2.45299	0.027128	0.179328	-3.57666	2.141777088	1.462943819	2.2021167	2.54441876	2.437831985	2.678160372
ENSMUSG00000057657.3	Rps18-ps3	0.59008	2.64542	2.383036	0.031089	0.189975	-3.78891	2.577876203	2.380481659	2.14715024	2.90698884	2.546473245	3.313552299
ENSMUSG00000100078.1	Gm6170	0.602295	2.872313	2.13586	0.049893	0.222753	-4.26091	2.851327224	2.037179913	2.79156659	3.281384354	2.661375339	3.611046176
ENSMUSG00000066684.10	Pilrb1	0.608276	1.873004	2.326764	0.034667	0.199707	-3.71628	1.404811494	1.256492942	1.99980852	2.086213402	2.248636956	2.242061257
ENSMUSG00000103439.1	Gm7019	0.623898	3.160673	3.581055	0.002797	0.05121	-1.6471	2.765809153	2.739275048	3.02289214	3.509653342	3.333394574	3.593012253
ENSMUSG00000104126.1	Gm37486	0.626134	2.913938	2.249636	0.040203	0.208938	-4.07605	2.473853138	2.160915282	3.09727433	3.448034224	2.728803267	3.574750047
ENSMUSG00000042992.15	Borcs5	0.632382	1.732572	2.369032	0.031945	0.19275	-3.61915	1.840910608	1.10037374	1.29353973	2.058991933	2.09356756	2.008049752
ENSMUSG00000084104.1	Gm13578	0.65054	3.425942	2.511304	0.024198	0.170977	-3.73401	3.045269107	3.004513725	3.24855227	3.864382668	3.168335328	4.224597351
ENSMUSG00000024205.7	Rpl36-ps2	0.654421	2.596928	3.22663	0.005755	0.079202	-2.22727	2.208119583	2.07961518	2.47187697	3.079456036	2.842759456	2.899737941
ENSMUSG00000036835.6	Psenen	0.664218	4.020099	3.274226	0.005224	0.07505	-2.404	3.771305141	3.190259244	4.04936435	4.341925896	4.091007319	4.676734079
ENSMUSG00000055148.7	Klf2	0.668006	1.552755	2.353045	0.03295	0.19504	-3.61499	1.513745865	1.180544089	1.01523856	1.719785847	1.77419517	2.113019312
ENSMUSG00000078480.3	Mrpl48-ps	0.672518	1.506491	2.27337	0.038416	0.206263	-3.7373	1.158650906	1.256492942	1.13307505	1.820479846	1.523871816	2.146377909
ENSMUSG00000091058.1	Gm17538	0.673597	1.337011	2.231544	0.041616	0.211612	-3.77616	0.941839517	0.829071718	1.24200942	1.367114228	1.848833971	1.79319888
ENSMUSG00000032845.15	Alpk2	0.67924	1.681914	2.515271	0.02401	0.170562	-3.36109	1.158650906	1.10037374	1.72743625	1.974103035	2.05204829	2.07887109
ENSMUSG00000025911.14	Adhfe1	0.692911	1.569107	2.347541	0.033303	0.196152	-3.62578	1.017788371	1.256492942	1.43792963	1.611532956	1.848833971	2.242061257
ENSMUSG00000073274.4	Gm14636	0.705226	1.469461	2.364781	0.03221	0.193215	-3.58018	1.565276166	0.725978225	1.07535956	1.611532956	1.848833971	1.989787546
ENSMUSG00000104923.1	Gm5865	0.724297	2.208991	3.164655	0.006527	0.08485	-2.28138	1.922210711	1.901828061	1.76443046	2.378169417	2.531445035	2.755862729
ENSMUSG00000101249.1	Gm29216	0.724443	3.049594	2.199868	0.044203	0.214663	-4.19034	2.552565114	2.629302206	2.87850223	3.616209443	2.633496307	3.98748843
ENSMUSG00000068200.6	Gm5931	0.72657	1.76286	2.869094	0.011869	0.11754	-2.7545	1.460306606	1.180544089	1.5267389	2.190236466	2.07295728	2.146377909
ENSMUSG00000107950.1	Gm6375	0.733626	1.601368	2.723097	0.015904	0.139769	-2.99049	1.017788371	1.397355478	1.29353973	2.031246943	1.824380927	2.043894951
ENSMUSG00000094344.1	Gm11942	0.743874	3.006854	2.165762	0.047155	0.219041	-4.23707	2.418886679	2.685336241	2.80937787	3.587920399	2.561346521	3.97825515
ENSMUSG00000106917.1	Gm7832	0.757553	4.421179	3.530344	0.003101	0.054188	-1.96321	4.187713372	3.954084298	3.98028596	5.057273416	4.223142887	5.124573539
ENSMUSG00000081010.3	Gm13880	0.788044	1.487517	2.2668	0.038903	0.207284	-3.73953	1.089938156	0.829071718	1.34329276	2.031246943	1.329055639	2.302497111
ENSMUSG00000035560.4	Wdr20rt	0.790733	1.001877	2.344217	0.033517	0.196492	-3.54806	0.776780271	1.015484842	0.1045059	1.367114228	1.257796956	1.489582663
ENSMUSG00000049517.7	Rps23	0.792697	1.540527	2.621539	0.019466	0.154745	-3.15461	1.224239248	0.614946913	1.5267389	2.058991933	1.722200532	2.096046233
ENSMUSG00000091223.2	Gm8775	0.80563	2.834391	3.418469	0.003895	0.062194	-1.89713	2.674900657	1.993458536	2.60020106	3.269558919	2.937009782	3.531217152
ENSMUSG00000052560.15	Cpne8	0.806681	1.459356	2.406038	0.02973	0.186632	-3.50528	1.017788371	0.829071718	1.24200942	2.355973671	1.396959703	1.914335318
ENSMUSG00000050270.12	Tmem220	0.820979	2.571502	3.557852	0.002932	0.052819	-1.59032	2.0361669	1.853733772	2.55868179	2.779374643	3.20668164	2.994374816
ENSMUSG00000057863.5	Rpl36	0.827996	4.981753	3.069545	0.007917	0.093916	-2.94549	4.456900005	4.458930757	4.7667114	5.799045709	4.555786996	5.853145247
ENSMUSG00000076613.3	Ighg2b	0.842876	0.942417	2.386437	0.030885	0.189242	-3.47231	1.017788371	0.058553564	0.50504383	1.17763643	1.257796956	1.637681302
ENSMUSG00000082491.1	Gm5909	0.847318	1.588132	3.109094	0.007307	0.090282	-2.30602	1.017788371	1.015484842	1.43792963	1.974103035	1.987439209	2.096046233
ENSMUSG00000049285.4	Mblac1	0.863286	0.498206	2.299386	0.036544	0.202683	-3.56638	-0.360723253	0.363408146	0.21553721	0.899335267	0.836659257	1.0350168
ENSMUSG00000080002.2	Gm5566	0.878986	1.120063	2.196513	0.044485	0.214943	-3.79048	-0.180151007	0.363408146	1.76443046	1.494493535	1.640516735	1.637681302
ENSMUSG00000066755.2	Tnfsf18	0.893921	0.930699	2.556541	0.022136	0.164159	-3.1958	0.776780271	-0.122018681	0.74605193	1.275483753	1.583372827	1.324523417
ENSMUSG00000094989.1	Rpl9-ps4	0.899553	2.378202	3.189925	0.006201	0.082246	-2.25435	1.922210711	2.237877264	1.6894684	2.90698884	2.3377205	3.174947061
ENSMUSG00000082895.1	Rpsa-ps9	0.903992	1.318296	2.518987	0.023835	0.170191	-3.2975	0.124703574	1.180544089	1.29353973	1.852541056	1.396959703	2.061489011
ENSMUSG00000082028.1	Gm1826	0.919036	0.109129	2.167482	0.047002	0.219041	-3.73251	-0.180151007	0.058553564	-0.83891057	0.389140535	0.508605059	0.71753461
ENSMUSG00000020234.10	4930404N11R	0.928457	0.927946	2.715977	0.016132	0.140566	-2.93404	0.255948108	0.494652679	0.58993273	1.719785847	1.182834898	1.324523417
ENSMUSG00000099241.1	Gm18852	0.961479	0.738003	2.738711	0.015416	0.13736	-2.88782	0.487273654	0.058553564	0.21553721	1.322026339	1.020105398	1.324523417
ENSMUSG00000066637.1	Ttc32	0.97591	0.54684	2.491669	0.025149	0.173779	-3.27335	0.487273654	0.058553564	-0.29142278	0.836599512	0.836659257	1.35337828
ENSMUSG00000090186.5	Gm7592	1.081793	0.61003	2.81568	0.013214	0.125576	-2.75998	0.686582462	-0.328469559	-0.14703287	1.17763643	0.97638402	1.295079639
ENSMUSG00000085213.1	Gm13091	1.083877	0.080551	2.223384	0.042269	0.212176	-3.65001	-1.097688847	0.494652679	-0.63245969	0.202727411	0.445869304	1.070206228
ENSMUSG00000058838.9	Rps27a-ps2	1.245321	0.680399	2.475126	0.025978	0.17624	-3.30297	-0.180151007	0.494652679	-0.14703287	1.944659258	0.380280962	1.58998456
ENSMUSG00000061167.9	Rpl15-ps3	1.301726	0.101717	2.715976	0.016132	0.140566	-2.92341	-0.180151007	-1.221554355	-0.29142278	0.95945626	0.239418426	1.104557733
ENSMUSG00000073234.3	Gm8773	1.375776	-0.43167	2.764442	0.014643	0.133032	-2.87363	-1.945685753	-0.328469559	-1.07991867	0.202727411	0.311568212	0.249755649
ENSMUSG00000057160.2	Gm16372	1.407012	0.798983	3.166678	0.0065	0.084626	-2.1861	-0.019686335	-0.858984276	1.01523856	1.852541056	1.143840766	1.660951082
ENSMUSG00000036944.5	Tmem71	1.428583	0.418737	3.014478	0.008851	0.099056	-2.44984	0.376242341	-1.706981182	0.31863071	1.126106129	1.29386621	1.104557733
ENSMUSG00000097166.1	9330179D12R	1.559933	-0.68258	2.332375	0.034293	0.198803	-3.48673	-2.682651348	-2.443946776	0.74605193	0.099633918	-0.001589673	0.187019893
ENSMUSG00000067058.7	Rps15a-ps5	1.570705	2.793014	2.542511	0.022757	0.166062	-3.4807	1.286975003	2.600447343	2.14715024	4.230442789	2.192420947	4.300646315
ENSMUSG00000020599.13	Rgs9	2.376784	4.830973	3.705094	0.002174	0.043866	-1.55157	2.552565114	1.901828061	6.14452158	6.638792729	6.160420121	5.587709614
