# Supplementary material for: Critical role of the BAF chromatin remodeling complex during murine neural crest development
Source: PLoS Genet. 2021 Mar 22;17(3):e1009446. doi: 10.1371/journal.pgen.1009446 (PMC8016319; doi:10.1371/journal.pgen.1009446)
Supplement: S2 Table — (PDF) [file pgen.1009446.s012.pdf]

Supplemental table 2. Transcription factor enrichment analysis using RNAseq data set

TF	status
SOX2	common_enrichr_icis_pscan
E2F7	common_enrichr_icis_pscan
E2F4	common_enrichr_icis_pscan
TFAP2C	common_enrichr_pscan
CTCF	common_enrichr_pscan
E2F1	common_enrichr_pscan
EGR1	common_enrichr_pscan
MYC	common_enrichr_pscan
AHR	common_enrichr_pscan
ARNT	common_enrichr_pscan
NFYA	common_enrichr_pscan
ZNF423	common_enrichr_pscan
NHLH1	common_enrichr_pscan
INSM1	common_enrichr_pscan
ZIC1	common_enrichr_pscan
SP8	common_enrichr_pscan
GLIS2	common_enrichr_pscan
HEY1	common_enrichr_pscan
ZIC4	common_enrichr_pscan
GLIS1	common_enrichr_pscan
ZIC3	common_enrichr_pscan
ASCL1	common_enrichr_pscan
MYCN	common_enrichr_pscan
GLIS3	common_enrichr_pscan
TFAP2B	common_enrichr_pscan
HES5	common_enrichr_pscan
HIF1A	common_enrichr_pscan
HEY2	common_enrichr_pscan
HIC2	common_enrichr_pscan
E2F8	common_enrichr_pscan
SP4	common_enrichr_pscan
TFAP2A	common_enrichr_pscan
ZFX	common_enrichr_pscan
KLF9	common_enrichr_pscan
NR2C2	common_enrichr_pscan
NFKB1	common_enrichr_pscan
EGR2	common_enrichr_pscan
KLF5	common_enrichr_pscan
EGR3	common_enrichr_pscan
WT1	common_enrichr_icis
ZFP281	common_enrichr_icis
SOX9	common_enrichr_icis
SOX3	common_enrichr_icis
SRY	common_enrichr_icis
SOX17	common_enrichr_icis
PBX1	common_enrichr_icis
SOX12	common_enrichr_icis
SOX1	common_enrichr_icis
PBX3	common_enrichr_icis
SOX5	common_enrichr_icis
HMGA2	common_enrichr_icis
SOX11	common_enrichr_icis
SOX4	common_enrichr_icis
SOX21	common_enrichr_icis
ZFP1	common_enrichr_icis
ZFP37	common_enrichr_icis
SOX13	common_enrichr_icis
NFIB	common_enrichr_icis
TCF7L1	common_enrichr_icis
SOX8	common_enrichr_icis
SOX6	common_enrichr_icis
HMGB2	common_enrichr_icis
TCF7L2	common_enrichr_icis
SOX10	common_enrichr_icis
BCL6	common_enrichr_icis
MZF1	common_icis_pscan
EP300	uniq_enrichr
SUZ12	uniq_enrichr

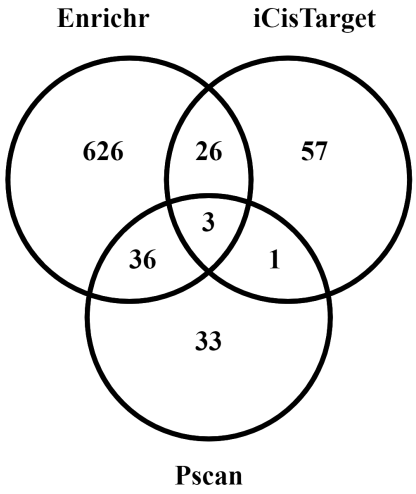

OLIG2	uniq_enrichr
RNF2	uniq_enrichr
RING1B	uniq_enrichr
MTF2	uniq_enrichr
EZH2	uniq_enrichr
ISL1	uniq_enrichr
DMRT1	uniq_enrichr
YAP1	uniq_enrichr
POU5F1	uniq_enrichr
ZNF217	uniq_enrichr
SMAD3	uniq_enrichr
KDM2B	uniq_enrichr
FOXM1	uniq_enrichr
LMO2	uniq_enrichr
SMAD4	uniq_enrichr
PAX3-FKH	uniq_enrichr
FOXA2	uniq_enrichr
JARID2	uniq_enrichr
PHC1	uniq_enrichr
TCF3	uniq_enrichr
SALL4	uniq_enrichr
SA1	uniq_enrichr
TAL1	uniq_enrichr
TP53	uniq_enrichr
CEBPD	uniq_enrichr
EED	uniq_enrichr
KLF4	uniq_enrichr
GF1	uniq_enrichr
POU3F1	uniq_enrichr
P300	uniq_enrichr
AR	uniq_enrichr
STAT3	uniq_enrichr
CBX2	uniq_enrichr
SMARCA4	uniq_enrichr
NANOG	uniq_enrichr
SMARCD1	uniq_enrichr
TCF4	uniq_enrichr
NFE2L2	uniq_enrichr
NRF2	uniq_enrichr
RUNX2	uniq_enrichr
SMC1	uniq_enrichr
GF1B	uniq_enrichr
TEAD4	uniq_enrichr
KDM5B	uniq_enrichr
REST	uniq_enrichr
PRDM14	uniq_enrichr
TCFCP2L1	uniq_enrichr
TCF3/E2A	uniq_enrichr
MEIS1	uniq_enrichr
SMAD2/3	uniq_enrichr
UBF1/2	uniq_enrichr
Oct-04	uniq_enrichr
BMI1	uniq_enrichr
RCOR2	uniq_enrichr
NR4A2	uniq_enrichr
MITF	uniq_enrichr
TBX20	uniq_enrichr
CEBPB	uniq_enrichr
RCOR3	uniq_enrichr
CTBP1	uniq_enrichr
MNX1	uniq_enrichr
CREB1	uniq_enrichr
BRD4	uniq_enrichr
RUNX1	uniq_enrichr
ESR1	uniq_enrichr
RARB	uniq_enrichr
P53	uniq_enrichr
TOP2B	uniq_enrichr
PBX	uniq_enrichr

CTNNB1	uniq_enrichr
HNF4A	uniq_enrichr
NACC1	uniq_enrichr
EBF1	uniq_enrichr
TP63	uniq_enrichr
DROSHA	uniq_enrichr
BACH1	uniq_enrichr
PPARG	uniq_enrichr
CMYC	uniq_enrichr
KAP1	uniq_enrichr
TET1	uniq_enrichr
CCND1	uniq_enrichr
IKZF1	uniq_enrichr
PPARD	uniq_enrichr
ZFP57	uniq_enrichr
ERA	uniq_enrichr
NFI	uniq_enrichr
PIAS1	uniq_enrichr
TBX3	uniq_enrichr
NUCKS1	uniq_enrichr
SMC3	uniq_enrichr
ESR2	uniq_enrichr
CLOCK	uniq_enrichr
NR3C1	uniq_enrichr
CTBP2	uniq_enrichr
VDR	uniq_enrichr
DPY	uniq_enrichr
RARG	uniq_enrichr
FUS	uniq_enrichr
SETDB1	uniq_enrichr
MEF2A	uniq_enrichr
ASH2L	uniq_enrichr
CDX2	uniq_enrichr
TBL1	uniq_enrichr
FOXP2	uniq_enrichr
GATA1	uniq_enrichr
ZBTB7A	uniq_enrichr
ZNF532	uniq_enrichr
ZNF608	uniq_enrichr
POU3F2	uniq_enrichr
KIAA1549	uniq_enrichr
POU3F3	uniq_enrichr
NR2F1	uniq_enrichr
PLXNA2	uniq_enrichr
NEUROD4	uniq_enrichr
EBF3	uniq_enrichr
MYT1	uniq_enrichr
ZNF704	uniq_enrichr
DACH1	uniq_enrichr
CHD7	uniq_enrichr
SETBP1	uniq_enrichr
DZIP1	uniq_enrichr
IRX1	uniq_enrichr
ZKSCAN2	uniq_enrichr
NHLH2	uniq_enrichr
MEIS3	uniq_enrichr
ZNF462	uniq_enrichr
ZNF521	uniq_enrichr
NEUROG	uniq_enrichr
ZNF536	uniq_enrichr
PLXNA4	uniq_enrichr
ZNF618	uniq_enrichr
CUX2	uniq_enrichr
ONECUT2	uniq_enrichr
GLI2	uniq_enrichr
FOXG1	uniq_enrichr
DPF1	uniq_enrichr
DBX1	uniq_enrichr
GBX2	uniq_enrichr

NPAS3	uniq_enrichr
SALL1	uniq_enrichr
TUB	uniq_enrichr
FGD1	uniq_enrichr
LHX9	uniq_enrichr
POU3F4	uniq_enrichr
ZNF827	uniq_enrichr
NFATC4	uniq_enrichr
PATZ1	uniq_enrichr
SMARCA1	uniq_enrichr
POU4F1	uniq_enrichr
SLC26A1(	uniq_enrichr
LHX5	uniq_enrichr
ZFHX4	uniq_enrichr
ZSCAN18	uniq_enrichr
ZNF362	uniq_enrichr
MEIS2	uniq_enrichr
MYT1L	uniq_enrichr
SCRT2	uniq_enrichr
SALL2	uniq_enrichr
TBR1	uniq_enrichr
ZC3H13	uniq_enrichr
ZBTB46	uniq_enrichr
EN2	uniq_enrichr
ZNF821	uniq_enrichr
POU2F1	uniq_enrichr
ST18	uniq_enrichr
SCRT1	uniq_enrichr
KLF7	uniq_enrichr
ZFHX2	uniq_enrichr
EBF4	uniq_enrichr
PKNOX2	uniq_enrichr
ID4	uniq_enrichr
TWIST1	uniq_enrichr
LHX1	uniq_enrichr
ZNF300	uniq_enrichr
OTP	uniq_enrichr
SORBS2	uniq_enrichr
TEAD2	uniq_enrichr
TBX5	uniq_enrichr
TBX18	uniq_enrichr
ZNF248	uniq_enrichr
TOX3	uniq_enrichr
PBRM1	uniq_enrichr
SNAI2	uniq_enrichr
HOXB8	uniq_enrichr
BARHL1	uniq_enrichr
PAX3	uniq_enrichr
ZNF804A	uniq_enrichr
NEUROG:	uniq_enrichr
RFX3	uniq_enrichr
TCF12	uniq_enrichr
BARHL2	uniq_enrichr
ZNF25	uniq_enrichr
ZNF91	uniq_enrichr
NR2F2	uniq_enrichr
TEAD1	uniq_enrichr
MKX	uniq_enrichr
ZNF219	uniq_enrichr
ZFP90	uniq_enrichr
SALL3	uniq_enrichr
CAMTA1	uniq_enrichr
ZNF84	uniq_enrichr
OTX1	uniq_enrichr
UNCX	uniq_enrichr
MEOX2	uniq_enrichr
PRRX1	uniq_enrichr
NFIA	uniq_enrichr
LCORL	uniq_enrichr

ZNF436	uniq_enrichr
ZNF74	uniq_enrichr
AHDC1	uniq_enrichr
FBN1	uniq_enrichr
ALX4	uniq_enrichr
ZEB1	uniq_enrichr
PRRX2	uniq_enrichr
FOXF1	uniq_enrichr
ZNF711	uniq_enrichr
ZMAT3	uniq_enrichr
TERF2IP	uniq_enrichr
HSF2	uniq_enrichr
GLI3	uniq_enrichr
HAND2	uniq_enrichr
TEAD3	uniq_enrichr
PHOX2B	uniq_enrichr
TSHZ3	uniq_enrichr
PHOX2A	uniq_enrichr
EN1	uniq_enrichr
OSR2	uniq_enrichr
FEZF1	uniq_enrichr
POGZ	uniq_enrichr
DACH2	uniq_enrichr
CREB3L1	uniq_enrichr
RORB	uniq_enrichr
ZNF606	uniq_enrichr
ZSCAN23	uniq_enrichr
HES3	uniq_enrichr
POU4F2	uniq_enrichr
ZNF48	uniq_enrichr
ZNF197	uniq_enrichr
BNC2	uniq_enrichr
FOXL1	uniq_enrichr
LHX2	uniq_enrichr
HAND1	uniq_enrichr
TCF21	uniq_enrichr
IRX2	uniq_enrichr
ZKSCAN1	uniq_enrichr
VEZF1	uniq_enrichr
DMRTA2	uniq_enrichr
ZNF627	uniq_enrichr
ZMAT4	uniq_enrichr
ATOH8	uniq_enrichr
FAM171B	uniq_enrichr
PLXNB1	uniq_enrichr
ETV1	uniq_enrichr
HOXD8	uniq_enrichr
OSR1	uniq_enrichr
PLXNA1	uniq_enrichr
PLXNA3	uniq_enrichr
CSDC2	uniq_enrichr
ATF2	uniq_enrichr
NKX6-2	uniq_enrichr
RBPJ	uniq_enrichr
ZNF260	uniq_enrichr
ZSCAN10	uniq_enrichr
OLIG3	uniq_enrichr
FMNL2	uniq_enrichr
TOX	uniq_enrichr
ARX	uniq_enrichr
ZNF254	uniq_enrichr
FOXC2	uniq_enrichr
ZNF334	uniq_enrichr
PAWR	uniq_enrichr
THAP2	uniq_enrichr
WDHD1	uniq_enrichr
LARP6	uniq_enrichr
SATB2	uniq_enrichr
SMAD9	uniq_enrichr

ZFHX3	uniq_enrichr
ADNP2	uniq_enrichr
ZIC5	uniq_enrichr
ZNF117	uniq_enrichr
ZNF404	uniq_enrichr
ZNF678	uniq_enrichr
ZNF428	uniq_enrichr
CARHSP1	uniq_enrichr
TRIM3	uniq_enrichr
SMARCA5	uniq_enrichr
MKRN3	uniq_enrichr
KNTC1	uniq_enrichr
SLC39A10	uniq_enrichr
ZIC2	uniq_enrichr
ZNF10	uniq_enrichr
ARNT2	uniq_enrichr
ZNF496	uniq_enrichr
SMARCC2	uniq_enrichr
GATAD2B	uniq_enrichr
SMARCC1	uniq_enrichr
NFIX	uniq_enrichr
HOXC8	uniq_enrichr
TSC22D1	uniq_enrichr
DLX2	uniq_enrichr
TBX2	uniq_enrichr
ZNF644	uniq_enrichr
DLX1	uniq_enrichr
FOXH1	uniq_enrichr
ZNF629	uniq_enrichr
ZSCAN29	uniq_enrichr
IRX5	uniq_enrichr
SMARCE1	uniq_enrichr
HOXB5	uniq_enrichr
EOMES	uniq_enrichr
NEUROD6	uniq_enrichr
ZHX3	uniq_enrichr
POU6F1	uniq_enrichr
EMX1	uniq_enrichr
ZNF540	uniq_enrichr
ZNF518B	uniq_enrichr
MTA3	uniq_enrichr
ADAMTS1	uniq_enrichr
TLX2	uniq_enrichr
KLF12	uniq_enrichr
THRA	uniq_enrichr
ZSCAN1	uniq_enrichr
ZNF589	uniq_enrichr
ZNF292	uniq_enrichr
YEATS2	uniq_enrichr
DEPDC1	uniq_enrichr
HMGB3	uniq_enrichr
TRIM23	uniq_enrichr
RAPGEF5	uniq_enrichr
LIN28B	uniq_enrichr
PLXNB3	uniq_enrichr
DLX5	uniq_enrichr
ID3	uniq_enrichr
ADNP	uniq_enrichr
FOXF2	uniq_enrichr
HDX	uniq_enrichr
ZNF649	uniq_enrichr
TGIF2	uniq_enrichr
DEK	uniq_enrichr
ZDHHC11	uniq_enrichr
TLX3	uniq_enrichr
ETV5	uniq_enrichr
CREB3L2	uniq_enrichr
ZNF398	uniq_enrichr
ZNF195	uniq_enrichr

FOXN4	uniq_enrichr
LHX8	uniq_enrichr
INSM2	uniq_enrichr
ZBTB41	uniq_enrichr
ZNF354C	uniq_enrichr
ZFP28	uniq_enrichr
ZNF781	uniq_enrichr
SSH1	uniq_enrichr
LGR4	uniq_enrichr
OLIG1	uniq_enrichr
LCOR	uniq_enrichr
ZNF449	uniq_enrichr
ZNF253	uniq_enrichr
DEPDC1B	uniq_enrichr
RGS11	uniq_enrichr
ZNF738	uniq_enrichr
RGS7	uniq_enrichr
HOXA11	uniq_enrichr
DLX6	uniq_enrichr
PAX7	uniq_enrichr
DMRT3	uniq_enrichr
MSX2	uniq_enrichr
VAX1	uniq_enrichr
SIX5	uniq_enrichr
ZNF92	uniq_enrichr
ZNF177	uniq_enrichr
ZNF33A	uniq_enrichr
ZNF418	uniq_enrichr
ZNF251	uniq_enrichr
ZBTB12	uniq_enrichr
AFF3	uniq_enrichr
NPAS4	uniq_enrichr
AFF4	uniq_enrichr
SMAD6	uniq_enrichr
CUL4B	uniq_enrichr
E2F2	uniq_enrichr
SMAD5	uniq_enrichr
HOXD3	uniq_enrichr
E2F5	uniq_enrichr
LMX1A	uniq_enrichr
OTX2	uniq_enrichr
ZNF146	uniq_enrichr
HOXD4	uniq_enrichr
DEAF1	uniq_enrichr
ZBED4	uniq_enrichr
FOXP4	uniq_enrichr
ZNF714	uniq_enrichr
ZNF488	uniq_enrichr
ZNF483	uniq_enrichr
ZNF664	uniq_enrichr
LHX6	uniq_enrichr
WHSC1	uniq_enrichr
PAX8	uniq_enrichr
LMX1B	uniq_enrichr
ZNF697	uniq_enrichr
ZNF503	uniq_enrichr
ZFP42	uniq_enrichr
ZNF358	uniq_enrichr
ZBTB10	uniq_enrichr
THAP10	uniq_enrichr
ZCCHC11	uniq_enrichr
ZNF770	uniq_enrichr
PDS5B	uniq_enrichr
HIST1H1E	uniq_enrichr
EPAS1	uniq_enrichr
NR2C1	uniq_enrichr
HOXD9	uniq_enrichr
NKX2-1	uniq_enrichr
NR6A1	uniq_enrichr

ZNF281	uniq_enrichr
HEYL	uniq_enrichr
TEF	uniq_enrichr
SHOX2	uniq_enrichr
ZNF160	uniq_enrichr
ESRRG	uniq_enrichr
HOXC6	uniq_enrichr
PRDM12	uniq_enrichr
ZNF365	uniq_enrichr
ZNF385B	uniq_enrichr
ZNF680	uniq_enrichr
ZNF607	uniq_enrichr
ZNF367	uniq_enrichr
BAZ2B	uniq_enrichr
SLC4A10	uniq_enrichr
HOXB3	uniq_enrichr
BCL6B	uniq_enrichr
ZFPM2	uniq_enrichr
NPAS2	uniq_enrichr
ZNF708	uniq_enrichr
FOXD3	uniq_enrichr
ZNF207	uniq_enrichr
BCL11A	uniq_enrichr
ZNF287	uniq_enrichr
ZNF385D	uniq_enrichr
PRDM8	uniq_enrichr
ZSCAN12	uniq_enrichr
ZNF226	uniq_enrichr
ZNF454	uniq_enrichr
ZNF286A	uniq_enrichr
ZNF69	uniq_enrichr
TERF1	uniq_enrichr
RAPGEF4	uniq_enrichr
ZC3H7B	uniq_enrichr
ARID2	uniq_enrichr
NFE2L3	uniq_enrichr
HOXD10	uniq_enrichr
NKX6-1	uniq_enrichr
ZBTB4	uniq_enrichr
ZNF551	uniq_enrichr
ZBTB39	uniq_enrichr
FOXS1	uniq_enrichr
ZFP82	uniq_enrichr
ZNF138	uniq_enrichr
FEZF2	uniq_enrichr
CASP8AP[illegible]	uniq_enrichr
FBXO41	uniq_enrichr
POU2F2	uniq_enrichr
SIX4	uniq_enrichr
HOXA13	uniq_enrichr
IRX4	uniq_enrichr
RAX	uniq_enrichr
ZNF12	uniq_enrichr
ZHX1	uniq_enrichr
SOHLH1	uniq_enrichr
ZNF35	uniq_enrichr
HIC1	uniq_enrichr
IRX3	uniq_enrichr
ZNF43	uniq_enrichr
HLF	uniq_enrichr
CUX1	uniq_enrichr
ZNF415	uniq_enrichr
DMRTA1	uniq_enrichr
TSHZ1	uniq_enrichr
DBX2	uniq_enrichr
HES4	uniq_enrichr
ZNF460	uniq_enrichr
ZNF579	uniq_enrichr
ARID5B	uniq_enrichr

TOX2	uniq_enrichr
NFXL1	uniq_enrichr
ZNF638	uniq_enrichr
RBM26	uniq_enrichr
CHD9	uniq_enrichr
HIST1H1C	uniq_enrichr
CEBPZ	uniq_enrichr
RBPJL	uniq_enrichr
NFIC	uniq_enrichr
ZEB2	uniq_enrichr
ZNF280C	uniq_enrichr
ZNF93	uniq_enrichr
ZNF302	uniq_enrichr
RLF	uniq_enrichr
IKZF4	uniq_enrichr
PRDM16	uniq_enrichr
ZNF443	uniq_enrichr
ZNF107	uniq_enrichr
ZKSCAN5	uniq_enrichr
ZNF433	uniq_enrichr
FOXD4L6	uniq_enrichr
NOC3L	uniq_enrichr
ZNF720	uniq_enrichr
RAD51	uniq_enrichr
HP1BP3	uniq_enrichr
ATMIN	uniq_enrichr
HIST1H1E	uniq_enrichr
PRMT3	uniq_enrichr
ZNF391	uniq_enrichr
FOXK1	uniq_enrichr
ZNF775	uniq_enrichr
ZNF772	uniq_enrichr
PLEKHA4	uniq_enrichr
HIST1H1D	uniq_enrichr
HES6	uniq_enrichr
SF3A3	uniq_enrichr
GATA6	uniq_enrichr
PGR	uniq_enrichr
NRL	uniq_enrichr
ATF6	uniq_enrichr
FOXO1	uniq_enrichr
FLI1	uniq_enrichr
NEUROG3	uniq_enrichr
TRPS1	uniq_enrichr
EMX2	uniq_enrichr
ETV2	uniq_enrichr
ESRRB	uniq_enrichr
ZBTB20	uniq_enrichr
BCL11B	uniq_enrichr
ZNF750	uniq_enrichr
ZBTB48	uniq_enrichr
PITX2	uniq_enrichr
PAX6	uniq_enrichr
ARID3A	uniq_enrichr
PLAGL2	uniq_enrichr
MYOD1	uniq_enrichr
RORC	uniq_enrichr
LIN28A	uniq_enrichr
PIN1	uniq_enrichr
NKX25	uniq_enrichr
MSGN1	uniq_enrichr
ADAR	uniq_enrichr
CEBPG	uniq_enrichr
ONECUT1	uniq_enrichr
FOXQ1	uniq_enrichr
GATA4	uniq_enrichr
KMT2A	uniq_enrichr
ATF3	uniq_enrichr
PAX2	uniq_enrichr

IRX6	uniq_enrichr
OVOL1	uniq_enrichr
NEUROD'	uniq_enrichr
XBP1	uniq_enrichr
ATF4	uniq_enrichr
LIN28	uniq_enrichr
GRHL2	uniq_enrichr
JUND	uniq_enrichr
RELA	uniq_enrichr
MYRF	uniq_enrichr
STAT1	uniq_enrichr
RBM10	uniq_enrichr
ZNF143	uniq_enrichr
YBX1	uniq_enrichr
GATA2	uniq_enrichr
IRF6	uniq_enrichr
BACH2	uniq_enrichr
SRF	uniq_enrichr
ZNF142	uniq_enrichr
EP400	uniq_enrichr
RARA	uniq_enrichr
DNMT1	uniq_enrichr
OVOL2	uniq_enrichr
NCOA3	uniq_enrichr
MECP2	uniq_enrichr
HSF1	uniq_enrichr
STAT6	uniq_enrichr
EHF	uniq_enrichr
PCGF2	uniq_enrichr
DDIT3	uniq_enrichr
U2AF1	uniq_enrichr
ADARB1	uniq_enrichr
GATA3	uniq_enrichr
SPDEF	uniq_enrichr
GTF2I	uniq_enrichr
NR2E3	uniq_enrichr
HOXC13	uniq_enrichr
YY1	uniq_enrichr
JUN	uniq_enrichr
HOXB4	uniq_enrichr
ESRRA	uniq_enrichr
FOSL1	uniq_enrichr
TWIST2	uniq_enrichr
AIRE	uniq_enrichr
JUNB	uniq_enrichr
ZNF581	uniq_enrichr
IRF1	uniq_enrichr
MEF2D	uniq_enrichr
FOXP3	uniq_enrichr
ZXDC	uniq_enrichr
CEBPA	uniq_enrichr
BPTF	uniq_enrichr
GLI1	uniq_enrichr
ELF3	uniq_enrichr
AFF1	uniq_enrichr
DUX4	uniq_enrichr
FOXA1	uniq_enrichr
FOXO3	uniq_enrichr
RC3H1	uniq_enrichr
MYB	uniq_enrichr
DOT1L	uniq_enrichr
ATF6B	uniq_enrichr
CHD1	uniq_enrichr
ETS1	uniq_enrichr
PHF20	uniq_enrichr
NR5A2	uniq_enrichr
TOX4	uniq_enrichr
PCGF6	uniq_enrichr
FOXP1	uniq_enrichr

HNF4G	uniq_enrichr
SATB1	uniq_enrichr
NR1I3	uniq_enrichr
2610008E	uniq_icis
A430033K	uniq_icis
DOF1.8	uniq_icis
DOF3	uniq_icis
FANK1	uniq_icis
GM2004	uniq_icis
HBP1	uniq_icis
HMGA1	uniq_icis
HMGA1-R	uniq_icis
IRF2	uniq_icis
IRF7	uniq_icis
LEF1	uniq_icis
M0647	uniq_icis
M0659	uniq_icis
M1611	uniq_icis
MAFK	uniq_icis
NRSF	uniq_icis
OAF1	uniq_icis
ORC2	uniq_icis
PBX2	uniq_icis
PBX4	uniq_icis
POLR2A	uniq_icis
RBAK	uniq_icis
RBBP9	uniq_icis
ROX1	uniq_icis
SIP4	uniq_icis
SOX14	uniq_icis
SOX15	uniq_icis
SOX18	uniq_icis
SOX30	uniq_icis
SOX7	uniq_icis
SREBF1	uniq_icis
SSRP1	uniq_icis
SUT1	uniq_icis
TAF1	uniq_icis
TCF7	uniq_icis
TPI1	uniq_icis
UBTF	uniq_icis
URC2	uniq_icis
ZBTB14	uniq_icis
ZFP12	uniq_icis
ZFP157	uniq_icis
ZFP182	uniq_icis
ZFP248	uniq_icis
ZFP300	uniq_icis
ZFP316	uniq_icis
ZFP334	uniq_icis
ZFP382	uniq_icis
ZFP39	uniq_icis
ZFP599	uniq_icis
ZFP663	uniq_icis
ZFP68	uniq_icis
ZFP780B	uniq_icis
ZFP810	uniq_icis
ZFP9	uniq_icis
ZFP949	uniq_icis
ZFP976	uniq_icis
TFDP1	uniq_pscan
SP2	uniq_pscan
HINFP	uniq_pscan
SP1	uniq_pscan
TCFL5	uniq_pscan
E2F6	uniq_pscan
EGR4	uniq_pscan
ZBTB33	uniq_pscan
NRF1	uniq_pscan

ZNF740	uniq_pscan
ZNF263	uniq_pscan
KLF16	uniq_pscan
PLAG1	uniq_pscan
CENPB	uniq_pscan
CTCFL	uniq_pscan
SP3	uniq_pscan
HES1	uniq_pscan
ZBTB7B	uniq_pscan
KLF14	uniq_pscan
ZBTB7C	uniq_pscan
MXI1	uniq_pscan
GCM2	uniq_pscan
RREB1	uniq_pscan
NFKB2	uniq_pscan
GMEB1	uniq_pscan
HES7	uniq_pscan
GCM1	uniq_pscan
EWSR1-FI	uniq_pscan
NFYB	uniq_pscan
KLF1	uniq_pscan
THAP1	uniq_pscan
PAX5	uniq_pscan
MTF1	uniq_pscan
