# Supplementary material for: Critical role of the BAF chromatin remodeling complex during murine neural crest development
Source: PLoS Genet. 2021 Mar 22;17(3):e1009446. doi: 10.1371/journal.pgen.1009446 (PMC8016319; doi:10.1371/journal.pgen.1009446)
Supplement: Attachment — Submitted filename: 109566_1_rebuttal_1897015_qynnzs.pdf [file pgen.1009446.s013.pdf]

Reviewer #1

Summary:

The manuscript by Bin-Lin et al examines the role of BAF subunits 155 and 170 in development. Using several different Cre lines, they knockout BAF155/170 function in Pax3 (neural plate border, somites etc), Wnt1 (neural crest), Foxg1 (epithelium etc) positive cells showing that BAF function is required for craniofacial, artery and outflow track development. Analysis of cell death and proliferation suggest that both processes are defective, which is supported by transcriptome analysis of sorted neural crest cells in the double knockout and reduction of Notch and Yap signaling components. Finally, the authors show that the BAF complex is essential for Tead dependent transcription of target genes. While the data is interesting and nicely presented, there are some issues that should be corrected before publication. My comments are below:

Response: *We would like to thank the reviewer for his/her positive evaluation of our manuscript.*

Comment: It is unclear why the authors use multiple Cre lines. The use of Pax3-Cre is not well explained. What is the rationale for using this Cre vs the others? FoxG1-Cre also targets pharyngeal pouches, and head and face ectoderm. Were other craniofacial structures affected in mutants besides palatal formation? In addition, it seems like there is a focus on the palate formation and neural crest cell development but then also present artery and outflow track. While this data is interesting, it disrupts the flow of the manuscript and could be added to the supplemental data.

Response: *Our goal was to use both Pax3 and Wnt1 cre to delete BAF155 and BAF170 in the neural crest cells. Wnt1Cre is a neural crest-specific cre. However, Pax3 is also expressed in neural crest, in addition to other tissues such as somite's. BAF155/170 deletion using both cre lines gave similar phenotypes.*

We have only analysed palate phenotype in FoxG1^{Cre};BAF155$^{fl/fl}$ mice as our focus was on the pharyngeal arch arteries and cardiac out flow tract (OFT). We have reduced the palate phenotype description and added it to the supplemental data in the revised manuscript so it does not disrupt the flow of the manuscript.

Comment: Where are BAF subunits (BAF155/170) expressed in development? Are they specifically expressed in NC and NC-derived tissues? Does each subunit, 155 or 170 have the same or different expression patterns? This should be discussed in the introduction if not presented.

Response: *During embryonic development, both BAF155 and BAF170 are ubiquitously expressed (Nguyen et al., 2016). BAF155 is strongly expressed in proliferating stem/progenitor cells and weakly in differentiated cells (Ho et al., 2009; Tuoc et al., 2013; Yan et al., 2008). In contrast, BAF170 is weakly expressed in proliferating stem/progenitor cells and strongly in differentiated cells (Ho et al., 2009; Tuoc et al., 2013; Yan et al., 2008). Although low expression of BAF170 is detected in stem/progenitor cells and BAF155 in differentiated cells, this expression is necessary*

and sufficient for stabilising the BAF complex (Narayanan et al., 2015; Nguyen et al., 2016). We have discussed this in the introduction section of the revised manuscript.

Comment: Some of the data is not shown such as the palate data for single BAF170 mutants. This should be included, at least in supplemental data. In addition, the authors focus on E14.5 for palate development (but this data is not shown), but also important to look at E15.5 after the palatal shelf have fused. Were palates also assessed in the Wnt-Cre and FoxG1-Cre BAF170 animals and also normal? What about the double BAF155/170 FoxG1-Cre? Do BAF155/BAF170 compensate for each other? Do their expression patterns change in the mutants?

Response: *We have not analysed the FoxG1-Cre BAF170 animals. However, in the revised manuscript, we have included palate data from E15.5 BAF170-deficient (Wnt1$^{Cre/+}$;BAF170$^{flox/flox}$;BAF155$^{flox/+}$) embryos and observed cleft palate (Supplemental Figure 2). All the palate analysis was performed at E15.5 as suggested by the reviewer (Supplemental Figure 2 and 3).*

We have not generated BAF155/170 FoxG1-Cre mice as focus of our study was neural crest. However, it has been demonstrated that the global deletion of BAF155/170 using CAG^{Cre} (induced at E9.5) leads to early embryonic lethality making it difficult to examine the palate defects.

To determine if they compensate each other, we have analyzed expression of BAF155 and BAF170 in BAF155-, BAF170- and BAF155/170-knockdown O9-1 cells but did not observed any significant changes at the RNA levels suggesting compensation. We can include these results in the revised manuscript if Editor suggest.

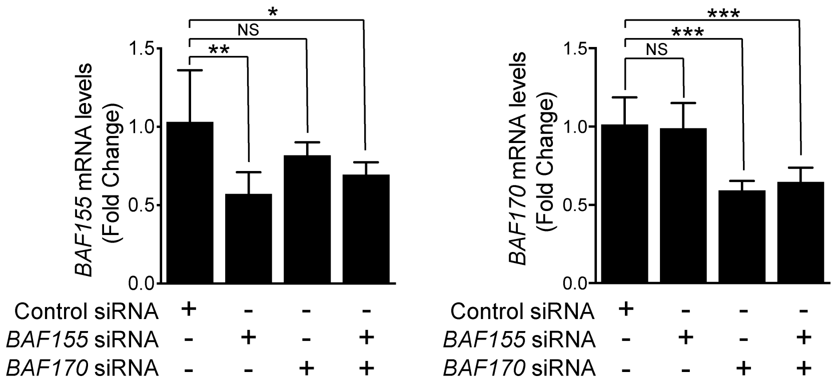

Comment: There is an issue with presentation of the "single" mutants as the data is in BAF155 fl/+;BAF170 fl/fl which is homozygous for 170 but heterozygous for 155. These cannot be considered single mutants as there may be phenotypes when one allele is reduced. This brings up the question if combinatorial heterozygote animals have phenotypes in any of the Cre mutants presented such as BAF155 fl/+;BAF170 fl/+? What is the degree of penetrance for all phenotypes analyzed, for example, hemorrhaging in the telencephalon?

Response: *We agree with the reviewer. In the revised manuscript, we have now presented the actual genotypes analyzed in each experiments. The combine heterozygous mice (Pax3$^{Cre/+}$;BAF155$^{fl/+}$;BAF170$^{fl/+}$ and Wnt1$^{Cre/+}$;BAF155$^{fl/+}$;BAF170$^{fl/+}$) are normal and fertile and show no phenotype. These mice were crossed to BAF155$^{fl/fl}$;BAF170$^{fl/fl}$*

mice to generate all the genotypes analysed in this study. We had included this information in the result section.

We have included the degree of penetrance for all phenotypes analyzed in the respective figure legend.

Comment: In general, all the analysis of the data presented needs to be better quantified. The gene expression and GFP signal in the lineage tracing is not quantified. In Figure 4, NCC migration to the developing heart was assessed by evaluating the number of neural crest in the conotruncal cushions of the OFT. In the text, this data was quantified but not shown. Please show this quantification. Additionally, what was quantified should be better described. BrdU pulse chase is a better assessment of proliferation rather than Ki67 staining. Is the number of Ki67 positive cells a reflection of the total number of cells per tissue area? What is the normalization of quantification?

Response: *We have quantified the distance migrated by the NCCs into the OFT and NCCs positive (GFP+) area within the OFT. These results have been included in the revised manuscript (Figure 4 E-F). Both Ki67 and BrdU are most commonly used markers to evaluate cell proliferation in embryonic tissues. Quantification of cell proliferation was calculated as the ratio of Ki67-positive cells to the total number of cells as determined by Dapi counterstaining in the defined area of the neural tube and pharyngeal arch. We have included these details in the results, figure legend as well as in the materials and methods section of the revised manuscript.*

Comment: For IP experiments, does single loss of either BAF subunit cause the same disruption of Brg1 with Tead? The phenotypes in the animal model of the BAF155 mutants were very similar to the BAF155/170 double mutants, only mildly more severe than the single mutant. This suggests BAF155 is the key subunit. Therefore, is the interaction between Brg1 of the BAF complex and Tead clearly dependent on BAF155 alone?

Response: *We agree with the reviewer that BAF155 is the key subunit. Literature suggest that BAF155 is strongly expressed in proliferating stem/progenitor cells and weakly in differentiated cells (Ho et al., 2009; Tuoc et al., 2013; Yan et al., 2008). In contrast, BAF170 is weakly expressed in proliferating stem/progenitor cells and strongly in differentiated cells (Ho et al., 2009; Tuoc et al., 2013; Yan et al., 2008). As we are deleting BAF155/170 in neural crest progenitors, BAF155 seems to play a more dominant role compared to BAF170. To determine the role of BAF155 in modulating the interaction between Brg1 and Tead, we have performed new IP experiments using BAF155 knockdown O9-1 cells. We see that with reduced levels of BAF155 alone, the interaction between Brg1 and Tead is impaired. We have included these results in Figure 8 of the revised manuscript.*

<u>Minor comments:</u>

Comment: Add references to single BAF subunit mutants in the introduction.

Response: *We have included more references to the introduction part of the revised manuscript.*

Comment: Sections in Figure 3I-L don't look consistent spatially across genotypes.

***Response:** We have revised the figure with additional images to make it consistent across all genotypes.*

Comment: If would be nice to add references to the previously published roll of BAF in chromatin modification and how that fits with this new story.

***Response:** We have included more references in the introduction section and discussed the data in context to the existing literature related to embryonic development and most specifically to neural crest cells.*

Significance:

This manuscript advances our understanding of the role of BAF 155/170 in development. It adds to the existing knowledge of the role in chromatin modification to direct transcriptional regulators. The audience is developmental biologists. Our expertise is in neural crest and craniofacial development.

***Response:** We would like to thank again the reviewer for his/her positive evaluation of our manuscript. We have addressed most of the recommendations this reviewer has provided.*

Reviewer #2

Summary:

In this manuscript, Bi-Lin and colleagues investigate the role of BAF chromatin remodeling complex in mouse NCC development. They deleted BAF155/BAF170 in NCCs using two different Cre-expressing mouse lineages. NCC-specific BAF155/BAF170 knockout leads to embryonic lethality and developmental defects involving craniofacial, pharyngeal arch artery, and OFT defects. RNAseq and transcription factor enrichment analysis revealed that the BAF complex modulates expression of multiple signaling pathway genes including Hippo and Notch. In vitro experiments demonstrated that BAF complex is essential for Brg1-Yap-Tead-dependent transcription activity in NCCs. The authors conclude that the BAF complex is important in modulating the gene regulatory network essential for neural crest development. Overall, this manuscript is interesting and data are well presented. However, the manuscript suffers from several flaws that reduce the impact of the findings as commented below.

***Response:** We would like to thank the reviewer for his/her positive evaluation of our manuscript.*

Specific comments:

Comment: In Figure 3M-P, the authors show a severe deficiency of SM22alpha+ cells in the vascular wall of the pharyngeal arteries in BAF155/170(Wnt1-CKO) or BAF155/170(Pax3-CKO)embryos and conclude that the BAF complex is essential for

mature smooth muscle differentiation from NCCs. The authors should indicate which pharyngeal arteries are shown and show that SM22alpha-negative NCCs are present around the arterial wall. The authors should also note that SM22alpha is not a "mature" smooth muscle marker, but is expressed in immature smooth muscle cells and myofibroblast-like cells. If SM22alpha-negative NCCs are surrounding the pharyngeal arch endothelium, it may suggest that an earlier stage than smooth muscle maturation may be disrupted.

Response: *In Figure 3M-P, 4th pharyngeal arch artery is shown. We have included this information in the figure legend. We have performed additional neural crest-lineage tracing experiments and performed GFP and SM22α double immunostaining to show SM22α^+ pharyngeal artery and GFP$^+$ NCCs around the arterial wall. We agree with the reviewer that SM22α is not a mature smooth muscle marker. Our goal was to show that NCCs differentiation into smooth muscle cells is affected due to BAF155/170 deletion. We did not intend to evaluate maturation of smooth muscle cells. Results from the lineage tracing experiments demonstrate SM22α negative NCCs surrounding the pharyngeal arch endothelium suggesting that the smooth muscle cells differentiation but not maturation was disrupted. We have included these results in the revised Figure 3M-P and modified the text to reflect.*

Comment: In Figure 4G, H, the authors show a decreased number of SM22alpha+ cells in the BAF155/170(Wnt1-CKO) conotruncal cushion. The authors should show whether these SM22alpha+ cells are NCCs or of non-NCC origin. If SM22alpha expression is impaired in BAF155/170-deficient NCCs, the latter is expected.

Response: *We observed decreased number of SM22α^+ cells in the BAF155/170-deficient conotruncal cushion (Figure 4I-J). This is consistent with the decreased number of migrating NCCs in the conotruncal cushion (Figure 4A-F). We have now quantified the NCCs (GFP$^+$) area in the conotruncal cushion and see severe reduction in BAF155/170 mutants. Consistently, reduced PlexinA2, a marker for cardiac neural crest, expression support the notion that there are very little NCCs present in the conotruncal cushion of the OFT. These results clearly suggest that the reduction in the number of SM22α^+ cells are due the reduced number of cardiac NCCs in the OFT. There are numerous papers demonstrating that the smooth muscle cells of the conotruncal cushion and pharyngeal arch arteries are derived from the neural crest cells (High et al., 2007; Kim et al., 2013; Majesky, 2007; Schussler et al., 2020; Singh et al., 2011).*

We can still perform GFP and SM22α double immunostaining to demonstrate their neural crest origin if suggested by the editor.

Comment: In Figure 5, the authors should evaluate proliferative and apoptotic cells in NCCs especially in the pharyngeal arches of control and BAF155/170(Wnt1-CKO) pharyngeal arches. Although the authors show increased Ki67+ and TUNEL+ cells in the knockout tissues, it is possible that BAF155/170-deficient NCCs may affect neighborhood cells in a non-cell autonomous manner.

Response: *Our RNAseq analysis in isolated NCCs clearly demonstrate that genes required for NCCs proliferation are severely downregulated (including Hippo signalling genes) and apoptotic genes are significantly upregulated suggesting that cellular*

changes observed in BAF155/170-deficient embryos are due to changes in a cell autonomous manner (Figure 6B).

Though we don't think it is essential in the light of RNAseq data on isolated NCCs, we can still perform GFP and Ki67 double staining and quantify the number of proliferating NCCs again if suggested by the editor.

Comment: In Figure 6, were NCCs FACS sorted from whole-body samples? If so, are there any differences in the proportion of cranial, cardiac/vagal and trunk NC populations between control and KO embryos? It may affect the interpretation of data thereafter.

Response: *Yes, the NCCs were sorted from the whole embryos. Our goal was not to determine the role of BAF complex in different neural crest progenitor population but rather overall role in all neural crest population. As our data and published data indelicate that BAF155/170 are ubiquitously expressed in embryos, profiling all the neural crest will give a better picture of the role of BAF complex in neural crest development. Our analysis have demonstrated that most of the neural crest population (cranial, cardiac and trunk) are severely affected. We have looked into specific markers to determine the effect. For example, PlexinA2 is a specific marker for cardiac neural crest and its expression is significantly reduced in BAF155/170-deficient embryos.*

Comment: In Figure 8C, an illustration of the Tead-luciferase construct with Tead and Yap should be given.

Response: *We have included an illustration of the Tead-luciferase construct with Tead and Yap (Figure 8C).*

Comment: It is interesting that BAF155/170 knockdown disrupts Brg1-Tead interaction, but does not affect Brg1-Yap interaction. Did the authors have a chance to examine the effects of single knockout of BAF155 or BAF170? Further, does BAF155/170 knockdown affect Yap-Tead interaction, which is likely critical to the Tead-luciferase promoter activity?

Response: *To address this comment, we have performed new experiments to determine if interaction between Yap-Tead is also affected due to BAF155/170 deletion. In BAF155/170 knockdown cells, similar to Brg1-Tead, Yap-Tead interaction is also impaired (Figure 8). This result is consistent with the luciferase date set presented in Figure 8C, demonstrating that in the absence of BAF155/170, Yap-mediated activation of Tead reporter is significantly reduced.*

Due to phenotypic similarity between BAF155-deficient embryos and BAF155/170-deficient embryos, we decided to perform CoIP experiments on BAF155 knockdown O9-1 cells. We analysed Brg1-Tead, Brg1-Yap and Yap-Tead interactions in BAF155 knockdown O9-1 cells. New results demonstrate that Brg1-Yap interaction is intact but both Brg1-Tead and Yap-Tead interactions are severely affected due to BAF155 knockdown. We have included these new results in the revised Figure 8.

Comment: Considering the possible pleiotropic effects of Hippo signaling pathway, it seems difficult to identify which NCC lineage at which stage is affected in terms of proliferation, migration, differentiation and/or survival by inactivation of Brg1-Yap-Tead interaction. However, in situ examination of Tead-reporter activity or downstream gene expression in control and BAF155/170(Wnt1-CKO) embryos may give a clue to this issue.

Response: *To identify the cell population that is most severely affected due to inactivation of Brg1-Yap-Tead interaction, we have taken an unbiased approach. We analysed the Tead binding motifs in the 5kb promoter of differentially expressed genes (DEGs) identified in BAF155/170-deficient NCCs (Supplemental Figure 8A). We observed significant number of DEGs with Tead motifs. Next, we determined the number of Tead motifs in each DEGs and found that over hundred genes with more than one and less than five Tead motifs suggesting that they are potential Brg1-Yap-Tead targets (Supplemental Figure 8B). To determine the most severely affected population, we compared these DEGs with the neural crest scRNAseq data set. We observed that majority if these DEGs belong mesenchymal, sensory, pre-delaminatory and neural tube clusters suggesting that proliferation, differentiation and/or survival of these clusters are dependent on Brg1-Yap-Tead interaction (Supplemental Figure 8C). These results have been included in the revised manuscript (Supplemental Figure 8).*

Some of the recently published papers suggest role of Hippo signalling components Yap/Taz in regulating different aspects (proliferation, differentiation and survival) of neural crest development (Hindley et al., 2016; Kumar et al., 2019; Manderfield et al., 2015a; Manderfield et al., 2015b; Wang et al., 2016). For example, YAP is active in premigratory neural crest cells and loss of YAP function inhibits neural crest emigration (Kumar et al., 2019). YAP stimulates G1/S transition, mitosis and survival of premigratory neural crest cells (Kumar et al., 2019). Deletion of Yap and Taz in neural crest results in impaired smooth muscle differentiation (Manderfield et al., 2015a).Vascular defects, hemorrhage and neural tube defects were also observed after Yap and Taz inactivation (Wang et al., 2016). Our results are consistent with these published literature.

Significance:

Comment: The importance of BAF chromatin remodeling complex in NCC development has been reported by another group, as the authors cite in the manuscript (Li et al. PNAS 2013). However, the present manuscript provides new information about the nature of phenotypes and possible downstream mechanisms including Notch and Hippo pathway.

Response: *We would like to thank the reviewer again for his/her positive evaluation of our manuscript. Our data provide new insight into BAF155/170 dependent regulation of neural crest development. It is also important to note that our unbiased RNAseq approach has not been able to reproduce the gene expression changes observed previously by Li et al using a candidate based approach (Li et al., 2013).*

References:

High, F. A., Zhang, M., Proweller, A., Tu, L., Parmacek, M. S., Pear, W. S., and Epstein, J. A. (2007). An essential role for Notch in neural crest during cardiovascular development and smooth muscle differentiation. J Clin Invest *117*, 353-363.

Hindley, C. J., Condurat, A. L., Menon, V., Thomas, R., Azmitia, L. M., Davis, J. A., and Pruszak, J. (2016). The Hippo pathway member YAP enhances human neural crest cell fate and migration. Sci Rep *6*, 23208.

Ho, L., Ronan, J. L., Wu, J., Staahl, B. T., Chen, L., Kuo, A., Lessard, J., Nesvizhskii, A. I., Ranish, J., and Crabtree, G. R. (2009). An embryonic stem cell chromatin remodeling complex, esBAF, is essential for embryonic stem cell self-renewal and pluripotency. Proc Natl Acad Sci U S A *106*, 5181-5186.

Kim, K. S., Arima, Y., Kitazawa, T., Nishiyama, K., Asai, R., Uchijima, Y., Sato, T., Levi, G., Kitanaka, S., Igarashi, T., *et al.* (2013). Endothelin regulates neural crest deployment and fate to form great vessels through Dlx5/Dlx6-independent mechanisms. Mech Dev *130*, 553-566.

Kumar, D., Nitzan, E., and Kalcheim, C. (2019). YAP promotes neural crest emigration through interactions with BMP and Wnt activities. Cell Commun Signal *17*, 69.

Li, W., Xiong, Y., Shang, C., Twu, K. Y., Hang, C. T., Yang, J., Han, P., Lin, C. Y., Lin, C. J., Tsai, F. C., *et al.* (2013). Brg1 governs distinct pathways to direct multiple aspects of mammalian neural crest cell development. Proc Natl Acad Sci U S A *110*, 1738-1743.

Majesky, M. W. (2007). Developmental basis of vascular smooth muscle diversity. Arterioscler Thromb Vasc Biol *27*, 1248-1258.

Manderfield, L. J., Aghajanian, H., Engleka, K. A., Lim, L. Y., Liu, F., Jain, R., Li, L., Olson, E. N., and Epstein, J. A. (2015a). Hippo signaling is required for Notch-dependent smooth muscle differentiation of neural crest. Development *142*, 2962-2971.

Manderfield, L. J., Engleka, K. A., Aghajanian, H., Gupta, M., Yang, S., Li, L., Baggs, J. E., Hogenesch, J. B., Olson, E. N., and Epstein, J. A. (2015b). Pax3 and Hippo Signaling Coordinate Melanocyte Gene Expression in Neural Crest. Cell Rep *10*, 841.

Narayanan, R., Pirouz, M., Kerimoglu, C., Pham, L., Wagener, R. J., Kiszka, K. A., Rosenbusch, J., Seong, R. H., Kessel, M., Fischer, A., *et al.* (2015). Loss of BAF (mSWI/SNF) Complexes Causes Global Transcriptional and Chromatin State Changes in Forebrain Development. Cell Rep *13*, 1842-1854.

Nguyen, H., Sokpor, G., Pham, L., Rosenbusch, J., Stoykova, A., Staiger, J. F., and Tuoc, T. (2016). Epigenetic regulation by BAF (mSWI/SNF) chromatin remodeling complexes is indispensable for embryonic development. Cell Cycle *15*, 1317-1324.

Schussler, O., Gharibeh, L., Mootoosamy, P., Murith, N., Tien, V., Rougemont, A. L., Sologashvili, T., Suuronen, E., Lecarpentier, Y., and Ruel, M. (2020). Cardiac Neural Crest Cells: Their Rhombomeric Specification, Migration, and Association with Heart and Great Vessel Anomalies. Cell Mol Neurobiol.

Singh, N., Trivedi, C. M., Lu, M., Mullican, S. E., Lazar, M. A., and Epstein, J. A. (2011). Histone deacetylase 3 regulates smooth muscle differentiation in neural crest cells and development of the cardiac outflow tract. Circ Res *109*, 1240-1249.

Tuoc, T. C., Boretius, S., Sansom, S. N., Pitulescu, M. E., Frahm, J., Livesey, F. J., and Stoykova, A. (2013). Chromatin regulation by BAF170 controls cerebral cortical size and thickness. Dev Cell *25*, 256-269.

Wang, J., Xiao, Y., Hsu, C. W., Martinez-Traverso, I. M., Zhang, M., Bai, Y., Ishii, M., Maxson, R. E., Olson, E. N., Dickinson, M. E.*, et al.* (2016). Yap and Taz play a crucial role in neural crest-derived craniofacial development. Development *143*, 504-515.

Yan, Z., Wang, Z., Sharova, L., Sharov, A. A., Ling, C., Piao, Y., Aiba, K., Matoba, R., Wang, W., and Ko, M. S. (2008). BAF250B-associated SWI/SNF chromatin-remodeling complex is required to maintain undifferentiated mouse embryonic stem cells. Stem Cells *26*, 1155-1165.
