# Supplementary material for: Critical role of the BAF chromatin remodeling complex during murine neural crest development
Source: PLoS Genet. 2021 Mar 22;17(3):e1009446. doi: 10.1371/journal.pgen.1009446 (PMC8016319; doi:10.1371/journal.pgen.1009446)
Supplement: Attachment — Submitted filename: Response to Reviewers.pdf [file pgen.1009446.s014.pdf]

Reviewer #1 (PLOS Genetics):

Comment: A major weakness of the paper is that the craniofacial anomalies particularly with respect to the pharyngeal arches is evident in their smaller size at E10.5. The authors should have performed analyses of proliferation and apoptosis at E9.5 and if alterations were observed at that time point then they should have looked even earlier. It's clear that there is a deficiency in the number of migrating neural crest cells which on its own can explain the craniofacial anomalies including cleft palate.

Response: *We have performed morphological and histological analysis on E9.5 embryos and included the data in the revised manuscript (Figure 2A-D). No morphological differences were observed between different genotypes observed (Figure 2A-D). We have performed lineage tracing analysis at E9.5 but did not observe any obvious differences in the migration of NCCs (Figure 3A-D). We have also evaluated proliferation and apoptosis at E9.5. No significant change in the proliferation rate was observed. However, there was a significant increase in the number of apoptotic cells (Figure 5E and J). Similarly, a significant reduction in the proliferation and increase in apoptosis was observed in the palate shelves of the BAF170-knockout embryos compared to the controls, likely causing cleft palate (Sup Figure 3I-N). These results have been included in the revised manuscript.*

Comment: In general I found the analyses of the distinct phenotypes a bit superficial. There was no examination of why the palatal shelves failed to fuse in the BAF155 CKO mice. It's not clear to me why the authors traced neural crest cells using mTmG and then immunostained for GFP with a red secondary fluorescent antibody as an indication of neural crest cell localization, nor why they chose to also reveal PlexinA2 immunostaining with red fluorescence. This was a missed opportunity to show co-localization of neural crest cells with the respective markers. It's important to demonstrate whether the absence of protein immunostaining is due to an absence of the neural crest cells in that same territory or an indication of a cell fate change. Nonetheless this was the extent of the cellular analyses in the cko mutants.

Response: *The focus of our study was not palate phenotype but OFT and PAA. However, during the review of our manuscript by "Review Commons" the reviewers suggested looking at the BAF155 and BAF170 single knockout for cleft palate. As neural crest-specific BAF155-KO embryos die early we asked our collaborator to analyze the palates in Foxg1Cre/BAF155-KO. We observed cleft palate in the BAF155 KO mice (Sup Figure 4). Besides, we also analyzed palates in neural crest-specific BAF170-KO and observed cleft palate (Sup Figure 3A-H). These results were included in the revised manuscript.*

To address this point further, now we have analyzed proliferation and apoptotic assay on E15.5 control and BAF170-knockout embryos and observed a significant reduction in the number of proliferating cells and an increase in the number of apoptotic cells explaining the cleft palate phenotypes (Sup Figure 3I-N).

As suggested by this reviewer, we have also performed double immunostaining for GFP and Smooth muscle marker and observe that a reduced number of NCCs in the OFT is the reason for the lack of smooth muscle cells. We have modified the text to reflect this and included all the new results in the revised manuscript.

Comment: It's not clear to me why the authors chose E11.25 for their neural crest cell isolation and RNA-seq analyses. My concern is that many of the changes observed are likely secondary effects since they are observed well after the onset of the phenotype. But even if they could be classified as primary direct affects, the mechanism then needs to be evaluated in the mutant embryos. It doesn't make sense that the authors isolated neural crest cells at E11.25 and yet their data suggests pre-migratory neural crest factors are highly represented.

Response: *In the revised manuscript we have analyzed the phenotype at E9.5 and do not observe any significant embryonic defects. E10.5 is the earliest timepoint where we see minor changes in the craniofacial tissues. The reason to choose the E11.25 stage was due to technical challenges as we could not get enough RNA for the RNAseq analysis from the sorted NCCs from individual E10.5 embryos. We were reluctant to pool samples from different embryos as it may not represent the real biological replicate and affect the overall analysis.*

We agree that in addition to primary changes, RNAseq results may also include secondary changes. To address this further we have compared the genes that are differentially expressed in our RNAseq analysis with a recently published E9.5 neural crest single-cell RNAseq (scRNAseq) study (Soldatov et al., 2019). We examined the enrichment of differentially expressed genes (DEGs) observed in our study among the various expression-based cell clusters reported from the scRNAseq study, via Fisher's exact test. We observed that the majority of DEGs in BAF155/170-deficient NCCs belong to mesenchymal, sensory, pre-delaminatory, and neural tube clusters. Among the significant clusters, the strongest enrichment was observed for the mesenchymal cluster and the weakest for the neural tube cluster (Figure 9B-D). Also, to identify the primary changes due to the inactivation of Brg1-Yap-Tead interaction, we have taken an unbiased approach. We analyzed the Tead binding motifs in the 5kb promoter of differentially expressed genes (DEGs) identified in BAF155/170-deficient NCCs (Sup Figure 10A). We observed a significant number of DEGs with Tead motifs. Next, we determined the number of Tead motifs in each DEGs and found that over a hundred genes with more than one and less than five Tead motifs suggesting that they are potential Brg1-Yap-Tead targets (Sup Figure 10B). To determine the most severely affected population, we compared these DEGs with the neural crest scRNAseq data set. We observed that majority of these DEGs belong to mesenchymal, sensory, pre-delaminatory, and neural tube clusters suggesting that proliferation, differentiation, and/or survival of these clusters are dependent on Brg1-Yap-Tead interaction (Sup Figure 10C). These results have been included in the revised manuscript (Sup Figure 10).

Comment: The authors use luciferase and co-immunoprecipitation assays and competitive interactions assays in O9-1 cells to validate an interaction between BAF and Notch signaling, as well as BAF and HIPPO (Yap-Taz-Tead) signaling in vitro. While these interactions may be quite valid in neural crest cells at the E11.5 time point, the mutant embryo phenotype is apparent well before then. Thus there is a disconnect between the signaling links and their validation in a cell line with the temporal onset of the mutant embryo phenotype. As a result the authors rely heavily on assumed connections between these pathways from the literature and make correlative assertions about their importance in underlying the phenotypes but fail to demonstrate

causation. For example a thorough examination of altered Notch siganling as a key driver of the cardiovasular defects should be demonstrated spatiotemporally in the mutants. Similarly HIPPO/Tead signaling should also be validated in the same way. Ultimately there was a missed opportunity to chemically stimulate Notch and HIPPO signaling to test if this could ameliorate the cardiovascular phenotype and in doing so validate the phenotypic connection to these pathways.

Response: *In the revised manuscript we have analyzed the phenotype at E9.5 and do not observe any significant embryonic defects. E10.5 is the earliest timepoint where we see minor changes in the craniofacial tissues. The neural crest cell line O9-1 was chosen to determine the interaction between BAF and Notch signaling, as well as BAF and Hippo (Yap-Brg1-Tead) signaling as it was not possible to perform such analysis on sorted NCCs from embryos. Before using the O9-1 cell line, we performed a test round to estimate how much protein extract we could get from sorted NCCs from an individual embryo. Unfortunately, the protein amount we got was not even enough for one western blot making it impossible to used sorted NCCs for this experiment. Our RNAseq analysis and qPCR validation on isolated NCCs demonstrate that Notch and Hippo signaling pathway genes are significantly downregulated due to BAF deletion. In the revised manuscript, we have also performed immunostaining for pan-Tead (as Brg1 interaction with Tead is severely affected) on E10.5 control and BAF155/170-knockout embryos and see downregulation (Sup Figure 8).*

To determine if chemical stimulation of Notch and Hippo signaling pathways could ameliorate the cardiovascular phenotype observed in BAF155/170 knockout embryos, we decided to activate the Yap-dependent signaling by treating the embryos with a commercially available Hippo signaling kinase inhibitor XMUMP1 (Tocris, Cat. No. 6482)(Fan et al., 2016). Before doing the in vivo experiment, we tested the efficacy of this inhibitor in an in vitro experiment. We transfected HEK293T cells with Tead-luciferase (Tead-Luc) reporter with or without Yap in the absence or presence of XMU MP1 (three different concentrations were used, 0.1μM, 1μM, and 5μM) (A). If XMUMP1 inhibits the upstream endogenous kinase activity, Yap will not be phosphorylated. Unphosphorylated Yap will then translocate to the nucleus and activate the luciferase reporter. Our results showed that instead of activating, XMUMP1 treatment reduced the luciferase reporter activity. Next, we tested the efficacy of XMUMP1 inhibitor in a similar luciferase assay in the presence of Hippo signaling kinases Mst1 or Lats2 (B-C). We transfected HEK293T cells with Tead-Luc reporter with Yap and Mst1 in the absence or presence of XMU MP1 (B). Yap activated the Tead-Luc reporter activity. As expected, the addition of Mst1 reduced the Tead-Luc reporter activity. We expected that if XMUMP1 inhibits the Mst1 activity directly or indirectly, we will observe an increase in the Tead-Luc reporter activity. Unexpectedly, we observed further reduction in the Tead-Luc reporter activity suggesting that XMUMP1 is not affecting the kinases activity or Yap-dependent Tead-Luc reporter activity as expected. A similar trend was also observed when we replaced Mst1 with Lats2 kinase (C). Together these results suggest that XMUMP1 is not a specific and efficient Hippo kinase inhibitor and will not be suitable for in vivo rescue experiments.

Besides, our manuscript demonstrates that loss of BAF155/170 not only leads to the gene expression changes in Hippo signaling components but also their interaction at the protein levels. For example, BAF155 or BAF155/170 knockdown affects Tead interaction with both Yap and Brg1. So even if we can increase the endogenous Yap

activity by using a Hippo kinase inhibitor, it will not have any effect on the altered protein-protein interaction and it will not be able to rescue the developmental defects observed in BAF knockouts.

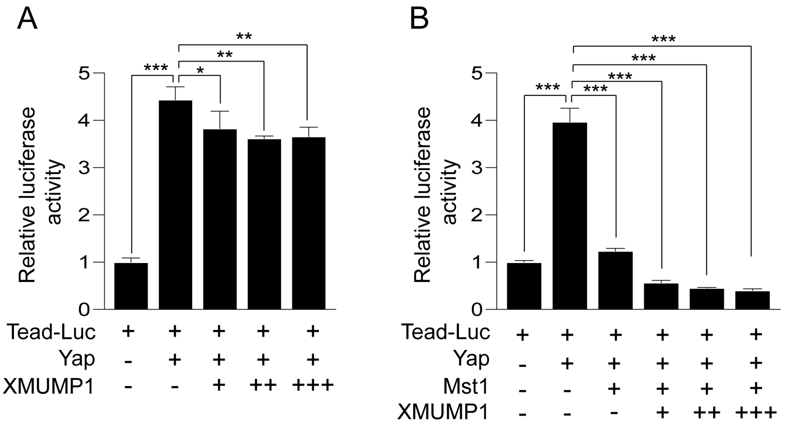

To the best of our knowledge, no Notch signaling activator is commercially available. A recent report has shown that N-methylhemeanthidine chloride can activate Notch signaling in acute myeloid leukemia (Ye et al., 2016). However, this chemical is not commercially available. Besides, N-methylhemeanthidine chloride has been shown to inhibits cell proliferation making it not suitable for use in the proposed rescue experiment.

Minor Comment: The authors state in the first line of the introduction that neural crest cells originate in the dorsal neural tube. This is incorrect with respect to cranial and cardiac neural crest cells in mice, which form and migrate from the dorsolateral edges of the neural plate prior to formation of the neural tube.

Response: *We have modified the text to reflect this.*

Reviewer #2 (PLOS Genetics):

An interesting manuscript detailing the role of members of the BAF complex in neural crest development and downstream transcriptional consequences. Images are clear and denote profound phenotypes that are likely interpreted correctly. I applaud the extra work that was performed by the authors to satisfy previous reviewer concerns. While there are some minor spelling and grammatical issues, no major concerns are noted.

Response: *We would like to thank the reviewer for his/her positive evaluation of our manuscript. We have corrected the spelling and grammatical issues in the revised manuscript.*

Reviewer #3 (PLOS Genetics):

Comment: In the manuscript “Critical role of the BAF chromatin remodeling complex during murine neural crest development” Bin et al. use different Cre-lines (Pax3, Wnt1, FoxG1) to analyze the function of core subunits of the BAF complex (BAF155 and BAF170) in mouse development. They observe that neural crest-specific deletion of BAF155/170 causes defects in cranio-facial, pharyngeal arch artery, cardiac outflow tract development and results in embryonic lethality. Proliferation and apoptotic assays suggest that BAF155/170 is required for neural crest proliferation and survival. Furthermore, RNAseq analysis suggests that the BAF complex controls the expression of signaling pathway genes critical for neural crest proliferation, migration and differentiation, like regulators of Notch and Hippo signaling. The authors present interesting data suggesting that the BAF complex plays a role in neural crest development and they provide new insight that BAF155/170 is required for Brg1-Yap-Tead-dependent transcription of target genes. However, my concern is that they may be looking here at secondary effects.

***Response:** In the revised manuscript we have analyzed the phenotype at E9.5 and do not observe any significant embryonic defects. E10.5 is the earliest timepoint where we see minor changes in the craniofacial tissues. The reason to choose the E11.25 stage was due to technical challenges as we could not get enough RNA for the RNAseq analysis from the sorted NCCs from individual E10.5 embryos. We were reluctant to pool samples from different embryos as it may not represent the real biological replicate and affect the overall analysis. We agree with the reviewer that the RNAseq results may also include secondary changes. To address this further we have compared the genes that are differentially expressed in our RNAseq analysis with a recently published E9.5 neural crest single-cell RNAseq (scRNAseq) study (Soldatov et al., 2019). We examined the enrichment of differentially expressed genes (DEGs) observed in our study among the various expression-based cell clusters reported from the scRNAseq study, via Fisher's exact test. We observed that the majority of DEGs in BAF155/170-deficient NCCs belong to mesenchymal, sensory, pre-delaminatory, and neural tube clusters. Among the significant clusters, the strongest enrichment was observed for the mesenchymal cluster and the weakest for the neural tube cluster (Figure 9B-D). Also, to identify the primary changes due to the inactivation of Brg1-Yap-Tead interaction, we have taken an unbiased approach. We analyzed the Tead binding motifs in the 5kb promoter of differentially expressed genes (DEGs) identified in BAF155/170-deficient NCCs (Sup Figure 10A). We observed a significant number of DEGs with Tead motifs. Next, we determined the number of Tead motifs in each DEGs and found that over a hundred genes with more than one and less than five Tead motifs suggesting that they are potential Brg1-Yap-Tead targets (Sup Figure 10B). To determine the most severely affected population, we compared these DEGs with the neural crest scRNAseq data set. We observed that majority of these DEGs belong to mesenchymal, sensory, pre-delaminatory, and neural tube clusters suggesting that proliferation, differentiation, and/or survival of these clusters are dependent on Brg1-Yap-Tead interaction (Sup Figure 10C). These results have been included in the revised manuscript (Sup Figure 10).*

Comment: The authors analyzed their Cre knockout lines at different time points (9.5 to 14.5). Fig. 1 and 2 starts by showing embryos at stage 10.5. Are there earlier defects

in neural crest development? (The authors should also comment, why they choose to analyze embryos at stages 9.5 to 14.5.)

Response: *We have analyzed the neural crest-specific embryos from E9.5-E12.5 in the revised manuscript. We focused our analysis on these stages due to the reasons that no morphological changes were observed at E9.5 and most of the double knockout embryos die after E12.5. For palate analysis, we have analyzed BAF155 and BAF170-knockouts at E15.5 as at this stage the secondary palate is formed. During secondary palate formation, palatal shelves initially grow vertically flanking the developing tongue and subsequently reorient to the horizontal position above the tongue in a process known as palatal shelf elevation. Following elevation, the palatal shelves grow towards the midline where they meet and fuse.*

Comment: What is the temporal expression of BAF155/170 in particular at different stages of neural crest development (induction, specification, etc.)? They only briefly comment on BAF155/170 expression in progenitor cells versus differentiated cells in the introduction. However, it would be helpful to characterize the expression of BAF155/170 at different stages using IHC, like the authors have previously shown in the olfactory epithelium (Bachmann et al., 2016).

Response: *Previous reports have shown that during mouse embryonic development, both BAF155 and BAF170 are ubiquitously expressed (Nguyen et al., 2016). BAF155 is strongly expressed in proliferating stem/progenitor cells and weakly in differentiated cells (Ho et al., 2009; Tuoc et al., 2013; Yan et al., 2008). In contrast, BAF170 is weakly expressed in proliferating stem/progenitor cells and strongly in differentiated cells (Ho et al., 2009; Tuoc et al., 2013; Yan et al., 2008). Although low expression of BAF170 is detected in stem/progenitor cells and BAF155 in differentiated cells, this expression is necessary and sufficient for stabilizing the BAF complex (Narayanan et al., 2015; Nguyen et al., 2016). We have discussed this in the introduction section of the revised manuscript.*

We have also performed double immunostaining for BAF155/Pax3 and BAF170/Pax3 to determine their temporal expression during neural crest development. Consistent with previous reports, we observed that both BAF155 and BAF170 are ubiquitously expressed. They nicely co-localize with Pax3 positive neural crest cells (Sup Figure 1). These results have been included in the revised manuscript.

Comment: The authors performed RNAseq analysis of FACS-sorted neural crest cells of their Wnt1-cre lines. Why did they choose embryos of E11.25? Would it not be useful to look at earlier stages to determine when BAF155/170 activity is initially required? As they observe gene expression changes in neural crest specifiers (Fig. 6) this may indicate defects in neural crest specification. Thus, the observed effects on neural crest guidance cues, hippo signaling etc. may just be secondary to defects in neural crest specification.

Response: *In the revised manuscript we have analyzed the phenotype at E9.5 and do not observe any significant embryonic defects. E10.5 is the earliest timepoint where we see minor changes in the craniofacial tissues. The reason to choose the E11.25 stage was due to technical challenges as we could not get enough RNA for the RNAseq analysis from the sorted NCCs from individual E10.5 embryos. We were*

reluctant to pool samples from different embryos as it may not represent the real biological replicate and affect the overall analysis.

We agree with the reviewer that the RNAseq results may also include secondary changes. To address this further we have compared the genes that are differentially expressed in our RNAseq analysis with a recently published E9.5 neural crest single-cell RNAseq (scRNAseq) study (Soldatov et al., 2019). We examined the enrichment of differentially expressed genes (DEGs) observed in our study among the various expression-based cell clusters reported from the scRNAseq study, via Fisher's exact test. We observed that the majority of DEGs in BAF155/170-deficient NCCs belong to mesenchymal, sensory, pre-delaminatory, and neural tube clusters. Among the significant clusters, the strongest enrichment was observed for the mesenchymal cluster and the weakest for the neural tube cluster (Figure 9B-D). Also, to identify the primary changes due to the inactivation of Brg1-Yap-Tead interaction, we have taken an unbiased approach. We analyzed the Tead binding motifs in the 5kb promoter of differentially expressed genes (DEGs) identified in BAF155/170-deficient NCCs (Sup Figure 10A). We observed a significant number of DEGs with Tead motifs. Next, we determined the number of Tead motifs in each DEGs and found that over a hundred genes with more than one and less than five Tead motifs suggesting that they are potential Brg1-Yap-Tead targets (Sup Figure 10B). To determine the most severely affected population, we compared these DEGs with the neural crest scRNAseq data set. We observed that majority of these DEGs belong to mesenchymal, sensory, pre-delaminatory, and neural tube clusters suggesting that proliferation, differentiation, and/or survival of these clusters are dependent on Brg1-Yap-Tead interaction (Sup Figure 10C). These results have been included in the revised manuscript (Sup Figure 10).

Comment: As it is known that Wnt1-Cre labels a large population of non-neural crest cells in the neural plate this Cre-line may not be the best choice to determine gene expression changes in neural crest cells. In fact, they detect gene expression changes in a number of genes involved in axon guidance and neural crest migration, like different classes of semaphorins (Fig. 6). One would expect that these guidance cues are expressed in non-neural crest tissue, while neural crest cells express for example plexin receptors. Which leaves the question, what is the nature of the cells that they have been analyzing?

Response: *Among all the cre lines used for the genetic fate mapping of neural crest cells in the mouse, only Wnt1-Cre and Pax3-Cre lines are active in pre-migratory neural crest cells (Debbache et al., 2018). All other cre lines (AP2a, P0, Plp, Sox10, etc.) express Cre-recombinase in neural crest cells not before they undergo an EMT in the dorsal neural tube, but only as the cells begin to migrate, making them unsuitable for the present study (Debbache et al., 2018). To the best of our knowledge, Wnt1-Cre is a well-established and best cre-driver line for neural crest lineage tracing and functional analysis (Debbache et al., 2018).*

For the RNAseq analysis, we have sorted GFP$^+$ NCCs from lineage traced embryos so we are not only collecting NCCs but also NC-derived cells. The reason why we observe changes in some genes involved in axon guidance is that neural crest cells contribute to the formation of the nervous system (Cranial NCCs contribute to cranial ganglia, Vagal NCCs contribute to enteric ganglia, Trunk NCCs contribute to dorsal

root and sympathetic ganglia and Sacral NCCs contribute to enteric and sympathetic ganglia) (Rothstein et al., 2018). Many of the Semaphorins and Plexins identified in our RNAseq analysis have been implicated in neural crest biology (Anderson et al., 2007; Berndt and Halloran, 2006; Fujiwara et al., 2018; Gammill et al., 2007; Gammill et al., 2006; Kodo et al., 2017; Luzon-Toro et al., 2013; Lwigale and Bronner-Fraser, 2009; Plein et al., 2015; Toyofuku et al., 2008; Ufartes et al., 2018; Waimey et al., 2008; York et al., 2018).

Comment: The authors use lineage-tracing to analyze if neural crest migration or differentiation are affected (Fig. 3). Although there are GFP-positive cells in the branchial arches of the BAF155/170Wnr1-CKO, the branchial arch area is dramatically smaller and there seem to be fewer migrating cells (Fig. 3D), which may indicate migration defects. The authors also show frontal sections, however, it is difficult to compare these; for example the controls seem to show less GFP signal. Better imaging and quantification is required to exclude migration defects.

Response: *In the revised manuscript we have also included results from E9.5 lineage tracing analysis. Using both whole-mount GFP imaging and GFP immunostaining on sections, we do not see obvious migration defects in the developing arches. We think the reduction in the branchial arch area is most likely due to the reduction in cell proliferation and increase in apoptosis due to BAF155/170 deletion. Lineage tracing analysis from E9.5, E10.5, and E12.5 control and BAF155/170 knockouts show that NCCs can migrate to arches. We have revised the figure with new data and better imaging.*

Comment: In addition to point 5: The lack of SM2alpha (Fig. 3 M-P) or plexinA2-positive cells (Fig. 4 E-H) may simply result from defects in proliferation or increased apoptosis and not represent a defect in differentiation. For example the data in Fig. 3M-P could be interpreted as a differentiation defect, but may also result from defects in migration, proliferation or increased apoptosis.

Response: *We agree with the reviewer that defects in proliferation and apoptosis may also contribute to the lack of SM22a cells. Our conclusion that the lack of smooth muscle in the PAA is due to defective differentiation is based on the fact that even in the BAF155/170-knockout samples, there are enough GFP$^+$ NCCs around the PAA but not able to differentiate efficiently (Figure 3Q-T). In contrast to PAA, our new data suggest that lack of smooth muscle in the OFT is not due to defective differentiation but due to lack of GFP$^+$ NCCs, likely due to impaired migration, proliferation, or increased apoptosis as suggested by the reviewer (Figure 4K-L). We have included the new results and revised the text to reflect this.*

Comment: Quantification of the relative distance of cardiac neural crest migration. How was the distance (arrowhead in Figs. 4) determined? From Figs. 4B, D it is not clear how they chose the length of the double head arrows.

Response: *Quantification of the relative distance of cardiac neural crest migration into the cardiac OFT was done by measuring the distance between the start of the OFT towards the last visible neural crest cell as shown by GFP immunofluorescent staining. 3 control samples and 4 BAF155/170 knockout samples were stained and 5 sections from each embryos were used for quantification.*

Reviewer #1 (Review Commons):

Comment (Summary): The manuscript by Bin-Lin et al examines the role of BAF subunits 155 and 170 in development. Using several different Cre lines, they knockout BAF155/170 function in Pax3 (neural plate border, somites etc), Wnt1 (neural crest), Foxg1 (epithelium etc) positive cells showing that BAF function is required for craniofacial, artery and outflow track development. Analysis of cell death and proliferation suggest that both processes are defective, which is supported by transcriptome analysis of sorted neural crest cells in the double knockout and reduction of Notch and Yap signaling components. Finally, the authors show that the BAF complex is essential for Tead dependent transcription of target genes. While the data is interesting and nicely presented, there are some issues that should be corrected before publication. My comments are below:

Response: *We would like to thank the reviewer for his/her positive evaluation of our manuscript.*

Comment: It is unclear why the authors use multiple Cre lines. The use of Pax3-Cre is not well explained. What is the rationale for using this Cre vs the others? FoxG1-Cre also targets pharyngeal pouches, and head and face ectoderm. Were other craniofacial structures affected in mutants besides palatal formation? In addition, it seems like there is a focus on the palate formation and neural crest cell development but then also present artery and outflow track. While this data is interesting, it disrupts the flow of the manuscript and could be added to the supplemental data.

Response: *Our goal was to use both Pax3 and Wnt1 cre to delete BAF155 and BAF170 in the neural crest cells. Wnt1Cre is a neural crest-specific cre. However, Pax3 is also expressed in the neural crest, in addition to other tissues such as somites. BAF155/170 deletion using both cre lines gave similar phenotypes.*

We have only analyzed palate phenotype in $FoxG1^{Cre}$;$BAF155^{fl/fl}$ mice as our focus was on the pharyngeal arch arteries and cardiac outflow tract (OFT). We have reduced the palate phenotype description and added it to the supplemental data in the revised manuscript so it does not disrupt the flow of the manuscript.

Comment: Where are BAF subunits (BAF155/170) expressed in development? Are they specifically expressed in NC and NC-derived tissues? Does each subunit, 155 or 170 have the same or different expression patterns? This should be discussed in the introduction if not presented.

Response: *During embryonic development, both BAF155 and BAF170 are ubiquitously expressed (Nguyen et al., 2016). BAF155 is strongly expressed in proliferating stem/progenitor cells and weakly in differentiated cells (Ho et al., 2009; Tuoc et al., 2013; Yan et al., 2008). In contrast, BAF170 is weakly expressed in proliferating stem/progenitor cells and strongly in differentiated cells (Ho et al., 2009; Tuoc et al., 2013; Yan et al., 2008). Although low expression of BAF170 is detected in stem/progenitor cells and BAF155 in differentiated cells, this expression is necessary and sufficient for stabilizing the BAF complex (Narayanan et al., 2015; Nguyen et al., 2016). We have discussed this in the introduction section of the revised manuscript.*

We have also performed double immunostaining for BAF155/Pax3 and BAF170/Pax3 to determine their temporal expression during neural crest development. Consistent with previous reports, we observed that both BAF155 and BAF170 are ubiquitously expressed. They nicely co-localize with Pax3 positive neural crest cells (Sup Figure 1). These results have been included in the revised manuscript.

Comment: Some of the data is not shown such as the palate data for single BAF170 mutants. This should be included, at least in supplemental data. In addition, the authors focus on E14.5 for palate development (but this data is not shown), but also important to look at E15.5 after the palatal shelf have fused. Were palates also assessed in the Wnt-Cre and FoxG1-Cre BAF170 animals and also normal? What about the double BAF155/170 FoxG1-Cre? Do BAF155/BAF170 compensate for each other? Do their expression patterns change in the mutants?

Response: *We have not analyzed the FoxG1-Cre BAF170 animals. However, in the revised manuscript, we have included palate data from E15.5 BAF170-deficient ($Wnt1^{Cre/+}$;$BAF170^{flox/flox}$;$BAF155^{flox/+}$) embryos and observed cleft palate (Sup Figure 3). All the palate analysis was performed at E15.5 as suggested by the reviewer (Sup Figure 3 and 4). We provide evidence that cell proliferation and apoptosis were significantly affected in the BAF170-deficient palate shelves (Sup Figure 3I-N)*

We have not generated BAF155/170 FoxG1-Cre mice as the focus of our study was the neural crest. However, it has been demonstrated that the global deletion of BAF155/170 using CAG^{Cre} (induced at E9.5) leads to early embryonic lethality making it difficult to examine the palate defects.

To determine if they compensate for each other, we have analyzed the expression of BAF155 and BAF170 in BAF155-, BAF170- and BAF155/170-knockdown O9-1 cells but did not observe any significant changes at the RNA levels suggesting compensation. We can include these results in the revised manuscript if Editor suggests.

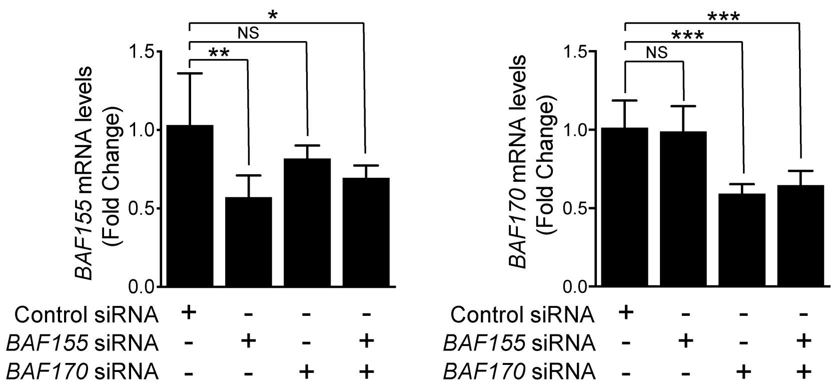

Comment: There is an issue with presentation of the "single" mutants as the data is in BAF155 fl/+;BAF170 fl/fl which is homozygous for 170 but heterozygous for 155. These cannot be considered single mutants as there may be phenotypes when one allele is reduced. This brings up the question if combinatorial heterozygote animals have phenotypes in any of the Cre mutants presented such as BAF155 fl/+;BAF170

fl/+? What is the degree of penetrance for all phenotypes analyzed, for example, hemorrhaging in the telencephalon?

Response: *We agree with the reviewer. In the revised manuscript, we have now presented the actual genotypes analyzed in each experiment. The combine heterozygous mice (Pax3$^{Cre/+}$;BAF155$^{fl/+}$;BAF170$^{fl/+}$ and Wnt1$^{Cre/+}$;BAF155$^{fl/+}$;BAF170$^{fl/+}$) are normal and fertile and show no phenotype. These mice were crossed to BAF155$^{fl/fl}$;BAF170$^{fl/fl}$ mice to generate all the genotypes analyzed in this study. We had included this information in the result section.*

We have included the degree of penetrance for all phenotypes analyzed in the respective figure legend.

Comment: In general, all the analysis of the data presented needs to be better quantified. The gene expression and GFP signal in the lineage tracing is not quantified. In Figure 4, NCC migration to the developing heart was assessed by evaluating the number of neural crest in the conotruncal cushions of the OFT. In the text, this data was quantified but not shown. Please show this quantification. Additionally, what was quantified should be better described. BrdU pulse chase is a better assessment of proliferation rather than Ki67 staining. Is the number of Ki67 positive cells a reflection of the total number of cells per tissue area? What is the normalization of quantification?

Response: *We have quantified the distance migrated by the NCCs into the OFT and NCCs positive (GFP+) area within the OFT. These results have been included in the revised manuscript (Figure 4E-F). Both Ki67 and BrdU are the most commonly used markers to evaluate cell proliferation in embryonic tissues. Quantification of cell proliferation was calculated as the ratio of Ki67-positive cells to the total number of cells as determined by Dapi counterstaining in the defined area of the neural tube and pharyngeal arch. We have included these details in the results, figure legend as well as in the materials and methods section of the revised manuscript.*

Comment: For IP experiments, does single loss of either BAF subunit cause the same disruption of Brg1 with Tead? The phenotypes in the animal model of the BAF155 mutants were very similar to the BAF155/170 double mutants, only mildly more severe than the single mutant. This suggests BAF155 is the key subunit. Therefore, is the interaction between Brg1 of the BAF complex and Tead clearly dependent on BAF155 alone?

Response: *We agree with the reviewer that BAF155 is the key subunit. Literature suggests that BAF155 is strongly expressed in proliferating stem/progenitor cells and weakly in differentiated cells (Ho et al., 2009; Tuoc et al., 2013; Yan et al., 2008). In contrast, BAF170 is weakly expressed in proliferating stem/progenitor cells and strongly in differentiated cells (Ho et al., 2009; Tuoc et al., 2013; Yan et al., 2008). As we are deleting BAF155/170 in neural crest progenitors, BAF155 seems to play a more dominant role compared to BAF170. To determine the role of BAF155 in modulating the interaction between Brg1 and Tead, we have performed new IP experiments using BAF155 knockdown O9-1 cells. We see that with reduced levels of BAF155 alone, the interaction between Brg1 and Tead is impaired. We have included these results in Figure 8 of the revised manuscript.*

Comment (Minor): Add references to single BAF subunit mutants in the introduction.

Response: *We have included more references to the introduction part of the revised manuscript.*

Comment (Minor): Sections in Figure 3I-L don't look consistent spatially across genotypes.

Response: *We have revised the figure with additional images to make it consistent across all genotypes.*

Comment (Minor): If would be nice to add references to the previously published roll of BAF in chromatin modification and how that fits with this new story.

Response: *We have included more references in the introduction section and discussed the data in context to the existing literature related to embryonic development and most specifically to neural crest cells.*

Comment (Significance): This manuscript advances our understanding of the role of BAF 155/170 in development. It adds to the existing knowledge of the role in chromatin modification to direct transcriptional regulators. The audience is developmental biologists. Our expertise is in neural crest and craniofacial development.

Response: *We would like to thank again the reviewer for his/her positive evaluation of our manuscript. We have addressed most of the recommendations this reviewer has provided.*

Reviewer #2 (Review Commons):

Comment (Summary): In this manuscript, Bi-Lin and colleagues investigate the role of BAF chromatin remodeling complex in mouse NCC development. They deleted BAF155/BAF170 in NCCs using two different Cre-expressing mouse lineages. NCC-specific BAF155/BAF170 knockout leads to embryonic lethality and developmental defects involving craniofacial, pharyngeal arch artery, and OFT defects. RNAseq and transcription factor enrichment analysis revealed that the BAF complex modulates expression of multiple signaling pathway genes including Hippo and Notch. In vitro experiments demonstrated that BAF complex is essential for Brg1-Yap-Tead-dependent transcription activity in NCCs. The authors conclude that the BAF complex is important in modulating the gene regulatory network essential for neural crest development. Overall, this manuscript is interesting and data are well presented. However, the manuscript suffers from several flaws that reduce the impact of the findings as commented below.

Response: *We would like to thank the reviewer for his/her positive evaluation of our manuscript.*

Comment: In Figure 3M-P, the authors show a severe deficiency of SM22alpha+ cells in the vascular wall of the pharyngeal arteries in BAF155/170(Wnt1-CKO) or BAF155/170(Pax3-CKO)embryos and conclude that the BAF complex is essential for mature smooth muscle differentiation from NCCs. The authors should indicate which pharyngeal arteries are shown and show that SM22alpha-negative NCCs are present around the arterial wall. The authors should also note that SM22alpha is not a "mature" smooth muscle marker, but is expressed in immature smooth muscle cells and myofibroblast-like cells. If SM22alpha-negative NCCs are surrounding the pharyngeal arch endothelium, it may suggest that an earlier stage than smooth muscle maturation may be disrupted.

Response: *In Figure 3M-P, the 4th pharyngeal arch artery is shown. We have included this information in the figure legend. We have performed additional neural crest-lineage tracing experiments and performed GFP and SM22α double immunostaining to show SM22α^{+} pharyngeal artery and GFP^{+} NCCs around the arterial wall. We agree with the reviewer that SM22α is not a mature smooth muscle marker. Our goal was to show that NCCs differentiation into smooth muscle cells is affected due to BAF155/170 deletion. We did not intend to evaluate the maturation of smooth muscle cells. Results from the lineage-tracing experiments demonstrate SM22α negative NCCs surrounding the pharyngeal arch endothelium suggesting that the smooth muscle cells differentiation but not maturation was disrupted. We have included these results in the revised Figure 3M-P and modified the text to reflect them.*

Comment: In Figure 4G, H, the authors show a decreased number of SM22alpha+ cells in the BAF155/170(Wnt1-CKO) conotruncal cushion. The authors should show whether these SM22alpha+ cells are NCCs or of non-NCC origin. If SM22alpha expression is impaired in BAF155/170-deficient NCCs, the latter is expected.

Response: *We observed a decreased number of SM22α^{+} cells in the BAF155/170-deficient conotruncal cushion (Figure 4I-J). This is consistent with the decreased number of migrating NCCs in the conotruncal cushion (Figure 4A-F). We have now*

quantified the NCCs (GFP⁺) area in the conotruncal cushion and see a severe reduction in BAF155/170 mutants. Consistently, reduced PlexinA2, a marker for cardiac neural crest, expression supports the notion that there are very few NCCs present in the conotruncal cushion of the OFT. These results suggest that the reduction in the number of SM22α^+ cells is due to the reduced number of cardiac NCCs in the OFT. We have performed GFP and SM22α double immunostaining to demonstrate their neural crest origin as suggested by the editor and included the new result in the revised manuscript (Figure 4K-L). Numerous papers have demonstrated that the smooth muscle cells of the conotruncal cushion and pharyngeal arch arteries are derived from the neural crest cells (High et al., 2007; Kim et al., 2013; Majesky, 2007; Schussler et al., 2020; Singh et al., 2011).

Comment: In Figure 5, the authors should evaluate proliferative and apoptotic cells in NCCs especially in the pharyngeal arches of control and BAF155/170(Wnt1-CKO) pharyngeal arches. Although the authors show increased Ki67+ and TUNEL+ cells in the knockout tissues, it is possible that BAF155/170-deficient NCCs may affect neighborhood cells in a non-cell autonomous manner.

Response: *Our RNAseq analysis in isolated NCCs demonstrates that genes required for NCCs proliferation are severely downregulated (including Hippo signaling genes) and apoptotic genes are significantly upregulated suggesting that cellular changes observed in BAF155/170-deficient embryos are due to changes in a cell-autonomous manner (Figure 6B). Also, we have now included proliferation and apoptotic quantification data from E9.5 control and BAF155/170-knockout embryos as well as E15.5 control and BAF170-knockout palates in the revised manuscript (Figure 5E and J; Sup Figure 3I-M).*

Comment: In Figure 6, were NCCs FACS sorted from whole-body samples? If so, are there any differences in the proportion of cranial, cardiac/vagal and trunk NC populations between control and KO embryos? It may affect the interpretation of data thereafter.

Response: *Yes, the NCCs were sorted from the whole embryos. Our goal was not to determine the role of the BAF complex in different neural crest progenitor populations but rather its overall role in all neural crest populations. As our data and published data indelicate that BAF155/170 are ubiquitously expressed in embryos, profiling all the neural crests will give a better picture of the role of the BAF complex in neural crest development. Our analysis has demonstrated that most of the neural crest population (cranial, cardiac, and trunk) are severely affected. We have looked into specific markers to determine the effect. For example, PlexinA2 is a specific marker for cardiac neural crest and its expression is significantly reduced in BAF155/170-deficient embryos.*

Comment: In Figure 8C, an illustration of the Tead-luciferase construct with Tead and Yap should be given.

Response: *We have included an illustration of the Tead-luciferase construct with Tead and Yap (Figure 8C).*

Comment: It is interesting that BAF155/170 knockdown disrupts Brg1-Tead interaction, but does not affect Brg1-Yap interaction. Did the authors have a chance to examine the effects of single knockout of BAF155 or BAF170? Further, does BAF155/170 knockdown affect Yap-Tead interaction, which is likely critical to the Tead-luciferase promoter activity?

Response: *To address this comment, we have performed new experiments to determine if the interaction between Yap-Tead is also affected due to BAF155/170 deletion. In BAF155/170 knockdown cells, similar to Brg1-Tead, Yap-Tead interaction is also impaired (Figure 8). This result is consistent with the luciferase data set presented in Figure 8C, demonstrating that in the absence of BAF155/170, Yap-mediated activation of Tead reporter is significantly reduced.*

Due to phenotypic similarity between BAF155-deficient embryos and BAF155/170-deficient embryos, we decided to perform CoIP experiments on BAF155 knockdown O9-1 cells. We analyzed Brg1-Tead, Brg1-Yap, and Yap-Tead interactions in BAF155 knockdown O9-1 cells. New results demonstrate that Brg1-Yap interaction is intact but both Brg1-Tead and Yap-Tead interactions are severely affected due to BAF155 knockdown. We have included these new results in revised Figure 8.

Comment: Considering the possible pleiotropic effects of Hippo signaling pathway, it seems difficult to identify which NCC lineage at which stage is affected in terms of proliferation, migration, differentiation and/or survival by inactivation of Brg1-Yap-Tead interaction. However, in situ examination of Tead-reporter activity or downstream gene expression in control and BAF155/170(Wnt1-CKO) embryos may give a clue to this issue.

Response: *To identify the cell population that is most severely affected due to the inactivation of Brg1-Yap-Tead interaction, we have taken an unbiased approach. We analyzed the Tead binding motifs in the 5kb promoter of differentially expressed genes (DEGs) identified in BAF155/170-deficient NCCs (Sup Figure 10A). We observed a significant number of DEGs with Tead motifs. Next, we determined the number of Tead motifs in each DEGs and found that over a hundred genes with more than one and less than five Tead motifs suggesting that they are potential Brg1-Yap-Tead targets (Sup Figure 10B). To determine the most severely affected population, we compared these DEGs with the neural crest scRNAseq data set. We observed that majority of these DEGs belong to mesenchymal, sensory, pre-delaminatory, and neural tube clusters suggesting that proliferation, differentiation, and/or survival of these clusters are dependent on Brg1-Yap-Tead interaction (Sup Figure 10C). These results have been included in the revised manuscript (Sup Figure 10).*

Some of the recently published papers suggest the role of Hippo signaling components Yap/Taz in regulating different aspects (proliferation, differentiation, and survival) of neural crest development (Hindley et al., 2016; Kumar et al., 2019; Manderfield et al., 2015a; Manderfield et al., 2015b; Wang et al., 2016). For example, YAP is active in premigratory neural crest cells and loss of YAP function inhibits neural crest emigration (Kumar et al., 2019). YAP stimulates G1/S transition, mitosis, and survival of premigratory neural crest cells (Kumar et al., 2019). Deletion of Yap and Taz in neural crest results in impaired smooth muscle differentiation (Manderfield et al., 2015a). Vascular defects, hemorrhage, and neural tube defects were also observed after Yap

and Taz inactivation (Wang et al., 2016). Our results are consistent with these published literature.

Comment (Significance): The importance of BAF chromatin remodeling complex in NCC development has been reported by another group, as the authors cite in the manuscript (Li et al. PNAS 2013). However, the present manuscript provides new information about the nature of phenotypes and possible downstream mechanisms including Notch and Hippo pathway.

Response: *We would like to thank the reviewer again for his/her positive evaluation of our manuscript. Our data provide new insight into BAF155/170 dependent regulation of neural crest development. It is also important to note that our unbiased RNAseq approach has not been able to reproduce the gene expression changes observed previously by Li et al using a candidate-based approach (Li et al., 2013).*

References:

Anderson, R.B., Bergner, A.J., Taniguchi, M., Fujisawa, H., Forrai, A., Robb, L., and Young, H.M. (2007). Effects of different regions of the developing gut on the migration of enteric neural crest-derived cells: a role for Sema3A, but not Sema3F. Dev Biol *305*, 287-299.

Berndt, J.D., and Halloran, M.C. (2006). Semaphorin 3d promotes cell proliferation and neural crest cell development downstream of TCF in the zebrafish hindbrain. Development *133*, 3983-3992.

Debbache, J., Parfejevs, V., and Sommer, L. (2018). Cre-driver lines used for genetic fate mapping of neural crest cells in the mouse: An overview. Genesis *56*, e23105.

Fan, F., He, Z., Kong, L.L., Chen, Q., Yuan, Q., Zhang, S., Ye, J., Liu, H., Sun, X., Geng, J., et al. (2016). Pharmacological targeting of kinases MST1 and MST2 augments tissue repair and regeneration. Sci Transl Med *8*, 352ra108.

Fujiwara, N., Miyahara, K., Nakazawa-Tanaka, N., Akazawa, C., and Yamataka, A. (2018). Increased expression of Semaphorin 3A in the endothelin receptor-B null mouse model of Hirschsprung disease. J Pediatr Surg *53*, 326-329.

Gammill, L.S., Gonzalez, C., and Bronner-Fraser, M. (2007). Neuropilin 2/semaphorin 3F signaling is essential for cranial neural crest migration and trigeminal ganglion condensation. Dev Neurobiol *67*, 47-56.

Gammill, L.S., Gonzalez, C., Gu, C., and Bronner-Fraser, M. (2006). Guidance of trunk neural crest migration requires neuropilin 2/semaphorin 3F signaling. Development *133*, 99-106.

High, F.A., Zhang, M., Proweller, A., Tu, L., Parmacek, M.S., Pear, W.S., and Epstein, J.A. (2007). An essential role for Notch in neural crest during cardiovascular development and smooth muscle differentiation. J Clin Invest *117*, 353-363.

Hindley, C.J., Condurat, A.L., Menon, V., Thomas, R., Azmitia, L.M., Davis, J.A., and Pruszak, J. (2016). The Hippo pathway member YAP enhances human neural crest cell fate and migration. Sci Rep *6*, 23208.

Ho, L., Ronan, J.L., Wu, J., Staahl, B.T., Chen, L., Kuo, A., Lessard, J., Nesvizhskii, A.I., Ranish, J., and Crabtree, G.R. (2009). An embryonic stem cell chromatin

remodeling complex, esBAF, is essential for embryonic stem cell self-renewal and pluripotency. Proc Natl Acad Sci U S A *106*, 5181-5186.
Kim, K.S., Arima, Y., Kitazawa, T., Nishiyama, K., Asai, R., Uchijima, Y., Sato, T., Levi, G., Kitanaka, S., Igarashi, T., et al. (2013). Endothelin regulates neural crest deployment and fate to form great vessels through Dlx5/Dlx6-independent mechanisms. Mech Dev *130*, 553-566.
Kodo, K., Shibata, S., Miyagawa-Tomita, S., Ong, S.G., Takahashi, H., Kume, T., Okano, H., Matsuoka, R., and Yamagishi, H. (2017). Regulation of Sema3c and the Interaction between Cardiac Neural Crest and Second Heart Field during Outflow Tract Development. Sci Rep *7*, 6771.
Kumar, D., Nitzan, E., and Kalcheim, C. (2019). YAP promotes neural crest emigration through interactions with BMP and Wnt activities. Cell Commun Signal *17*, 69.
Li, W., Xiong, Y., Shang, C., Twu, K.Y., Hang, C.T., Yang, J., Han, P., Lin, C.Y., Lin, C.J., Tsai, F.C., et al. (2013). Brg1 governs distinct pathways to direct multiple aspects of mammalian neural crest cell development. Proc Natl Acad Sci U S A *110*, 1738-1743.
Luzon-Toro, B., Fernandez, R.M., Torroglosa, A., de Agustin, J.C., Mendez-Vidal, C., Segura, D.I., Antinolo, G., and Borrego, S. (2013). Mutational spectrum of semaphorin 3A and semaphorin 3D genes in Spanish Hirschsprung patients. PLoS One *8*, e54800.
Lwigale, P.Y., and Bronner-Fraser, M. (2009). Semaphorin3A/neuropilin-1 signaling acts as a molecular switch regulating neural crest migration during cornea development. Dev Biol *336*, 257-265.
Majesky, M.W. (2007). Developmental basis of vascular smooth muscle diversity. Arterioscler Thromb Vasc Biol *27*, 1248-1258.
Manderfield, L.J., Aghajanian, H., Engleka, K.A., Lim, L.Y., Liu, F., Jain, R., Li, L., Olson, E.N., and Epstein, J.A. (2015a). Hippo signaling is required for Notch-dependent smooth muscle differentiation of neural crest. Development *142*, 2962-2971.
Manderfield, L.J., Engleka, K.A., Aghajanian, H., Gupta, M., Yang, S., Li, L., Baggs, J.E., Hogenesch, J.B., Olson, E.N., and Epstein, J.A. (2015b). Pax3 and Hippo Signaling Coordinate Melanocyte Gene Expression in Neural Crest. Cell Rep *10*, 841.
Narayanan, R., Pirouz, M., Kerimoglu, C., Pham, L., Wagener, R.J., Kiszka, K.A., Rosenbusch, J., Seong, R.H., Kessel, M., Fischer, A., et al. (2015). Loss of BAF (mSWI/SNF) Complexes Causes Global Transcriptional and Chromatin State Changes in Forebrain Development. Cell Rep *13*, 1842-1854.
Nguyen, H., Sokpor, G., Pham, L., Rosenbusch, J., Stoykova, A., Staiger, J.F., and Tuoc, T. (2016). Epigenetic regulation by BAF (mSWI/SNF) chromatin remodeling complexes is indispensable for embryonic development. Cell Cycle *15*, 1317-1324.
Plein, A., Calmont, A., Fantin, A., Denti, L., Anderson, N.A., Scambler, P.J., and Ruhrberg, C. (2015). Neural crest-derived SEMA3C activates endothelial NRP1 for cardiac outflow tract septation. J Clin Invest *125*, 2661-2676.
Rothstein, M., Bhattacharya, D., and Simoes-Costa, M. (2018). The molecular basis of neural crest axial identity. Dev Biol *444 Suppl 1*, S170-S180.
Schussler, O., Gharibeh, L., Mootoosamy, P., Murith, N., Tien, V., Rougemont, A.L., Sologashvili, T., Suuronen, E., Lecarpentier, Y., and Ruel, M. (2020). Cardiac Neural Crest Cells: Their Rhombomeric Specification, Migration, and Association with Heart and Great Vessel Anomalies. Cell Mol Neurobiol.
Singh, N., Trivedi, C.M., Lu, M., Mullican, S.E., Lazar, M.A., and Epstein, J.A. (2011). Histone deacetylase 3 regulates smooth muscle differentiation in neural crest cells and development of the cardiac outflow tract. Circ Res *109*, 1240-1249.

Soldatov, R., Kaucka, M., Kastriti, M.E., Petersen, J., Chontorotzea, T., Englmaier, L., Akkuratova, N., Yang, Y., Haring, M., Dyachuk, V., et al. (2019). Spatiotemporal structure of cell fate decisions in murine neural crest. Science *364*.
Toyofuku, T., Yoshida, J., Sugimoto, T., Yamamoto, M., Makino, N., Takamatsu, H., Takegahara, N., Suto, F., Hori, M., Fujisawa, H., et al. (2008). Repulsive and attractive semaphorins cooperate to direct the navigation of cardiac neural crest cells. Dev Biol *321*, 251-262.
Tuoc, T.C., Boretius, S., Sansom, S.N., Pitulescu, M.E., Frahm, J., Livesey, F.J., and Stoykova, A. (2013). Chromatin regulation by BAF170 controls cerebral cortical size and thickness. Dev Cell *25*, 256-269.
Ufartes, R., Schwenty-Lara, J., Freese, L., Neuhofer, C., Moller, J., Wehner, P., van Ravenswaaij-Arts, C.M.A., Wong, M.T.Y., Schanze, I., Tzschach, A., et al. (2018). Sema3a plays a role in the pathogenesis of CHARGE syndrome. Hum Mol Genet *27*, 1343-1352.
Waimey, K.E., Huang, P.H., Chen, M., and Cheng, H.J. (2008). Plexin-A3 and plexin-A4 restrict the migration of sympathetic neurons but not their neural crest precursors. Dev Biol *315*, 448-458.
Wang, J., Xiao, Y., Hsu, C.W., Martinez-Traverso, I.M., Zhang, M., Bai, Y., Ishii, M., Maxson, R.E., Olson, E.N., Dickinson, M.E., et al. (2016). Yap and Taz play a crucial role in neural crest-derived craniofacial development. Development *143*, 504-515.
Yan, Z., Wang, Z., Sharova, L., Sharov, A.A., Ling, C., Piao, Y., Aiba, K., Matoba, R., Wang, W., and Ko, M.S. (2008). BAF250B-associated SWI/SNF chromatin-remodeling complex is required to maintain undifferentiated mouse embryonic stem cells. Stem Cells *26*, 1155-1165.
Ye, Q., Jiang, J., Zhan, G., Yan, W., Huang, L., Hu, Y., Su, H., Tong, Q., Yue, M., Li, H., et al. (2016). Small molecule activation of NOTCH signaling inhibits acute myeloid leukemia. Sci Rep *6*, 26510.
York, J.R., Yuan, T., Lakiza, O., and McCauley, D.W. (2018). An ancestral role for Semaphorin3F-Neuropilin signaling in patterning neural crest within the new vertebrate head. Development *145*.
