# Supplementary material for: Critical role of the BAF chromatin remodeling complex during murine neural crest development
Source: PLoS Genet. 2021 Mar 22;17(3):e1009446. doi: 10.1371/journal.pgen.1009446 (PMC8016319; doi:10.1371/journal.pgen.1009446)
Supplement: Attachment — Submitted filename: Response to Reviewers.pdf [file pgen.1009446.s015.pdf]

Reviewer #1:

Comment: The authors have made considerable revisions which have improved the manuscript. The new apoptotic data at E10.5 helps to explain the smaller pharyngeal arches in the mutants at this stage and prefaces the ensuing defects or agenesis of neural crest cell derivatives. The smaller pharyngeal arches are also evident in the 2H3 stained E10.5 embryos. One of the surprising outcomes from the RNA-seq data and subsequent bioinformatic and pathway analyses is the presence of neural crest cell pre-EMT and delamination as major pathways involving differentially expressed genes. Considering the data was obtained from neural crest cells in E11.25 embryos there is a bit of a temporal disconnect since these events in neural crest development take place from E8.5-9.5 and the BAF155/170 mutants don't exhibit an EMT/delamination failure phenotype. The authors should at least comment on this temporal developmental discrepancy as well as the phenotypic discrepancy. The phenotypic discrepancy calls to mind a reported limitation of the Wnt1-Cre line which has been suggested to not be active early enough to fully explore gene function in EMT in mice (Barriga et al 2015) so perhaps this is why the authors observe more survival and differentiation defects in their mutants. Overall however, the manuscript contains a lot of novel interesting data about the requirement for proper epigenetic regulation during neural crest cell development, particularly with respect to craniofacial and cardiac development.

Response: *We would like to thank the reviewer for his/her positive evaluation of our manuscript. We agree with the reviewer. To address this comments, we have removed the Pre-EMT and Delamination heat map from the RNAseq data as the genes listed in this category are not solely involved in EMT/Delamination process but also control other aspects of neural crest development as indicated in Figure 6. For example, Sox5, 9 and 10 also belong to neural crest-specification cluster. Msx1 is implicated in OFT development. Pdgfra and Pdgfrb regulate proliferation and differentiation of NCCs and modulate craniofacial development. Figure 6 has been revised accordingly.*

Minor Comment: The Pax3 and Wnt1 Cre lines are described as being active in neural crest cells. This is an oversimplification and technically not correct. For example, Wnt1-Cre is active in the dorsal neuroepithelium which encompasses the territory from which neural crest cells are derived, but Wnt1 is however not expressed in neural crest cells. Pax3 is a little bit different but it's full expression pattern should be described to help the reader understand the phenotype.

Response: *We have described both Pax3 and Wnt1 cre lines with more details as suggested by the reviewer.*

Minor Comment: Page 10. The authors note the number of SM22a cells were significantly decreased. No statistics were provided however, hence significant should be avoided. Considerably decreased or visibly decreased would be more accurate in the absence of statistics.

Response: *We have revised the text on page 10 to "considerably decreased" as suggested by the reviewer.*

Reviewer #2:

Comment: Authors have satisfied all previous comments and concerns from this reviewer. No additional comments are noted.
Response: *We would like to thank the reviewer for his/her positive evaluation of our manuscript.*

Reviewer #3:

Comment: The authors have significantly revised the manuscript and addressed all of my concerns.
Response: *We would like to thank the reviewer for his/her positive evaluation of our manuscript.*
